# Hyperbrain features of team mental models within a juggling paradigm: a proof of concept

**DOI:** 10.7717/peerj.2457

**Published:** 2016-09-20

**Authors:** Edson Filho, Maurizio Bertollo, Gabriella Tamburro, Lorenzo Schinaia, Jonas Chatel-Goldman, Selenia di Fronso, Claudio Robazza, Silvia Comani

**Affiliations:** 1 BIND–Behavioral Imaging and Neural Dynamics Center, University “G. d’Annunzio” of Chieti–Pescara, Chieti, Italy; 2 School of Psychology, University of Central Lancashire, Preston, Lancashire, United Kingdom; 3 Department of Medicine and Aging Sciences, University “G. d’Annunzio” of Chieti-Pescara, Chieti, Italy; 4 Department of Neurology, Casa di Cura Privata Villa Serena, Città Sant’Angelo, Italy; 5 Department of Neuroscience, Imaging and Clinical Sciences, University “G. d’Annunzio” Chieti-Pescara, Chieti, Italy

**Keywords:** Hyperscanning, Juggling paradigm, Shared mental models, Complementary mental models, Functional connectivity, Graph theory analysis, Social neuroscience, Team mental models

## Abstract

**Background:**

Research on cooperative behavior and the social brain exists, but little research has focused on real-time motor cooperative behavior and its neural correlates. In this proof of concept study, we explored the conceptual notion of shared and complementary mental models through EEG mapping of two brains performing a real-world interactive motor task of increasing difficulty. We used the recently introduced participative “juggling paradigm,” and collected neuro-physiological and psycho-social data. We were interested in analyzing the between-brains coupling during a dyadic juggling task, and in exploring the relationship between the motor task execution, the jugglers’skill level and the task difficulty. We also investigated how this relationship could be mirrored in the coupled functional organization of the interacting brains.

**Methods:**

To capture the neural schemas underlying the notion of shared and complementary mental models, we examined the functional connectivity patterns and hyperbrain features of a juggling dyad involved in cooperative motor tasks of increasing difficulty. Jugglers’ cortical activity was measured using two synchronized 32-channel EEG systems during dyadic juggling performed with 3, 4, 5 and 6 balls. Individual and hyperbrain functional connections were quantified through coherence maps calculated across all electrode pairs in the theta and alpha bands (4–8 and 8–12 Hz). Graph metrics were used to typify the global topology and efficiency of the functional networks for the four difficulty levels in the theta and alpha bands.

**Results:**

Results indicated that, as task difficulty increased, the cortical functional organization of the more skilled juggler became progressively more segregated in both frequency bands, with a small-world organization in the theta band during easier tasks, indicative of a flow-like state in line with the neural efficiency hypothesis. Conversely, more integrated functional patterns were observed for the less skilled juggler in both frequency bands, possibly related to cognitive overload due to the difficulty of the task at hand (reinvestment hypothesis). At the hyperbrain level, a segregated functional organization involving areas of the visuo-attentional networks of both jugglers was observed in both frequency bands and for the easier task only.

**Discussion:**

These results suggest that cooperative juggling is supported by integrated activity of specialized cortical areas from both brains only during easier tasks, whereas it relies on individual skills, mirrored in uncorrelated individual brain activations, during more difficult tasks. These findings suggest that task difficulty and jugglers’ personal skills may influence the features of the hyperbrain network in its shared/integrative and complementary/segregative tendencies.

## Introduction

There is empirical evidence indicating that the neocortex evolved to meet the greater memory requirements of complex social environments (Dunbar, 1998; Dunbar, 2009). In his classic article on the “social brain,” Dunbar (1998) elegantly demonstrated that the information-processing capacity of different species is linearly related to their ability to establish cooperative social groups. Thus, it was the ability to manipulate socio-cognitive information that allowed our ancestors to defend against competing species, while accumulating numerous resources such as food supplies and territorial dominance. Importantly, given that individuals’ ability to manipulate socio-cognitive information is finite, researchers in social neuroscience have used a “perturbational approach,” through tasks of increasing difficulty, to determine the brain’s ability to manipulate (not simply store) information on cognitive and motor tasks (Dunbar, 1998; Kelso, 2012; Massimini et al., 2009). For instance, increasing the difficulty of a task (e.g., juggling with an increased number of balls) has been used to determine the brains’ ability to remain in an “expert” parallel processing mode rather than a “novice” step-by-step mode (Ericsson & Ward, 2007). Although research on cooperative behavior and the social brain exists, there is little, if any, research on real-time motor cooperative behavior (Dumas et al., 2014; Schilbach et al., 2013; Tognoli, 2008). To date, most studies are based on a passive paradigm and observational tasks (De Jaegher & Di Paolo, 2013; Konvalinka & Roepstorff, 2012).

It is important to note that the need for additional studies based on an interactive, rather than passive, paradigm has been acknowledged in the literature (Chatel-Goldman et al., 2013; Di Paolo & De Jaegher, 2012; Konvalinka & Roepstorff, 2012; Schilbach et al., 2013; Tognoli, 2008). Specifically, scholars have argued that it is important to study interactive tasks (i.e., information flows bidirectionally between two or more involved systems) in an ecological environment rather than observational and diachronic tasks (i.e., information flows unidirectionally from an active to a disengaged system) in a laboratory setting (Di Paolo & De Jaegher, 2012; Konvalinka & Roepstorff, 2012). The use of an ecological environment is important because social cognition is fundamentally different when people interact, as opposed to when people merely observe one another (Bahrami & Frith, 2011; Tognoli, 2008). Observing someone or interacting with a computer avatar in a laboratory setting is different from interacting with a person in an ecological, real-world task. Interacting with another individual goes beyond mere observation or reacting to a computer algorithm and involves the utilization and development of shared and complementary mental models in the definition of a “we-space” (Schilbach et al., 2013).

Through means of these shared or complementary mental models, working teams (sports, musicians in orchestras, medical surgical team) are able to achieve high-level spatio-temporal coordination and consequently optimal performance (Filho & Tenenbaum, 2012; Mohammed, Ferzandi & Hamilton, 2010; Salas et al., 2006). To this extent, previous research on real-time cooperative motor tasks has shown that inter-personal spatio-temporal coordination tends to rely on synchronized individual neurophysiological responses, such as cardiac, respiratory and cortical signals (Filho et al., 2016; Lindenberger et al., 2009; McFarland, 2001; Müller & Lindenberger, 2011). Recently, for instance, Müller, Sänger & Lindenberger (2013) observed frequency dependent inter-brain synchrony in two guitar dyads. Cooperative dyads complement each other’s actions to achieve spatio-temporal coordination (Di Paolo & De Jaegher, 2012). In this respect, Reed et al. (2006) noted that people performing a motor shared dyadic task developed complementary schemas so that “one member took on early parts of the motion and the other late parts” (p. 2109).

Although the notion of shared and complementary mental models has been widely examined in the applied social psychology literature (for reviews see Filho & Tenenbaum, 2012; Mohammed, Ferzandi & Hamilton, 2010), neurophysiological studies have yet to uncover the spatio-temporal neural patterns underlying these conceptual constructs. In this regard, Tognoli & Kelso (2014a) expanded the classical coordination dynamics framework and posited that a human brain’s meta-stability framework can be used as praxis to study shared and complementary coordination between brains. Specifically, various scholars (Kelso, 2012; Kelso, Dumas & Tognoli, 2013; Tognoli & Kelso, 2009; Tognoli & Kelso, 2014b) have noted that integrative and segregative tendencies coexist in the brain, thereby allowing for greater functional flexibility.

To better understand the brain’s spatio-temporal dynamics during social interactions, neuroscientists have suggested the use of hyperbrain analysis of simultaneous EEG recordings (Babiloni et al., 2007a; Babiloni et al., 2007b; Fallani et al., 2010). More recently, scholars have investigated network coherence between two brains engaged in joint action using Graph Theory (Sänger, Müller & Lindenberger, 2013; Schilbach et al., 2013). To this extent, measures of functional connectivity and indices of functional organization can be used to describe the topology and characterize the spatio-temporal efficiency (in terms of segregation and integration tendencies) of hyperbrain networks during joint action (see Bullmore & Sporns, 2009; Tognoli & Kelso, 2009; Tognoli & Kelso, 2014b). In the present study, we used coherence analysis and graph measures in the theta and alpha frequency bands to explore segregative and integrative tendencies in between-brains coupling during a dyadic juggling task of increased difficulty. The alpha and theta frequency bands have been used to study skilled performance in visual motor tasks, which are often characterized by a relaxed yet focused mental state (Klimesch, 1999; Yarrow, Brown & Krakauer, 2009). Alpha power has been related to somatosensory information processing, whereas theta power has been associated with focused attention (Baumeister et al., 2008; Cheng et al., 2015; Comani et al., 2014). Both frequency bands have been demonstrated to be directly related to cognitive and memory performance (Klimesch, 1999), fundamental functions of attention (i.e., suppression and selection, see Foxe & Snyder, 2011; Klimesch, 2012), optimal attentional engagement (Kao, Huang & Hung, 2013), and brain proficiency (Bertollo et al., 2016). Thus, coherence analysis of these two frequency bands allows for a representative assessment of the information processing dynamics implicated in skilled motor performance.

In addition to neurophysiological methods, studies on between-brains communication should also consider whether the joint action between individuals carries “historicity” elements (Di Paolo & De Jaegher, 2012; Schilbach et al., 2013; Teufel, Fletcher & Davis, 2010). Historicity elements refer to previous interactions in performing a given task together, which in turn can influence present and future interpersonal brain and body dynamics. For example, in a dual-EEG study Yun, Watanabe & Shimojo (2012) observed increased interpersonal synchrony involving both unconscious movements and neural activities after cooperative motor (fingertip) interaction. It follows that previous interactions are expected to foster the development of shared and complementary mental schemas, and influence group cohesion and efficacy beliefs (Filho, Tenenbaum & Yang, 2015; Peterson et al., 2000). Between-brains studies should also assess psychological states that may alter the quality of a given social interaction. In this regard, extant research in applied psychology has shown that affective states influence group dynamics and performance in both cognitive and motor tasks (Hanin, 2007; Robazza et al., 2016; Tenenbaum et al., 2013), and each performer can show idiosyncratic perceived control and hedonic tone (Medeiros Filho, Moraes & Tenenbaum, 2009; Robazza et al., 2016).

In this proof of concept study, we were interested in further examining the conceptual notion of shared (collective task knowledge that team members bring to a situation) and complementary (idiosyncratic task knowledge that team members bring to a situation) mental models through EEG mapping of two brains performing a real-world interactive motor task of increasing difficulty. To this aim, we used a recently introduced participative paradigm, the “juggling paradigm” (Filho et al., 2015), that employs cooperative dyadic juggling as a platform to capture peripheral (e.g., skin conductance, breathing and heart rates, electromyographic signals) and central neuropsychophysiological (e.g., functional connectivity within and between brains) markers underlying the conceptual notion of team mental models (TMM). Furthermore, to minimize historicity effects we selected two jugglers with no previous history of juggling together.

According to the “juggling paradigm,” wherein psycho-social factors are proposed as moderators of team-level interaction in cooperative juggling (Filho et al., 2015), we also collected data on psycho-social variables, in agreement with the notion that affective and cognitive states influence social interactions (Oullier et al., 2008; Teufel, Fletcher & Davis, 2010). In particular, we assessed arousal and pleasantness levels as these variables underlie the notion of core affect, and represent individuals’ subjective assessment about their overall psychological state (Russell, 1979; Russell, 1980; Russell, Weiss & Mendelsohn, 1989). We also collected data on attentional strategies to assess how the jugglers changed between dissociative (unrelated to the task at hand) and associative (related to the task at hand) attentional strategies throughout the juggling task (for review, see Brick, MacIntyre & Campbell, 2014). In this respect, previous research has suggested that people tend to adopt an associative attentional focus, directing attention inwards, when exposed to tasks of increasing difficulty (Tenenbaum, 2005). Furthermore, attentional focus directed at “core components of action” (i.e., task relevant focus; see Bortoli et al., 2012) has been shown to elicit functional performance states (Bertollo et al., 2015), while excessive attentional focus can lead to poor performance (see Beilock et al., 2002). Finally, we gathered responses on self-efficacy and other efficacy beliefs as task-related confidence of one’s self and others is a major predictor of performance in various domains of human interest (Bandura, 1997).

Coherently with recent efforts in social neuroscience (Sänger, Müller & Lindenberger, 2013), we used global measures of functional connectivity and efficiency of the jugglers’ hyperbrain network to characterize their coupled functional organization, while considering the outcome of these analyses in the light of their subjective psycho-social states (i.e., arousal, pleasantness, attention, self-efficacy and other efficacy). In this context, we first hypothesized that: (H1) the jugglers’ affective and cognitive states would vary with respect to task difficulty. This is consistent with previous research in performance psychology whereby task difficulty has been found to moderate the affective and cognitive responses among individuals from varying skill levels (see Hanin, 2007; Tenenbaum, 2005; Tenenbaum et al., 2013). Second, we hypothesized that: (H2) the jugglers’ affective and cognitive states would correlate with one another across tasks, akin to previous theoretical and empirical accounts that an overlap of psychophysiological responses tends to occur in joint motor action (Filho et al., 2015; Filho et al., 2016; Yun, Watanabe & Shimojo, 2012). Third, congruent with recent hyperbrain research in music and card playing (Babiloni et al., 2007b; Fallani et al., 2010; Sänger, Müller & Lindenberger, 2013), we hypothesized that: (H3) between-brains coupling would occur during the dyadic juggling task. Specifically, we expected that this between-brains coupling would involve areas specialized for attentional control and information processing, which are included in the dorsal (top-down visuo-spatial) and ventral (bottom-up re-orienting) frontoparietal systems. More specifically, these areas are the intraparietal sulcus (IPS) and frontal eye fields (FEF) of each hemisphere, the temporoparietal junction (TPJ) and ventral frontal cortex (VFC), and visual cortex (for a review, see Vossel, Geng & Fink, 2014).

Our fourth hypothesis focused on the type of efficiency established in the hyperbrain network during the execution of dyadic juggling of increasing difficulty. In line with the concept of *neural efficiency*, which purports that skilled performance relies on an automatic processing mode characterized by a decrease in global cortical activation (see Grabner, Neubauer & Stern, 2006), we expected that: (H4) an automated execution of a motor task would depend on both the jugglers’ skill level, and the difficulty level of the task. In particular, as skilled performance is characterized by lower brain activation compared to unskilled performance, we expected to find a more segregated brain functional organization in the more skilled juggler across tasks, particularly in the easier task, and a more integrated brain functional organization in the less skilled juggler, particularly in the more difficult tasks.

## Materials and Methods

### Design

**Figure 1:**
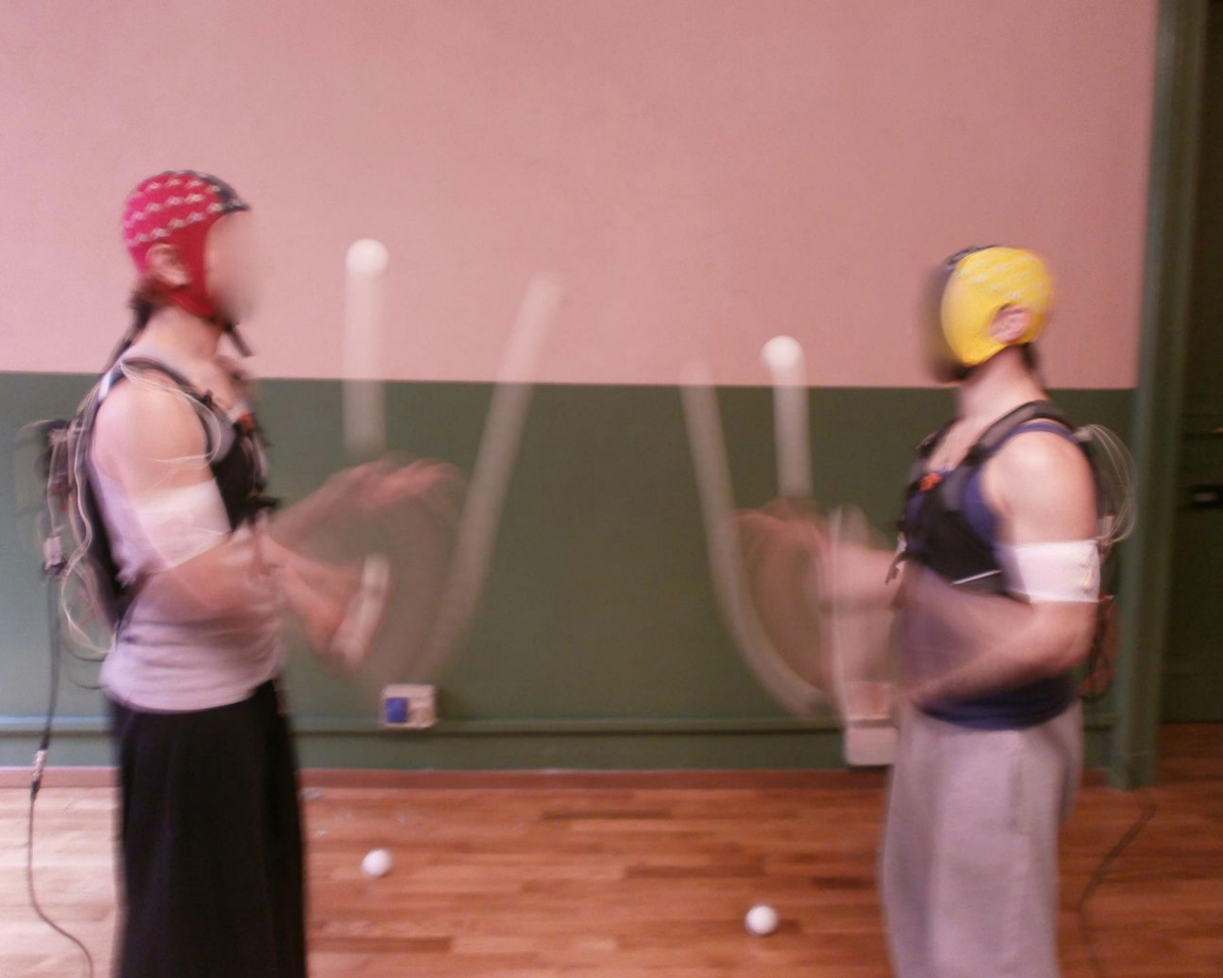
Example of dyadic juggling execution using portable EEG systems. The different colors for the EEG caps refer to their different sizes.

We conducted a proof of concept study using a juggling dyad. Figure 1 is an example of dyadic juggling. To test our working hypotheses, we combined subjective psychological assessments and objective physiological recordings. There is a general agreement that case studies should rely on multiple sources of information and combine both subjective and objective measures (Tate et al., 2014). Recently, single-case designs and small-n studies using juggling as a platform have been suggested as a valid ecological approach to study joint-coupling of peripheral and central physiological responses (Filho et al., 2015; Filho et al., 2016).

### Participants

Two right handed jugglers able to consistently juggle up to six balls participated in the study. The participants were recreational jugglers who practiced juggling for approximately 6 h a week in a circus school in Southeastern Italy. Prior to participation, the jugglers were educated on the purposes of the study, and upon voluntary agreement they signed an informed consent sheet. At the time of the study, Juggler 1 (J1) was 29 years old and had nine years of juggling experience. Juggler 2 (J2) was 20 years old and had three years of juggling experience. The juggler’s practice schedule did not involve systematic training in a dyadic setting. The study was conducted in accordance with the declaration of Helsinki and was approved by the local Ethics Committee (ref. n. 10-21/05/2015).

### Procedure and tasks

We acquired synchronous EEG signals from the two jugglers while gathering their self-reports. EEG recordings can provide an ecological approach to study real-time social interaction (Konvalinka & Roepstorff, 2012; Sänger, Müller & Lindenberger, 2013), as opposed to constrained settings such as functional Magnetic Resonance Imaging (fMRI) or Magnetoencephalography (MEG). Additionally, self-reports are important tools to gain insight on individuals’ affective responses during social interactions (Bandura, 2006), and support phenomenological investigations of inter-subjectivity (Froese & Gallagher, 2012).

We collected data while the participants were performing a cooperative dyadic juggling task (i.e., between-brains condition), as we were interested in studying between-brains interactions. Furthermore, we adopted a “perturbational approach” to examine whether the ability of the jugglers’ brains to manipulate information changed as a function of task difficulty (Dunbar, 1998; Kelso, 2012; Massimini et al., 2009). To this aim, we progressively augmented the difficulty level of the juggling task by increasing the number of balls, which therefore acted as the control parameter of the coupled system under investigation (i.e., the two brains working together). This allowed for the examination of whether the functional topology and efficiency of the hyperbrain network varied based on task difficulty. Of note, the importance of “creating perturbation” is also at the core of the expert performance approach (Ericsson & Ward, 2007; Williams & Ericsson, 2005), which uses challenging tasks to identify the underlying mechanisms of skilled motor performance.

Two peer-debriefing meetings with the jugglers preceded data collection, during which the overarching purposes of the study were explained to the jugglers and the experimental protocol was pilot tested. Based on insights from both jugglers, the well-established “cascade juggling pattern,” the first-learned symmetric pattern in juggling, was used in the study (see Dancey, 2003). Data collection occurred in a spacious athletic gymnasium. It was also established that 2 m distance between the jugglers allowed for optimal amplitude of movement and reliable data collection.

### Experimental protocol

The experimental protocol consisted of dyadic juggling (between-brains condition) performed at four different difficulty levels, established on the basis of the jugglers’ abilities and implemented by increasing the number of balls juggled, as summarized in Table 1. Specifically, in agreement with the “perturbational approach” previously discussed, as well as with as well as with the guidelines on incremental workload tests in psychophysiology research (see Weisman & Zeballos, 2002), the jugglers started with the Easy task and progressed to the Very Hard task. Incremental experimental protocols allow for the identification of how different systems (e.g., bioenergetic, cardiovascular, neural, respiratory) contribute to physical and cognitive performance during the execution of a given task (see Bertuzzi et al., 2013).

**Table 1:** Description of the different phases of the experimental protocol.

Phase	Difficulty level	Number of balls
1	Easy	3
2	Medium	4
3	Hard	5
4	Very hard	6

The between-brains condition was preceded by a baseline assessment, during which the jugglers stood quietly, fixating on a point on the wall in front of them for 3 min. Following the baseline assessment, the jugglers were given a series of familiarization trials until they reported feeling comfortable with the EEG apparatus. This familiarization period lasted approximately 10 min.

The actual between-brains EEG and psychological data acquisition consisted of four phases (see Table 1), each lasting about 5 min. Each phase consisted of a sequence of trials whose average duration decreased as the difficulty level increased. Only trials lasting at least 10 s were retained for further EEG data processing (see section 2.7.2). A trial started when the first ball was thrown in the air, and ended when one ball was dropped. For each trial, J1 and J2 alternated who would launch the first ball. If required, J1 and J2 were allowed to rest and recover from fatigue feelings between subsequent trials and phases. The resting time was decided by the jugglers, in agreement with the notion that fatigue is ultimately voluntarily regulated (Blanchfield et al., 2014). The needed time to complete the experimental protocol was approximately 2 h.

### Psychological data acquisition

Before and after each juggling phase, self-report measures were collected through single-item measures of: (a) arousal and pleasantness, (b) attention, (c) self-efficacy, and (d) other’s efficacy. Importantly, the use of single-item measurements is recommended for scholars collecting data during (rather than prior to or post task assessments) real-time interactions (Bandura, 2006; Kamata, Tenenbaum & Hanin, 2002). Specifically, single-item instruments are less intrusive than multi-item measures in ecological experimental studies (Kamata, Tenenbaum & Hanin, 2002).

#### Arousal and pleasantness levels

A modified version of the Affect Grid was used to measure affect throughout the juggling task. Extensive research supports the notion that core affect is a by-product of two key bipolar affective dimensions: pleasure-displeasure and degree of arousal (for a review, see Russell, 1979; Russell, 1980; Russell, Weiss & Mendelsohn, 1989). The participants were asked to report their perceptions of pleasure on a continuum ranging from 1 (unpleasant) to 9 (pleasant). Similarly, the jugglers rated their arousal levels on a continuum ranging from 1 (sleepiness) to 9 (high arousal).

#### Attention

Attention was measured on a 10-point scale ranging from 0 (e.g., task-unrelated, distracting thoughts or external cues) to 10 (e.g., task-related, juggling performance) throughout the juggling task. This scale was designed to capture a continuum of attentional strategies ranging from 0 (pure dissociation) to 10 (pure association) and has been used in previous research in applied psychology (Basevitch et al., 2011; Razon, Hutchinson & Tenenbaum, 2012; Tenenbaum, 2005).

#### Self-efficacy

Throughout the juggling task, the participants responded to a single-item self-efficacy measure, developed according to Bandura (2006) recommendation for designing task-specific and content relevant measures of efficacy beliefs. Specifically, the participants were asked to rate their confidence on their juggling ability using a Likert scale ranging from 0 to 10, and three verbal anchors for 0 = “cannot do at all;” 5 = “moderately can do;” and 10 = “highly certainly can do.” The probe was: “How confident are you in your ability to successfully juggle with ‘X’ (‘three,’ ‘four,’ ‘five’ or ‘six’) balls?”

#### Other’s efficacy

Other’s efficacy was measured using a single-item, designed in agreement with Bandura (2006) guide for constructing efficacy scales. Throughout the juggling task, the participants were asked to rate their degree of confidence in their partners’ ability to juggle with three, four, five or six balls. The participants used the same Likert scale previously described. The specific probe was: “How confident are you that your juggling partner is able to successfully juggle with ‘X’ (‘three,’ ‘four,’ ‘five’ or ‘six’) balls?”

### Neurophysiological data acquisition and pre-processing

EEG data were recorded via two synchronized EEG systems (eegosports and ASAlab, ANT Neuro B.V., Enschede, Netherlands, respectively mounted on J1 and J2), each using a 32 electrode waveguard cap (ANT Neuro B.V., Enschede, Netherlands). EEG data were continuously recorded with a sampling frequency of 1,024 Hz from 32 Ag/AgCl electrodes positioned over the scalp according to the 10/5 system (Oostenveld & Praamstra, 2001). EEG signals were recorded with the ground electrode in AFz, positioned between Fpz and Fz. The common average reference approach was used, wherein the reference is the average power across all electrodes. Low impedance values were kept for both systems (Z < 10 kΩ). The EEG signals from the two jugglers were synchronized before and after each acquisition phase through a pushbutton trigger signal that was sent simultaneously to the two EEG systems.

EEG signal pre-processing consisted of several steps. First, the raw EEG data were visually inspected to identify electrodes not working properly (M1, M2 and Oz). The signals from these electrodes were removed from further analysis (McMenamin et al., 2010). The resulting individual raw EEG data sets (including 29 EEG signals from the retained 29 electrodes) were segmented according to the four phases of the experimental protocol.

**Figure 2:**
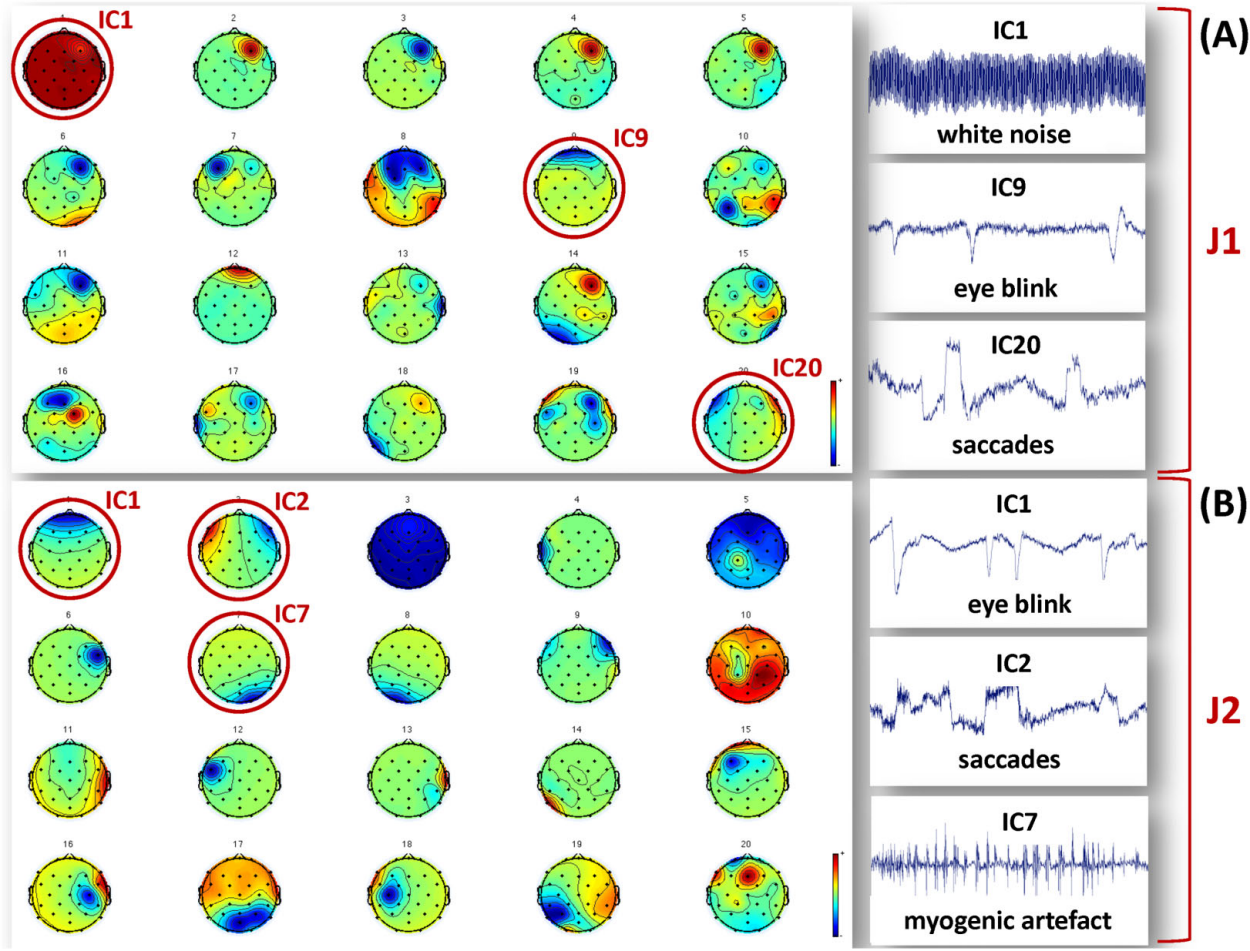
Examples of artefactual ICs separated from the EEG recordings of J1 and J2. Examples of ICs not considered to reconstruct the EEG signals used for analysis are identified by a red circle, and their time course is shown in the figures on the right hand side. (A) ICs separated for J1. The example artefactual ICs regard white noise, eye blinks and saccades. (B) ICs separated for J2. The example artefactual ICs regard eye blinks, saccades and myogenic artifacts.

The segmented raw EEG data were then offline down-sampled to 256 Hz using the ASA software (ANT Neuro B.V., Enschede) to reduce subsequent computational load, notch filtered at 50 Hz to remove power line interference, and bandpass filtered (0.5–100 Hz) to eliminate extreme low and high frequency noise. After applying Principal Components Analysis (PCA) to reduce data dimensionality (Delorme, Westerfield & Makeig, 2007), the InfoMax algorithm implementing Independent Component Analysis (ICA) was used to separate and reject the independent components (ICs) related to biological and environmental artifacts (Bell & Sejnowski, 1995; Lee, Girolami & Sejnowski, 1999). We allowed for a total of 20 ICs, and the artefactual ICs were identified on the basis of their time course, scalp topography and power spectrum (McMenamin et al., 2010). Examples of artefactual components separated for J1 and J2 are shown in Fig. 2. The true EEG signals were reconstructed from the retained ICs. All pre-processing steps were performed using MATLAB (The MathWorks Inc., Natick, MA, USA) and the EEGLab toolbox (http://sccn.ucsd.edu/eeglab).

### Data analysis

#### Psychological factors

We used descriptive line graphs to identify and compare the jugglers’ subjective accounts of arousal, pleasantness, attention, self-efficacy and other’s efficacy. Of note, line graphs are the most commonly used display in case studies and small-n research given its ability to illustrate trend-like relationships over time or across conditions (Tate et al., 2014).

#### Functional connectivity representations

For each juggler and each epoch (i.e., each juggling phase at different difficulty levels), the pre-processed EEG signals were segmented according to the duration of the individual trials (i.e., according to the duration of each dyadic juggling exchange within the samejuggling phase). Given that we were interested in studying the neural patterns of cooperative juggling when it was established and steady before errors or movements occurred and disrupted it, an interval of 4 s was retained from each trial. Each trial started 1 s after action initiation and ended at least 3 s before a ball dropping (i.e., the end of the trial). This approach was used for all trials and experimental conditions in order to correctly compare across difficulty levels. As a result of this methodology, only trials lasting for at least 10 s were retained. Only good quality signals could be used for connectivity and graph analysis, and as such several trials had to be excluded from further analysis as they were heavily affected by noise due to the jugglers’ movements. This occurred mainly during the Hard and Very Hard conditions. As a consequence, only a few intervals could be retained for each experimental condition. Table 2 summarizes the total number and average duration of the trials useful for the definition of the analysis intervals.

**Table 2:** Summary of the total number of useful trials per experimental condition. The average trial duration is also provided in seconds (Mean ± STD).

Difficulty level	Number of balls	Number of trials	Average trial duration
Easy	3	6	30.2 ± 12.4
Medium	4	8	18.5 ± 7.2
Hard	5	11	9.8 ± 2.6
Very hard	6	13	8.4 ± 1.6

With this approach, the EEG recordings of each interval were surely related to steady cooperative juggling, not affected by exceeding noise, and always the same number for both jugglers.

The patterns of individual (within-brain) cortical functional connectivity were estimated for each interval by calculating the coherence across the pre-processed and segmented EEG signals (data were analyzed using Brainwave v0.9.133.1, http://home.kpn.nl/stam7883/brainwave.html). Coherence is a statistical measure that essentially represents the probability of functional correlation between two given signals at a given time instant (or in a given time span) within a given frequency band. In our case, since we retained an array of 29 EEG signals, for each time interval we obtained a (29 × 29) coherence matrix, where each element *c_ij_* represents the coherence between the EEG signals from electrodes *i* and *j*. As we were interested in the visuo-attentional processes occurring during dyadic juggling, two coherence matrices were calculated for each interval: one in the alpha (8–12 Hz) and one in the theta band (4–8 Hz), respectively.

**Figure 3:**
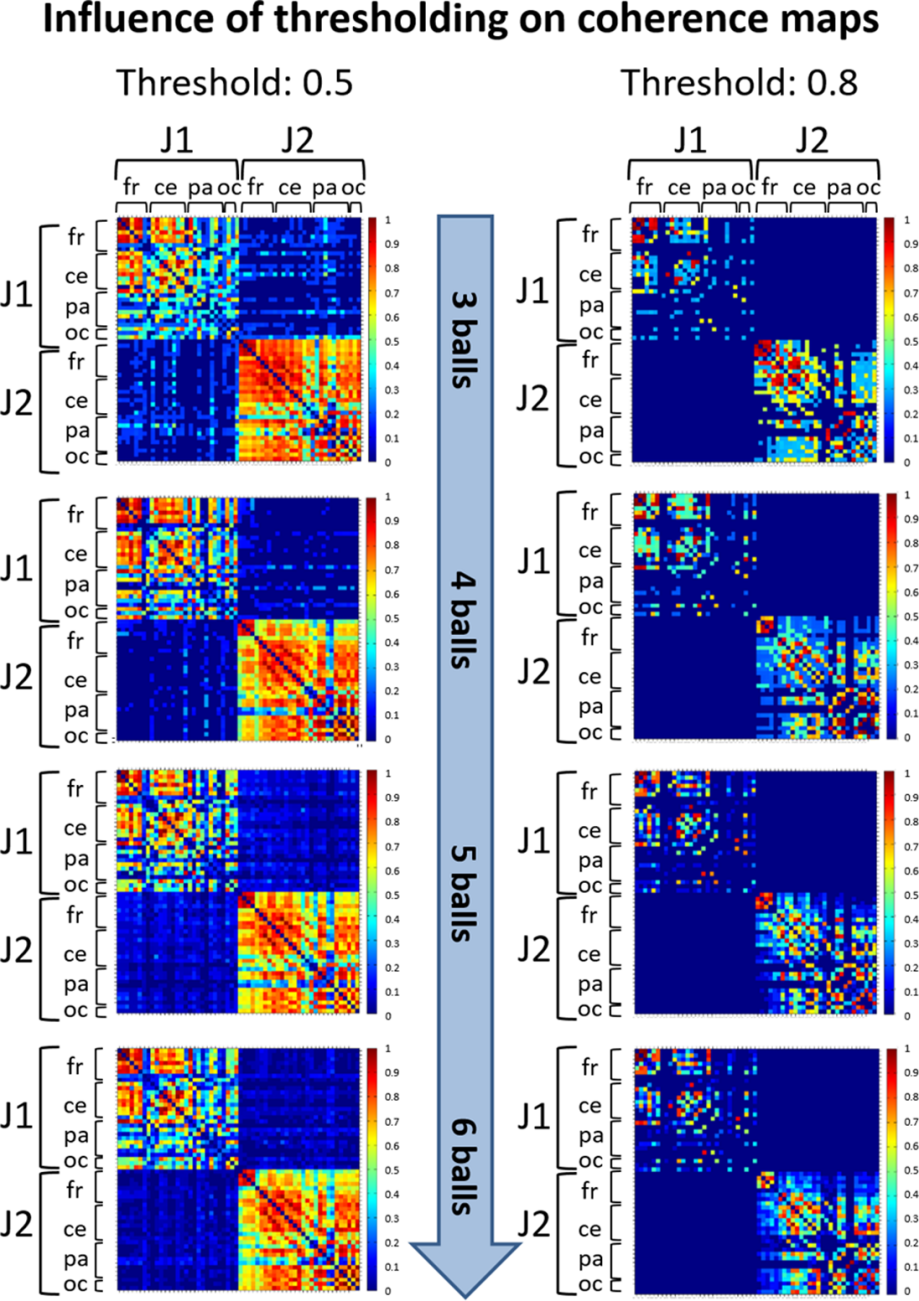
Illustration of coherence maps obtained using two different thresholds at values 0.5 and 0.8 in the alpha band. In these maps the same threshold was used for within-brain and between-brain coherence values. The maps are normalized after thresholding to enhance the differences across the retained functional connections. It can be seen that variation of the threshold value does not change the general direction of observed coherence patterns.

Due to the conductivity properties of the scalp, at any point in time each EEG signal is a linear combination of the activity at each cortical source. Therefore, in studies of coherence, volume conduction and residual artefactual noise can create artificially inflated coherence values between distant electrodes (Nunez et al., 1997). A thresholding procedure is generally applied to retain only higher coherence values that likely correspond to functional connections between pairs of EEG signals. To determine an appropriate threshold, a first judgment call (see APA Publications Communications Board Working Group, 2008) was made based on visual inspection of coherence matrices resulting from thresholding at various values (0.5, 0.6, 0.7 and 0.8). Once we assessed that different thresholds did not influence the observed coherence patterns (see an example in Fig. 3), we selected the thresholds 0.8 and 0.5 for within-brain and between-brain coherence matrices respectively, as these values retained about 15% of top connections in both types of matrices. This estimate was based on the evaluation of a cost function that compares the number of connections retained after thresholding at some value τ with the maximum number of connections that could exist in a network of N nodes (Bullmore & Bassett, 2011). As such, these thresholds offered an ideal trade-off between sensibility (best for lower threshold values) and pattern readability (best for higher threshold values) of the matrices.

The thresholded coherence matrices calculated for all the 4-s time intervals within each epoch and frequency band were then averaged to obtain a mean coherence map representing the individual cortical functional connectivity of one juggler’s brain for the given difficulty level within the considered frequency band. As a result, for each juggler, we had a total of eight individual mean coherence maps, i.e. four maps (as the number of juggling difficulty levels) for each frequency band (the alpha and theta bands).

To estimate the patterns of dyadic (between-brains) cortical functional connectivity, for each epoch and each interval the pre-processed and segmented EEG signals of J1 and J2 were concatenated by electrodes. Therefore, for each interval we had a hyperbrain EEG data set of 58 EEG signals of 4 s duration. To calculate the dyadic (hyperbrain) mean coherence maps, we followed the same procedure described above for the within-brain condition, the only difference being the threshold used. Typically, hyperbrain coherence maps are composed of four quadrants, two of which correspond to the within-brain condition, and two to the between-brains condition. On the basis of the procedure followed to reconstruct the hyperbrain EEG data sets, the upper left and lower right quadrants correspond to the individual coherence maps (within-brain) of the two jugglers, and were considered for further hyperbrain analysis. The other two quadrants (upper right and lower left) refer to the hyperbrain coherence maps representing the functional connections between the two jugglers’ brains. Due to the symmetry of these coherence maps, only one between-brains map was considered for subsequent analysis.

Estimate of the error variance in the point estimates of coherence is required to draw any conclusion on connectivity differences between subjects or conditions. Coherence standard error maps were then calculated using a bootstrapping procedure applied to the coherence values of all channel pairs (within- and between-brains) for each difficulty level and each frequency band. Two hundred bootstrap samples were drawn to estimate the standard errors (Efron & Tibshirani, 1993; Hesterberg et al., 2003).

#### Measures of functional organization

Graph Theory describes the topology of functional brain networks, and characterizes their local and global efficiency in terms of information segregation and integration properties. Within this framework, patterns of functional connections are represented as graphs where the elements of the network, the nodes (in our case the electrodes), are linked with edges that represent the relationships (or functional interactions) between nodes. We used graph theoretical concepts to study the topological features of the within-brain and between-brains functional networks represented by the individual and dyadic mean coherence maps, respectively. For each juggler (or for the dyad), for each juggling phase (i.e., difficulty level) and for each frequency band (i.e., alpha and theta), the cortical functional organization was characterized by means of segregation and integration measures calculated on the related mean coherence maps, where each coherence value (when different from zero) represents the strength of the functional connection between two given cortical sites (identified by the electrodes’ positions).

In the brain, functional segregation refers to specialized information processing that occurs within densely interconnected groups of brain regions (Rubinov & Sporns, 2010). Since coherence maps are calculated in the sensor space, segregated neural processing will be suggested by statistical dependencies between clustered electrodes. We calculated functional segregation by means of the *mean clustering coefficient C*, which is a weighted measure of the prevalence of clustered connectivity around individual nodes (Watts & Strogatz, 1998). High values of *C* indicate that a high number of connections exist among neighboring nodes. When high functional segregation is found in a functional connectivity matrix (such as a mean coherence map), the prevailing functional connections occur across neighboring brain areas.

On the other hand, functional integration in the brain represents the ability to rapidly combine specialized information from distributed brain regions. To estimate how easily different brain regions communicate during dyadic juggling (in both the within-brain and between-brains conditions), we calculated the *characteristic path length L*. This measure is defined as the mean of the minimum path lengths among all node pairs, where the lengths of paths connecting two given nodes estimate the potential for functional integration in the network (Watts & Strogatz, 1998). Low values of *L* (i.e., short paths) indicate a strong potential for integration within the network. High functional integration within a functional connectivity matrix suggests that the brain combines, through long distance functional connections, specialized and densely connected (segregated) areas for a more efficient information processing.

In order to compare the *mean clustering coefficient C* and *characteristic path length L* obtained for the mean coherence maps of the two jugglers, we normalized these values by two values *C_n_* and *L_n_*, which were obtained by averaging the *C* and *L* values of 100 random matrices with the same number of nodes and edges of the given coherence maps. Each of these random matrices was created by uniquely assigning each edge to a node pair with uniform probability (Humphries & Gurney, 2008).

However, highly segregated and highly integrated networks represent two extreme configurations (regular and random, respectively) that are rarely encountered in the adult brain. It has been demonstrated that the functional organization of the human brain has small-world attributes both at rest and during the execution of simple motor tasks (Bassett et al., 2006). A canonical small-world network is organized in highly clustered regions that are linked by long-range connections (Watts & Strogatz, 1998). A small-world network exhibits an optimal balance of functional integration and segregation (Rubinov & Sporns, 2010; Sporns, Tononi & Edelman, 2000). It is therefore highly efficient at both local and global levels, and is more capable of adapting to changing task demands (Bassett et al., 2006). In formal terms, a small-world network has a similar *characteristic path length L* but a greater *mean clustering coefficient C* than an equivalent random graph with the same number of nodes and edges (Bollobas, 2001; Watts & Strogatz, 1998).

To investigate whether the jugglers’ functional brain networks showed a small-world organization in the within-brain and/or between-brains conditions, we calculated a quantitative metric of “small-world-ness” introduced by Humphries & Gurney (2008), and defined as:

(1) $$SW = {{C/{C_n}} \over {L/{L_n}}}$$

where *C* and *L* are the *mean clustering coefficient* and the *characteristic path length* of the mean coherence map (individual or hyperbrain), and *C_n_* and *L_n_* are the normalized *C* and *L* obtained from 100 random matrices, as described above. With this approach, the analyzed functional network (i.e., the given coherence map) will have a small-world organization only if *SW* is greater than the unit (Humphries & Gurney, 2008). All graph metrics were calculated using Brainwave v0.9.133.1 (http://home.kpn.nl/stam7883/brainwave.html).

## Results

### Psychological factors

Descriptive and Spearman’s non-parametric correlational analyses were conducted to assess whether the jugglers’ affective and cognitive states: (1) changed as a function of task difficulty (H1), and (2) correlated with one another across tasks (H2).

#### Arousal and pleasantness levels

**Figure 4:**
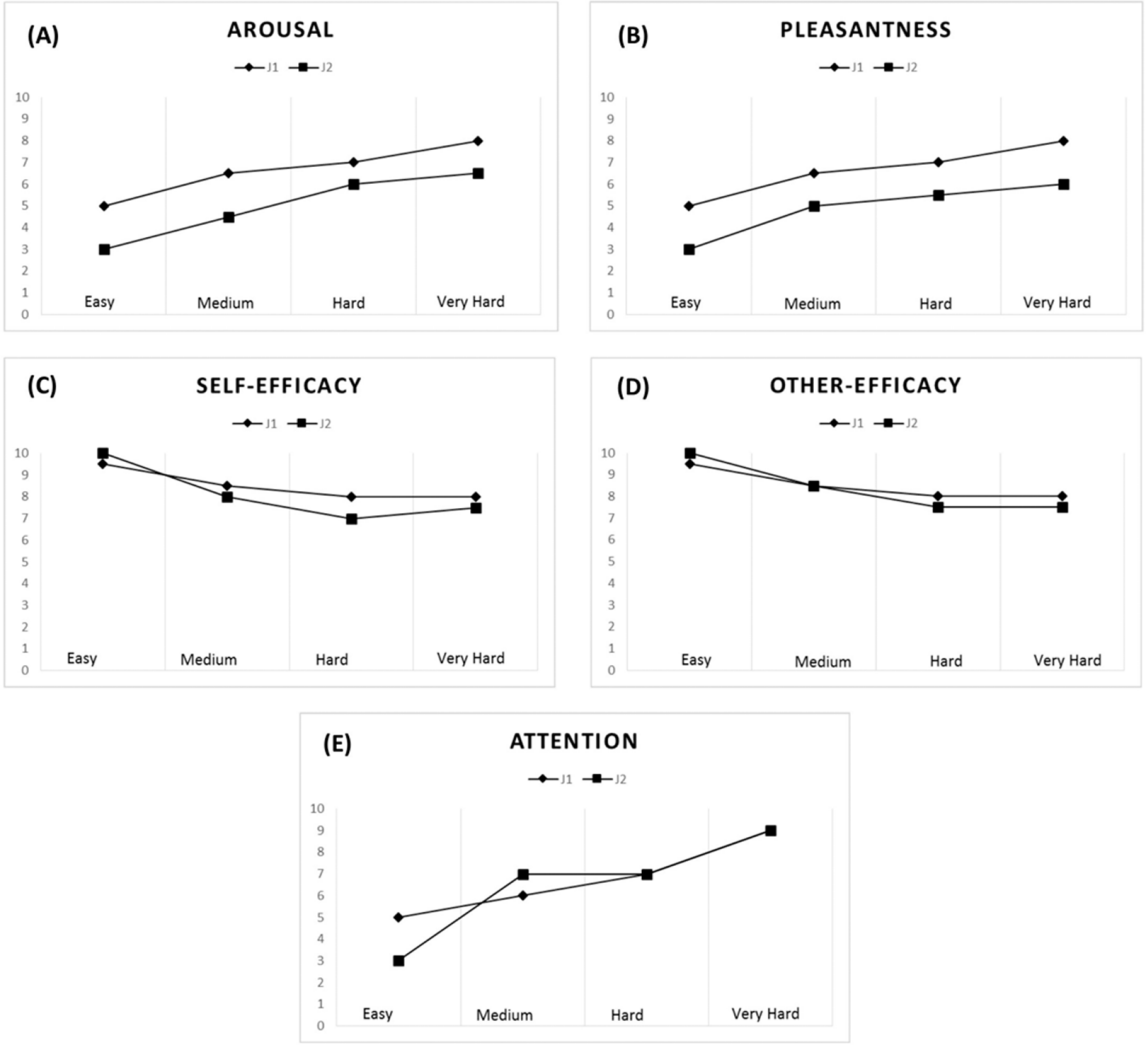
Psychological data for J1 and J2. Arousal (A), pleasantness (B), self-efficacy (C), other’s efficacy (D), and attentional levels for the dissociation-association continuum (E) according to task difficulty.

A strong correlation pattern emerged for the jugglers’ arousal (r_*s*_ = 0.91) and pleasantness levels (r_*s*_ = 0.82) across all tasks. Overall, an increase in task difficulty elicited higher levels of arousal and was perceived as being more pleasant by both jugglers (see Figs. 4A and 4B). With respect to the Easy task (*M* = 4.00, *SD* = 1.15), the jugglers reported increased levels of arousal for the Medium (*M* = 5.50, *SD* = 1.73, *d* = 1.02), Hard (*M* = 6.50, *SD* = 0.58, *d* = 2.75), and Very Hard tasks (*M* = 7.25, *SD* = 0.96, *d* = 3.06). Similarly, in comparison with the Easy task (*M* = 4.00, *SD* = 1.15, *d* = 1.45), the jugglers reported higher levels of pleasantness for the Medium (*M* = 5.75, *SD* = 1.26, *d* = 1.45), Hard (*M* = 6.25, *SD = 0.96*, *d* = 2.12), and Very Hard tasks (*M* = 7.00, *SD* = 1.41, *d* = 2.32).

#### Self-efficacy and other’s efficacy beliefs

The jugglers’ efficacy beliefs were not significantly correlated (r_*s*_ = 0.53), although other’s efficacy beliefs was found to be significantly correlated (r_*s*_ = 0.71). Efficacy beliefs also decreased with task difficulty (Figs. 4C and 4D). With respect to the Easy task (*M* = 9.75, *SD* = 0.50), the jugglers reported lower self-efficacy beliefs for the Medium (*M* = 8.25, *SD* = 1.26, *d* = −1.57), Hard (*M* = 7.50, *SD* = 1.0, *d* = −2.86), and Very Hard tasks (*M* = 7.75, *SD* = 0.50, *d* = −4.0). Similarly, in comparison with the Easy task (*M* = 9.75, *SD* = 0.50,), the jugglers reported lower levels of other’s efficacy beliefs for the Medium (*M* = 8.50, *SD* = 1.29, *d* = −1.27), Hard (*M* = 7.75, *SD* = 0.50, *d* = −4.0), and Very Hard tasks (*M* = 7.75, *SD* = 0.50, *d* = −4.0). Of note, the jugglers reported the same level of other’s efficacy beliefs for the Hard and Very Hard tasks.

#### Attention

The jugglers’ attentional focus was significantly related across tasks (r_*s*_ = 0.78). Generally, as the task difficulty increased, the jugglers’ adopted a more associative attentional focus (Fig. 4E). With respect to the Easy task (*M* = 4.25, *SD* = 1.50), the jugglers reported increased levels of arousal for the Medium (*M* = 6.00, *SD* = 1.41, *d* = 1.2), Hard (*M* = 7.0, *SD* = 0, *d* = 2.59), and Very Hard tasks (*M* = 8.25, *SD* = 0.96, *d* = 3.18). This trend of an increase in attentional association as a function of task difficulty corroborates the notion that harder physical tasks tend to elicit an associative focus response (see Tenenbaum, 2005).

Altogether, these findings (1) suggest that the jugglers affective-cognitive states overlapped and co-varied greatly throughout the experiment; and (2) offer support for our experimental manipulation and the notion that affective-cognitive states change as a function of task difficulty, akin to the “perturbational approach” (Massimini et al., 2009).

### Neurophysiological data

#### Functional connectivity representations

##### Within-brain analysis

**Figure 5:**
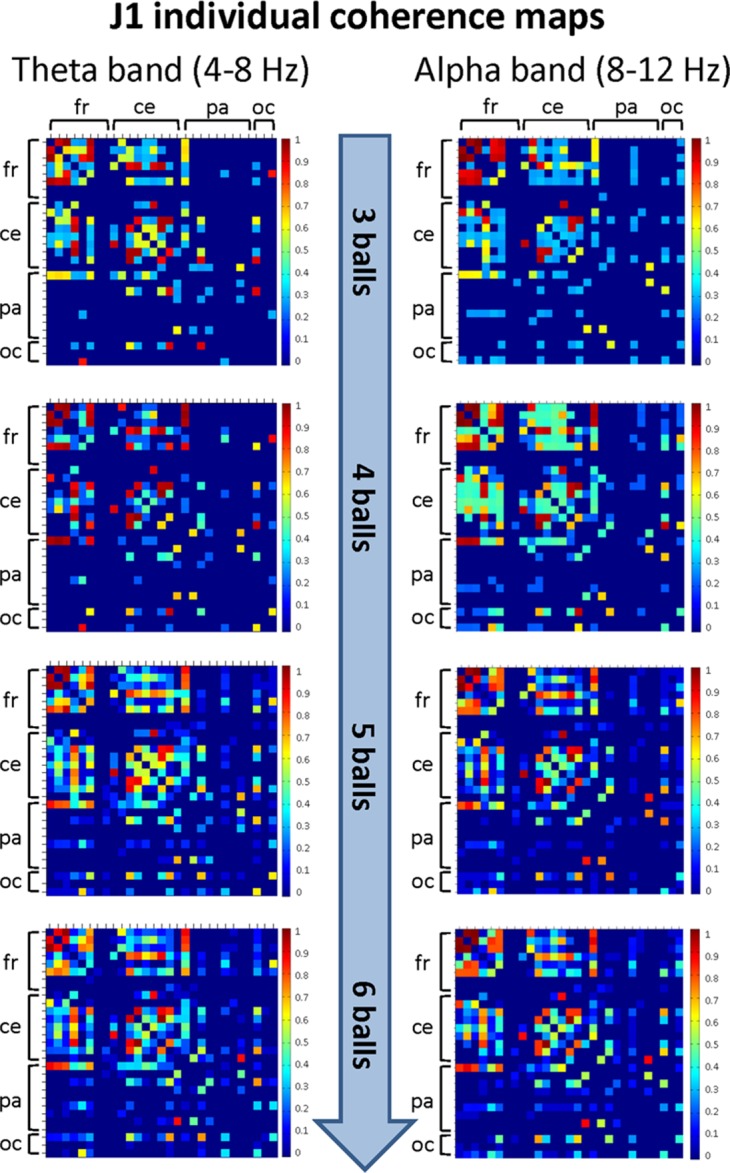
Individual (within-brain) coherence maps for J1 in the theta and alpha frequency bands and for the four difficulty levels (3, 4, 5 and 6 juggled balls). The maps are normalized after thresholding to enhance the differences across the retained functional connections. In each coherence map, the electrodes are grouped according to the cortical areas (frontal, central, parietal and occipital), and are listed in the following order from top to bottom and from left to right: “fr” includes the frontal electrodes Fp1, Fpz, Fp2, F7, F3, Fz, F4, F8; “ce” includes the central electrodes FC5, FC1, FC2, FC6, T7, C3, Cz, C4, T8; “pa” includes the parietal electrodes CP5, CP1, CP2, CP6, P7, P3, Pz, P4, P8; “oc” includes the occipital electrodes POz, O1, O2.

**Figure 6:**
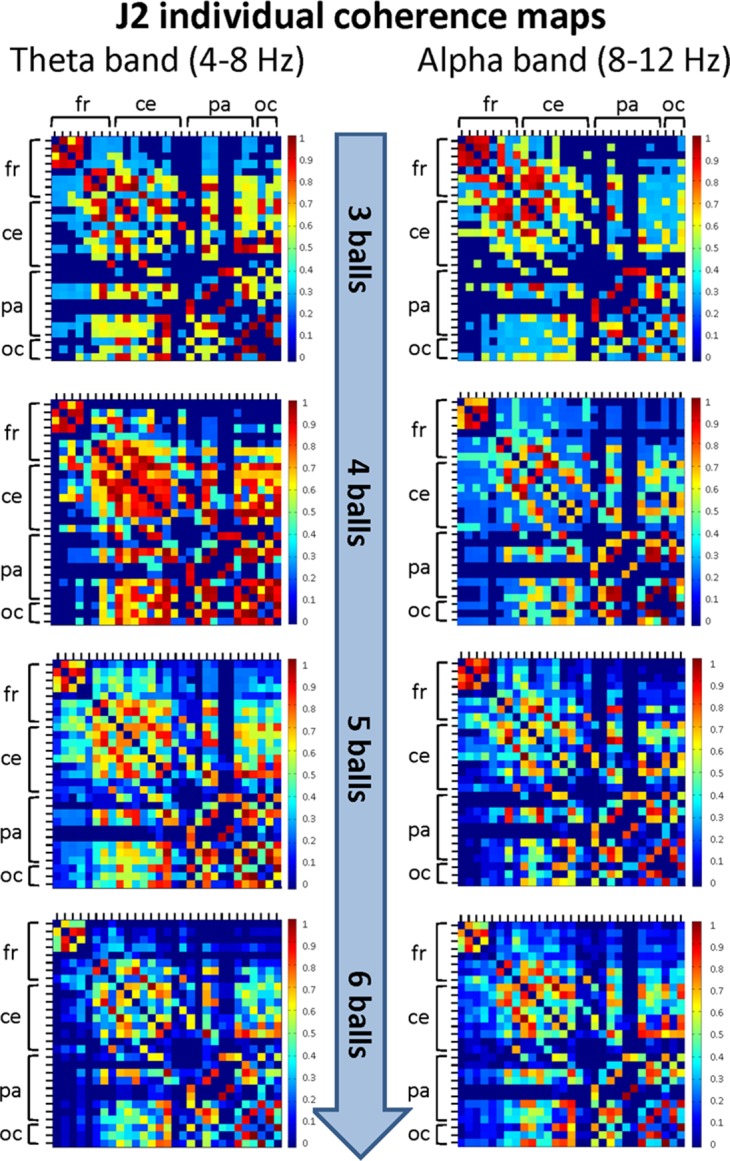
Individual (within-brain) coherence maps for J2 in the theta and alpha frequency bands and for the four difficulty levels (3, 4, 5 and 6 juggled balls). The maps are normalized after thresholding to enhance the differences across the retained functional connections. In each coherence map, the electrodes are grouped according to the cortical areas (frontal, central, parietal and occipital), and are listed in the following order from top to bottom and from left to right: “fr” includes the frontal electrodes Fp1, Fpz, Fp2, F7, F3, Fz, F4, F8; “ce” includes the central electrodes FC5, FC1, FC2, FC6, T7, C3, Cz, C4, T8; “pa” includes the parietal electrodes CP5, CP1, CP2, CP6, P7, P3, Pz, P4, P8; “oc” includes the occipital electrodes POz, O1, O2.

The mean coherence maps representing the individual (within-brain) functional connectivity at cortical level for J1 and J2 for each difficulty level in the alpha and theta bands are summarized in Figs. 5 and 6, respectively. Estimates of coherence standard error were small (all values < 5% of mean coherence except one: J1, alpha band, Easy task) for all within-brain coherence maps (Table 3). We observed that, in both bands, the mean coherence maps of J1 showed a more clustered pattern of connections mainly involving the frontal and central areas. As task difficulty increased, the intensity of the functional connections in the alpha band increased, and also the parietal and occipital areas were included in the functional networks, although to a minor extent. On the other hand, in the theta band the highest coherence values were detected for the Medium difficulty level (4 balls).

**Table 3:** Estimate of coherence standard error for the theta and alpha bands obtained through bootstrapping procedure for the difficulty levels.

	Mean value of standard error				Percentage of mean value %			
*Theta band*								
Difficulty level	3 balls	4 balls	5 balls	6 balls	3 balls	4 balls	5 balls	6 balls
Juggler J1	0.03	0.02	0.02	0.03	3.49	2.93	2.48	2.57
Juggler J2	0.05	0.04	0.03	0.02	4.66	4.26	2.56	2.49
Hyperbrain	0.03	0.01	0.01	0.01	2.99	1.31	1.05	1.08
*Alpha band*								
Difficulty level	3 balls	4 balls	5 balls	6 balls	3 balls	4 balls	5 balls	6 balls
Juggler J1	0.05	0.04	0.03	0.03	5.03	4.40	2.87	2.73
Juggler J2	0.05	0.05	0.03	0.04	4.72	4.62	3.27	3.09
Hyperbrain	0.03	0.03	0.02	0.02	3.02	3.00	2.48	1.99

The mean coherence maps of J2 showed a more integrated pattern of connections for both frequency bands. The functional connections involved the frontal, central, parietal and occipital areas. Noteworthy, in the alpha band the coherence values between the parietal and occipital areas increased with task difficulty, whereas the coherence values related to other cortical areas underwent only small variations. It is also interesting to observe that the highest coherence values in the theta band were detected for the Medium difficulty level (4 balls), as for J1.

##### Between-brains analysis

**Figure 7:**
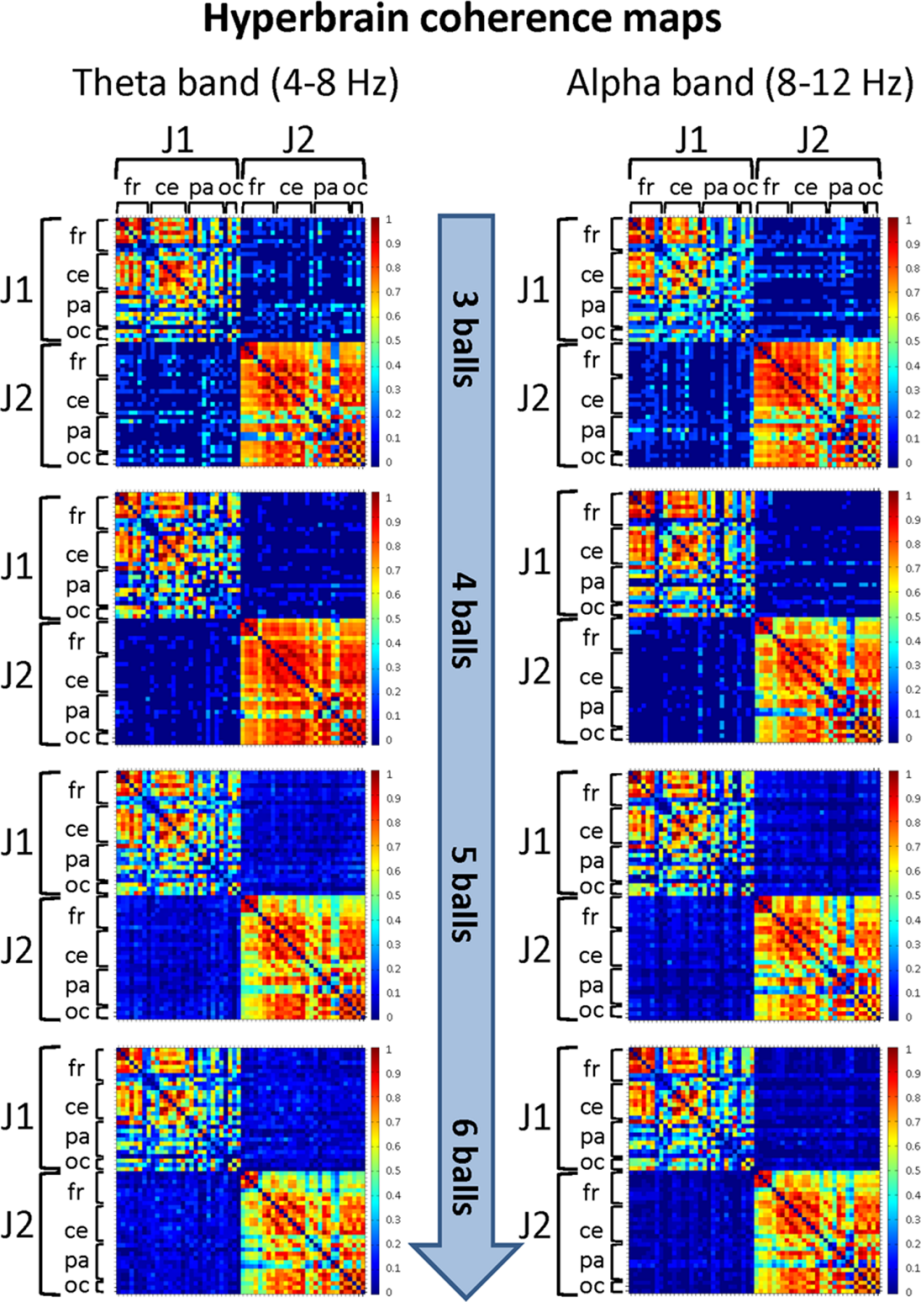
Hyperbrain (between-brains) coherence maps for J1 and J2 in the theta and alpha frequency bands and for the four difficulty levels (3, 4, 5 and 6 juggled balls). The maps are normalized after thresholding to enhance the differences across the retained functional connections. Each coherence map is composed of four quadrants: the upper left and lower right quadrants correspond to the individual coherence maps of J1 and J2, respectively. The upper right and lower left quadrants are the hyperbrain coherence maps between J1 and J2. These maps are specular and display the same type of information, as the upper right map is calculated as J1 vs. J2, whereas the lower left map is calculated as J2 vs. J1. In each quadrant (or individual/hyperbrain coherence map), the electrodes are grouped according to the cortical areas (frontal, central, parietal and occipital), and are listed in the following order from top to bottom and from left to right: “fr” includes the frontal electrodes Fp1, Fpz, Fp2, F7, F3, Fz, F4, F8; “ce” includes the central electrodes FC5, FC1, FC2, FC6, T7, C3, Cz, C4, T8; “pa” includes the parietal electrodes CP5, CP1, CP2, CP6, P7, P3, Pz, P4, P8; “oc” includes the occipital electrodes POz, O1, O2.

The mean coherence maps representing the hyperbrain (between-brains) functional connectivity at cortical level between J1 and J2 for each difficulty level in the alpha and theta bands are summarized in Fig. 7. Estimates of coherence standard error were small (all values ≤ 3% of mean coherence) for all between-brain coherence maps (Table 3). In both bands, these maps showed fairly defined connectivity patterns only for the Easy condition (3 balls). These patterns involved the frontal, central, parietal and occipital areas of both jugglers, but tended to disappear with increasing task difficulty.

In particular, in the alpha band and for the Easy condition (3 balls), we observed that significant functional connections involved: the frontal areas of J1 and the parietal areas of J2; the central areas of J1 and the frontal, central and parietal areas of J2; the parietal and, to a minor extent, the occipital areas of both jugglers. The strongest connections were detected for the central and parietal areas. At the Medium difficulty level (4 balls), only functional connections between the central-parietal areas of J1 and the frontal, central, parietal and occipital areas of J2 remained, but they were less intense than for the Easy difficulty level. For the other difficulty levels, no significant functional connectivity pattern could be identified.

In the theta band and for the Easy condition (3 balls), there was a relatively more complex connectivity pattern, which included strong functional connections involving: the frontal and central areas of J1 and the frontal, central-parietal and occipital areas of J2; the parietal areas of J1 and the fronto-central, central, parietal and occipital areas of J2; the parietal-occipital areas J1 and occipital areas of J2. The strongest connections involved the central, parietal and occipital areas. This pattern disappeared almost completely for the Medium difficulty level (4 balls), and only weak functional connections between the central, parietal and occipital areas of J1 and the parietal areas of J2 could be identified. No significant functional connectivity pattern was detected for the other difficulty levels.

#### Measures of functional organization

##### Within-brain analysis

The measures of functional organizations were performed by calculating the segregation, integration and small-world properties of the functional brain networks, represented by the mean coherence maps. These measures highlighted a different topology and type of efficiency for J1 and J2 at the individual level (see Tables 4 and 5, respectively).

**Table 4:** Normalized mean clustering coefficient C/Cn (related to information segregation), normalized characteristic path length L/Ln (related to information integration), and small-world (SW) measures for J1 in the theta and alpha bands.

Difficulty level	Nr. balls	C/Cn		L/Ln		SW	
		Theta	Alpha	Theta	Alpha	Theta	Alpha
Easy	3	1.73	1.33	1.45	1.56	1.20	0.86
Medium	4	1.65	1.11	1.42	1.41	1.16	0.79
Hard	5	1.21	1.24	1.34	1.95	0.90	0.64
Very hard	6	1.18	1.29	1.98	1.90	0.59	0.68

**Table 5:** Normalized mean clustering coefficient C/Cn (related to information segregation), normalized characteristic path length L/Ln (related to information integration), and small-world (SW) measures for J2 in the theta and alpha bands.

Difficulty level	Nr. balls	C/Cn		L/Ln		SW	
		Theta	Alpha	Theta	Alpha	Theta	Alpha
Easy	3	1.14	1.09	1.26	1.15	0.90	0.95
Medium	4	1.11	1.12	0.76	1.26	1.46	0.89
Hard	5	1.07	1.17	1.23	1.28	0.87	0.91
Very hard	6	1.12	1.09	1.38	1.34	0.81	0.82

In general, the functional network of J1 showed to be more segregated than integrated in both frequency bands, as assessed by the values of the normalized *mean clustering coefficient* and *characteristic path length*. This means that the functional network of J1 had a topology close to that of a regular network, characterized by an efficiency of local type. This topology was enhanced with increasing task difficulty. Values of “small-world” *SW* greater than unit were found only in the theta band and for the Easy and Medium difficulty levels, most probably because of sufficiently low normalized values of the *characteristic path length*. This indicates that, for relatively easy tasks, the functional organization of J1’s brain in the theta band was of a small-world type, i.e. capable of combining local and global efficiency, and of adapting to changing task demands (see Bassett et al., 2006).

On the other hand, the functional network of J2 showed to be more integrated than segregated in both frequency bands, as demonstrated by the values of the normalized *mean clustering coefficient* and *characteristic path length*, which were lower than those observed for J1. Therefore, the functional network of J2 had a topology closer to that of a random network, characterized by an efficiency of global type. No clear dependence of these metrics on task difficulty could be observed. However, in the theta band and for the Medium difficulty level, the normalized *characteristic path length* was sufficiently low to obtain a *SW* metrics greater than unit despite the rather low value of the normalized *mean clustering coefficient*. This indicates that, for this difficulty level, also the functional organization of J2’s brain in the theta band was of a small-world type, hence more efficient and capable to adapt to changing task demands (Bassett et al., 2006).

##### Between-brains analysis

The results of the functional organization measures on the hyperbrain network are summarized in Table 6. From the values of the normalized *mean clustering coefficient* and *characteristic path length* (the latter ones being always very high), one can see that the hyperbrain network of J1 and J2 was, at all difficulty levels, much more segregated than integrated in both frequency bands. As a consequence, the *SW* metrics was never greater than unit, indicating that the hyperbrain network of J1 and J2 had a prevailing local type of efficiency. It is worth noting that the higher *mean clustering coefficient* values (hence the more strongly segregated organization) was detected for the theta band and for the Easy and Medium difficulty levels.

**Table 6:** Normalized mean clustering coefficient C/Cn (related to information segregation), normalized characteristic path length L/Ln (related to information integration), and small-world (SW) measures of the hyperbrain functional network of J1 and J2 in the theta and alpha bands.

Difficulty level	Nr. balls	C/Cn		L/Ln		SW	
		Theta	Alpha	Theta	Alpha	Theta	Alpha
Easy	3	1.32	1.28	1.58	1.56	0.84	0.82
Medium	4	1.63	1.63	2.13	1.96	0.77	0.83
Hard	5	1.07	1.11	1.99	2.11	0.54	0.53
Very hard	6	1.07	1.23	1.91	2.36	0.56	0.52

## Discussion

In this proof of concept study, we used the “juggling paradigm” (Filho et al., 2015) as a platform to assess hyperbrain dynamics between two jugglers as a function of task difficulty and with respect to the functional features at individual level (within-brain condition). We targeted the integrative and segregative functional tendencies of the jugglers’ hyperbrain network as praxis to examine the notions of shared and complementary mental models. In addition to electrophysiological measures of the two interacting brains, we considered psychological measures of affect, self- and others efficacy and attentional focus on the task that have been found to influence performance. Next, we discuss the results pertaining to the psychological states of the jugglers throughout the juggling task and to the neurophysiological patterns obtained for the within-brain and between-brains conditions. Then, we comment on the limitations and strengths of this study. Finally, we provide a summary of the main findings and suggestions for future research avenues.

### Psychological factors

Our analysis revealed that both jugglers perceived higher levels of arousal and pleasantness as the juggling task became increasingly difficult. First, these findings are in line with H1 as the jugglers’ affective and cognitive responses changed as a function of task difficulty (H1). Specifically, our analyses revealed that both jugglers enjoyed challenging tasks more than easier tasks. Sufficiently challenging tasks, as defined in peer-debriefing meetings prior to the actual data collection, are more likely to elicit feelings of enjoyment in comparison to easier tasks. In fact, the proper balance between individuals’ challenge and skills is a cornerstone of the flow-feeling theory (Jackson & Csikszentmihalyi, 1999), which is a pervasive account of the antecedents of optimal performance in motor tasks. Second, the increase in perceived arousal levels was likely related to the need to allocate greater psychophysiological resources to the more challenging tasks, as is often the case for both motor and cognitively demanding tasks (Oxendine, 1970; Medeiros Filho, Moraes & Tenenbaum, 2009; Stern, Ray & Quigley, 2001).

The results for the data on efficacy beliefs and attention are in line with H1 as they echo the notion that cognitive-affective responses are linked to task difficulty. First, our findings support the theoretical notion that confidence, at the individual or group-level of analysis, tends to decrease with increasing task difficulty (Bandura, 1997). This is particularly true when increases in task difficulty and performance measures are objective and highly identifiable (Bandura, 2006), as in the case of juggling with an increasing number of balls. With respect to attentional focus, our findings corroborate previous research suggesting that harder physical tasks tend to elicit an associative attentional response, compared to easier tasks (Basevitch et al., 2011; Razon, Hutchinson & Tenenbaum, 2012; Tenenbaum, 2005). Challenging physical and motor-cognitive tasks require most attentional resources to be directed to the task rather than turning attention to distracting thoughts or external cues unrelated to performance. For the Hard and Very Hard tasks both jugglers reported the same degree of association. This suggests that during harder cooperative tasks partners need to “match” the attentional requirements of the task while mirroring each other’s focus states (Goldman, 2012; Kalick & Hamilton, 1986; Oxendine, 1970).

Perhaps most importantly, both jugglers were able to adapt to the increasing task demands by exhibiting a similar pattern of affective-cognitive responses, as related to core affect (arousal and pleasantness) and efficacy beliefs (self-efficacy and others efficacy) and attentional focus. These findings are in line with H2 and Theory of Mind. In fact, a central assumption of the Theory of Mind, which formed the basis for the development of the notion of shared and complementary mental models, is that the ability to mirror abstract affective-cognitive states is paramount in the establishment of complex social interactions (Goldman, 2012). Particular to motor tasks, the degree of similar affective-cognitive states exhibited by teammates has been linked to the likelihood of experiencing optimal performance in team contexts (Eccles, 2010; Filho, Tenenbaum & Yang, 2015). Altogether, these findings add support to the notion that similar affective-cognitive states are related to performance in cooperative tasks (Eccles, 2010; Filho, Tenenbaum & Yang, 2015).

### Within-brain features

The functional analysis at the individual brain level revealed that J1 exhibited, in general, a more clustered (segregated) pattern of functional connections and a more local type of functional efficiency, thereby suggesting that J1 relied more on specialized cortical areas to perform the task at hand. Interestingly, during the performance of the easier tasks, J1 showed a small-world functional organization in the theta band, indicative of a flow-like state (in line with the *neural efficiency hypothesis*) in which local and global processing are combined to adapt to changing task demands (see Bassett et al., 2006). However, this configuration rapidly moved to a more ordered structure in the Hard condition and even more so in the Very Hard condition. Therefore, the prevalent segregated configuration observed for J1 in both frequency bands became more evident as task difficulty increased. Conversely, in both frequency bands, J2 showed a more integrated functional organization and a more global type of functional efficiency, which did not change appreciably as task difficulty increased. A small-world functional organization was found only for the Medium difficulty level task in the theta band. This could reflect a temporarily more efficient performance based on the practice of the Easy task, which cannot be associated with steady memory performance but rather could be related to an increased attention focus occurring during the execution of a more difficult task (see Fig. 4E). The absence of any SW structure in the alpha band for both jugglers is in line with recent results observing that SW values are always lower in the alpha band than in the theta band (Vecchio et al., 2016), and can be related to poor task-specific mental representations (Klimesch, 1999; Vecchio et al., 2016). However, it is interesting to observe that the SW values of J1 in the alpha band were always smaller than those of J2, suggesting that the functional organization of J1’s brain was more ordered than that of J2’s brain.

These findings are likely related to the skill level of each juggler. J1 had more years of structured practice experience, which have been shown to underpin the acquisition of highly-skilled domain specific memory structures (for a review, see Ericsson, 2007). As such, J1 likely relied on the highly efficient integration of specialized brain areas and movement schemas (i.e., *neural efficiency hypothesis*; see Grabner, Neubauer & Stern, 2006) intrinsically related to the juggling task (*long-term working memory*; see Ericsson & Kintsch, 1995). As task difficulty increased, an even more specialized memory retrieval route was needed, as mirrored in the increasingly ordered (segregated) neural functional pattern exhibited by J1 (see Table 4) and in agreement with his perceived attention rating (see Fig. 4E). In other words, the increase in task difficulty likely led J1 to focus fully on the task at hand, thus “silencing” unnecessary cortical communication in the brain (Bertollo et al., 2013; Bortoli et al., 2012; Comani et al., 2014; Hatfield & Kerick, 2007; Nakata et al., 2010). It is interesting to note that this “full focus mode” has been given several names (e.g., “individual zones of optimal functioning,” “peak performance”), all denoting the multi-layered affective-cognitive-behavioral and neural adaptations needed to successfully accomplish difficult motor tasks (for a review, see Filho & Tenenbaum, 2015).

On the other hand, J2 was a less experienced juggler in comparison to J1, and that is likely why he exhibited a less ordered, less specialized neural network while performing the juggling tasks. The more integrated functional pattern observed for J2 was possibly related to an over-recruitment of cortical resources (i.e., multiple specialized cortical areas) due to cognitive overload or reinvestment of attention needed to cope with the increasing difficulty level of the task. In fact, extensive research suggests that “neural efficiency” or “automaticity” is positively related to skill level (Yarrow, Brown & Krakauer, 2009; Nakata et al., 2010). That is, with an increase in task difficulty, less experienced performers tend to over-analyze every action (serial, step-by-step processing) to accomplish a given motor task (*reinvestment hypothesis*; see Masters & Maxwell, 2008). This so called “reinvestment of attention” might be represented, in neural terms, by the integrated recruitment of multiple brain networks to accomplish the assigned task.

Therefore, while J1’s attentional focus to the task at hand was likely optimal and sustained by a more segregated neural functional pattern and local type of efficiency (according to the *neural efficiency hypothesis*), J2’s internal attentional focus mirrored a cognitive overload (according to the *reinvestment hypothesis*) and was characterized by a functional pattern more integrated than that of J1, suggesting a more global type of efficiency. In fact, extensive research suggests that skill level moderates the influence of attentional focus on performance (Ericsson, 2007; Ericsson & Kintsch, 1995; Ericsson & Ward, 2007; Tenenbaum, 2003), in the sense that a similar attentional focus might reflect either optimal or poor adaptation to the task depending on one’s skill level.

In all, our results for the within-brain analysis indicate that the two jugglers presented different patterns of functional connections and type of efficiency that are likely explained by their skill level. These findings confirm our study hypothesis that a more segregated brain functional organization would underlie the execution of automated tasks by a more expert juggler, whereas a more integrated brain functional organization would support the execution of the dyadic tasks by a less skilled juggler.

### Between-brains features

The hyperbrain analysis in the alpha band highlighted various connections between the brains of J1 and J2, in particular in the frontal, central, parietal, and occipital areas. These connections were likely related to two intertwined needs of the jugglers: (a) the need to *externally adapt* to the dyadic task and its increasing difficulty level, and (b) the need to *internally adapt* to one’s own “system” while simultaneously co-adapting to one’s “partners’ system.” In respect to the former, it is well-established that juggling involves attentional control (frontal lobe and central midline area) and gaze at specific locations (occipital lobe), along with an overall need for integrating all inputs into the parietal lobe (Dessing, Rey & Beek, 2012; Draganski et al., 2004). Although we did not control for hyperconnections resulting from coincidental synchrony between subjects (Burgess, 2013), the fact that we observed hyperbrain connectivity patterns only for the Easy condition suggests that our experimental manipulation was reliable. That is, the reciprocal influence of the two systems (i.e., the jugglers) adjusting to each other depended on task difficulty. In fact, the observed connections vanished for the more difficult tasks, thereby suggesting that the increasing complexity of the assigned task contributed to the disruption of the observed hyperbrain pattern. Indeed, previous research in dyadic guitar playing and choral singing has noted that at least part of the coupled peripheral (e.g., heart rate; see Vickhoff et al., 2013) or central (i.e., hyperbrains; see Lindenberger et al., 2009; Müller, Sänger & Lindenberger, 2013) dynamics among interactive patterns is due to the task at hand.

The hyperbrain analysis in the theta band also underlined possible connections between the brains of J1 and J2, involving areas of the attentional network, i.e. the frontal, central, parietal, and occipital lobes. Having acknowledged the role of the task in activating these brain areas and “binding” the two brains together, it is worth noting that the strongest hyperbrain connections in the theta band involved J1’s frontal and central areas and J2’s frontal, central and occipital areas. While the theta activity in the fronto-central areas of both jugglers is likely related to attentional demands of the task, the theta activity observed in the parietal areas was possibly due to information integration processes. Conversely, the singular activation of J2’s occipital area might be due to attentional control strategies. Grounded on evidence that less experienced performers rely heavily on visual information mainly processed in the occipital lobe (Hatfield & Kerick, 2007; Yarrow, Brown & Krakauer, 2009), it is possible that J2 engaged in “target control strategy” which involves eye-following an object rather than gazing at a central location (“context control,” see Dessing, Rey & Beek, 2012; Tenenbaum, 2003). To fully verify this thesis, future studies of cooperative juggling should combine EEG and eye-tracking measures.

Of potential greater theoretical and applied relevance are the results of graph analysis. The main observations include: a) hyperbrain functional patterns only for the Easy and Medium difficulty level tasks, and b) a segregated topology supporting a prevailing local type of functional efficiency (i.e., the recruitment of specialized cortical areas to perform the task). No small-world organization could be observed, although it is worth noting that the SW index decreased in both bands as task difficulty increased (Table 5). Altogether, these findings suggest that cooperative dyadic juggling is supported by integrated activity from both brains with a segregated (i.e., specialized) hyperbrain functional organization during the execution of relatively easy tasks. Conversely, the performance of harder tasks was not supported by a significant hyperbrain functional organization. In the latter case, cooperative juggling seems to rely on individual skills, as mirrored in the uncorrelated individual functional brain patterns. From a psychological perspective, these results seem to support the notion that easier dyadic tasks rely on shared mental models (i.e., on the functional integration of specialized cortical areas from the two brains), whereas harder tasks require the recruitment of complementary mental models mirrored in uncorrelated individual cortical activation patterns (i.e., idiosyncratic knowledge held by each team member).

Overall, these findings seem to support H3 in which a between-brains functional coupling between the two jugglers would exist during the execution of a cooperative motor task, although only for relatively easy tasks. The overall segregated functional organization of the corresponding hyperbrain network is also in line with H4 in the sense that automated actions rely more on the recruitment of specialized cortical areas. Therefore, the *neural efficiency hypothesis* (Grabner, Neubauer & Stern, 2006) seems to hold true not only at an individual level but also at a team level. This interpretation of our hyperbrain results is also in agreement with the theoretical notion that cooperative tasks of increasing difficulty (realized in this study through the increase of the degrees of freedom, i.e., the number of juggled balls) rely on idiosyncratic knowledge held by teammates (i.e., complementary mental models). Relatively easier tasks can be accomplished with shared knowledge (Eccles, 2010; Eccles & Tenenbaum, 2004; Filho & Tenenbaum, 2012). To this extent, in another recent case study on the intra-team psychophysiological rhythms of a cooperative juggling dyad, we found that two juggling partners exhibited higher degrees of shared psychophysiological responses (i.e., heart rate, breathing rate, perceived arousal) in an easy task, when compared to a hard task. In the hard task, the more skilled juggler exhibited significantly lower psychophysiological responses than his less skilled juggling partner (Filho et al., 2016).

These results are consistent with our findings on the functional organization of the individual cortical networks of the two jugglers, and partially confirm H4 in that a more integrated brain functional organization would be associated with lower skill levels. Indeed we found that the more skilled juggler, J1, exhibited a more segregated functional organization (which is likely to be at the basis of his automatized skill execution), whereas the less skilled juggler, J2, showed a more integrated functional organization, most likely related to an attempt to compensate for his lack of automaticity. At a hyperbrain level of analysis, the more segregated functional pattern found for the Easy and Medium difficulty levels was likely due to the low skill level of the two jugglers as a dyad. Indeed, we found no meaningful hyperbrain pattern for the harder tasks. To perform the dyadic task, the jugglers most likely exhibited idiosyncratic responses that ultimately complemented each other so that collectively they could “match” the psychophysiological demands needed to accomplish the task (see “the matching hypothesis;” Hanin, 2007; Kalick & Hamilton, 1986; Oxendine, 1970). Thus, the relationship between skill level (which is linked to cognitive-affective mental representations and the perception of task difficulty) and the degree of shared and complementary responses might hold true not only for peripheral but also central nervous system responses at both the individual and dyadic levels of analysis.

### Limitations and avenues for future research

Our study is limited by a number of factors that we report in an attempt to critically integrate our findings in the current literature. We also offer ideas on how to advance research in the area of multi-brain interactions.

First, our results should not to be generalized, as power is limited in case studies. In particular, the total number of trials per condition did not yield great statistical power. Future research might consider inter-subject validation. Notwithstanding, scholars should remain aware of the “individual response stereotype” (individual level effect; i.e., different individuals show different bio-psycho-social responses to different task stimuli) and “stimulus-response specificity” (i.e., group-level effect; “average response” in the population for a given task stimuli) that are at play in team-level interactions (for a review, see Stern, Ray & Quigley, 2001). Thus, an appropriate nomothetic study in behavioral sciences and social neuroscience should involve a power analysis considering the nested structure of dyadic interactions and its inherent compound variance at the individual and group-level of analysis (Cacioppo & Berntson, 1992; Raudenbush & Bryk, 2002). However, this would likely require a large and potentially unrealistic number of skilled juggling dyads. For this reason, we maintain that a series of single case and small-n studies is a more realistic and appropriate design for researchers interested in multi-brain interactions in motor tasks in general and juggling in particular. Multi-site studies are also warranted to increase statistical power and the internal validity of hyperbrain studies using interactive neuro-scientific paradigms, such as the juggling paradigm. To this extent, multi-site studies have become the gold standard in neuro-scientific research, as they allow for larger statistical power, replication and standardization of best research and practice guidelines (see Keshavan et al., 2016).

Second, the fact that the jugglers did not have the same level of experience and skill may be considered as a weakness of the study. Although jugglers with similar skill levels would likely increase the reproducibility of our case study, it was not easy to find jugglers able to juggle with more than five balls. In the real world, skill level is a continuum and teams are usually a mix of individuals of varying abilities (Bandura, 1997; Eccles, 2010; Filho & Tenenbaum, 2012). Furthermore, important insights into group processes in social psychology (e.g., Kohler effect, social facilitation, cohesiveness levels) are derived from studies with individuals of varying skill levels. Future research should then continue to examine how the integrative and segregative tendencies of hyperbrain networks vary in respect to cooperative partners with more or less similar skill levels. Specifically, a hyperbrain study on “group-flow” or “momentum” is particularly warranted as the balance between “skill and challenge” (see Jackson & Csikszentmihalyi, 1999) is much harder to determine in team contexts, given that this is a by-product of individual characteristics.

Third, in this proof of concept study we did not evaluate the direction of information flow between the two jugglers. The availability of information on the direction of the functional exchange between the two jugglers would have allowed us to identify the leader and the follower in the cooperative interaction, thereby enhancing our understanding of the hyperbrain dynamics. To go beyond the simple evaluation of functional correlations between the partners, and to determine the role played by each individual to accomplish the task at hand, future studies should include synchronization measures across the EEG signals from the two brains, such as the Phase Synchronization Index or the Integrative Coupling Index, as in other hyperbrain studies (Müller & Lindenberger, 2011; Müller, Sänger & Lindenberger, 2013; Sänger, Müller & Lindenberger, 2013). Also, adopting a counterbalanced design across task difficulties, rather than the incremental design reported herein, may clarify the role of fatigue on hyperbrain dynamics in dyadic juggling in particular, and interactive motor tasks at large.

Despite these limitations, we consider that our proof of concept study contributes to advance research in social neuroscience, as for the first time we studied the functional interactions of human brains involved in a highly interactive motor task. To our best knowledge, since the discovery of the mirror neurons in the 1980s and the first appearance of the word “social neuroscience” in a paper by Cacioppo & Berntson (1992), there has been no research on multi-brains during motor tasks characterized by a perturbation/adjustment mode during the reciprocal interaction. Previous studies have relied on observational paradigms, diachronic tasks (e.g., I play cards, so that you can respond) or simpler tasks carried out in constrained environments (e.g., finger tapping in fMRI). While there is much work to be done in the field, our findings have both theoretical and applied implications to behavioral and social neuroscientists, as well as educators across domains of human interest.

From a theoretical standpoint, the observation that hyperbrains exhibit the same functional characteristics as single brains adds to the discussion that single brains are fractals of dual-hyperbrains, which in turn are fractals of triade-hyperbrains, and so forth. In the end, hyperbrains might be part of a larger pattern that repeats itself in nature (“The Universe as a Giant Brain;” see Krioukov et al., 2012). Further exploring this notion might expand our understanding of the Theory of Mind and Social Brain Hypothesis, in which the human ability to establish cooperative social groups is bounded not only by our brain as an individual unit but also by the replication of integrative/segregative capabilities of individual brains in multiple hyperbrain networks (group-level of analysis).

From an applied standpoint, the notion of shared regulation training, mainly limited to virtual environments, can be advanced through the development of bio-neurofeedback agendas. Such agendas should aim to develop co-regulation exercises that elicit functional adaptations in the peripheral (e.g., heart) and central (i.e., brain) systems. Future applied studies should consider the differences between performing single tasks (solo juggling) and team tasks (dyadic juggling). While it is challenging to compare difficulty levels for individual and cooperative tasks, this experimental design setup would make it possible to establish different guidelines for self- and co-regulation training.

## Conclusions

We observed that the jugglers’ within-brain functional network in the alpha and theta bands was likely related to their skill level, whereas pattern variations with respect to task difficulty were tenuous. Specifically, the more experienced juggler exhibited more segregated functional patterns across brain areas (i.e., a more local type of functional efficiency), consistent with the notion that increased skill is associated with “neural-efficiency” (recruitment of specific areas of the brain related to the task at hand) and “automatic processing.” The less skilled juggler exhibited more integrated functional patterns of neural activity (i.e., a more global type of functional efficiency), probably due to cognitive overload and lack of specialized mnemonic structures, as is often the case for less skilled individuals.

The hyperbrain dynamics seemed to be related to external adaptations to the task (i.e., activation of the brain circuits needed to juggle) and internal adaptations related to the skill level of each juggler. Perhaps most importantly, easier tasks were associated with hyperbrain patterns where specialized cortical areas from the two brains were integrated to produce a functional network with a more local type of efficiency (shared mental models), whereas harder tasks fostered the emergence of segregative tendencies (complementary mental models) that manifested as individual cortical activations uncorrelated at the hyperbrain level. Altogether, these findings may suggest that the personal characteristics of the jugglers (e.g., skill level) and the level of task difficulty influence the hyperbrain features of two interacting brains and the hyperbrain shared/integrative and complementary/segregative tendencies.

## Supplemental Information

10.7717/peerj.2457/supp-1 Supplemental Information 1 Matrix of standard errors of the hyperbrain coherence map calculated for the alpha band and at the Easy difficulty level.

10.7717/peerj.2457/supp-2 Supplemental Information 2 Matrix of standard errors of the hyperbrain coherence map calculated for the alpha band and at the Medium difficulty level.

10.7717/peerj.2457/supp-3 Supplemental Information 3 Matrix of standard errors of the hyperbrain coherence map calculated for the alpha band and at the Hard difficulty level.

10.7717/peerj.2457/supp-4 Supplemental Information 4 Matrix of standard errors of the hyperbrain coherence map calculated for the alpha band and at the Very Hard difficulty level.

10.7717/peerj.2457/supp-5 Supplemental Information 5 Matrix of standard errors of the hyperbrain coherence map calculated for the theta band and at the Easy difficulty level.

10.7717/peerj.2457/supp-6 Supplemental Information 6 Matrix of standard errors of the hyperbrain coherence map calculated for the theta band and at the Medium difficulty level.

10.7717/peerj.2457/supp-7 Supplemental Information 7 Matrix of standard errors of the hyperbrain coherence map calculated for the theta band and at the Hard difficulty level.

10.7717/peerj.2457/supp-8 Supplemental Information 8 Matrix of standard errors of the hyperbrain coherence map calculated for the theta band and at the Very Hard difficulty level.

## List of Non-Standard Abbreviations

C: mean clustering coefficient
L: characteristic path length
C/Cn: normalized mean clustering coefficient
L/Ln: normalized characteristic path length
SW: small-world index.

## Additional Information and Declarations

### Data Deposition

The following information was supplied regarding data availability: Filho E, Bertollo M, Tamburro G, Schinaia L, Jonas Chatel-Goldman, Di Fronso S, Robazza C, Comani S, 2016. Hyperbrain features of team mental models within a juggling paradigm: a proof of concept. Zenodo: 10.5281/zenodo.61256.
