# Supplementary material for: Hyperbrain features of team mental models within a juggling paradigm: a proof of concept
Source: PeerJ. 2016 Sep 20;4:e2457. doi: 10.7717/peerj.2457 (PMC5036110; doi:10.7717/peerj.2457)
Supplement: Supplemental Information 1 [file peerj-04-2457-s001.pdf]

subject 1

		Fp1	Fpz	Fp2	F7	F3	Fz	F4	F8	FC5	FC1	FC2	FC6	T7	C3	Cz	C4	T8	CP5	CP1	CP2	CP6	P7	P3	Pz	P4	P8	POz	O1	O2
subject 1	Fp1	0	0,019533	0,004037	0,046582	0,006232	0,021536	0,049228	0,039094	0,044765	0,020107	0,066734	0,047688	0,055528	0,022365	0,066625	0,052181	0,016135	0,049851	0,034633	0,035187	0,072635	0,057863	0,073245	0,045816	0,014784	0,103928	0,056308	0,0634	0,094636
	Fpz	0,019533	0	0,012306	0,07367	0,021867	0,036202	0,050708	0,050583	0,042609	0,011913	0,075836	0,083487	0,086135	0,038444	0,071844	0,070576	0,015941	0,054855	0,020249	0,049176	0,059453	0,053907	0,097027	0,029111	0,037943	0,107435	0,066814	0,055993	0,09252
	Fp2	0,004037	0,012306	0	0,053311	0,011415	0,026381	0,049791	0,048833	0,036688	0,01679	0,069904	0,061355	0,057368	0,022707	0,0752	0,060899	0,024571	0,045093	0,025523	0,051699	0,067829	0,053934	0,082876	0,052028	0,005489	0,106101	0,078446	0,059735	0,105355
	F7	0,046582	0,07367	0,053311	0	0,066159	0,045814	0,073235	0,016185	0,047314	0,01117	0,020626	0,056068	0,114335	0,049766	0,050945	0,008412	0,062002	0,067901	0,014441	0,076992	0,036229	0,034942	0,088656	0,036522	0,030131	0,062137	0,062696	0,058712	0,039394
	F3	0,006232	0,021867	0,011415	0,066159	0	0,037263	0,041806	0,030476	0,059231	0,020004	0,058109	0,062655	0,057192	0,035643	0,065572	0,053199	0,026961	0,04493	0,00718	0,060227	0,058784	0,037912	0,093868	0,041647	0,022496	0,083972	0,067043	0,043358	0,087429
	Fz	0,021536	0,036202	0,026381	0,045814	0,037263	0	0,061591	0,022436	0,059708	0,032541	0,049559	0,03468	0,060432	0,040928	0,049278	0,058804	0,016444	0,04515	0,030659	0,051795	0,043662	0,034139	0,094385	0,022314	0,014625	0,082125	0,059121	0,050551	0,093575
	F4	0,049228	0,050708	0,049791	0,073235	0,041806	0,061591	0	0,019718	0,051427	0,02396	0,093423	0,100766	0,076815	0,063038	0,089528	0,074051	0,06327	0,066173	0,017779	0,059447	0,029196	0,083995	0,070445	0,042141	0,034837	0,034413	0,085194	0,074001	0,054264
	F8	0,039094	0,050583	0,048833	0,016185	0,030476	0,022436	0,019718	0	0,035341	0,06489	0,040481	0,050181	0,021819	0,041813	0,018643	0,035351	0,048406	0,046805	0,067742	0,0348	0,031643	0,019379	0,042302	0,020976	0,06361	0,068243	0,055887	0,021381	0,035
	FC5	0,044765	0,042609	0,036688	0,047314	0,059231	0,059708	0,051427	0,035341	0	0,024774	0,06352	0,103862	0,139748	0,013725	0,083923	0,022067	0,0219	0,100678	0,019663	0,043929	0,020499	0,053653	0,070225	0,021871	0,015842	0,046831	0,073229	0,058756	0,027393
	FC1	0,020107	0,011913	0,01679	0,01117	0,020004	0,032541	0,02396	0,06489	0,024774	0	0,051556	0,028387	0,032781	0,007229	0,024927	0,048197	0,025157	0,022669	0,023562	0,010636	0,075518	0,037751	0,03738	0,069583	0,042674	0,094666	0,056257	0,057321	0,060873
	FC2	0,066734	0,075836	0,069904	0,020626	0,058109	0,049559	0,093423	0,040481	0,06352	0,051556	0	0,035109	0,086342	0,068829	0,030631	0,016635	0,050973	0,067472	0,01353	0,08102	0,067704	0,045492	0,091564	0,020999	0,030958	0,052541	0,054443	0,037047	0,071001
	FC6	0,047688	0,083487	0,061355	0,056068	0,062655	0,03468	0,100766	0,050181	0,103862	0,028387	0,035109	0	0,087812	0,095679	0,028158	0,056484	0,007922	0,074923	0,02225	0,062678	0,049017	0,030994	0,07374	0,022845	0,010013	0,071581	0,050128	0,041778	0,048601
	T7	0,055528	0,086135	0,057368	0,114335	0,057192	0,060432	0,076815	0,021819	0,139748	0,032781	0,086342	0,087812	0	0,125216	0,084493	0,092857	0,034609	0,053264	0,017685	0,040634	0,017908	0,02113	0,064309	0,063776	0,01182	0,057828	0,06557	0,025538	0,071751
	C3	0,022365	0,038444	0,022707	0,049766	0,035643	0,040928	0,063038	0,041813	0,013725	0,007229	0,068829	0,095679	0,125216	0	0,090432	0,0317	0,035031	0,086366	0,025041	0,054631	0,023058	0,051497	0,080709	0,011598	0,041673	0,058303	0,073335	0,054925	0,040746
	Cz	0,066625	0,071844	0,0752	0,050945	0,065572	0,049278	0,089528	0,018643	0,083923	0,024927	0,030631	0,028158	0,084493	0,090432	0	0,042927	0,03157	0,068661	0,02176	0,078555	0,056022	0,042165	0,08693	0,018562	0,021019	0,041506	0,059698	0,039452	0,05317
	C4	0,052181	0,070576	0,060899	0,008412	0,053199	0,058804	0,074051	0,035351	0,022067	0,048197	0,016635	0,056484	0,092857	0,0317	0,042927	0	0,055697	0,070529	0,02479	0,101671	0,086799	0,052521	0,110918	0,038084	0,01681	0,044351	0,070156	0,056464	0,086553
	T8	0,016135	0,015941	0,024571	0,062002	0,026961	0,016444	0,06327	0,048406	0,0219	0,025157	0,050973	0,007922	0,034609	0,035031	0,03157	0,055697	0	0,015098	0,088425	0,079816	0,069041	0,055667	0,045167	0,081126	0,070858	0,050196	0,032073	0,097333	0,007052
	CP5	0,049851	0,054855	0,045093	0,067901	0,04493	0,04515	0,066173	0,046805	0,100678	0,022669	0,067472	0,074923	0,053264	0,086366	0,068661	0,070529	0,015098	0	0,006491	0,039013	0,067167	0,038347	0,080293	0,03362	0,030565	0,096965	0,069066	0,039093	0,085089
	CP1	0,034633	0,020249	0,025523	0,014441	0,00718	0,030659	0,017779	0,067742	0,019663	0,023562	0,01353	0,02225	0,017685	0,025041	0,02176	0,02479	0,088425	0,006491	0	0,015211	0,088556	0,049378	0,049791	0,072555	0,039618	0,077391	0,047868	0,051684	0,040598
	CP2	0,035187	0,049176	0,051699	0,076992	0,060227	0,051795	0,059447	0,0348	0,043929	0,010636	0,08102	0,062678	0,040634	0,054631	0,078555	0,101671	0,079816	0,039013	0,015211	0	0,028445	0,03371	0,078025	0,078847	0,03946	0,022504	0,051481	0,026897	0,068794
	CP6	0,072635	0,059453	0,067829	0,036229	0,058784	0,043662	0,029196	0,031643	0,020499	0,075518	0,067704	0,049017	0,017908	0,023058	0,056022	0,086799	0,069041	0,067167	0,088556	0,028445	0	0,044571	0,046006	0,030739	0,089559	0,016691	0,067413	0,026172	0,05341
	P7	0,057863	0,053907	0,053934	0,034942	0,037912	0,034139	0,083995	0,019379	0,053653	0,037751	0,045492	0,030994	0,02113	0,051497	0,042165	0,052521	0,055667	0,038347	0,049378	0,03371	0,044571	0	0,053619	0,027051	0,043676	0,022474	0,037309	0,036675	0,038089
	P3	0,073245	0,097027	0,082876	0,088656	0,093868	0,094385	0,070445	0,042302	0,070225	0,03738	0,091564	0,07374	0,064309	0,080709	0,08693	0,110918	0,045167	0,080293	0,049791	0,078025	0,046006	0,053619	0	0,01037	0,026723	0,05165	0,089231	0,047237	0,093262
	Pz	0,045816	0,029111	0,052028	0,036522	0,041647	0,022314	0,042141	0,020976	0,021871	0,069583	0,020999	0,022845	0,063776	0,011598	0,018562	0,038084	0,081126	0,03362	0,072555	0,078847	0,030739	0,027051	0,01037	0	0,082958	0,048973	0,062513	0,062001	0,049342
	P4	0,014784	0,037943	0,005489	0,030131	0,022496	0,014625	0,034837	0,06361	0,015842	0,042674	0,030958	0,010013	0,01182	0,041673	0,021019	0,01681	0,070858	0,030565	0,039618	0,03946	0,089559	0,043676	0,026723	0,082958	0	0,047863	0,038233	0,070702	0,029064
	P8	0,103928	0,107435	0,106101	0,062137	0,083972	0,082125	0,034413	0,068243	0,046831	0,094666	0,052541	0,071581	0,057828	0,058303	0,041506	0,044351	0,050196	0,096965	0,077391	0,022504	0,016691	0,022474	0,05165	0,048973	0,047863	0	0,050061	0,019202	0,053676
	POz	0,056308	0,066814	0,078446	0,062696	0,067043	0,059121	0,085194	0,055887	0,073229	0,056257	0,054443	0,050128	0,06557	0,073335	0,059698	0,070156	0,032073	0,069066	0,047868	0,051481	0,067413	0,037309	0,089231	0,062513	0,038233	0,050061	0	0,028511	0,06619
	O1	0,0634	0,055993	0,059735	0,058712	0,043358	0,050551	0,074001	0,021381	0,058756	0,057321	0,037047	0,041778	0,025538	0,054925	0,039452	0,056464	0,097333	0,039093	0,051684	0,026897	0,026172	0,036675	0,047237	0,062001	0,070702	0,019202	0,028511	0	0,033873
	O2	0,094636	0,09252	0,105355	0,039394	0,087429	0,093575	0,054264	0,035	0,027393	0,060873	0,071001	0,048601	0,071751	0,040746	0,05317	0,086553	0,007052	0,085089	0,040598	0,068794	0,05341	0,038089	0,093262	0,049342	0,029064	0,053676	0,06619	0,033873	0
subject 2	Fp1	0,041431	0,042181	0,033255	0,030209	0,049646	0,029915	0,044535	0,067376	0,030833	0,029093	0,017282	0,036509	0,038335	0,052357	0,048594	0,032853	0,022959	0,024575	0,040571	0,02306	0,012513	0,021118	0,050857	0,004906	0,041341	0,051776	0,040175	0,034135	0,038422
	Fpz	0,046672	0,049934	0,04436	0,025961	0,050264	0,032628	0,049332	0,044382	0,023859	0,037795	0,013514	0,036181	0,040674	0,053095	0,033025	0,013714	0,007519	0,034368	0,031784	0,039363	0,026267	0,020661	0,050513	0,019403	0,026604	0,054793	0,042538	0,032606	0,045744
	Fp2	0,023655	0,030883	0,022082	0,027924	0,040237	0,013488	0,048775	0,040751	0,020916	0,016127	0,007814	0,033937	0,035463	0,052133	0,03118	0,005302	0,012879	0,01229	0,020023	0,050301	0,019381	0,026177	0,039378	0,022985	0,024437	0,046704	0,06244	0,03757	0,048934
	F7	0,028762	0,028154	0,023195	0,025745	0,031358	0,022218	0,055096	0,032295	0,024455	0,013729	0,007595	0,041737	0,040962	0,049029	0,030808	0,013253	0,006223	0,012135	0,024613	0,054233	0,019908	0,027977	0,036057	0,021596	0,02276	0,068865	0,059729	0,02427	0,037507
	F3	0,005757	0,007904	0,004757	0,025407	0,024932	0,015807	0,043183	0,015353	0,031469	0,003414	0,015413	0,015386	0,035081	0,044217	0,016787	0,002928	0,016771	0,021864	0,014168	0,032513	0,019396	0,027314	0,030183	0,01818	0,014365	0,066158	0,048082	0,021777	0,036202
	Fz	0,027015	0,018083	0,02819	0,026541	0,039868	0,023279	0,045632	0,04098	0,014922	0,014711	0,007116	0,024748	0,010593	0,037036	0,015713	0,010506	0,013775	0,019164	0,036121	0,030396	0,009203	0,032104	0,048404	0,030245	0,037402	0,073704	0,047937	0,029629	0,043707
	F4	0,021587	0,007914	0,021301	0,005068	0,027735	0,019804	0,040009	0,020782	0,012464	0,012945	0,014928	0,012675	0,025184	0,029632	0,008993	0,026437	0,004773	0,024991	0,019432	0,033543	0,025784	0,032127	0,045418	0,034394	0,032893	0,05366	0,043688	0,02515	0,041323
	F8	0,035245	0,037045	0,031705	0,034208	0,037573	0,029999	0,063094	0,015486	0,019065	0,020667	0,030232	0,047622	0,063357	0,045145	0,042936	0,038512	0,015444	0,01786	0,021591	0,033836	0,032762	0,024646	0,057877	0,010131	0,013686	0,046068	0,025413	0,028503	0,035134
	FC5	0,034591	0,029741	0,027095	0,030625	0,04034	0,031146	0,044585	0,021101	0,039002	0,018776	0,007524	0,037698	0,026027	0,040308	0,020472	0,021865	0,012932	0,019855	0,016237	0,037911	0,033292	0,03895	0,056562	0,006719	0,022462	0,057623	0,03661	0,023728	0,040915
	FC1	0,005626	0,005126	0,004402	0,021012	0,024405	0,009212	0,039004	0,024609	0,02385	0,012145	0,007505	0,027716	0,036784	0,024567	0,006842	0,023341	0,015733	0,016292	0,010473	0,031638	0,030684	0,041963	0,036726	0,024088	0,015315	0,061926	0,034004	0,021113	0,033113
	FC2	0,025022	0,005341	0,02553	0,00533	0,033777	0,018099	0,03647	0,021844	0,020286	0,009901	0,016739	0,014425	0,014511	0,031647	0,008798	0,029344	0,008523	0,018811	0,027942	0,035449	0,040107	0,037219	0,044223	0,03134	0,037151	0,06151	0,046334	0,035173	0,038772
	FC6	0,029442	0,028245	0,026984	0,045054	0,0417	0,030415	0,043545	0,030078	0,046614	0,014971	0,016222	0,03592	0,053368	0,030169	0,020753	0,017996	0,050141	0,009481	0,001513	0,023308	0,013975	0,026458	0,055231	0,008229	0,035975	0,033389	0,028936	0,029023	0,031185
	T7	0,035733	0,036009	0,031866	0,033787	0,045713	0,036699	0,044612	0,026581	0,042695	0,021025	0,016649	0,038445	0,017721	0,04684	0,017365	0,01883	0,013857	0,026399	0,019749	0,047034	0,030093	0,038892	0,057263	0,007397	0,025224	0,050003	0,048836	0,037036	0,054242
	C3	0,020331	0,015501	0,023056	0,038223	0,03869	0,016937	0,047116	0,019517	0,048242	0,025824	0,01856	0,031564	0,016228	0,025694	0,012024	0,032074	0,030636	0,012227	0,015376	0,034917	0,029465	0,036483	0,037018	0,010659	0,005342	0,048785	0,031971	0,01561	0,042842
	Cz	0,031404	0,032666	0,032218	0,035043	0,038198	0,024736	0,045576	0,024528	0,047371	0,021318	0,010963	0,034172	0,025507	0,03824	0,015795	0,019861	0,030145	0,005395	0,016392	0,042032	0,047875	0,048761	0,043963	0,018067	0,007934	0,037829	0,034944	0,007139	0,0347
	C4	0,005453	0,017421	0,006878	0,032402	0,005994	0,015474	0,035311	0,029535	0,019868	0,01511	0,038432	0,070219	0,033413	0,024273	0,052556	0,037572	0,005896	0,015023	0,045694	0,026968	0,04514	0,01873	0,030777	0,029609	0,040664	0,013371	0,043438	0,018692	0,025027
	T8	0,043867	0,050535	0,04847	0,035283	0,055083	0,037838	0,072792	0,011108	0,020679	0,055769	0,040175	0,040599	0,067288	0,037608	0,035719	0,046625	0,030379	0,035195	0,052238	0,037389	0,031074	0,027272	0,072544	0,030419	0,012379	0,047655	0,043185	0,027902	0,054962
	CP5	0,022224	0,023095	0,027437	0,050134	0,04986	0,028223	0,030118	0,0335	0,043199	0,021734	0,0394	0,032262	0,005776	0,027362	0,019267	0,048235	0,020176	0,022579	0,0225	0,037461	0,019045	0,044116	0,046924	0,024324	0,02041	0,026706	0,040709	0,032015	0,050273
	CP1	0,042369	0,040529	0,04392	0,039383	0,031318	0,029467	0,020201	0,026089	0,058153	0,039264	0,034354	0,020126	0,038974	0,054616	0,034873	0,057262	0,042972	0,029833	0,039141	0,052762	0,03359	0,054212	0,009862	0,017659	0,052731	0,027575	0,01493	0,009599	0,002448
	CP2	0,026081	0,023575	0,029365	0,027618	0,036784	0,021016	0,036698	0,006264	0,034133	0,015595	0,003795	0,032818	0,018506	0,024783	0,012618	0,01853	0,01663	0,007834	0,011625	0,029986	0,0493	0,048822	0,041225	0,023804	0,008223	0,045437	0,030723	0,015406	0,038693
	CP6	0,027206	0,018611	0,024427	0,012487	0,013381	0,029884	0,020748	0,010127	0,007071	0,006984	0,025489	0,02474	0,030917	0,002248	0,020294	0,036585	0,006339	0,023499	0,021368	0,032848	0,047343	0,041248	0,031521	0,032933	0,010176	0,024209	0,037858	0,030927	0,018079
	P7	0,019089	0,022152	0,02792	0,051038	0,050116	0,022947	0,018088	0,047909	0,025226	0,020765	0,050378	0,028241	0,014464	0,011479	0,033793	0,060928	0,020494	0,030261	0,028923	0,038696	0,019948	0,046347	0,0459	0,035175	0,026608	0,008819	0,039486	0,042782	0,051891
	P3	0,030473	0,035291	0,037168	0,061304	0,062275	0,033507	0,021023	0,044138	0,031377	0,031821	0,053255	0,041848	0,025811	0,024733	0,043213	0,063617	0,028465	0,033851	0,03667	0,040641	0,026966	0,041691	0,035356	0,036748	0,021363	0,019264	0,043605	0,04247	0,051309
	Pz	0,053969	0,051955	0,0464	0,0503	0,053403	0,041725	0,036027	0,025674	0,062354	0,048024	0,023532	0,035268	0,011708	0,056612	0,022528	0,031427	0,049255	0,025134	0,030704	0,051069	0,056632	0,044286	0,02719	0,022973	0,023077	0,035828	0,038207	0,014361	0,053077
	P4	0,031106	0,030888	0,030212	0,025926	0,032419	0,016506	0,02957	0,028352	0,049367	0,021065	0,009727	0,022685	0,013552	0,035398	0,005303	0,031101	0,030731	0,00529	0,027353	0,040277	0,049281	0,061939	0,043581	0,024282	0,022411	0,025107	0,019805	0,018155	0,02304
	P8	0,024174	0,023327	0,021644	0,013897	0,029082	0,018639	0,051624	0,033826	0,035212	0,016925	0,016766	0,023623	0,018784	0,026269	0,009008	0,038667	0,020735	0,01839	0,013069	0,050059	0,035163	0,059626	0,052311	0,022049	0,019386	0,034442	0,020994	0,017703	0,023295
	POz	0,029901	0,02924	0,032514	0,050288	0,051348	0,031804	0,035961	0,032621	0,056176	0,027238	0,029728	0,04093	0,013623	0,039168	0,020477	0,038358	0,034622	0,019984	0,018358	0,049766	0,032545	0,030985	0,043012	0,008463	0,003517	0,035723	0,044416	0,023159	0,053258
	O1	0,022771	0,020006	0,020657	0,009088	0,028003	0,013825	0,045666	0,040089	0,033183	0,014593	0,022943	0,019979	0,016342	0,023814	0,012916	0,042664	0,026889	0,016361	0,023592	0,048263	0,039129	0,057807	0,056077	0,022287	0,02892	0,028041	0,012485	0,01999	0,02381
	O2	0,028308	0,027121	0,024824	0,043401	0,041673	0,028431	0,040569	0,028725	0,046838	0,021894	0,019016	0,040394	0,023798	0,034032	0,019574	0,029174	0,03295	0,014078	0,010543	0,050006	0,034448	0,038379	0,036345	0,007987	0,004141	0,045335	0,043425	0,015822	0,048455

subject 2

		Fp1	Fpz	Fp2	F7	F3	Fz	F4	F8	FC5	FC1	FC2	FC6	T7	C3	Cz	C4	T8	CP5	CP1	CP2	CP6	P7	P3	Pz	P4	P8	POz	O1	O2
subject 1	Fp1	0,041431	0,046672	0,023655	0,028762	0,005757	0,027015	0,021587	0,035245	0,034591	0,005626	0,025022	0,029442	0,035733	0,020331	0,031404	0,005453	0,043867	0,022224	0,042369	0,026081	0,027206	0,019089	0,030473	0,053969	0,031106	0,024174	0,029901	0,022771	0,028308
	Fpz	0,042181	0,049934	0,030883	0,028154	0,007904	0,018083	0,007914	0,037045	0,029741	0,005126	0,005341	0,028245	0,036009	0,015501	0,032666	0,017421	0,050535	0,023095	0,040529	0,023575	0,018611	0,022152	0,035291	0,051955	0,030888	0,023327	0,02924	0,020006	0,027121
	Fp2	0,033255	0,04436	0,022082	0,023195	0,004757	0,02819	0,021301	0,031705	0,027095	0,004402	0,02553	0,026984	0,031866	0,023056	0,032218	0,006878	0,04847	0,027437	0,04392	0,029365	0,024427	0,02792	0,037168	0,0464	0,030212	0,021644	0,032514	0,020657	0,024824
	F7	0,030209	0,025961	0,027924	0,025745	0,025407	0,026541	0,005068	0,034208	0,030625	0,021012	0,00533	0,045054	0,033787	0,038223	0,035043	0,032402	0,035283	0,050134	0,039383	0,027618	0,012487	0,051038	0,061304	0,0503	0,025926	0,013897	0,050288	0,009088	0,043401
	F3	0,049646	0,050264	0,040237	0,031358	0,024932	0,039868	0,027735	0,037573	0,04034	0,024405	0,033777	0,0417	0,045713	0,03869	0,038198	0,005994	0,055083	0,04986	0,031318	0,036784	0,013381	0,050116	0,062275	0,053403	0,032419	0,029082	0,051348	0,028003	0,041673
	Fz	0,029915	0,032628	0,013488	0,022218	0,015807	0,023279	0,019804	0,029999	0,031146	0,009212	0,018099	0,030415	0,036699	0,016937	0,024736	0,015474	0,037838	0,028223	0,029467	0,021016	0,029884	0,022947	0,033507	0,041725	0,016506	0,018639	0,031804	0,013825	0,028431
	F4	0,044535	0,049332	0,048775	0,055096	0,043183	0,045632	0,040009	0,063094	0,044585	0,039004	0,03647	0,043545	0,044612	0,047116	0,045576	0,035311	0,072792	0,030118	0,020201	0,036698	0,020748	0,018088	0,021023	0,036027	0,02957	0,051624	0,035961	0,045666	0,040569
	F8	0,067376	0,044382	0,040751	0,032295	0,015353	0,04098	0,020782	0,015486	0,021101	0,024609	0,021844	0,030078	0,026581	0,019517	0,024528	0,029535	0,011108	0,0335	0,026089	0,006264	0,010127	0,047909	0,044138	0,025674	0,028352	0,033826	0,032621	0,040089	0,028725
	FC5	0,030833	0,023859	0,020916	0,024455	0,031469	0,014922	0,012464	0,019065	0,039002	0,02385	0,020286	0,046614	0,042695	0,048242	0,047371	0,019868	0,020679	0,043199	0,058153	0,034133	0,007071	0,025226	0,031377	0,062354	0,049367	0,035212	0,056176	0,033183	0,046838
	FC1	0,029093	0,037795	0,016127	0,013729	0,003414	0,014711	0,012945	0,020667	0,018776	0,012145	0,009901	0,014971	0,021025	0,025824	0,021318	0,01511	0,055769	0,021734	0,039264	0,015595	0,006984	0,020765	0,031821	0,048024	0,021065	0,016925	0,027238	0,014593	0,021894
	FC2	0,017282	0,013514	0,007814	0,007595	0,015413	0,007116	0,014928	0,030232	0,007524	0,007505	0,016739	0,016222	0,016649	0,01856	0,010963	0,038432	0,040175	0,0394	0,034354	0,003795	0,025489	0,050378	0,053255	0,023532	0,009727	0,016766	0,029728	0,022943	0,019016
	FC6	0,036509	0,036181	0,033937	0,041737	0,015386	0,024748	0,012675	0,047622	0,037698	0,027716	0,014425	0,03592	0,038445	0,031564	0,034172	0,070219	0,040599	0,032262	0,020126	0,032818	0,02474	0,028241	0,041848	0,035268	0,022685	0,023623	0,04093	0,019979	0,040394
	T7	0,038335	0,040674	0,035463	0,040962	0,035081	0,010593	0,025184	0,063357	0,026027	0,036784	0,014511	0,053368	0,017721	0,016228	0,025507	0,033413	0,067288	0,005776	0,038974	0,018506	0,030917	0,014464	0,025811	0,011708	0,013552	0,018784	0,013623	0,016342	0,023798
	C3	0,052357	0,053095	0,052133	0,049029	0,044217	0,037036	0,029632	0,045145	0,040308	0,024567	0,031647	0,030169	0,04684	0,025694	0,03824	0,024273	0,037608	0,027362	0,054616	0,024783	0,002248	0,011479	0,024733	0,056612	0,035398	0,026269	0,039168	0,023814	0,034032
	Cz	0,048594	0,033025	0,03118	0,030808	0,016787	0,015713	0,008993	0,042936	0,020472	0,006842	0,008798	0,020753	0,017365	0,012024	0,015795	0,052556	0,035719	0,019267	0,034873	0,012618	0,020294	0,033793	0,043213	0,022528	0,005303	0,009008	0,020477	0,012916	0,019574
	C4	0,032853	0,013714	0,005302	0,013253	0,002928	0,010506	0,026437	0,038512	0,021865	0,023341	0,029344	0,017996	0,01883	0,032074	0,019861	0,037572	0,046625	0,048235	0,057262	0,01853	0,036585	0,060928	0,063617	0,031427	0,031101	0,038667	0,038358	0,042664	0,029174
	T8	0,022959	0,007519	0,012879	0,006223	0,016771	0,013775	0,004773	0,015444	0,012932	0,015733	0,008523	0,050141	0,013857	0,030636	0,030145	0,005896	0,030379	0,020176	0,042972	0,01663	0,006339	0,020494	0,028465	0,049255	0,030731	0,020735	0,034622	0,026889	0,03295
	CP5	0,024575	0,034368	0,01229	0,012135	0,021864	0,019164	0,024991	0,01786	0,019855	0,016292	0,018811	0,009481	0,026399	0,012227	0,005395	0,015023	0,035195	0,022579	0,029833	0,007834	0,023499	0,030261	0,033851	0,025134	0,00529	0,01839	0,019984	0,016361	0,014078
	CP1	0,040571	0,031784	0,020023	0,024613	0,014168	0,036121	0,019432	0,021591	0,016237	0,010473	0,027942	0,001513	0,019749	0,015376	0,016392	0,045694	0,052238	0,0225	0,039141	0,011625	0,021368	0,028923	0,03667	0,030704	0,027353	0,013069	0,018358	0,023592	0,010543
	CP2	0,02306	0,039363	0,050301	0,054233	0,032513	0,030396	0,033543	0,033836	0,037911	0,031638	0,035449	0,023308	0,047034	0,034917	0,042032	0,026968	0,037389	0,037461	0,052762	0,029986	0,032848	0,038696	0,040641	0,051069	0,040277	0,050059	0,049766	0,048263	0,050006
	CP6	0,012513	0,026267	0,019381	0,019908	0,019396	0,009203	0,025784	0,032762	0,033292	0,030684	0,040107	0,013975	0,030093	0,029465	0,047875	0,04514	0,031074	0,019045	0,03359	0,0493	0,047343	0,019948	0,026966	0,056632	0,049281	0,035163	0,032545	0,039129	0,034448
	P7	0,021118	0,020661	0,026177	0,027977	0,027314	0,032104	0,032127	0,024646	0,03895	0,041963	0,037219	0,026458	0,038892	0,036483	0,048761	0,01873	0,027272	0,044116	0,054212	0,048822	0,041248	0,046347	0,041691	0,044286	0,061939	0,059626	0,030985	0,057807	0,038379
	P3	0,050857	0,050513	0,039378	0,036057	0,030183	0,048404	0,045418	0,057877	0,056562	0,036726	0,044223	0,055231	0,057263	0,037018	0,043963	0,030777	0,072544	0,046924	0,009862	0,041225	0,031521	0,0459	0,035356	0,02719	0,043581	0,052311	0,043012	0,056077	0,036345
	Pz	0,004906	0,019403	0,022985	0,021596	0,01818	0,030245	0,034394	0,010131	0,006719	0,024088	0,03134	0,008229	0,007397	0,010659	0,018067	0,029609	0,030419	0,024324	0,017659	0,023804	0,032933	0,035175	0,036748	0,022973	0,024282	0,022049	0,008463	0,022287	0,007987
	P4	0,041341	0,026604	0,024437	0,02276	0,014365	0,037402	0,032893	0,013686	0,022462	0,015315	0,037151	0,035975	0,025224	0,005342	0,007934	0,040664	0,012379	0,02041	0,052731	0,008223	0,010176	0,026608	0,021363	0,023077	0,022411	0,019386	0,003517	0,02892	0,004141
	P8	0,051776	0,054793	0,046704	0,068865	0,066158	0,073704	0,05366	0,046068	0,057623	0,061926	0,06151	0,033389	0,050003	0,048785	0,037829	0,013371	0,047655	0,026706	0,027575	0,045437	0,024209	0,008819	0,019264	0,035828	0,025107	0,034442	0,035723	0,028041	0,045335
	POz	0,040175	0,042538	0,06244	0,059729	0,048082	0,047937	0,043688	0,025413	0,03661	0,034004	0,046334	0,028936	0,048836	0,031971	0,034944	0,043438	0,043185	0,040709	0,01493	0,030723	0,037858	0,039486	0,043605	0,038207	0,019805	0,020994	0,044416	0,012485	0,043425
	O1	0,034135	0,032606	0,03757	0,02427	0,021777	0,029629	0,02515	0,028503	0,023728	0,021113	0,035173	0,029023	0,037036	0,01561	0,007139	0,018692	0,027902	0,032015	0,009599	0,015406	0,030927	0,042782	0,04247	0,014361	0,018155	0,017703	0,023159	0,01999	0,015822
	O2	0,038422	0,045744	0,048934	0,037507	0,036202	0,043707	0,041323	0,035134	0,040915	0,033113	0,038772	0,031185	0,054242	0,042842	0,0347	0,025027	0,054962	0,050273	0,002448	0,038693	0,018079	0,051891	0,051309	0,053077	0,02304	0,023295	0,053258	0,02381	0,048455
subject 2	Fp1	0	0,026776	0,039091	0,032627	0,071612	0,026819	0,04558	0,009354	0,019313	0,046877	0,042489	0,027368	0,025717	0,003213	0,026022	0,024145	0,039094	0,024748	0,066615	0,046503	0,042098	0,037961	0,037284	0,035882	0,056895	0,046361	0,02423	0,057542	0,017135
	Fpz	0,026776	0	0,018166	0,005999	0,066835	0,029318	0,037863	0,008915	0,019125	0,036202	0,036163	0,026699	0,02316	0,010912	0,021553	0,038463	0,048827	0,017353	0,071551	0,036835	0,037549	0,025286	0,022394	0,038713	0,062322	0,039721	0,023525	0,061168	0,018841
	Fp2	0,039091	0,018166	0	0,011513	0,056709	0,021794	0,031166	0,013332	0,021269	0,025127	0,029853	0,026567	0,023971	0,020573	0,02018	0,034623	0,049923	0,016732	0,078554	0,017572	0,027258	0,017275	0,018728	0,056454	0,051717	0,034967	0,03613	0,048874	0,030759
	F7	0,032627	0,005999	0,011513	0	0,061364	0,026742	0,021575	0,031199	0,029859	0,043231	0,027684	0,050582	0,034462	0,016888	0,011707	0,038094	0,05429	0,027848	0,067383	0,025882	0,042784	0,035327	0,027925	0,030741	0,050865	0,043245	0,017999	0,059807	0,017612
	F3	0,071612	0,066835	0,056709	0,061364	0	0,035242	0,006524	0,048326	0,039274	0,006427	0,006287	0,055142	0,055434	0,022765	0,045014	0,051839	0,052344	0,064126	0,046109	0,019268	0,046371	0,068817	0,05718	0,035571	0,073892	0,05163	0,045004	0,060999	0,035423
	Fz	0,026819	0,029318	0,021794	0,026742	0,035242	0	0,009751	0,040253	0,003578	0,015243	0,001657	0,067277	0,019109	0,041917	0,058374	0,051473	0,078157	0,037488	0,077479	0,036287	0,061609	0,030104	0,022298	0,061807	0,087285	0,066295	0,054662	0,081408	0,043261
	F4	0,04558	0,037863	0,031166	0,021575	0,006524	0,009751	0	0,039823	0,023324	0,026084	0,003548	0,066745	0,031236	0,055393	0,0525	0,065271	0,072069	0,046797	0,067552	0,022833	0,0368	0,035282	0,028653	0,074003	0,063027	0,047296	0,061644	0,05854	0,058086
	F8	0,009354	0,008915	0,013332	0,031199	0,048326	0,040253	0,039823	0	0,0586	0,055742	0,045632	0,007376	0,054572	0,079004	0,068225	0,073692	0,033632	0,052752	0,091379	0,042258	0,060943	0,040772	0,03288	0,097322	0,077498	0,051694	0,083643	0,065244	0,079052
	FC5	0,019313	0,019125	0,021269	0,029859	0,039274	0,003578	0,023324	0,0586	0	0,027978	0,024204	0,0815	0,032469	0,065781	0,043513	0,062111	0,069088	0,04462	0,056037	0,03684	0,068184	0,019432	0,013026	0,051543	0,085697	0,078762	0,0773	0,08254	0,074962
	FC1	0,046877	0,036202	0,025127	0,043231	0,006427	0,015243	0,026084	0,055742	0,027978	0	0,021509	0,067768	0,031599	0,03454	0,043399	0,055237	0,071016	0,047069	0,05125	0,013194	0,051046	0,058569	0,05209	0,024489	0,07766	0,045151	0,049624	0,061933	0,033373
	FC2	0,042489	0,036163	0,029853	0,027684	0,006287	0,001657	0,003548	0,045632	0,024204	0,021509	0	0,076116	0,035263	0,03752	0,041007	0,057445	0,077472	0,041337	0,065354	0,026211	0,039458	0,03798	0,030638	0,056662	0,069117	0,056131	0,050855	0,070366	0,043774
	FC6	0,027368	0,026699	0,026567	0,050582	0,055142	0,067277	0,066745	0,007376	0,0815	0,067768	0,076116	0	0,07387	0,091693	0,084203	0,102441	0,03355	0,05962	0,1084	0,064503	0,096405	0,034811	0,028163	0,112469	0,094088	0,075279	0,093656	0,083457	0,096519
	T7	0,025717	0,02316	0,023971	0,034462	0,055434	0,019109	0,031236	0,054572	0,032469	0,031599	0,035263	0,07387	0	0,0545	0,044974	0,050731	0,06141	0,043631	0,062611	0,037665	0,068916	0,023063	0,017577	0,058623	0,079137	0,071271	0,075486	0,088182	0,065944
	C3	0,003213	0,010912	0,020573	0,016888	0,022765	0,041917	0,055393	0,079004	0,065781	0,03454	0,03752	0,091693	0,0545	0	0,049212	0,05469	0,076516	0,049846	0,034904	0,032539	0,065201	0,015521	0,009286	0,030935	0,083327	0,066542	0,071183	0,075599	0,074327
	Cz	0,026022	0,021553	0,02018	0,011707	0,045014	0,058374	0,0525	0,068225	0,043513	0,043399	0,041007	0,084203	0,044974	0,049212	0	0,061144	0,078802	0,04259	0,036542	0,060402	0,07231	0,048441	0,055245	0,07931	0,112545	0,073407	0,049464	0,076643	0,055375
	C4	0,024145	0,038463	0,034623	0,038094	0,051839	0,051473	0,065271	0,073692	0,062111	0,055237	0,057445	0,102441	0,050731	0,05469	0,061144	0	0,054202	0,032213	0,069757	0,054351	0,042645	0,029387	0,022835	0,064375	0,063578	0,071499	0,058656	0,075991	0,063914
	T8	0,039094	0,048827	0,049923	0,05429	0,052344	0,078157	0,072069	0,033632	0,069088	0,071016	0,077472	0,03355	0,06141	0,076516	0,078802	0,054202	0	0,049953	0,058016	0,066259	0,064675	0,041662	0,028913	0,087504	0,073158	0,052708	0,076292	0,054417	0,082355
	CP5	0,024748	0,017353	0,016732	0,027848	0,064126	0,037488	0,046797	0,052752	0,04462	0,047069	0,041337	0,05962	0,043631	0,049846	0,04259	0,032213	0,049953	0	0,032672	0,046292	0,05387	0,034916	0,024168	0,009699	0,053784	0,06151	0,068259	0,05782	0,043777
	CP1	0,066615	0,071551	0,078554	0,067383	0,046109	0,077479	0,067552	0,091379	0,056037	0,05125	0,065354	0,1084	0,062611	0,034904	0,036542	0,069757	0,058016	0,032672	0	0,049803	0,034929	0,014092	0,012378	0,041574	0,034901	0,034839	0,036928	0,0195	0,032079
	CP2	0,046503	0,036835	0,017572	0,025882	0,019268	0,036287	0,022833	0,042258	0,03684	0,013194	0,026211	0,064503	0,037665	0,032539	0,060402	0,054351	0,066259	0,046292	0,049803	0	0,023926	0,047728	0,049401	0,038842	0,071239	0,022115	0,049079	0,030626	0,043962
	CP6	0,042098	0,037549	0,027258	0,042784	0,046371	0,061609	0,0368	0,060943	0,068184	0,051046	0,039458	0,096405	0,068916	0,065201	0,07231	0,042645	0,064675	0,05387	0,034929	0,023926	0	0,038271	0,015533	0,065923	0,047627	0,027733	0,073678	0,031381	0,073777
	P7	0,037961	0,025286	0,017275	0,035327	0,068817	0,030104	0,035282	0,040772	0,019432	0,058569	0,03798	0,034811	0,023063	0,015521	0,048441	0,029387	0,041662	0,034916	0,014092	0,047728	0,038271	0	0,035658	0,00814	0,036782	0,034775	0,038512	0,031707	0,023545
	P3	0,037284	0,022394	0,018728	0,027925	0,05718	0,022298	0,028653	0,03288	0,013026	0,05209	0,030638	0,028163	0,017577	0,009286	0,055245	0,022835	0,028913	0,024168	0,012378	0,049401	0,015533	0,035658	0	0,037473	0,019603	0,019376	0,024928	0,021256	0,007592
	Pz	0,035882	0,038713	0,056454	0,030741	0,035571	0,061807	0,074003	0,097322	0,051543	0,024489	0,056662	0,112469	0,058623	0,030935	0,07931	0,064375	0,087504	0,009699	0,041574	0,038842	0,065923	0,00814	0,037473	0	0,110425	0,069707	0,038261	0,06321	0,042389
	P4	0,056895	0,062322	0,051717	0,050865	0,073892	0,087285	0,063027	0,077498	0,085697	0,07766	0,069117	0,094088	0,079137	0,083327	0,112545	0,063578	0,073158	0,053784	0,034901	0,071239	0,047627	0,036782	0,019603	0,110425	0	0,068015	0,093729	0,062508	0,101419
	P8	0,046361	0,039721	0,034967	0,043245	0,05163	0,066295	0,047296	0,051694	0,078762	0,045151	0,056131	0,075279	0,071271	0,066542	0,073407	0,071499	0,052708	0,06151	0,034839	0,022115	0,027733	0,034775	0,019376	0,069707	0,068015	0	0,081615	0,00345	0,07503
	POz	0,02423	0,023525	0,03613	0,017999	0,045004	0,054662	0,061644	0,083643	0,0773	0,049624	0,050855	0,093656	0,075486	0,071183	0,049464	0,058656	0,076292	0,068259	0,036928	0,049079	0,073678	0,038512	0,024928	0,038261	0,093729	0,081615	0	0,0823	0,082799
	O1	0,057542	0,061168	0,048874	0,059807	0,060999	0,081408	0,05854	0,065244	0,08254	0,061933	0,070366	0,083457	0,088182	0,075599	0,076643	0,075991	0,054417	0,05782	0,0195	0,030626	0,031381	0,031707	0,021256	0,06321	0,062508	0,00345	0,0823	0	0,07776
	O2	0,017135	0,018841	0,030759	0,017612	0,035423	0,043261	0,058086	0,079052	0,074962	0,033373	0,043774	0,096519	0,065944	0,074327	0,055375	0,063914	0,082355	0,043777	0,032079	0,043962	0,073777	0,023545	0,007592	0,042389	0,101419	0,07503	0,082799	0,07776	0
