# Supplementary material for: Hyperbrain features of team mental models within a juggling paradigm: a proof of concept
Source: PeerJ. 2016 Sep 20;4:e2457. doi: 10.7717/peerj.2457 (PMC5036110; doi:10.7717/peerj.2457)
Supplement: Supplemental Information 2 [file peerj-04-2457-s002.pdf]

Rows: subject 1 — Columns: subject 1 (Fp1–C4)

subject 1	Fp1	Fpz	Fp2	F7	F3	Fz	F4	F8	FC5	FC1	FC2	FC6	T7	C3	Cz	C4
Fp1	0	0,009451	0,003696	0,068393	0,036847	0,022896	0,053831	0,026465	0,098034	0,031143	0,060105	0,055818	0,095526	0,076398	0,060856	0,049857
Fpz	0,009451	0	0,009883	0,053011	0,026038	0,021561	0,056466	0,027639	0,082626	0,049937	0,049156	0,051936	0,092801	0,045848	0,054649	0,045093
Fp2	0,003696	0,009883	0	0,06628	0,025679	0,023834	0,051463	0,02878	0,098279	0,035044	0,048258	0,044683	0,093974	0,071518	0,053575	0,043477
F7	0,068393	0,053011	0,06628	0	0,051682	0,052377	0,058487	0,027546	0,04639	0,076883	0,019894	0,038328	0,048435	0,029416	0,023892	0,023167
F3	0,036847	0,026038	0,025679	0,051682	0	0,039392	0,04932	0,012285	0,086932	0,062938	0,044566	0,05603	0,097099	0,058936	0,043758	0,049695
Fz	0,022896	0,021561	0,023834	0,052377	0,039392	0	0,051333	0,0201	0,08164	0,050036	0,033939	0,040416	0,074989	0,064178	0,041526	0,022743
F4	0,053831	0,056466	0,051463	0,058487	0,04932	0,051333	0	0,012461	0,047437	0,044923	0,041627	0,05814	0,063936	0,056467	0,048439	0,036858
F8	0,026465	0,027639	0,02878	0,027546	0,012285	0,0201	0,012461	0	0,043214	0,023906	0,051984	0,014804	0,020426	0,03021	0,036311	0,047542
FC5	0,098034	0,082626	0,098279	0,04639	0,086932	0,08164	0,047437	0,043214	0	0,095839	0,055129	0,062605	0,085794	0,018284	0,055705	0,033701
FC1	0,031143	0,049937	0,035044	0,076883	0,062938	0,050036	0,044923	0,023906	0,095839	0	0,056834	0,049402	0,087816	0,081269	0,048196	0,031609
FC2	0,060105	0,049156	0,048258	0,019894	0,044566	0,033939	0,041627	0,051984	0,055129	0,056834	0	0,032184	0,045507	0,049216	0,021973	0,016017
FC6	0,055818	0,051936	0,044683	0,038328	0,05603	0,040416	0,05814	0,014804	0,062605	0,049402	0,032184	0	0,05036	0,062901	0,027958	0,037222
T7	0,095526	0,092801	0,093974	0,048435	0,097099	0,074989	0,063936	0,020426	0,085794	0,087816	0,045507	0,05036	0	0,082233	0,051304	0,037006
C3	0,076398	0,045848	0,071518	0,029416	0,058936	0,064178	0,056467	0,03021	0,018284	0,081269	0,049216	0,062901	0,082233	0	0,054666	0,038357
Cz	0,060856	0,054649	0,053575	0,023892	0,043758	0,041526	0,048439	0,036311	0,055705	0,048196	0,021973	0,027958	0,051304	0,054666	0	0,031701
C4	0,049857	0,045093	0,043477	0,023167	0,049695	0,022743	0,036858	0,047542	0,033701	0,031609	0,016017	0,037222	0,037006	0,038357	0,031701	0
T8	0,054253	0,062938	0,050039	0,046947	0,045118	0,061194	0,025328	0,017425	0,03844	0,026543	0,049467	0,054467	0,052167	0,048052	0,058619	0,048446
CP5	0,036156	0,030104	0,034394	0,065314	0,046965	0,03798	0,053761	0,024074	0,079398	0,05377	0,058141	0,052509	0,085435	0,060261	0,048971	0,047398
CP1	0,09748	0,090688	0,088609	0,04479	0,077695	0,075926	0,008539	0,03586	0,060885	0,050267	0,045738	0,044404	0,058477	0,071363	0,021272	0,016521
CP2	0,026049	0,025558	0,032417	0,032439	0,031563	0,032306	0,018321	0,042831	0,03003	0,019184	0,045726	0,039426	0,056581	0,024487	0,038774	0,047231
CP6	0,02539	0,016942	0,022593	0,035657	0,028397	0,033182	0,030255	0,0519	0,042687	0,027388	0,045137	0,038018	0,068975	0,025561	0,040492	0,042918
P7	0,008649	0,009367	0,007061	0,024751	0,024777	0,017986	0,057559	0,030284	0,023954	0,03074	0,017396	0,017981	0,017042	0,01311	0,0154	0,021931
P3	0,044522	0,038377	0,047703	0,054031	0,040802	0,048986	0,031108	0,036964	0,054593	0,063249	0,055109	0,040579	0,076939	0,037978	0,044307	0,052044
Pz	0,036639	0,045635	0,039155	0,067717	0,055944	0,037985	0,048186	0,029849	0,032153	0,041008	0,072084	0,060193	0,069112	0,060531	0,070102	0,054797
P4	0,053429	0,049084	0,045068	0,043439	0,027451	0,052028	0,01833	0,035063	0,029098	0,030949	0,051553	0,055719	0,054814	0,056004	0,051696	0,043302
P8	0,036895	0,02326	0,033649	0,035183	0,036837	0,028116	0,016178	0,025039	0,03371	0,072633	0,031563	0,031343	0,033829	0,017912	0,032049	0,044996
POz	0,052361	0,049213	0,048727	0,041687	0,050523	0,034193	0,027832	0,0352	0,033006	0,047605	0,035786	0,044355	0,044164	0,049988	0,039397	0,029822
O1	0,044253	0,064461	0,051526	0,079127	0,059158	0,060978	0,068822	0,027558	0,035215	0,038134	0,073074	0,072009	0,057439	0,048762	0,068147	0,059535
O2	0,047044	0,039408	0,03848	0,044761	0,022894	0,029775	0,034848	0,018621	0,04816	0,061649	0,028019	0,036107	0,066879	0,042282	0,031044	0,02311

Rows: subject 1 — Columns: subject 1 (T8–O2)

subject 1	T8	CP5	CP1	CP2	CP6	P7	P3	Pz	P4	P8	POz	O1	O2
Fp1	0,054253	0,036156	0,09748	0,026049	0,02539	0,008649	0,044522	0,036639	0,053429	0,036895	0,052361	0,044253	0,047044
Fpz	0,062938	0,030104	0,090688	0,025558	0,016942	0,009367	0,038377	0,045635	0,049084	0,02326	0,049213	0,064461	0,039408
Fp2	0,050039	0,034394	0,088609	0,032417	0,022593	0,007061	0,047703	0,039155	0,045068	0,033649	0,048727	0,051526	0,03848
F7	0,046947	0,065314	0,04479	0,032439	0,035657	0,024751	0,054031	0,067717	0,043439	0,035183	0,041687	0,079127	0,044761
F3	0,045118	0,046965	0,077695	0,031563	0,028397	0,024777	0,040802	0,055944	0,027451	0,036837	0,050523	0,059158	0,022894
Fz	0,061194	0,03798	0,075926	0,032306	0,033182	0,017986	0,048986	0,037985	0,052028	0,028116	0,034193	0,060978	0,029775
F4	0,025328	0,053761	0,008539	0,018321	0,030255	0,057559	0,031108	0,048186	0,01833	0,016178	0,027832	0,068822	0,034848
F8	0,017425	0,024074	0,03586	0,042831	0,0519	0,030284	0,036964	0,029849	0,035063	0,025039	0,0352	0,027558	0,018621
FC5	0,03844	0,079398	0,060885	0,03003	0,042687	0,023954	0,054593	0,032153	0,029098	0,03371	0,033006	0,035215	0,04816
FC1	0,026543	0,05377	0,050267	0,019184	0,027388	0,03074	0,063249	0,041008	0,030949	0,072633	0,047605	0,038134	0,061649
FC2	0,049467	0,058141	0,045738	0,045726	0,045137	0,017396	0,055109	0,072084	0,051553	0,031563	0,035786	0,073074	0,028019
FC6	0,054467	0,052509	0,044404	0,039426	0,038018	0,017981	0,040579	0,060193	0,055719	0,031343	0,044355	0,072009	0,036107
T7	0,052167	0,085435	0,058477	0,056581	0,068975	0,017042	0,076939	0,069112	0,054814	0,033829	0,044164	0,057439	0,066879
C3	0,048052	0,060261	0,071363	0,024487	0,025561	0,01311	0,037978	0,060531	0,056004	0,017912	0,049988	0,048762	0,042282
Cz	0,058619	0,048971	0,021272	0,038774	0,040492	0,0154	0,044307	0,070102	0,051696	0,032049	0,039397	0,068147	0,031044
C4	0,048446	0,047398	0,016521	0,047231	0,042918	0,021931	0,052044	0,054797	0,043302	0,044996	0,029822	0,059535	0,02311
T8	0	0,057146	0,036332	0,070687	0,04339	0,042033	0,055197	0,046137	0,036877	0,054978	0,048461	0,015981	0,037494
CP5	0,057146	0	0,08322	0,020648	0,035318	0,017376	0,032616	0,05152	0,046969	0,019115	0,050041	0,054417	0,044485
CP1	0,036332	0,08322	0	0,057303	0,055625	0,040493	0,066459	0,084392	0,02656	0,067366	0,017705	0,023144	0,055377
CP2	0,070687	0,020648	0,057303	0	0,036474	0,031794	0,042884	0,015337	0,063931	0,041922	0,033249	0,05208	0,036089
CP6	0,04339	0,035318	0,055625	0,036474	0	0,021093	0,02487	0,017864	0,065963	0,047793	0,033972	0,022914	0,031263
P7	0,042033	0,017376	0,040493	0,031794	0,021093	0	0,035907	0,015626	0,049874	0,037465	0,039292	0,056691	0,04447
P3	0,055197	0,032616	0,066459	0,042884	0,02487	0,035907	0	0,043468	0,029623	0,039274	0,042795	0,041307	0,032008
Pz	0,046137	0,05152	0,084392	0,015337	0,017864	0,015626	0,043468	0	0,061149	0,009325	0,044399	0,035529	0,049158
P4	0,036877	0,046969	0,02656	0,063931	0,065963	0,049874	0,029623	0,061149	0	0,059392	0,035617	0,021419	0,020059
P8	0,054978	0,019115	0,067366	0,041922	0,047793	0,037465	0,039274	0,009325	0,059392	0	0,03465	0,011816	0,031996
POz	0,048461	0,050041	0,017705	0,033249	0,033972	0,039292	0,042795	0,044399	0,035617	0,03465	0	0,073373	0,026327
O1	0,015981	0,054417	0,023144	0,05208	0,022914	0,056691	0,041307	0,035529	0,021419	0,011816	0,073373	0	0,054877
O2	0,037494	0,044485	0,055377	0,036089	0,031263	0,04447	0,032008	0,049158	0,020059	0,031996	0,026327	0,054877	0

Rows: subject 1 — Columns: subject 2 (Fp1–C4)

subject 1	Fp1	Fpz	Fp2	F7	F3	Fz	F4	F8	FC5	FC1	FC2	FC6	T7	C3	Cz	C4
Fp1	0,053095	0,036294	0,032752	0,048963	0,042501	0,009229	0,014624	0,028667	0,014379	0,016396	0,019665	0,02243	0,013041	0,018791	0,008319	0,009739
Fpz	0,056388	0,040646	0,039802	0,055533	0,032509	0,008815	0,023069	0,040675	0,022166	0,022283	0,027134	0,033116	0,019202	0,016682	0,023036	0,016415
Fp2	0,046966	0,036481	0,032013	0,046888	0,041877	0,008381	0,013617	0,029605	0,015037	0,020485	0,014887	0,022572	0,014433	0,019976	0,010688	0,004863
F7	0,035894	0,029335	0,041718	0,037005	0,026805	0,026966	0,029728	0,049199	0,036173	0,033498	0,037068	0,052985	0,032691	0,03223	0,033861	0,029485
F3	0,04762	0,03849	0,03958	0,047813	0,01394	0,008138	0,006883	0,035072	0,016893	0,029075	0,023052	0,027319	0,019511	0,022251	0,022961	0,010053
Fz	0,056059	0,030337	0,029312	0,043132	0,034601	0,00899	0,016798	0,039912	0,02069	0,022456	0,022317	0,037167	0,018394	0,024715	0,020462	0,01424
F4	0,033499	0,033966	0,03526	0,041628	0,045024	0,051103	0,045388	0,046944	0,031318	0,027395	0,043386	0,041211	0,019131	0,021238	0,025552	0,034447
F8	0,019822	0,03449	0,035118	0,018104	0,017304	0,014747	0,02733	0,010137	0,024506	0,011289	0,024267	0,031049	0,029495	0,01395	0,003805	0,027089
FC5	0,034039	0,032412	0,037155	0,046367	0,008706	0,021213	0,019243	0,032029	0,048828	0,046478	0,044397	0,041881	0,04282	0,039707	0,04824	0,035709
FC1	0,057072	0,043685	0,032424	0,035984	0,05091	0,036925	0,027761	0,020035	0,023967	0,032887	0,018961	0,023019	0,023076	0,036883	0,019941	0,030178
FC2	0,049091	0,038504	0,041313	0,038961	0,026657	0,015739	0,030545	0,050269	0,026856	0,022243	0,031014	0,050877	0,026388	0,037358	0,026328	0,014805
FC6	0,043016	0,03041	0,036369	0,043482	0,024332	0,036284	0,031033	0,045075	0,035772	0,02787	0,03814	0,041263	0,034487	0,024719	0,036324	0,0217
T7	0,026842	0,021612	0,033087	0,036933	0,047191	0,038527	0,035737	0,04763	0,032531	0,030563	0,04742	0,05211	0,029751	0,023129	0,029682	0,02496
C3	0,038243	0,027449	0,034463	0,054176	0,013268	0,008371	0,022542	0,037671	0,03938	0,03835	0,035881	0,041713	0,031203	0,034528	0,039334	0,032616
Cz	0,027623	0,016363	0,024486	0,029771	0,025697	0,036063	0,040606	0,041037	0,02876	0,024039	0,032289	0,044219	0,02856	0,027953	0,026385	0,022255
C4	0,037833	0,032406	0,032356	0,040036	0,022279	0,016829	0,025821	0,046875	0,015576	0,020739	0,020434	0,043711	0,018099	0,033482	0,022131	0,013629
T8	0,025994	0,037575	0,034231	0,048152	0,052447	0,043307	0,047717	0,034471	0,047657	0,024428	0,03615	0,050822	0,056688	0,026348	0,027945	0,010932
CP5	0,056989	0,037489	0,024714	0,040135	0,031448	0,013851	0,032421	0,037582	0,027668	0,027157	0,041403	0,034464	0,020194	0,015443	0,020956	0,018521
CP1	0,052041	0,056818	0,045564	0,021045	0,054585	0,049351	0,047274	0,035933	0,044001	0,050995	0,050895	0,049326	0,043357	0,031947	0,030282	0,048302
CP2	0,015863	0,025819	0,030576	0,027054	0,01901	0,019709	0,021471	0,029843	0,012354	0,013613	0,013589	0,03431	0,014387	0,018275	0,015657	0,0139
CP6	0,027715	0,029655	0,033	0,028559	0,019584	0,023686	0,032546	0,027878	0,033748	0,018004	0,020785	0,031563	0,036221	0,013069	0,009698	0,048818
P7	0,015861	0,026527	0,032542	0,014344	0,036809	0,033315	0,044128	0,034229	0,018535	0,038377	0,049517	0,023847	0,028107	0,007668	0,026028	0,043457
P3	0,049536	0,031538	0,02896	0,034506	0,019702	0,033519	0,035116	0,038906	0,037492	0,027552	0,035005	0,0343	0,020563	0,013975	0,026635	0,008093
Pz	0,033176	0,016868	0,029202	0,035094	0,030888	0,021463	0,02318	0,035254	0,014259	0,019532	0,024516	0,021398	0,009166	0,025258	0,011717	0,036442
P4	0,023609	0,040489	0,025965	0,028902	0,057023	0,040092	0,030811	0,031667	0,03635	0,022422	0,025932	0,048765	0,03976	0,020599	0,014736	0,013514
P8	0,026336	0,031805	0,032227	0,021671	0,041553	0,030519	0,040538	0,032713	0,026057	0,055065	0,042353	0,021882	0,021473	0,033599	0,043508	0,047329
POz	0,0352	0,026451	0,029683	0,033128	0,032901	0,034658	0,041795	0,058032	0,023487	0,030949	0,041681	0,052287	0,023939	0,033721	0,02854	0,032451
O1	0,007807	0,010126	0,006338	0,012741	0,029546	0,025922	0,040483	0,022352	0,026693	0,011111	0,021652	0,012787	0,028641	0,036632	0,011366	0,024283
O2	0,053395	0,042516	0,050215	0,043019	0,019244	0,021076	0,016605	0,045381	0,021232	0,035459	0,022733	0,043567	0,027496	0,032763	0,029596	0,020351

Rows: subject 1 — Columns: subject 2 (T8–O2)

subject 1	T8	CP5	CP1	CP2	CP6	P7	P3	Pz	P4	P8	POz	O1	O2
Fp1	0,02864	0,027874	0,010337	0,018966	0,019021	0,032655	0,039074	0,017233	0,014299	0,024736	0,023636	0,029742	0,020403
Fpz	0,035966	0,01705	0,023162	0,033579	0,033524	0,032552	0,034231	0,01136	0,031531	0,036612	0,011352	0,036435	0,013623
Fp2	0,026409	0,027314	0,011322	0,019203	0,017945	0,035506	0,040685	0,018582	0,015301	0,023569	0,023214	0,025193	0,021603
F7	0,047072	0,024562	0,040833	0,039142	0,044983	0,034803	0,034338	0,029842	0,038579	0,041684	0,028237	0,050991	0,03502
F3	0,026782	0,019674	0,024739	0,025792	0,026848	0,038804	0,03536	0,016522	0,020373	0,027773	0,015403	0,02932	0,016486
Fz	0,03876	0,040666	0,016712	0,028048	0,026493	0,042834	0,041709	0,023071	0,02146	0,02455	0,029456	0,025341	0,02474
F4	0,05073	0,032643	0,019835	0,026974	0,032011	0,029617	0,037828	0,024338	0,025027	0,027458	0,031378	0,029104	0,019994
F8	0,037658	0,019627	0,021542	0,0169	0,011995	0,021204	0,005771	0,013659	0,021556	0,014745	0,020396	0,016075	0,024941
FC5	0,035029	0,019417	0,032501	0,056738	0,048538	0,040319	0,031162	0,046553	0,049987	0,051562	0,041713	0,043219	0,060145
FC1	0,028712	0,059116	0,01065	0,013366	0,016601	0,0519	0,057396	0,044491	0,015685	0,017409	0,051661	0,017265	0,053702
FC2	0,04991	0,02873	0,031221	0,029052	0,040471	0,043162	0,030342	0,027522	0,025089	0,031801	0,031361	0,034145	0,032197
FC6	0,050213	0,036638	0,024466	0,039002	0,043567	0,03818	0,050241	0,029677	0,030834	0,035683	0,028887	0,035355	0,040286
T7	0,057991	0,0096	0,041536	0,036779	0,034928	0,029925	0,032933	0,045005	0,032549	0,043034	0,015815	0,033552	0,010808
C3	0,044411	0,022496	0,043505	0,047069	0,0448	0,044262	0,029921	0,036067	0,038942	0,050327	0,031674	0,043828	0,039467
Cz	0,04965	0,032613	0,023806	0,032746	0,032389	0,037559	0,035987	0,028925	0,027372	0,031564	0,034972	0,033454	0,035099
C4	0,052525	0,026385	0,028727	0,028462	0,031277	0,046504	0,030013	0,025754	0,025692	0,02933	0,027955	0,034338	0,035805
T8	0,042798	0,039591	0,034274	0,035365	0,034609	0,056632	0,042685	0,037789	0,037445	0,042192	0,035084	0,039517	0,031057
CP5	0,041923	0,016177	0,021335	0,029926	0,026738	0,031833	0,031142	0,016051	0,024372	0,033329	0,015099	0,036177	0,014865
CP1	0,029669	0,050659	0,018775	0,033976	0,040044	0,061493	0,052225	0,050164	0,030508	0,031907	0,061017	0,027329	0,054065
CP2	0,045329	0,028678	0,034783	0,020965	0,021221	0,034897	0,020653	0,023254	0,024037	0,024697	0,024474	0,027059	0,017363
CP6	0,024938	0,024358	0,027507	0,009316	0,022495	0,021652	0,021903	0,018386	0,018152	0,020605	0,020074	0,029693	0,017763
P7	0,035616	0,01957	0,045127	0,052331	0,053263	0,03994	0,023228	0,029418	0,055375	0,055377	0,01707	0,055674	0,02857
P3	0,041955	0,020192	0,019337	0,029325	0,02929	0,038783	0,026038	0,018294	0,023346	0,034015	0,015193	0,02944	0,020796
Pz	0,029961	0,037371	0,021029	0,021822	0,023994	0,039252	0,034624	0,025041	0,023903	0,017405	0,023205	0,021485	0,025093
P4	0,026353	0,052144	0,022061	0,018842	0,023775	0,063394	0,046838	0,029908	0,019123	0,034782	0,039692	0,035336	0,034521
P8	0,039135	0,02502	0,034251	0,047451	0,044526	0,012123	0,006839	0,041237	0,042793	0,049771	0,038456	0,056649	0,043013
POz	0,053547	0,032734	0,037278	0,039759	0,043533	0,046476	0,030719	0,026649	0,039376	0,033005	0,034303	0,024723	0,033774
O1	0,028879	0,044196	0,021222	0,011436	0,025795	0,055911	0,037092	0,028112	0,010373	0,012985	0,027314	0,00742	0,02703
O2	0,05595	0,02212	0,023337	0,026699	0,031893	0,039534	0,02082	0,027939	0,024899	0,026576	0,021739	0,023527	0,025099

Rows: subject 2 — Columns: subject 1 (Fp1–C4)

subject 2	Fp1	Fpz	Fp2	F7	F3	Fz	F4	F8	FC5	FC1	FC2	FC6	T7	C3	Cz	C4
Fp1	0,053095	0,056388	0,046966	0,035894	0,04762	0,056059	0,033499	0,019822	0,034039	0,057072	0,049091	0,043016	0,026842	0,038243	0,027623	0,037833
Fpz	0,036294	0,040646	0,036481	0,029335	0,03849	0,030337	0,033966	0,03449	0,032412	0,043685	0,038504	0,03041	0,021612	0,027449	0,016363	0,032406
Fp2	0,032752	0,039802	0,032013	0,041718	0,03958	0,029312	0,03526	0,035118	0,037155	0,032424	0,041313	0,036369	0,033087	0,034463	0,024486	0,032356
F7	0,048963	0,055533	0,046888	0,037005	0,047813	0,043132	0,041628	0,018104	0,046367	0,035984	0,038961	0,043482	0,036933	0,054176	0,029771	0,040036
F3	0,042501	0,032509	0,041877	0,026805	0,01394	0,034601	0,045024	0,017304	0,008706	0,05091	0,026657	0,024332	0,047191	0,013268	0,025697	0,022279
Fz	0,009229	0,008815	0,008381	0,026966	0,008138	0,00899	0,051103	0,014747	0,021213	0,036925	0,015739	0,036284	0,038527	0,008371	0,036063	0,016829
F4	0,014624	0,023069	0,013617	0,029728	0,006883	0,016798	0,045388	0,02733	0,019243	0,027761	0,030545	0,031033	0,035737	0,022542	0,040606	0,025821
F8	0,028667	0,040675	0,029605	0,049199	0,035072	0,039912	0,046944	0,010137	0,032029	0,020035	0,050269	0,045075	0,04763	0,037671	0,041037	0,046875
FC5	0,014379	0,022166	0,015037	0,036173	0,016893	0,02069	0,031318	0,024506	0,048828	0,023967	0,026856	0,035772	0,032531	0,03938	0,02876	0,015576
FC1	0,016396	0,022283	0,020485	0,033498	0,029075	0,022456	0,027395	0,011289	0,046478	0,032887	0,022243	0,02787	0,030563	0,03835	0,024039	0,020739
FC2	0,019665	0,027134	0,014887	0,037068	0,023052	0,022317	0,043386	0,024267	0,044397	0,018961	0,031014	0,03814	0,04742	0,035881	0,032289	0,020434
FC6	0,02243	0,033116	0,022572	0,052985	0,027319	0,037167	0,041211	0,031049	0,041881	0,023019	0,050877	0,041263	0,05211	0,041713	0,044219	0,043711
T7	0,013041	0,019202	0,014433	0,032691	0,019511	0,018394	0,019131	0,029495	0,04282	0,023076	0,026388	0,034487	0,029751	0,031203	0,02856	0,018099
C3	0,018791	0,016682	0,019976	0,03223	0,022251	0,024715	0,021238	0,01395	0,039707	0,036883	0,037358	0,024719	0,023129	0,034528	0,027953	0,033482
Cz	0,008319	0,023036	0,010688	0,033861	0,022961	0,020462	0,025552	0,003805	0,04824	0,019941	0,026328	0,036324	0,029682	0,039334	0,026385	0,022131
C4	0,009739	0,016415	0,004863	0,029485	0,010053	0,01424	0,034447	0,027089	0,035709	0,030178	0,014805	0,0217	0,02496	0,032616	0,022255	0,013629
T8	0,02864	0,035966	0,026409	0,047072	0,026782	0,03876	0,05073	0,037658	0,035029	0,028712	0,04991	0,050213	0,057991	0,044411	0,04965	0,052525
CP5	0,027874	0,01705	0,027314	0,024562	0,019674	0,040666	0,032643	0,019627	0,019417	0,059116	0,02873	0,036638	0,0096	0,022496	0,032613	0,026385
CP1	0,010337	0,023162	0,011322	0,040833	0,024739	0,016712	0,019835	0,021542	0,032501	0,01065	0,031221	0,024466	0,041536	0,043505	0,023806	0,028727
CP2	0,018966	0,033579	0,019203	0,039142	0,025792	0,028048	0,026974	0,0169	0,056738	0,013366	0,029052	0,039002	0,036779	0,047069	0,032746	0,028462
CP6	0,019021	0,033524	0,017945	0,044983	0,026848	0,026493	0,032011	0,011995	0,048538	0,016601	0,040471	0,043567	0,034928	0,0448	0,032389	0,031277
P7	0,032655	0,032552	0,035506	0,034803	0,038804	0,042834	0,029617	0,021204	0,040319	0,0519	0,043162	0,03818	0,029925	0,044262	0,037559	0,046504
P3	0,039074	0,034231	0,040685	0,034338	0,03536	0,041709	0,037828	0,005771	0,031162	0,057396	0,030342	0,050241	0,032933	0,029921	0,035987	0,030013
Pz	0,017233	0,01136	0,018582	0,029842	0,016522	0,023071	0,024338	0,013659	0,046553	0,044491	0,027522	0,029677	0,045005	0,036067	0,028925	0,025754
P4	0,014299	0,031531	0,015301	0,038579	0,020373	0,02146	0,025027	0,021556	0,049987	0,015685	0,025089	0,030834	0,032549	0,038942	0,027372	0,025692
P8	0,024736	0,036612	0,023569	0,041684	0,027773	0,02455	0,027458	0,014745	0,051562	0,017409	0,031801	0,035683	0,043034	0,050327	0,031564	0,02933
POz	0,023636	0,011352	0,023214	0,028237	0,015403	0,029456	0,031378	0,020396	0,041713	0,051661	0,031361	0,028887	0,015815	0,031674	0,034972	0,027955
O1	0,029742	0,036435	0,025193	0,050991	0,02932	0,025341	0,029104	0,016075	0,043219	0,017265	0,034145	0,035355	0,033552	0,043828	0,033454	0,034338
O2	0,020403	0,013623	0,021603	0,03502	0,016486	0,02474	0,019994	0,024941	0,060145	0,053702	0,032197	0,040286	0,010808	0,039467	0,035099	0,035805

Rows: subject 2 — Columns: subject 1 (T8–O2)

subject 2	T8	CP5	CP1	CP2	CP6	P7	P3	Pz	P4	P8	POz	O1	O2
Fp1	0,025994	0,056989	0,052041	0,015863	0,027715	0,015861	0,049536	0,033176	0,023609	0,026336	0,0352	0,007807	0,053395
Fpz	0,037575	0,037489	0,056818	0,025819	0,029655	0,026527	0,031538	0,016868	0,040489	0,031805	0,026451	0,010126	0,042516
Fp2	0,034231	0,024714	0,045564	0,030576	0,033	0,032542	0,02896	0,029202	0,025965	0,032227	0,029683	0,006338	0,050215
F7	0,048152	0,040135	0,021045	0,027054	0,028559	0,014344	0,034506	0,035094	0,028902	0,021671	0,033128	0,012741	0,043019
F3	0,052447	0,031448	0,054585	0,01901	0,019584	0,036809	0,019702	0,030888	0,057023	0,041553	0,032901	0,029546	0,019244
Fz	0,043307	0,013851	0,049351	0,019709	0,023686	0,033315	0,033519	0,021463	0,040092	0,030519	0,034658	0,025922	0,021076
F4	0,047717	0,032421	0,047274	0,021471	0,032546	0,044128	0,035116	0,02318	0,030811	0,040538	0,041795	0,040483	0,016605
F8	0,034471	0,037582	0,035933	0,029843	0,027878	0,034229	0,038906	0,035254	0,031667	0,032713	0,058032	0,022352	0,045381
FC5	0,047657	0,027668	0,044001	0,012354	0,033748	0,018535	0,037492	0,014259	0,03635	0,026057	0,023487	0,026693	0,021232
FC1	0,024428	0,027157	0,050995	0,013613	0,018004	0,038377	0,027552	0,019532	0,022422	0,055065	0,030949	0,011111	0,035459
FC2	0,03615	0,041403	0,050895	0,013589	0,020785	0,049517	0,035005	0,024516	0,025932	0,042353	0,041681	0,021652	0,022733
FC6	0,050822	0,034464	0,049326	0,03431	0,031563	0,023847	0,0343	0,021398	0,048765	0,021882	0,052287	0,012787	0,043567
T7	0,056688	0,020194	0,043357	0,014387	0,036221	0,028107	0,020563	0,009166	0,03976	0,021473	0,023939	0,028641	0,027496
C3	0,026348	0,015443	0,031947	0,018275	0,013069	0,007668	0,013975	0,025258	0,020599	0,033599	0,033721	0,036632	0,032763
Cz	0,027945	0,020956	0,030282	0,015657	0,009698	0,026028	0,026635	0,011717	0,014736	0,043508	0,02854	0,011366	0,029596
C4	0,010932	0,018521	0,048302	0,0139	0,048818	0,043457	0,008093	0,036442	0,013514	0,047329	0,032451	0,024283	0,020351
T8	0,042798	0,041923	0,029669	0,045329	0,024938	0,035616	0,041955	0,029961	0,026353	0,039135	0,053547	0,028879	0,05595
CP5	0,039591	0,016177	0,050659	0,028678	0,024358	0,01957	0,020192	0,037371	0,052144	0,02502	0,032734	0,044196	0,02212
CP1	0,034274	0,021335	0,018775	0,034783	0,027507	0,045127	0,019337	0,021029	0,022061	0,034251	0,037278	0,021222	0,023337
CP2	0,035365	0,029926	0,033976	0,020965	0,009316	0,052331	0,029325	0,021822	0,018842	0,047451	0,039759	0,011436	0,026699
CP6	0,034609	0,026738	0,040044	0,021221	0,022495	0,053263	0,02929	0,023994	0,023775	0,044526	0,043533	0,025795	0,031893
P7	0,056632	0,031833	0,061493	0,034897	0,021652	0,03994	0,038783	0,039252	0,063394	0,012123	0,046476	0,055911	0,039534
P3	0,042685	0,031142	0,052225	0,020653	0,021903	0,023228	0,026038	0,034624	0,046838	0,006839	0,030719	0,037092	0,02082
Pz	0,037789	0,016051	0,050164	0,023254	0,018386	0,029418	0,018294	0,025041	0,029908	0,041237	0,026649	0,028112	0,027939
P4	0,037445	0,024372	0,030508	0,024037	0,018152	0,055375	0,023346	0,023903	0,019123	0,042793	0,039376	0,010373	0,024899
P8	0,042192	0,033329	0,031907	0,024697	0,020605	0,055377	0,034015	0,017405	0,034782	0,049771	0,033005	0,012985	0,026576
POz	0,035084	0,015099	0,061017	0,024474	0,020074	0,01707	0,015193	0,023205	0,039692	0,038456	0,034303	0,027314	0,021739
O1	0,039517	0,036177	0,027329	0,027059	0,029693	0,055674	0,02944	0,021485	0,035336	0,056649	0,024723	0,00742	0,023527
O2	0,031057	0,014865	0,054065	0,017363	0,017763	0,02857	0,020796	0,025093	0,034521	0,043013	0,033774	0,02703	0,025099

Rows: subject 2 — Columns: subject 2 (Fp1–C4)

subject 2	Fp1	Fpz	Fp2	F7	F3	Fz	F4	F8	FC5	FC1	FC2	FC6	T7	C3	Cz	C4
Fp1	0	0,044658	0,051749	0,026945	0,067045	0,062267	0,071292	0,075826	0,069856	0,075523	0,075123	0,076544	0,076162	0,072829	0,061974	0,069937
Fpz	0,044658	0	0,014147	0,013036	0,065168	0,072595	0,077064	0,065637	0,061948	0,065349	0,057381	0,061498	0,062345	0,068061	0,054178	0,061781
Fp2	0,051749	0,014147	0	0,019588	0,076585	0,067458	0,058136	0,037334	0,04339	0,049983	0,043839	0,046491	0,041345	0,0625	0,050261	0,054915
F7	0,026945	0,013036	0,019588	0	0,083139	0,066273	0,067874	0,048574	0,056746	0,079964	0,059762	0,049247	0,061488	0,077737	0,058991	0,063406
F3	0,067045	0,065168	0,076585	0,083139	0	0,071586	0,069277	0,086443	0,064075	0,067722	0,060095	0,082417	0,055565	0,049804	0,054244	0,065842
Fz	0,062267	0,072595	0,067458	0,066273	0,071586	0	0,029962	0,06969	0,047861	0,048722	0,041189	0,059229	0,03193	0,037901	0,044788	0,056752
F4	0,071292	0,077064	0,058136	0,067874	0,069277	0,029962	0	0,054263	0,045238	0,024796	0,031687	0,055161	0,025837	0,030177	0,034335	0,049502
F8	0,075826	0,065637	0,037334	0,048574	0,086443	0,06969	0,054263	0	0,036227	0,058814	0,042821	0,009842	0,033105	0,054844	0,064985	0,074718
FC5	0,069856	0,061948	0,04339	0,056746	0,064075	0,047861	0,045238	0,036227	0	0,038237	0,026798	0,023219	0,009875	0,037602	0,03239	0,048872
FC1	0,075523	0,065349	0,049983	0,079964	0,067722	0,048722	0,024796	0,058814	0,038237	0	0,026296	0,050501	0,043328	0,028378	0,035939	0,038986
FC2	0,075123	0,057381	0,043839	0,059762	0,060095	0,041189	0,031687	0,042821	0,026798	0,026296	0	0,044424	0,021415	0,031185	0,022194	0,029482
FC6	0,076544	0,061498	0,046491	0,049247	0,082417	0,059229	0,055161	0,009842	0,023219	0,050501	0,044424	0	0,019659	0,054723	0,051261	0,067646
T7	0,076162	0,062345	0,041345	0,061488	0,055565	0,03193	0,025837	0,033105	0,009875	0,043328	0,021415	0,019659	0	0,05001	0,052361	0,05053
C3	0,072829	0,068061	0,0625	0,077737	0,049804	0,037901	0,030177	0,054844	0,037602	0,028378	0,031185	0,054723	0,05001	0	0,0252	0,038025
Cz	0,061974	0,054178	0,050261	0,058991	0,054244	0,044788	0,034335	0,064985	0,03239	0,035939	0,022194	0,051261	0,052361	0,0252	0	0,037428
C4	0,069937	0,061781	0,054915	0,063406	0,065842	0,056752	0,049502	0,074718	0,048872	0,038986	0,029482	0,067646	0,05053	0,038025	0,037428	0
T8	0,067886	0,057379	0,051037	0,04465	0,083204	0,064412	0,05065	0,022818	0,048426	0,046045	0,039439	0,033753	0,043822	0,058299	0,052696	0,063759
CP5	0,070873	0,061704	0,058033	0,066977	0,025771	0,032467	0,031212	0,043701	0,03513	0,029525	0,037375	0,037498	0,038591	0,018653	0,0452	0,039943
CP1	0,04396	0,042696	0,034546	0,031626	0,073629	0,0411	0,046502	0,053576	0,03411	0,064662	0,043987	0,047927	0,039742	0,04957	0,05552	0,063408
CP2	0,066001	0,048279	0,042866	0,053925	0,05665	0,042263	0,04253	0,060453	0,031933	0,050011	0,010074	0,057111	0,048654	0,03831	0,031396	0,040155
CP6	0,06303	0,045435	0,040945	0,042308	0,09237	0,053622	0,058365	0,050664	0,021782	0,046824	0,031845	0,041999	0,035028	0,039903	0,037124	0,038689
P7	0,05509	0,054387	0,049851	0,052368	0,023403	0,06019	0,042952	0,037831	0,040883	0,039455	0,050853	0,028309	0,035493	0,040557	0,051989	0,048804
P3	0,054463	0,065278	0,057999	0,058112	0,039645	0,054517	0,051782	0,032765	0,057455	0,034415	0,058433	0,022705	0,051137	0,033416	0,038022	0,054027
Pz	0,090127	0,070798	0,060445	0,079365	0,069263	0,060415	0,047019	0,052306	0,035242	0,063877	0,040555	0,04723	0,055897	0,043814	0,044548	0,043303
P4	0,064595	0,045204	0,037411	0,045834	0,064385	0,045556	0,039258	0,05999	0,031788	0,051436	0,034559	0,05756	0,048211	0,046086	0,044851	0,049638
P8	0,056525	0,037905	0,027954	0,036091	0,074601	0,042121	0,047129	0,055806	0,027199	0,049392	0,029896	0,052202	0,044824	0,053347	0,047648	0,05789
POz	0,073877	0,06232	0,059547	0,06661	0,053052	0,048207	0,046116	0,047576	0,033972	0,064446	0,033595	0,040278	0,050746	0,052978	0,035699	0,034242
O1	0,048356	0,031856	0,025509	0,032745	0,065427	0,044096	0,049513	0,060824	0,028251	0,047486	0,031555	0,046465	0,044598	0,044182	0,049644	0,052836
O2	0,079188	0,054758	0,054896	0,063466	0,060861	0,043765	0,043066	0,059545	0,036376	0,055989	0,033166	0,044058	0,053931	0,058417	0,045079	0,041072

Rows: subject 2 — Columns: subject 2 (T8–O2)

subject 2	T8	CP5	CP1	CP2	CP6	P7	P3	Pz	P4	P8	POz	O1	O2
Fp1	0,067886	0,070873	0,04396	0,066001	0,06303	0,05509	0,054463	0,090127	0,064595	0,056525	0,073877	0,048356	0,079188
Fpz	0,057379	0,061704	0,042696	0,048279	0,045435	0,054387	0,065278	0,070798	0,045204	0,037905	0,06232	0,031856	0,054758
Fp2	0,051037	0,058033	0,034546	0,042866	0,040945	0,049851	0,057999	0,060445	0,037411	0,027954	0,059547	0,025509	0,054896
F7	0,04465	0,066977	0,031626	0,053925	0,042308	0,052368	0,058112	0,079365	0,045834	0,036091	0,06661	0,032745	0,063466
F3	0,083204	0,025771	0,073629	0,05665	0,09237	0,023403	0,039645	0,069263	0,064385	0,074601	0,053052	0,065427	0,060861
Fz	0,064412	0,032467	0,0411	0,042263	0,053622	0,06019	0,054517	0,060415	0,045556	0,042121	0,048207	0,044096	0,043765
F4	0,05065	0,031212	0,046502	0,04253	0,058365	0,042952	0,051782	0,047019	0,039258	0,047129	0,046116	0,049513	0,043066
F8	0,022818	0,043701	0,053576	0,060453	0,050664	0,037831	0,032765	0,052306	0,05999	0,055806	0,047576	0,060824	0,059545
FC5	0,048426	0,03513	0,03411	0,031933	0,021782	0,040883	0,057455	0,035242	0,031788	0,027199	0,033972	0,028251	0,036376
FC1	0,046045	0,029525	0,064662	0,050011	0,046824	0,039455	0,034415	0,063877	0,051436	0,049392	0,064446	0,047486	0,055989
FC2	0,039439	0,037375	0,043987	0,010074	0,031845	0,050853	0,058433	0,040555	0,034559	0,029896	0,033595	0,031555	0,033166
FC6	0,033753	0,037498	0,047927	0,057111	0,041999	0,028309	0,022705	0,04723	0,05756	0,052202	0,040278	0,046465	0,044058
T7	0,043822	0,038591	0,039742	0,048654	0,035028	0,035493	0,051137	0,055897	0,048211	0,044824	0,050746	0,044598	0,053931
C3	0,058299	0,018653	0,04957	0,03831	0,039903	0,040557	0,033416	0,043814	0,046086	0,053347	0,052978	0,044182	0,058417
Cz	0,052696	0,0452	0,05552	0,031396	0,037124	0,051989	0,038022	0,044548	0,044851	0,047648	0,035699	0,049644	0,045079
C4	0,063759	0,039943	0,063408	0,040155	0,038689	0,048804	0,054027	0,043303	0,049638	0,05789	0,034242	0,052836	0,041072
T8	0	0,04646	0,042642	0,047437	0,041642	0,038022	0,034545	0,068707	0,049536	0,039793	0,055125	0,041647	0,053508
CP5	0,04646	0	0,03348	0,050796	0,039806	0,024089	0,014619	0,025459	0,037589	0,032116	0,020643	0,029833	0,026694
CP1	0,042642	0,03348	0	0,043115	0,043623	0,034023	0,042655	0,042921	0,031206	0,040223	0,05109	0,029287	0,04454
CP2	0,047437	0,050796	0,043115	0	0,017607	0,05385	0,045494	0,034186	0,012325	0,010056	0,033315	0,019674	0,026519
CP6	0,041642	0,039806	0,043623	0,017607	0	0,042251	0,039895	0,040279	0,016004	0,012143	0,039008	0,01476	0,032011
P7	0,038022	0,024089	0,034023	0,05385	0,042251	0	0,037264	0,046309	0,03547	0,032154	0,048836	0,0261	0,051423
P3	0,034545	0,014619	0,042655	0,045494	0,039895	0,037264	0	0,036493	0,020105	0,021402	0,029172	0,018429	0,042427
Pz	0,068707	0,025459	0,042921	0,034186	0,040279	0,046309	0,036493	0	0,030985	0,036164	0,031451	0,032023	0,043676
P4	0,049536	0,037589	0,031206	0,012325	0,016004	0,03547	0,020105	0,030985	0	0,011701	0,042062	0,013934	0,031442
P8	0,039793	0,032116	0,040223	0,010056	0,012143	0,032154	0,021402	0,036164	0,011701	0	0,036561	0,007773	0,031869
POz	0,055125	0,020643	0,05109	0,033315	0,039008	0,048836	0,029172	0,031451	0,042062	0,036561	0	0,037966	0,018882
O1	0,041647	0,029833	0,029287	0,019674	0,01476	0,0261	0,018429	0,032023	0,013934	0,007773	0,037966	0	0,030927
O2	0,053508	0,026694	0,04454	0,026519	0,032011	0,051423	0,042427	0,043676	0,031442	0,031869	0,018882	0,030927	0
