# Supplementary material for: Hyperbrain features of team mental models within a juggling paradigm: a proof of concept
Source: PeerJ. 2016 Sep 20;4:e2457. doi: 10.7717/peerj.2457 (PMC5036110; doi:10.7717/peerj.2457)
Supplement: Supplemental Information 3 [file peerj-04-2457-s003.pdf]

subject 1

		Fp1	Fpz	Fp2	F7	F3	Fz	F4	F8	FC5	FC1	FC2	FC6	T7	C3	Cz	C4	T8	CP5	CP1	CP2	CP6	P7	P3	Pz	P4	P8	POz	O1	O2
subject 1	Fp1	0	0,013108	0,010606	0,026213	0,031461	0,030386	0,036047	0,016691	0,025165	0,033274	0,044096	0,039107	0,035295	0,024422	0,042774	0,043602	0,025555	0,031865	0,025698	0,02729	0,019548	0,027197	0,024875	0,038414	0,025325	0,02066	0,0409	0,038853	0,040263
	Fpz	0,013108	0	0,018714	0,030476	0,033101	0,026242	0,032471	0,023902	0,025495	0,038054	0,039391	0,041137	0,041273	0,02714	0,039617	0,05028	0,024753	0,027043	0,019148	0,034811	0,01904	0,027082	0,029296	0,032885	0,021125	0,021886	0,038452	0,0344	0,037449
	Fp2	0,010606	0,018714	0	0,030097	0,019582	0,02926	0,032483	0,020911	0,023194	0,031745	0,04117	0,044524	0,034256	0,026464	0,04256	0,045433	0,028795	0,043791	0,026952	0,031917	0,023235	0,023776	0,032432	0,039481	0,027961	0,0233	0,040799	0,037473	0,036361
	F7	0,026213	0,030476	0,030097	0	0,034723	0,019675	0,022765	0,024082	0,027111	0,023045	0,014637	0,035398	0,055593	0,027741	0,02704	0,040167	0,022437	0,040457	0,018066	0,034726	0,028766	0,035243	0,030758	0,015152	0,02839	0,017732	0,038671	0,032013	0,037231
	F3	0,031461	0,033101	0,019582	0,034723	0	0,024317	0,02523	0,036716	0,030054	0,030794	0,041843	0,044747	0,033946	0,031322	0,040396	0,04798	0,031825	0,042339	0,02491	0,038431	0,024168	0,015001	0,036555	0,029845	0,021619	0,02137	0,039658	0,035785	0,032984
	Fz	0,030386	0,026242	0,02926	0,019675	0,024317	0	0,033924	0,036442	0,026163	0,039763	0,018417	0,027701	0,03733	0,025573	0,029221	0,041869	0,022978	0,036284	0,015645	0,03462	0,024035	0,032562	0,026137	0,031972	0,019519	0,017698	0,035674	0,043072	0,040193
	F4	0,036047	0,032471	0,032483	0,022765	0,02523	0,033924	0	0,03742	0,019692	0,027962	0,02197	0,025296	0,023912	0,026838	0,022563	0,03149	0,015114	0,033285	0,013751	0,025459	0,015908	0,03142	0,018747	0,026448	0,01759	0,026457	0,020866	0,029707	0,02873
	F8	0,016691	0,023902	0,020911	0,024082	0,036716	0,036442	0,03742	0	0,013425	0,024707	0,017688	0,02734	0,017658	0,018493	0,028296	0,022198	0,040418	0,023023	0,032708	0,044788	0,035515	0,029376	0,026406	0,026897	0,035036	0,039275	0,028322	0,033849	0,031156
	FC5	0,025165	0,025495	0,023194	0,027111	0,030054	0,026163	0,019692	0,013425	0	0,030079	0,029505	0,029878	0,037436	0,010121	0,033303	0,034741	0,03832	0,029819	0,012429	0,026888	0,021388	0,027448	0,01929	0,025741	0,028839	0,022878	0,032435	0,01878	0,02994
	FC1	0,033274	0,038054	0,031745	0,023045	0,030794	0,039763	0,027962	0,024707	0,030079	0	0,023601	0,016216	0,020337	0,031106	0,018593	0,036612	0,021847	0,03913	0,020001	0,014867	0,015273	0,028992	0,021865	0,03629	0,027602	0,012625	0,021028	0,025422	0,028726
	FC2	0,044096	0,039391	0,04117	0,014637	0,041843	0,018417	0,02197	0,017688	0,029505	0,023601	0	0,024622	0,051089	0,031089	0,019213	0,027226	0,019822	0,039397	0,022101	0,038653	0,025915	0,030641	0,035508	0,029711	0,030644	0,019029	0,035774	0,033331	0,034611
	FC6	0,039107	0,041137	0,044524	0,035398	0,044747	0,027701	0,025296	0,02734	0,029878	0,016216	0,024622	0	0,036274	0,036739	0,016891	0,049135	0,025813	0,037037	0,021671	0,035431	0,024955	0,033237	0,0261	0,018917	0,029362	0,013087	0,032106	0,020402	0,033797
	T7	0,035295	0,041273	0,034256	0,055593	0,033946	0,03733	0,023912	0,017658	0,037436	0,020337	0,051089	0,036274	0	0,052913	0,039759	0,04788	0,047207	0,034782	0,013537	0,033893	0,022761	0,030577	0,018823	0,019911	0,02518	0,023902	0,034119	0,017692	0,034313
	C3	0,024422	0,02714	0,026464	0,027741	0,031322	0,025573	0,026838	0,018493	0,010121	0,031106	0,031089	0,036739	0,052913	0	0,042437	0,043963	0,034056	0,034331	0,012695	0,030835	0,024997	0,037114	0,025442	0,016253	0,022185	0,02395	0,03265	0,024571	0,035012
	Cz	0,042774	0,039617	0,04256	0,02704	0,040396	0,029221	0,022563	0,028296	0,033303	0,018593	0,019213	0,016891	0,039759	0,042437	0	0,040757	0,01821	0,037352	0,022669	0,039985	0,025152	0,03711	0,032534	0,018574	0,025862	0,016325	0,036115	0,027528	0,031846
	C4	0,043602	0,05028	0,045433	0,040167	0,04798	0,041869	0,03149	0,022198	0,034741	0,036612	0,027226	0,049135	0,04788	0,043963	0,040757	0	0,032412	0,052906	0,021593	0,041839	0,034957	0,030145	0,044503	0,029813	0,030715	0,026986	0,04517	0,042973	0,039195
	T8	0,025555	0,024753	0,028795	0,022437	0,031825	0,022978	0,015114	0,040418	0,03832	0,021847	0,019822	0,025813	0,047207	0,034056	0,01821	0,032412	0	0,026691	0,022076	0,042052	0,037985	0,017663	0,033249	0,020955	0,02138	0,014543	0,030485	0,017711	0,036426
	CP5	0,031865	0,027043	0,043791	0,040457	0,042339	0,036284	0,033285	0,023023	0,029819	0,03913	0,039397	0,037037	0,034782	0,034331	0,037352	0,052906	0,026691	0	0,018955	0,034369	0,023043	0,02721	0,024547	0,03523	0,018048	0,020348	0,030913	0,034064	0,036907
	CP1	0,025698	0,019148	0,026952	0,018066	0,02491	0,015645	0,013751	0,032708	0,012429	0,020001	0,022101	0,021671	0,013537	0,012695	0,022669	0,021593	0,022076	0,018955	0	0,013358	0,029749	0,015624	0,02061	0,01169	0,012461	0,028331	0,019664	0,026873	0,027339
	CP2	0,02729	0,034811	0,031917	0,034726	0,038431	0,03462	0,025459	0,044788	0,026888	0,014867	0,038653	0,035431	0,033893	0,030835	0,039985	0,041839	0,042052	0,034369	0,013358	0	0,021744	0,021502	0,033279	0,026011	0,019099	0,019494	0,019941	0,027705	0,029822
	CP6	0,019548	0,01904	0,023235	0,028766	0,024168	0,024035	0,015908	0,035515	0,021388	0,015273	0,025915	0,024955	0,022761	0,024997	0,025152	0,034957	0,037985	0,023043	0,029749	0,021744	0	0,021798	0,025575	0,015675	0,037249	0,019205	0,026664	0,026125	0,029148
	P7	0,027197	0,027082	0,023776	0,035243	0,015001	0,032562	0,03142	0,029376	0,027448	0,028992	0,030641	0,033237	0,030577	0,037114	0,03711	0,030145	0,017663	0,02721	0,015624	0,021502	0,021798	0	0,031631	0,026882	0,016401	0,020675	0,028923	0,015405	0,019162
	P3	0,024875	0,029296	0,032432	0,030758	0,036555	0,026137	0,018747	0,026406	0,01929	0,021865	0,035508	0,0261	0,018823	0,025442	0,032534	0,044503	0,033249	0,024547	0,02061	0,033279	0,025575	0,031631	0	0,027844	0,023741	0,01803	0,030656	0,030485	0,035792
	Pz	0,038414	0,032885	0,039481	0,015152	0,029845	0,031972	0,026448	0,026897	0,025741	0,03629	0,029711	0,018917	0,019911	0,016253	0,018574	0,029813	0,020955	0,03523	0,01169	0,026011	0,015675	0,026882	0,027844	0	0,022489	0,026277	0,028992	0,028836	0,031484
	P4	0,025325	0,021125	0,027961	0,02839	0,021619	0,019519	0,01759	0,035036	0,028839	0,027602	0,030644	0,029362	0,02518	0,022185	0,025862	0,030715	0,02138	0,018048	0,012461	0,019099	0,037249	0,016401	0,023741	0,022489	0	0,018679	0,022077	0,018088	0,032148
	P8	0,02066	0,021886	0,0233	0,017732	0,02137	0,017698	0,026457	0,039275	0,022878	0,012625	0,019029	0,013087	0,023902	0,02395	0,016325	0,026986	0,014543	0,020348	0,028331	0,019494	0,019205	0,020675	0,01803	0,026277	0,018679	0	0,024689	0,016902	0,013406
	POz	0,0409	0,038452	0,040799	0,038671	0,039658	0,035674	0,020866	0,028322	0,032435	0,021028	0,035774	0,032106	0,034119	0,03265	0,036115	0,04517	0,030485	0,030913	0,019664	0,019941	0,026664	0,028923	0,030656	0,028992	0,022077	0,024689	0	0,031986	0,027941
	O1	0,038853	0,0344	0,037473	0,032013	0,035785	0,043072	0,029707	0,033849	0,01878	0,025422	0,033331	0,020402	0,017692	0,024571	0,027528	0,042973	0,017711	0,034064	0,026873	0,027705	0,026125	0,015405	0,030485	0,028836	0,018088	0,016902	0,031986	0	0,03302
	O2	0,040263	0,037449	0,036361	0,037231	0,032984	0,040193	0,02873	0,031156	0,02994	0,028726	0,034611	0,033797	0,034313	0,035012	0,031846	0,039195	0,036426	0,036907	0,027339	0,029822	0,029148	0,019162	0,035792	0,031484	0,032148	0,013406	0,027941	0,03302	0
subject 2	Fp1	0,029919	0,025005	0,030319	0,024775	0,026127	0,031428	0,022423	0,017518	0,022716	0,029954	0,029928	0,028279	0,024953	0,018572	0,026909	0,03002	0,018377	0,027655	0,01512	0,028226	0,018293	0,015539	0,024209	0,02398	0,025854	0,017483	0,027448	0,027746	0,025222
	Fpz	0,029363	0,029437	0,030248	0,024806	0,029549	0,032019	0,025478	0,020052	0,020725	0,023662	0,03152	0,026689	0,027995	0,019288	0,027286	0,034526	0,020937	0,02784	0,017571	0,032791	0,021558	0,010864	0,029562	0,024469	0,020227	0,024214	0,030064	0,02889	0,028144
	Fp2	0,027579	0,0292	0,030916	0,029917	0,028564	0,03852	0,02801	0,025501	0,025423	0,025723	0,035363	0,033954	0,031039	0,021877	0,029631	0,035582	0,020677	0,028633	0,011875	0,032373	0,024058	0,011245	0,026954	0,023136	0,019749	0,017383	0,031933	0,030291	0,027553
	F7	0,029843	0,02356	0,024114	0,025036	0,022265	0,032364	0,027325	0,022748	0,023134	0,025133	0,030449	0,026199	0,022722	0,016757	0,0255	0,033127	0,028362	0,01982	0,02593	0,032366	0,025392	0,014027	0,026562	0,023372	0,027941	0,022931	0,029259	0,025945	0,026691
	F3	0,033855	0,033408	0,035923	0,026809	0,031263	0,039948	0,017105	0,026757	0,019803	0,025124	0,038698	0,029102	0,025071	0,016473	0,034214	0,042011	0,019887	0,024272	0,02039	0,030197	0,018885	0,018553	0,026965	0,022953	0,015392	0,017154	0,038112	0,022311	0,030252
	Fz	0,030246	0,031868	0,028762	0,030893	0,028385	0,036346	0,022868	0,029287	0,026228	0,017297	0,037321	0,033075	0,027506	0,021238	0,036204	0,043742	0,023655	0,024738	0,022151	0,033998	0,027241	0,020954	0,027116	0,025438	0,02581	0,015115	0,033446	0,023808	0,026733
	F4	0,030278	0,034147	0,034717	0,029178	0,029443	0,042227	0,022063	0,020956	0,022034	0,020612	0,033844	0,03003	0,022987	0,02094	0,033141	0,0388	0,019881	0,026794	0,025291	0,028475	0,019622	0,014446	0,029808	0,023999	0,022806	0,019654	0,034792	0,028923	0,027456
	F8	0,024109	0,025869	0,027156	0,026906	0,026002	0,03724	0,026551	0,021319	0,023291	0,021166	0,039076	0,029378	0,024949	0,017415	0,030683	0,039697	0,022585	0,023177	0,02312	0,038657	0,024386	0,012748	0,02624	0,024286	0,02639	0,02052	0,036326	0,026443	0,027358
	FC5	0,026261	0,028374	0,027503	0,027431	0,029577	0,034362	0,026866	0,021116	0,029122	0,023889	0,037564	0,024331	0,022119	0,021105	0,03002	0,032391	0,020768	0,025514	0,023983	0,032645	0,025472	0,014267	0,025315	0,021533	0,024719	0,018273	0,032483	0,026975	0,029366
	FC1	0,029183	0,032498	0,034126	0,026115	0,034365	0,042061	0,026998	0,021072	0,025355	0,020073	0,03649	0,028461	0,026008	0,018488	0,028916	0,030605	0,024201	0,021389	0,02827	0,029645	0,020498	0,014855	0,025531	0,025179	0,028369	0,021743	0,028851	0,024624	0,026784
	FC2	0,027651	0,031327	0,028161	0,022983	0,025949	0,035851	0,024421	0,01813	0,021565	0,019717	0,031632	0,024211	0,022543	0,015454	0,028246	0,033451	0,021163	0,026416	0,02497	0,027948	0,019425	0,015136	0,026338	0,025651	0,025126	0,018207	0,029217	0,024181	0,025723
	FC6	0,028737	0,0274	0,026199	0,021548	0,026385	0,036893	0,027463	0,023924	0,024237	0,022348	0,036227	0,028738	0,016753	0,016806	0,031767	0,038936	0,020188	0,017995	0,022524	0,035233	0,023315	0,016205	0,023096	0,02361	0,020634	0,019675	0,038694	0,027284	0,025146
	T7	0,028136	0,029479	0,027001	0,026211	0,026633	0,035174	0,027663	0,022017	0,028184	0,022465	0,030971	0,026244	0,023306	0,025217	0,027145	0,036787	0,020538	0,025066	0,021066	0,029179	0,026877	0,01304	0,030546	0,023498	0,023999	0,015091	0,028966	0,030163	0,03139
	C3	0,027869	0,031274	0,028598	0,02729	0,030475	0,035016	0,022722	0,022876	0,022948	0,024023	0,035201	0,024899	0,025542	0,021231	0,027221	0,037196	0,02155	0,018541	0,022064	0,03034	0,0263	0,009824	0,032426	0,022713	0,025857	0,019669	0,030842	0,024806	0,030235
	Cz	0,030329	0,029537	0,030185	0,024726	0,033493	0,035618	0,023199	0,015731	0,024205	0,018569	0,031944	0,020564	0,023027	0,01825	0,022829	0,032996	0,017585	0,025628	0,020782	0,025317	0,019103	0,018081	0,022554	0,026404	0,022828	0,020567	0,031073	0,024181	0,034952
	C4	0,029816	0,029108	0,028034	0,02327	0,021381	0,038569	0,026568	0,013402	0,016569	0,025774	0,027015	0,021184	0,016376	0,017608	0,024893	0,023689	0,022314	0,025523	0,017568	0,023723	0,014994	0,018575	0,022233	0,029995	0,026699	0,01829	0,02296	0,021941	0,021134
	T8	0,019687	0,022281	0,024973	0,023077	0,023912	0,03484	0,030458	0,019307	0,027429	0,020402	0,034677	0,027154	0,022544	0,024965	0,029323	0,032655	0,020254	0,019522	0,016689	0,033365	0,028854	0,016435	0,023204	0,023492	0,018285	0,021094	0,03202	0,019791	0,022556
	CP5	0,020056	0,022212	0,018562	0,026227	0,023233	0,021625	0,020362	0,025219	0,021055	0,015873	0,027079	0,021645	0,018919	0,019602	0,022622	0,026306	0,020426	0,015709	0,021325	0,026106	0,022503	0,016308	0,025652	0,021449	0,019415	0,022795	0,023821	0,027964	0,02362
	CP1	0,027579	0,028761	0,027653	0,018473	0,022898	0,031564	0,022499	0,015822	0,011744	0,02519	0,026269	0,016805	0,021976	0,017538	0,017755	0,024657	0,020162	0,025328	0,023892	0,025544	0,019638	0,016802	0,025547	0,027655	0,024833	0,016385	0,026303	0,025439	0,019907
	CP2	0,027884	0,027188	0,027763	0,021678	0,029503	0,038149	0,026463	0,014159	0,0241	0,019279	0,02886	0,023598	0,015441	0,015136	0,026446	0,032715	0,021487	0,022526	0,022522	0,02336	0,015959	0,019135	0,024298	0,020617	0,026917	0,01976	0,029647	0,021185	0,028742
	CP6	0,027342	0,030105	0,029166	0,020313	0,028782	0,035891	0,026328	0,012756	0,016014	0,023877	0,030185	0,022005	0,014293	0,017671	0,022971	0,029523	0,016637	0,026323	0,015722	0,027335	0,01722	0,019975	0,02307	0,025838	0,02083	0,020523	0,031912	0,026025	0,024574
	P7	0,02131	0,018148	0,017139	0,019911	0,016335	0,013744	0,024161	0,024784	0,020565	0,010564	0,020359	0,020488	0,015169	0,023995	0,020273	0,020794	0,025855	0,012817	0,016875	0,023097	0,022439	0,016475	0,020071	0,020629	0,025528	0,019193	0,020533	0,016928	0,015568
	P3	0,011934	0,012466	0,009432	0,020065	0,011717	0,011075	0,018617	0,020217	0,020772	0,011175	0,017926	0,020906	0,013202	0,018393	0,020301	0,018762	0,019904	0,010477	0,020657	0,022862	0,017747	0,019941	0,016169	0,017941	0,020108	0,020594	0,015738	0,023637	0,014376
	Pz	0,026703	0,030362	0,029985	0,025382	0,028968	0,035497	0,022972	0,020071	0,027778	0,019458	0,030216	0,023304	0,026748	0,021597	0,021165	0,030409	0,022918	0,020945	0,021163	0,030264	0,024291	0,015475	0,027825	0,022768	0,023573	0,021458	0,028658	0,026252	0,035224
	P4	0,030735	0,028149	0,031812	0,018936	0,029932	0,037989	0,027471	0,01523	0,022456	0,022623	0,032268	0,017106	0,018188	0,014378	0,024556	0,031001	0,024458	0,025916	0,021741	0,026793	0,015214	0,019679	0,023118	0,026402	0,024546	0,020559	0,032631	0,023289	0,030699
	P8	0,027543	0,029186	0,027522	0,021174	0,029539	0,035795	0,026389	0,020087	0,023111	0,021028	0,032625	0,021854	0,018916	0,014753	0,026502	0,035013	0,024276	0,026329	0,024939	0,027666	0,016074	0,022517	0,026842	0,023998	0,023046	0,018175	0,032182	0,021614	0,030184
	POz	0,025994	0,024758	0,02552	0,029166	0,025857	0,032282	0,021486	0,021834	0,022376	0,021562	0,029938	0,023188	0,023847	0,023063	0,024307	0,034724	0,023311	0,018853	0,024411	0,029102	0,025619	0,012444	0,023399	0,026019	0,022458	0,020423	0,029692	0,025475	0,029572
	O1	0,030695	0,033886	0,032169	0,020511	0,03236	0,038694	0,026027	0,022134	0,024053	0,022721	0,036478	0,023856	0,02024	0,014765	0,02992	0,037533	0,022385	0,031268	0,02142	0,029909	0,01963	0,019252	0,026629	0,0273	0,02336	0,019267	0,034554	0,027524	0,031768
	O2	0,027823	0,026634	0,02854	0,027986	0,024024	0,032095	0,023839	0,02278	0,026652	0,025751	0,034342	0,024142	0,022293	0,023659	0,02608	0,035504	0,022917	0,019364	0,020425	0,029505	0,02704	0,014066	0,023017	0,028104	0,021743	0,019989	0,032029	0,027002	0,030545

subject 2

		Fp1	Fpz	Fp2	F7	F3	Fz	F4	F8	FC5	FC1	FC2	FC6	T7	C3	Cz	C4	T8	CP5	CP1	CP2	CP6	P7	P3	Pz	P4	P8	POz	O1	O2
subject 1	Fp1	0,029919	0,029363	0,027579	0,029843	0,033855	0,030246	0,030278	0,024109	0,026261	0,029183	0,027651	0,028737	0,028136	0,027869	0,030329	0,029816	0,019687	0,020056	0,027579	0,027884	0,027342	0,02131	0,011934	0,026703	0,030735	0,027543	0,025994	0,030695	0,027823
	Fpz	0,025005	0,029437	0,0292	0,02356	0,033408	0,031868	0,034147	0,025869	0,028374	0,032498	0,031327	0,0274	0,029479	0,031274	0,029537	0,029108	0,022281	0,022212	0,028761	0,027188	0,030105	0,018148	0,012466	0,030362	0,028149	0,029186	0,024758	0,033886	0,026634
	Fp2	0,030319	0,030248	0,030916	0,024114	0,035923	0,028762	0,034717	0,027156	0,027503	0,034126	0,028161	0,026199	0,027001	0,028598	0,030185	0,028034	0,024973	0,018562	0,027653	0,027763	0,029166	0,017139	0,009432	0,029985	0,031812	0,027522	0,02552	0,032169	0,02854
	F7	0,024775	0,024806	0,029917	0,025036	0,026809	0,030893	0,029178	0,026906	0,027431	0,026115	0,022983	0,021548	0,026211	0,02729	0,024726	0,02327	0,023077	0,026227	0,018473	0,021678	0,020313	0,019911	0,020065	0,025382	0,018936	0,021174	0,029166	0,020511	0,027986
	F3	0,026127	0,029549	0,028564	0,022265	0,031263	0,028385	0,029443	0,026002	0,029577	0,034365	0,025949	0,026385	0,026633	0,030475	0,033493	0,021381	0,023912	0,023233	0,022898	0,029503	0,028782	0,016335	0,011717	0,028968	0,029932	0,029539	0,025857	0,03236	0,024024
	Fz	0,031428	0,032019	0,03852	0,032364	0,039948	0,036346	0,042227	0,03724	0,034362	0,042061	0,035851	0,036893	0,035174	0,035016	0,035618	0,038569	0,03484	0,021625	0,031564	0,038149	0,035891	0,013744	0,011075	0,035497	0,037989	0,035795	0,032282	0,038694	0,032095
	F4	0,022423	0,025478	0,02801	0,027325	0,017105	0,022868	0,022063	0,026551	0,026866	0,026998	0,024421	0,027463	0,027663	0,022722	0,023199	0,026568	0,030458	0,020362	0,022499	0,026463	0,026328	0,024161	0,018617	0,022972	0,027471	0,026389	0,021486	0,026027	0,023839
	F8	0,017518	0,020052	0,025501	0,022748	0,026757	0,029287	0,020956	0,021319	0,021116	0,021072	0,01813	0,023924	0,022017	0,022876	0,015731	0,013402	0,019307	0,025219	0,015822	0,014159	0,012756	0,024784	0,020217	0,020071	0,01523	0,020087	0,021834	0,022134	0,02278
	FC5	0,022716	0,020725	0,025423	0,023134	0,019803	0,026228	0,022034	0,023291	0,029122	0,025355	0,021565	0,024237	0,028184	0,022948	0,024205	0,016569	0,027429	0,021055	0,011744	0,0241	0,016014	0,020565	0,020772	0,027778	0,022456	0,023111	0,022376	0,024053	0,026652
	FC1	0,029954	0,023662	0,025723	0,025133	0,025124	0,017297	0,020612	0,021166	0,023889	0,020073	0,019717	0,022348	0,022465	0,024023	0,018569	0,025774	0,020402	0,015873	0,02519	0,019279	0,023877	0,010564	0,011175	0,019458	0,022623	0,021028	0,021562	0,022721	0,025751
	FC2	0,029928	0,03152	0,035363	0,030449	0,038698	0,037321	0,033844	0,039076	0,037564	0,03649	0,031632	0,036227	0,030971	0,035201	0,031944	0,027015	0,034677	0,027079	0,026269	0,02886	0,030185	0,020359	0,017926	0,030216	0,032268	0,032625	0,029938	0,036478	0,034342
	FC6	0,028279	0,026689	0,033954	0,026199	0,029102	0,033075	0,03003	0,029378	0,024331	0,028461	0,024211	0,028738	0,026244	0,024899	0,020564	0,021184	0,027154	0,021645	0,016805	0,023598	0,022005	0,020488	0,020906	0,023304	0,017106	0,021854	0,023188	0,023856	0,024142
	T7	0,024953	0,027995	0,031039	0,022722	0,025071	0,027506	0,022987	0,024949	0,022119	0,026008	0,022543	0,016753	0,023306	0,025542	0,023027	0,016376	0,022544	0,018919	0,021976	0,015441	0,014293	0,015169	0,013202	0,026748	0,018188	0,018916	0,023847	0,02024	0,022293
	C3	0,018572	0,019288	0,021877	0,016757	0,016473	0,021238	0,02094	0,017415	0,021105	0,018488	0,015454	0,016806	0,025217	0,021231	0,01825	0,017608	0,024965	0,019602	0,017538	0,015136	0,017671	0,023995	0,018393	0,021597	0,014378	0,014753	0,023063	0,014765	0,023659
	Cz	0,026909	0,027286	0,029631	0,0255	0,034214	0,036204	0,033141	0,030683	0,03002	0,028916	0,028246	0,031767	0,027145	0,027221	0,022829	0,024893	0,029323	0,022622	0,017755	0,026446	0,022971	0,020273	0,020301	0,021165	0,024556	0,026502	0,024307	0,02992	0,02608
	C4	0,03002	0,034526	0,035582	0,033127	0,042011	0,043742	0,0388	0,039697	0,032391	0,030605	0,033451	0,038936	0,036787	0,037196	0,032996	0,023689	0,032655	0,026306	0,024657	0,032715	0,029523	0,020794	0,018762	0,030409	0,031001	0,035013	0,034724	0,037533	0,035504
	T8	0,018377	0,020937	0,020677	0,028362	0,019887	0,023655	0,019881	0,022585	0,020768	0,024201	0,021163	0,020188	0,020538	0,02155	0,017585	0,022314	0,020254	0,020426	0,020162	0,021487	0,016637	0,025855	0,019904	0,022918	0,024458	0,024276	0,023311	0,022385	0,022917
	CP5	0,027655	0,02784	0,028633	0,01982	0,024272	0,024738	0,026794	0,023177	0,025514	0,021389	0,026416	0,017995	0,025066	0,018541	0,025628	0,025523	0,019522	0,015709	0,025328	0,022526	0,026323	0,012817	0,010477	0,020945	0,025916	0,026329	0,018853	0,031268	0,019364
	CP1	0,01512	0,017571	0,011875	0,02593	0,02039	0,022151	0,025291	0,02312	0,023983	0,02827	0,02497	0,022524	0,021066	0,022064	0,020782	0,017568	0,016689	0,021325	0,023892	0,022522	0,015722	0,016875	0,020657	0,021163	0,021741	0,024939	0,024411	0,02142	0,020425
	CP2	0,028226	0,032791	0,032373	0,032366	0,030197	0,033998	0,028475	0,038657	0,032645	0,029645	0,027948	0,035233	0,029179	0,03034	0,025317	0,023723	0,033365	0,026106	0,025544	0,02336	0,027335	0,023097	0,022862	0,030264	0,026793	0,027666	0,029102	0,029909	0,029505
	CP6	0,018293	0,021558	0,024058	0,025392	0,018885	0,027241	0,019622	0,024386	0,025472	0,020498	0,019425	0,023315	0,026877	0,0263	0,019103	0,014994	0,028854	0,022503	0,019638	0,015959	0,01722	0,022439	0,017747	0,024291	0,015214	0,016074	0,025619	0,01963	0,02704
	P7	0,015539	0,010864	0,011245	0,014027	0,018553	0,020954	0,014446	0,012748	0,014267	0,014855	0,015136	0,016205	0,01304	0,009824	0,018081	0,018575	0,016435	0,016308	0,016802	0,019135	0,019975	0,016475	0,019941	0,015475	0,019679	0,022517	0,012444	0,019252	0,014066
	P3	0,024209	0,029562	0,026954	0,026562	0,026965	0,027116	0,029808	0,02624	0,025315	0,025531	0,026338	0,023096	0,030546	0,032426	0,022554	0,022233	0,023204	0,025652	0,025547	0,024298	0,02307	0,020071	0,016169	0,027825	0,023118	0,026842	0,023399	0,026629	0,023017
	Pz	0,02398	0,024469	0,023136	0,023372	0,022953	0,025438	0,023999	0,024286	0,021533	0,025179	0,025651	0,02361	0,023498	0,022713	0,026404	0,029995	0,023492	0,021449	0,027655	0,020617	0,025838	0,020629	0,017941	0,022768	0,026402	0,023998	0,026019	0,0273	0,028104
	P4	0,025854	0,020227	0,019749	0,027941	0,015392	0,02581	0,022806	0,02639	0,024719	0,028369	0,025126	0,020634	0,023999	0,025857	0,022828	0,026699	0,018285	0,019415	0,024833	0,026917	0,02083	0,025528	0,020108	0,023573	0,024546	0,023046	0,022458	0,02336	0,021743
	P8	0,017483	0,024214	0,017383	0,022931	0,017154	0,015115	0,019654	0,02052	0,018273	0,021743	0,018207	0,019675	0,015091	0,019669	0,020567	0,01829	0,021094	0,022795	0,016385	0,01976	0,020523	0,019193	0,020594	0,021458	0,020559	0,018175	0,020423	0,019267	0,019989
	POz	0,027448	0,030064	0,031933	0,029259	0,038112	0,033446	0,034792	0,036326	0,032483	0,028851	0,029217	0,038694	0,028966	0,030842	0,031073	0,02296	0,03202	0,023821	0,026303	0,029647	0,031912	0,020533	0,015738	0,028658	0,032631	0,032182	0,029692	0,034554	0,032029
	O1	0,027746	0,02889	0,030291	0,025945	0,022311	0,023808	0,028923	0,026443	0,026975	0,024624	0,024181	0,027284	0,030163	0,024806	0,024181	0,021941	0,019791	0,027964	0,025439	0,021185	0,026025	0,016928	0,023637	0,026252	0,023289	0,021614	0,025475	0,027524	0,027002
	O2	0,025222	0,028144	0,027553	0,026691	0,030252	0,026733	0,027456	0,027358	0,029366	0,026784	0,025723	0,025146	0,03139	0,030235	0,034952	0,021134	0,022556	0,02362	0,019907	0,028742	0,024574	0,015568	0,014376	0,035224	0,030699	0,030184	0,029572	0,031768	0,030545
subject 2	Fp1	0	0,027978	0,028895	0,030232	0,031988	0,040936	0,043002	0,030247	0,028842	0,040375	0,045828	0,033622	0,03009	0,024768	0,035706	0,044462	0,031926	0,020443	0,02448	0,028399	0,032531	0,025185	0,019657	0,029002	0,027897	0,024627	0,022506	0,025108	0,027104
	Fpz	0,027978	0	0,017675	0,010271	0,038638	0,044871	0,040792	0,023709	0,027772	0,040737	0,041208	0,027121	0,029832	0,026408	0,031756	0,039966	0,022384	0,026414	0,025355	0,029388	0,031241	0,025879	0,027231	0,02994	0,025377	0,028108	0,025701	0,033294	0,030432
	Fp2	0,028895	0,017675	0	0,01842	0,032926	0,049559	0,043792	0,019876	0,026253	0,044272	0,041478	0,025449	0,027872	0,039251	0,037351	0,038283	0,022526	0,026091	0,022815	0,027364	0,02806	0,025453	0,022335	0,038443	0,028067	0,033206	0,031063	0,034496	0,033645
	F7	0,030232	0,010271	0,01842	0	0,031355	0,031107	0,030469	0,02513	0,025649	0,034788	0,033599	0,027287	0,030361	0,031167	0,030743	0,037481	0,025636	0,024173	0,026852	0,027432	0,03331	0,025801	0,031919	0,027341	0,02007	0,020992	0,026166	0,02325	0,030908
	F3	0,031988	0,038638	0,032926	0,031355	0	0,028943	0,043543	0,033363	0,037006	0,039796	0,045491	0,035357	0,034901	0,040015	0,050213	0,045326	0,036217	0,031794	0,029361	0,043885	0,043138	0,034692	0,03124	0,040757	0,038796	0,03409	0,036552	0,036833	0,042723
	Fz	0,040936	0,044871	0,049559	0,031107	0,028943	0	0,01398	0,035737	0,019218	0,027725	0,020367	0,031111	0,018912	0,029143	0,038865	0,031153	0,043062	0,02005	0,029046	0,03331	0,02605	0,031237	0,026774	0,042339	0,036637	0,040854	0,030748	0,036035	0,032847
	F4	0,043002	0,040792	0,043792	0,030469	0,043543	0,01398	0	0,032736	0,029078	0,023544	0,014366	0,035455	0,028771	0,033146	0,045485	0,042677	0,047194	0,027395	0,028196	0,029574	0,028082	0,030875	0,028156	0,027729	0,038377	0,033897	0,028007	0,029175	0,034893
	F8	0,030247	0,023709	0,019876	0,02513	0,033363	0,035737	0,032736	0	0,022523	0,035433	0,039217	0,015302	0,0179	0,031928	0,039261	0,041207	0,022555	0,022148	0,027088	0,029549	0,037556	0,032453	0,026562	0,023999	0,027486	0,029534	0,027071	0,02842	0,034464
	FC5	0,028842	0,027772	0,026253	0,025649	0,037006	0,019218	0,029078	0,022523	0	0,023889	0,022635	0,026521	0,007099	0,030106	0,034348	0,038952	0,034553	0,025927	0,020085	0,020848	0,026677	0,033067	0,028179	0,031003	0,023441	0,019575	0,02827	0,018238	0,032862
	FC1	0,040375	0,040737	0,044272	0,034788	0,039796	0,027725	0,023544	0,035433	0,023889	0	0,01757	0,032157	0,025987	0,0474	0,051623	0,040346	0,045088	0,036809	0,024381	0,032105	0,026313	0,03683	0,031406	0,048941	0,045622	0,038986	0,041739	0,040612	0,046423
	FC2	0,045828	0,041208	0,041478	0,033599	0,045491	0,020367	0,014366	0,039217	0,022635	0,01757	0	0,035306	0,02612	0,023054	0,03253	0,036759	0,045898	0,022524	0,026326	0,015349	0,015808	0,031902	0,029953	0,02591	0,024891	0,023307	0,023429	0,024573	0,030045
	FC6	0,033622	0,027121	0,025449	0,027287	0,035357	0,031111	0,035455	0,015302	0,026521	0,032157	0,035306	0	0,024435	0,034009	0,034606	0,044332	0,030662	0,02595	0,041661	0,036864	0,044821	0,032966	0,02852	0,022586	0,031835	0,026772	0,029393	0,026143	0,027435
	T7	0,03009	0,029832	0,027872	0,030361	0,034901	0,018912	0,028771	0,0179	0,007099	0,025987	0,02612	0,024435	0	0,036737	0,033142	0,038756	0,026267	0,032798	0,025102	0,025692	0,028585	0,029382	0,023921	0,028716	0,023626	0,02123	0,034535	0,021117	0,037075
	C3	0,024768	0,026408	0,039251	0,031167	0,040015	0,029143	0,033146	0,031928	0,030106	0,0474	0,023054	0,034009	0,036737	0	0,050979	0,041631	0,031779	0,053236	0,023812	0,033186	0,026938	0,048141	0,044917	0,056031	0,037045	0,0384	0,04979	0,038736	0,056629
	Cz	0,035706	0,031756	0,037351	0,030743	0,050213	0,038865	0,045485	0,039261	0,034348	0,051623	0,03253	0,034606	0,033142	0,050979	0	0,038407	0,045857	0,037211	0,030881	0,055156	0,027446	0,032993	0,030024	0,042551	0,058738	0,049024	0,052933	0,048264	0,057931
	C4	0,044462	0,039966	0,038283	0,037481	0,045326	0,031153	0,042677	0,041207	0,038952	0,040346	0,036759	0,044332	0,038756	0,041631	0,038407	0	0,043728	0,032421	0,041164	0,036571	0,035484	0,02537	0,029317	0,034553	0,040166	0,042019	0,032842	0,042209	0,039827
	T8	0,031926	0,022384	0,022526	0,025636	0,036217	0,043062	0,047194	0,022555	0,034553	0,045088	0,045898	0,030662	0,026267	0,031779	0,045857	0,043728	0	0,015311	0,045557	0,04606	0,052435	0,034568	0,031646	0,028255	0,041031	0,042619	0,025398	0,044504	0,029887
	CP5	0,020443	0,026414	0,026091	0,024173	0,031794	0,02005	0,027395	0,022148	0,025927	0,036809	0,022524	0,02595	0,032798	0,053236	0,037211	0,032421	0,015311	0	0,022075	0,022968	0,023759	0,046097	0,044711	0,047543	0,029329	0,02885	0,054112	0,03063	0,05079
	CP1	0,02448	0,025355	0,022815	0,026852	0,029361	0,029046	0,028196	0,027088	0,020085	0,024381	0,026326	0,041661	0,025102	0,023812	0,030881	0,041164	0,045557	0,022075	0	0,026409	0,022109	0,029729	0,020651	0,034467	0,024232	0,024427	0,027112	0,028477	0,032174
	CP2	0,028399	0,029388	0,027364	0,027432	0,043885	0,03331	0,029574	0,029549	0,020848	0,032105	0,015349	0,036864	0,025692	0,033186	0,055156	0,036571	0,04606	0,022968	0,026409	0	0,015311	0,030492	0,024387	0,025359	0,03386	0,02912	0,028091	0,027626	0,034013
	CP6	0,032531	0,031241	0,02806	0,03331	0,043138	0,02605	0,028082	0,037556	0,026677	0,026313	0,015808	0,044821	0,028585	0,026938	0,027446	0,035484	0,052435	0,023759	0,022109	0,015311	0	0,03367	0,031523	0,024458	0,022961	0,020851	0,024972	0,021512	0,025198
	P7	0,025185	0,025879	0,025453	0,025801	0,034692	0,031237	0,030875	0,032453	0,033067	0,03683	0,031902	0,032966	0,029382	0,048141	0,032993	0,02537	0,034568	0,046097	0,029729	0,030492	0,03367	0	0,03629	0,050299	0,035856	0,035281	0,046508	0,035639	0,046579
	P3	0,019657	0,027231	0,022335	0,031919	0,03124	0,026774	0,028156	0,026562	0,028179	0,031406	0,029953	0,02852	0,023921	0,044917	0,030024	0,029317	0,031646	0,044711	0,020651	0,024387	0,031523	0,03629	0	0,042595	0,027896	0,029726	0,046782	0,027938	0,043949
	Pz	0,029002	0,02994	0,038443	0,027341	0,040757	0,042339	0,027729	0,023999	0,031003	0,048941	0,02591	0,022586	0,028716	0,056031	0,042551	0,034553	0,028255	0,047543	0,034467	0,025359	0,024458	0,050299	0,042595	0	0,034341	0,04001	0,051949	0,037193	0,052616
	P4	0,027897	0,025377	0,028067	0,02007	0,038796	0,036637	0,038377	0,027486	0,023441	0,045622	0,024891	0,031835	0,023626	0,037045	0,058738	0,040166	0,041031	0,029329	0,024232	0,03386	0,022961	0,035856	0,027896	0,034341	0	0,051322	0,038574	0,051121	0,04299
	P8	0,024627	0,028108	0,033206	0,020992	0,03409	0,040854	0,033897	0,029534	0,019575	0,038986	0,023307	0,026772	0,02123	0,0384	0,049024	0,042019	0,042619	0,02885	0,024427	0,02912	0,020851	0,035281	0,029726	0,04001	0,051322	0	0,036182	0,048708	0,037611
	POz	0,022506	0,025701	0,031063	0,026166	0,036552	0,030748	0,028007	0,027071	0,02827	0,041739	0,023429	0,029393	0,034535	0,04979	0,052933	0,032842	0,025398	0,054112	0,027112	0,028091	0,024972	0,046508	0,046782	0,051949	0,038574	0,036182	0	0,039079	0,053105
	O1	0,025108	0,033294	0,034496	0,02325	0,036833	0,036035	0,029175	0,02842	0,018238	0,040612	0,024573	0,026143	0,021117	0,038736	0,048264	0,042209	0,044504	0,03063	0,028477	0,027626	0,021512	0,035639	0,027938	0,037193	0,051121	0,048708	0,039079	0	0,04053
	O2	0,027104	0,030432	0,033645	0,030908	0,042723	0,032847	0,034893	0,034464	0,032862	0,046423	0,030045	0,027435	0,037075	0,056629	0,057931	0,039827	0,029887	0,05079	0,032174	0,034013	0,025198	0,046579	0,043949	0,052616	0,04299	0,037611	0,053105	0,04053	0
