# Supplementary material for: Hyperbrain features of team mental models within a juggling paradigm: a proof of concept
Source: PeerJ. 2016 Sep 20;4:e2457. doi: 10.7717/peerj.2457 (PMC5036110; doi:10.7717/peerj.2457)
Supplement: Supplemental Information 5 [file peerj-04-2457-s005.pdf]

		subject 1													
		Fp1	Fpz	Fp2	F7	F3	Fz	F4	F8	FC5	FC1	FC2	FC6	T7	C3
subject 1	Fp1	0	0,0432025	0,00802402	0,05522735	0,02800458	0,02183564	0,00732266	0,07913773	0,06720126	0,00840932	0,04502185	0,03403625	0,03951835	0,06949392
	Fpz	0,0432025	0	0,04335705	0,01979167	0,0226331	0,02989305	0,01186288	0,06603882	0,04237711	0,02651526	0,03439514	0,01264838	0,01496897	0,02916185
	Fp2	0,00802402	0,04335705	0	0,03926275	0,0430828	0,01132296	0,01094433	0,0767484	0,06572306	0,00639451	0,02957892	0,02215185	0,03647162	0,06007165
	F7	0,05522735	0,01979167	0,03926275	0	0,02055701	0,04144894	0,02674143	0,04326184	0,03765962	0,02791821	0,00769151	0,01319225	0,00922577	0,02659412
	F3	0,02800458	0,0226331	0,0430828	0,02055701	0	0,0390004	0,02222861	0,05275825	0,01230761	0,03543336	0,0286126	0,0189475	0,02900268	0,01181234
	Fz	0,02183564	0,02989305	0,01132296	0,04144894	0,0390004	0	0,00828991	0,0195213	0,06437178	0,03058808	0,02903187	0,01991938	0,01647135	0,06343007
	F4	0,00732266	0,01186288	0,01094433	0,02674143	0,02222861	0,00828991	0	0,06029845	0,04462568	0,00393484	0,03642569	0,01691919	0,03018524	0,04150871
	F8	0,07913773	0,06603882	0,0767484	0,04326184	0,05275825	0,0195213	0,06029845	0	0,06207611	0,13580949	0,03027431	0,062872	0,01660742	0,06189402
	FC5	0,06720126	0,04237711	0,06572306	0,03765962	0,01230761	0,06437178	0,04462568	0,06207611	0	0,03344725	0,02843699	0,04328128	0,05870162	0,02671365
	FC1	0,00840932	0,02651526	0,00639451	0,02791821	0,03543336	0,03058808	0,00393484	0,13580949	0,03344725	0	0,02040726	0,01975348	0,03640405	0,03435322
	FC2	0,04502185	0,03439514	0,02957892	0,00769151	0,0286126	0,02903187	0,03642569	0,03027431	0,02843699	0,02040726	0	0,00396704	0,03884105	0,01760755
	FC6	0,03403625	0,01264838	0,02215185	0,01319225	0,0189475	0,01991938	0,01691919	0,062872	0,04328128	0,01975348	0,00396704	0	0,02100703	0,02665737
	T7	0,03951835	0,01496897	0,03647162	0,00922577	0,02900268	0,01647135	0,03018524	0,01660742	0,05870162	0,03640405	0,03884105	0,02100703	0	0,04547954
	C3	0,06949392	0,02916185	0,06007165	0,02659412	0,01181234	0,06343007	0,04150871	0,06189402	0,02671365	0,03435322	0,01760755	0,02665737	0,04547954	0
	Cz	0,03328946	0,01733935	0,02206491	0,00798415	0,01504826	0,02316291	0,01491778	0,07420465	0,03057396	0,0308571	0,00576138	0,00485203	0,03309884	0,02336415
	C4	0,0372599	0,02003857	0,0392528	0,01016811	0,01956755	0,03163582	0,00955058	0,06253219	0,02683916	0,04665242	0,01740313	0,01591146	0,03469545	0,02665978
	T8	0,04916041	0,01726771	0,04639029	0,04823817	0,03217738	0,03597685	0,03341955	0,10409851	0,05872843	0,07997781	0,05886694	0,04413665	0,01375602	0,04085383
	CP5	0,00789531	0,03915486	0,00341423	0,04193148	0,01980008	0,0134679	0,00491905	0,07460504	0,07023998	0,00304802	0,03382383	0,0229164	0,02169154	0,06978863
	CP1	0,04009605	0,03023819	0,03806665	0,01600929	0,03517537	0,00396079	0,03423487	0,10967686	0,01442098	0,04816643	0,0370935	0,02097598	0,02582776	0,00551325
	CP2	0,00790333	0,02292915	0,01429468	0,03197712	0,01478745	0,05183206	0,01077145	0,10068474	0,01290934	0,07745132	0,03717481	0,04044756	0,06773462	0,01209661
	CP6	0,05925614	0,03396601	0,05222044	0,02892457	0,02408472	0,01279034	0,04420179	0,12171276	0,01293999	0,09216163	0,00808535	0,02907576	0,03227191	0,01778541
	P7	0,03262923	0,01065728	0,03529105	0,01330406	0,03109324	0,00824164	0,03507057	0,01370444	0,06294066	0,04070326	0,03401855	0,02194892	0,00281066	0,05116858
	P3	0,07140515	0,06326514	0,06480317	0,0308125	0,01566772	0,05645412	0,07384489	0,02114437	0,02257536	0,05168842	0,04075192	0,04404482	0,05371323	0,01437006
	Pz	0,02180342	0,01245579	0,02353424	0,04074141	0,01090167	0,03399218	0,01665873	0,02512994	0,04096856	0,03073133	0,00597839	0,02462188	0,02267138	0,0396827
	P4	0,04593534	0,02300192	0,04164252	0,02855671	0,02748739	0,00519944	0,03895542	0,10478702	0,02573091	0,05553486	0,0264446	0,02416285	0,02197403	0,01345749
	P8	0,04054582	0,02821343	0,03702784	0,01412765	0,03119224	0,00683127	0,03162719	0,11569499	0,0312585	0,05935127	0,01187562	0,00769966	0,03467753	0,01809555
	POz	0,04806917	0,0355235	0,0449133	0,01411108	0,01092146	0,06331367	0,0505101	0,06479933	0,02633847	0,00653705	0,01515848	0,01835148	0,05398423	0,02016599
	O1	0,04484409	0,02952129	0,03126203	0,03174362	0,026252	0,02374071	0,03595911	0,00574145	0,03289188	0,0342755	0,03515888	0,03695861	0,04836564	0,02833276
	O2	0,07426685	0,05127986	0,0705179	0,025998	0,01975992	0,07525357	0,07081885	0,0174345	0,03122283	0,06474941	0,04097199	0,01918861	0,03922744	0,03473077
subject 2	Fp1	0,05441526	0,06431619	0,06441293	0,05733408	0,07387796	0,06811864	0,06408865	0,02860914	0,05028727	0,03656291	0,06433486	0,05899017	0,0579623	0,04945948
	Fpz	0,05118725	0,06604358	0,05831844	0,05999726	0,07292595	0,06153103	0,0594298	0,02361895	0,04816983	0,03275356	0,06487224	0,06214864	0,05627191	0,04266132
	Fp2	0,04559925	0,05657389	0,05338944	0,05069117	0,062985	0,057117	0,05256739	0,02202623	0,04295738	0,03439173	0,05701165	0,05188488	0,05002043	0,04381099
	F7	0,05539486	0,05581999	0,06078659	0,05538871	0,06831263	0,05957306	0,05889474	0,02537994	0,04785338	0,0367052	0,07228333	0,05782212	0,05714461	0,04526715
	F3	0,03822395	0,04525935	0,04104487	0,05754778	0,05651067	0,05922129	0,04630888	0,01661223	0,03941671	0,03216748	0,06335059	0,05533299	0,04490535	0,03784
	Fz	0,03156599	0,03717104	0,0342002	0,04324648	0,03772144	0,03971438	0,04072039	0,00619066	0,02455811	0,02913373	0,04031192	0,03788506	0,03539982	0,02502349
	F4	0,03744954	0,03955369	0,04020398	0,04220796	0,0406379	0,03736968	0,0435446	0,01216045	0,02520113	0,03086644	0,04491053	0,03798492	0,03736604	0,02629018
	F8	0,02726143	0,03278993	0,03340944	0,0311213	0,04088427	0,04107836	0,03189217	0,0109065	0,02314272	0,02426818	0,0442849	0,02837924	0,0289355	0,01741375
	FC5	0,0309226	0,03384447	0,03532205	0,02669594	0,03820012	0,0308955	0,03691104	0,00554537	0,01974637	0,02970621	0,03197937	0,02504278	0,02874492	0,01570985
	FC1	0,03420727	0,04876221	0,03873494	0,04963665	0,0486254	0,05456655	0,04174862	0,01252705	0,03351853	0,03276952	0,06264227	0,0491979	0,04631608	0,03517291
	FC2	0,0317343	0,03767944	0,0314029	0,03228609	0,03394663	0,02662391	0,03150588	0,02168252	0,02058346	0,03406484	0,03451005	0,02461824	0,03452888	0,02235988
	FC6	0,02024873	0,02849792	0,02496618	0,02189618	0,02839746	0,02679085	0,02821526	0,00688154	0,01729781	0,02597573	0,03028628	0,02224124	0,02692564	0,01337696
	T7	0,03001569	0,03074561	0,03365827	0,03183494	0,03677453	0,03067404	0,0350709	0,0057408	0,01901896	0,03006902	0,03722974	0,03120775	0,03218972	0,01408424
	C3	0,02613062	0,03313866	0,03009797	0,04218189	0,03791558	0,03442983	0,03895989	0,00365693	0,02300503	0,02834	0,04095586	0,04138716	0,03551221	0,0263581
	Cz	0,0241686	0,03285324	0,02777798	0,03261375	0,0361968	0,02203056	0,0290302	0,01443716	0,02601292	0,03042027	0,02791514	0,02828245	0,03188741	0,02764068
	C4	0,03239162	0,03815764	0,03931154	0,01994286	0,03056301	0,03612406	0,03950249	0,00627602	0,01127617	0,02813094	0,02189463	0,0183951	0,02700908	0,01011846
	T8	0,02237687	0,03353936	0,02687506	0,03089208	0,03737786	0,03364591	0,03507575	0,0179388	0,02724396	0,02289914	0,04065183	0,02937453	0,02787764	0,02391899
	CP5	0,02276536	0,02570052	0,02925902	0,02880958	0,02931619	0,0201228	0,03041172	0,00412518	0,01254867	0,02087838	0,0323534	0,02684022	0,02782868	0,01175196
	CP1	0,03831507	0,04145427	0,04541027	0,04285864	0,04580266	0,04945607	0,03890644	0,02631724	0,03309719	0,03219132	0,04234272	0,04014472	0,04026213	0,03178082
	CP2	0,03124432	0,04083377	0,03732915	0,02836183	0,0428597	0,02924451	0,03063048	0,01939651	0,02038314	0,03095413	0,03113962	0,0224655	0,03098967	0,0205517
	CP6	0,03697426	0,03812021	0,04318558	0,02225807	0,03737996	0,04077195	0,03877542	0,0106944	0,0172565	0,03506034	0,03795668	0,02232437	0,0281651	0,01287213
	P7	0,03433435	0,02804021	0,03018438	0,02413956	0,02129499	0,02532375	0,03137143	0,00173447	0,00592528	0,02923693	0,02925655	0,02687433	0,02804521	0,00465165
	P3	0,02920667	0,02688188	0,0334333	0,02512762	0,01939681	0,02429723	0,03465863	0,00433932	0,0025834	0,02783195	0,03283307	0,02544843	0,02500534	0,0018918
	Pz	0,0185923	0,02520321	0,02106058	0,02318243	0,0334412	0,01574522	0,02396554	0,00176149	0,01609028	0,02213264	0,0251219	0,02337231	0,02089279	0,01873563
	P4	0,02948938	0,04637472	0,03494645	0,03637854	0,04996914	0,03410869	0,03822332	0,00635629	0,02971415	0,02581164	0,04222084	0,03263212	0,03795067	0,03178875
	P8	0,02909421	0,04182203	0,03076206	0,0370076	0,04610922	0,03665933	0,03235645	0,00713258	0,02735637	0,02174643	0,04599259	0,02995797	0,03762167	0,02876792
	POz	0,03008037	0,03693162	0,03785987	0,04064336	0,04121701	0,03557892	0,03772458	0,01180517	0,02614963	0,02406069	0,04389755	0,03355572	0,03444506	0,02399199
	O1	0,0243265	0,03910311	0,03057214	0,0341923	0,04531207	0,03092932	0,03085686	0,00907089	0,02146174	0,02018068	0,04035307	0,02565972	0,03128989	0,02681884
	O2	0,03079246	0,04074968	0,03910855	0,04522701	0,05128286	0,04237081	0,04241567	0,01644037	0,03146368	0,02297325	0,05403604	0,04257154	0,03887102	0,02881433

		subject 1														
		Cz	C4	T8	CP5	CP1	CP2	CP6	P7	P3	Pz	P4	P8	POz	O1	O2
subject 1	Fp1	0,03328946	0,0372599	0,04916041	0,00789531	0,04009605	0,00790333	0,05925614	0,03262923	0,07140515	0,02180342	0,04593534	0,04054582	0,04806917	0,04484409	0,07426685
	Fpz	0,01733935	0,02003857	0,01726771	0,03915486	0,03023819	0,02292915	0,03396601	0,01065728	0,06326514	0,01245579	0,02300192	0,02821343	0,0355235	0,02952129	0,05127986
	Fp2	0,02206491	0,0392528	0,04639029	0,00341423	0,03806665	0,01429468	0,05222044	0,03529105	0,06480317	0,02353424	0,04164252	0,03702784	0,0449133	0,03126203	0,0705179
	F7	0,00798415	0,01016811	0,04823817	0,04193148	0,01600929	0,03197712	0,02892457	0,01330406	0,0308125	0,04074141	0,02855671	0,01412765	0,01411108	0,03174362	0,025998
	F3	0,01504826	0,01956755	0,03217738	0,01980008	0,03517537	0,01478745	0,02408472	0,03109324	0,01566772	0,01090167	0,02748739	0,03119224	0,01092146	0,026252	0,01975992
	Fz	0,02316291	0,03163582	0,03597685	0,0134679	0,00396079	0,05183206	0,01279034	0,00824164	0,05645412	0,03399218	0,00519944	0,00683127	0,06331367	0,02374071	0,07525357
	F4	0,01491778	0,00955058	0,03341955	0,00491905	0,03423487	0,01077145	0,04420179	0,03507057	0,07384489	0,01665873	0,03895542	0,03162719	0,0505101	0,03595911	0,07081885
	F8	0,07420465	0,06253219	0,10409851	0,07460504	0,10967686	0,10068474	0,12171276	0,01370444	0,02114437	0,02512994	0,10478702	0,11569499	0,06479933	0,00574145	0,0174345
	FC5	0,03057396	0,02683916	0,05872843	0,07023998	0,01442098	0,01290934	0,01293999	0,06294066	0,02257536	0,04096856	0,02573091	0,0312585	0,02633847	0,03289188	0,03122283
	FC1	0,0308571	0,04665242	0,07997781	0,00304802	0,04816643	0,07745132	0,09216163	0,04070326	0,05168842	0,03073133	0,05553486	0,05935127	0,00653705	0,0342755	0,06474941
	FC2	0,00576138	0,01740313	0,05886694	0,03382383	0,0370935	0,03717481	0,00808535	0,03401855	0,04075192	0,00597839	0,0264446	0,01187562	0,01515848	0,03515888	0,04097199
	FC6	0,00485203	0,01591146	0,04413665	0,0229164	0,02097598	0,04044756	0,02907576	0,02194892	0,04404482	0,02462188	0,02416285	0,00769966	0,01835148	0,03695861	0,01918861
	T7	0,03309884	0,03469545	0,01375602	0,02169154	0,02582776	0,06773462	0,03227191	0,00281066	0,05371323	0,02267138	0,02197403	0,03467753	0,05398423	0,04836564	0,03922744
	C3	0,02336415	0,02665978	0,04085383	0,06978863	0,00551325	0,01209661	0,01778541	0,05116858	0,01437006	0,0396827	0,01345749	0,01809555	0,02016599	0,02833276	0,03473077
	Cz	0	0,03140851	0,06482522	0,02623459	0,04049684	0,03576952	0,03093414	0,03415226	0,04260765	0,02590461	0,02764148	0,01855045	0,01147679	0,03420556	0,01625423
	C4	0,03140851	0	0,10160753	0,02909277	0,06543169	0,02439842	0,05756258	0,03248575	0,02156588	0,01013706	0,04873114	0,05041247	0,05356479	0,01413347	0,0088159
	T8	0,06482522	0,10160753	0	0,03772647	0,05473726	0,09534768	0,0969714	0,0254773	0,02792082	0,0567975	0,07211566	0,0969468	0,05207484	0,02084431	0,02087859
	CP5	0,02623459	0,02909277	0,03772647	0	0,03032635	0,0053329	0,04549289	0,02215185	0,06356983	0,00822972	0,03536321	0,02341056	0,0419738	0,04001794	0,06128863
	CP1	0,04049684	0,06543169	0,05473726	0,03032635	0	0,08811326	0,05723419	0,03483632	0,00326324	0,0173293	0,02537472	0,04199761	0,04831062	0,01089137	0,01163208
	CP2	0,03576952	0,02439842	0,09534768	0,0053329	0,08811326	0	0,02621667	0,07300479	0,04213181	0,02718588	0,05285335	0,05915316	0,00896339	0,03876529	0,0267926
	CP6	0,03093414	0,05756258	0,0969714	0,04549289	0,05723419	0,02621667	0	0,04168217	0,01503014	0,03560311	0,02783908	0,03045233	0,03116962	0,00330074	0,01242249
	P7	0,03415226	0,03248575	0,0254773	0,02215185	0,03483632	0,07300479	0,04168217	0	0,05537788	0,02345555	0,02633677	0,043601	0,0624699	0,04290044	0,03727617
	P3	0,04260765	0,02156588	0,02792082	0,06356983	0,00326324	0,04213181	0,01503014	0,05537788	0	0,02030545	0,00891154	0,02802657	0,04138146	0,05872428	0,0192843
	Pz	0,02590461	0,01013706	0,0567975	0,00822972	0,0173293	0,02718588	0,03560311	0,02345555	0,02030545	0	0,03577654	0,01485234	0,04892784	0,03862962	0,06955667
	P4	0,02764148	0,04873114	0,07211566	0,03536321	0,02537472	0,05285335	0,02783908	0,02633677	0,00891154	0,03577654	0	0,06695212	0,04118686	0,00564624	0,02643331
	P8	0,01855045	0,05041247	0,0969468	0,02341056	0,04199761	0,05915316	0,03045233	0,043601	0,02802657	0,01485234	0,06695212	0	0,04266527	0,00493366	0,01170224
	POz	0,01147679	0,05356479	0,05207484	0,0419738	0,04831062	0,00896339	0,03116962	0,0624699	0,04138146	0,04892784	0,04118686	0,04266527	0	0,03999001	0,03261435
	O1	0,03420556	0,01413347	0,02084431	0,04001794	0,01089137	0,03876529	0,00330074	0,04290044	0,05872428	0,03862962	0,00564624	0,00493366	0,03999001	0	0,0528744
	O2	0,01625423	0,0088159	0,02087859	0,06128863	0,01163208	0,0267926	0,01242249	0,03727617	0,0192843	0,06955667	0,02643331	0,01170224	0,03261435	0,0528744	0
subject 2	Fp1	0,06518208	0,04072997	0,0220087	0,06061108	0,0201232	0,06272248	0,04160399	0,06176802	0,07991661	0,02755021	0,03151793	0,04008067	0,06233184	0,05996538	0,06352476
	Fpz	0,05981489	0,03582959	0,01874776	0,06283214	0,02198772	0,06115296	0,038233	0,05403316	0,07130901	0,02561952	0,02505532	0,04383506	0,05728076	0,05887754	0,063215
	Fp2	0,05093526	0,03261003	0,0142476	0,04946225	0,01812892	0,05459866	0,03317309	0,05501046	0,07157963	0,02372772	0,02522981	0,04167552	0,05210489	0,05213959	0,05462868
	F7	0,05761057	0,03785091	0,02175162	0,0599155	0,02020505	0,0619416	0,04013655	0,04995485	0,08120731	0,0271911	0,03018646	0,04605042	0,05531013	0,0605226	0,06426616
	F3	0,05453097	0,034763	0,02268994	0,04369231	0,02924693	0,05639353	0,04244936	0,04541476	0,06557882	0,02944938	0,04151482	0,05029998	0,0545082	0,05526914	0,05484564
	Fz	0,03813526	0,02826163	0,01484251	0,03558101	0,02807313	0,04554725	0,03156719	0,03635813	0,04011264	0,01272279	0,02832663	0,04999024	0,03709698	0,03765449	0,0308576
	F4	0,04057278	0,02635014	0,00810929	0,03735792	0,03630942	0,03662113	0,03057128	0,03788197	0,04055619	0,00996627	0,02460511	0,05365827	0,03491088	0,03621166	0,03179092
	F8	0,02955224	0,02536159	0,01228139	0,02816404	0,01317656	0,04352617	0,03178409	0,03015405	0,03908468	0,01433006	0,02562909	0,0356904	0,04083276	0,03603155	0,03262538
	FC5	0,02435304	0,0233465	0,02439818	0,03583361	0,02271572	0,03267974	0,03344664	0,0305906	0,03336243	0,01122595	0,02962361	0,04010259	0,02527124	0,02948372	0,02475291
	FC1	0,04997947	0,03465106	0,01961002	0,04489932	0,02714642	0,05361188	0,04296488	0,04432391	0,05561619	0,02147792	0,03303256	0,04050792	0,05025724	0,05241415	0,05191341
	FC2	0,03259698	0,02152226	0,01677652	0,03276282	0,04245798	0,03316682	0,03500951	0,03170406	0,03495796	0,00972728	0,03915347	0,05053525	0,02520562	0,02527596	0,02478022
	FC6	0,02011329	0,02684969	0,0149791	0,02112243	0,01314352	0,03403606	0,0279384	0,02662556	0,02488273	0,01640701	0,03026262	0,02819483	0,02601764	0,02901664	0,02185164
	T7	0,03006211	0,02352555	0,0120907	0,03377769	0,01990151	0,03799181	0,03072087	0,02773738	0,03088325	0,01450907	0,02999886	0,03596347	0,02847339	0,02681688	0,02688361
	C3	0,04223578	0,03863144	0,01843735	0,03179582	0,02448309	0,04805878	0,03795642	0,03680929	0,03861316	0,01395893	0,02497703	0,02992055	0,03663978	0,03632577	0,02991671
	Cz	0,02966762	0,0211676	0,02185216	0,03198334	0,03755534	0,03086178	0,03687297	0,02947281	0,03623496	0,00935294	0,04026237	0,04697814	0,02105695	0,01885249	0,02504583
	C4	0,02342278	0,00895435	0,02666831	0,03886214	0,02550109	0,02222588	0,02820932	0,03006784	0,03268636	0,01124644	0,03306781	0,03377861	0,02443889	0,03330728	0,0264779
	T8	0,03185326	0,03032481	0,00507022	0,0269297	0,01255328	0,04701126	0,03121159	0,03023083	0,03820142	0,01836611	0,02771289	0,03007948	0,04298969	0,03975156	0,03727397
	CP5	0,02185516	0,02198939	0,01625787	0,02790598	0,01994428	0,03410418	0,03190829	0,0278657	0,03067521	0,00509932	0,02886062	0,02504287	0,018376	0,02291132	0,02248909
	CP1	0,03353527	0,02047505	0,01219405	0,04352785	0,01626002	0,03733546	0,0262967	0,0407245	0,05374237	0,0195398	0,01945406	0,03502893	0,04166843	0,04345731	0,04372491
	CP2	0,02154801	0,02264652	0,02848415	0,03925873	0,0339221	0,03089934	0,03812199	0,02768734	0,03326027	0,01240624	0,03757685	0,05233023	0,02342259	0,02578979	0,02038879
	CP6	0,02448768	0,01501282	0,01808388	0,03809252	0,03652225	0,0299932	0,0360277	0,02517386	0,03446597	0,01447855	0,03612138	0,04902942	0,03044784	0,03387584	0,02902059
	P7	0,02567611	0,02142134	0,01975253	0,02693344	0,02034093	0,03517428	0,03808194	0,02746105	0,03017505	0,00678047	0,02997574	0,0282916	0,0228572	0,02204553	0,02360974
	P3	0,02550789	0,0195474	0,02776886	0,02731385	0,02406705	0,03028832	0,03644081	0,02564874	0,02424998	0,00633902	0,03057766	0,02665591	0,01854936	0,01927712	0,02271431
	Pz	0,02390944	0,02330955	0,01826595	0,02366568	0,01959092	0,02924143	0,02726225	0,0245918	0,031413	0,00802837	0,03009081	0,02599263	0,01265175	0,02085513	0,02386409
	P4	0,03335447	0,02364305	0,01092966	0,04307058	0,01994711	0,0263414	0,02672303	0,03978918	0,04480046	0,00900693	0,02693861	0,04748751	0,02694554	0,03137228	0,03297608
	P8	0,03229038	0,02844448	0,0126214	0,03465147	0,01466406	0,04069054	0,02867521	0,03269492	0,04722822	0,0102377	0,0240991	0,0354538	0,03863273	0,03271601	0,0326876
	POz	0,03441226	0,0320464	0,00820838	0,03461908	0,01372309	0,04159131	0,02949452	0,03311923	0,04552862	0,01192545	0,02339082	0,02912856	0,03378708	0,03192256	0,03510964
	O1	0,02903421	0,02537718	0,01327489	0,03348265	0,00910546	0,04062459	0,02395996	0,02767721	0,04271013	0,01565812	0,02542022	0,03367126	0,03587516	0,03415777	0,03237418
	O2	0,04452323	0,03050706	0,01539799	0,04059946	0,01256667	0,04882057	0,03362953	0,03853629	0,05149276	0,01775887	0,02582176	0,0359841	0,03971928	0,04043859	0,04686499

		subject 2													
		Fp1	Fpz	Fp2	F7	F3	Fz	F4	F8	FC5	FC1	FC2	FC6	T7	C3
subject 1	Fp1	0,05441526	0,05118725	0,04559925	0,05539486	0,03822395	0,03156599	0,03744954	0,02726143	0,0309226	0,03420727	0,0317343	0,02024873	0,03001569	0,02613062
	Fpz	0,06431619	0,06604358	0,05657389	0,05581999	0,04525935	0,03717104	0,03955369	0,03278993	0,03384447	0,04876221	0,03767944	0,02849792	0,03074561	0,03313866
	Fp2	0,06441293	0,05831844	0,05338944	0,06078659	0,04104487	0,0342002	0,04020398	0,03340944	0,03532205	0,03873494	0,0314029	0,02496618	0,03365827	0,03009797
	F7	0,05733408	0,05999726	0,05069117	0,05538871	0,05754778	0,04324648	0,04220796	0,0311213	0,02669594	0,04963665	0,03228609	0,02189618	0,03183494	0,04218189
	F3	0,07387796	0,07292595	0,062985	0,06831263	0,05651067	0,03772144	0,0406379	0,04088427	0,03820012	0,0486254	0,03394663	0,02839746	0,03677453	0,03791558
	Fz	0,06811864	0,06153103	0,057117	0,05957306	0,05922129	0,03971438	0,03736968	0,04107836	0,0308955	0,05456655	0,02662391	0,02679085	0,03067404	0,03442983
	F4	0,06408865	0,0594298	0,05256739	0,05889474	0,04630888	0,04072039	0,0435446	0,03189217	0,03691104	0,04174862	0,03150588	0,02821526	0,0350709	0,03895989
	F8	0,02860914	0,02361895	0,02202623	0,02537994	0,01661223	0,00619066	0,01216045	0,0109065	0,00554537	0,01252705	0,02168252	0,00688154	0,0057408	0,00365693
	FC5	0,05028727	0,04816983	0,04295738	0,04785338	0,03941671	0,02455811	0,02520113	0,02314272	0,01974637	0,03351853	0,02058346	0,01729781	0,01901896	0,02300503
	FC1	0,03656291	0,03275356	0,03439173	0,0367052	0,03216748	0,02913373	0,03086644	0,02426818	0,02970621	0,03276952	0,03406484	0,02597573	0,03006902	0,02834
	FC2	0,06433486	0,06487224	0,05701165	0,07228333	0,06335059	0,04031192	0,04491053	0,0442849	0,03197937	0,06264227	0,03451005	0,03028628	0,03722974	0,04095586
	FC6	0,05899017	0,06214864	0,05188488	0,05782212	0,05533299	0,03788506	0,03798492	0,02837924	0,02504278	0,0491979	0,02461824	0,02224124	0,03120775	0,04138716
	T7	0,0579623	0,05627191	0,05002043	0,05714461	0,04490535	0,03539982	0,03736604	0,0289355	0,02874492	0,04631608	0,03452888	0,02692564	0,03218972	0,03551221
	C3	0,04945948	0,04266132	0,04381099	0,04526715	0,03784	0,02502349	0,02629018	0,01741375	0,01570985	0,03517291	0,02235988	0,01337696	0,01408424	0,0263581
	Cz	0,06518208	0,05981489	0,05093526	0,05761057	0,05453097	0,03813526	0,04057278	0,02955224	0,02435304	0,04997947	0,03259698	0,02011329	0,03006211	0,04223578
	C4	0,04072997	0,03582959	0,03261003	0,03785091	0,034763	0,02826163	0,02635014	0,02536159	0,0233465	0,03465106	0,02152226	0,02684969	0,02352555	0,03863144
	T8	0,0220087	0,01874776	0,0142476	0,02175162	0,02268994	0,01484251	0,00810929	0,01228139	0,02439818	0,01961002	0,01677652	0,0149791	0,0120907	0,01843735
	CP5	0,06061108	0,06283214	0,04946225	0,0599155	0,04369231	0,03558101	0,03735792	0,02816404	0,03583361	0,04489932	0,03276282	0,02112243	0,03377769	0,03179582
	CP1	0,0201232	0,02198772	0,01812892	0,02020505	0,02924693	0,02807313	0,03630942	0,01317656	0,02271572	0,02714642	0,04245798	0,01314352	0,01990151	0,02448309
	CP2	0,06272248	0,06115296	0,05459866	0,0619416	0,05639353	0,04554725	0,03662113	0,04352617	0,03267974	0,05361188	0,03316682	0,03403606	0,03799181	0,04805878
	CP6	0,04160399	0,038233	0,03317309	0,04013655	0,04244936	0,03156719	0,03057128	0,03178409	0,03344664	0,04296488	0,03500951	0,0279384	0,03072087	0,03795642
	P7	0,06176802	0,05403316	0,05501046	0,04995485	0,04541476	0,03635813	0,03788197	0,03015405	0,0305906	0,04432391	0,03170406	0,02662556	0,02773738	0,03680929
	P3	0,07991661	0,07130901	0,07157963	0,08120731	0,06557882	0,04011264	0,04055619	0,03908468	0,03336243	0,05561619	0,03495796	0,02488273	0,03088325	0,03861316
	Pz	0,02755021	0,02561952	0,02372772	0,0271911	0,02944938	0,01272279	0,00996627	0,01433006	0,01122595	0,02147792	0,00972728	0,01640701	0,01450907	0,01395893
	P4	0,03151793	0,02505532	0,02522981	0,03018646	0,04151482	0,02832663	0,02460511	0,02562909	0,02962361	0,03303256	0,03915347	0,03026262	0,02999886	0,02497703
	P8	0,04008067	0,04383506	0,04167552	0,04605042	0,05029998	0,04999024	0,05365827	0,0356904	0,04010259	0,04050792	0,05053525	0,02819483	0,03596347	0,02992055
	POz	0,06233184	0,05728076	0,05210489	0,05531013	0,0545082	0,03709698	0,03491088	0,04083276	0,02527124	0,05025724	0,02520562	0,02601764	0,02847339	0,03663978
	O1	0,05996538	0,05887754	0,05213959	0,0605226	0,05526914	0,03765449	0,03621166	0,03603155	0,02948372	0,05241415	0,02527596	0,02901664	0,02681688	0,03632577
	O2	0,06352476	0,063215	0,05462868	0,06426616	0,05484564	0,0308576	0,03179092	0,03262538	0,02475291	0,05191341	0,02478022	0,02185164	0,02688361	0,02991671
subject 2	Fp1	0	0,00228285	0,0049551	0,0030054	0,07959572	0,03667284	0,01343304	0,0282995	0,10920586	0,03428041	0,050158	0,09622337	0,06866321	0,04005178
	Fpz	0,00228285	0	0,00181483	0,00213997	0,05707186	0,04490767	0,02355156	0,02735956	0,09344189	0,02520143	0,05699086	0,09105767	0,06251511	0,03640821
	Fp2	0,0049551	0,00181483	0	0,00277863	0,04330323	0,05189995	0,03813913	0,0207103	0,07595838	0,0322312	0,07113606	0,0827523	0,0478979	0,04556611
	F7	0,0030054	0,00213997	0,00277863	0	0,07291152	0,03489871	0,01256413	0,033714	0,0984007	0,02446084	0,05389524	0,09220698	0,06882662	0,03752056
	F3	0,07959572	0,05707186	0,04330323	0,07291152	0	0,01684041	0,00919004	0,04052484	0,12836604	0,01042166	0,03137707	0,1023688	0,10226859	0,01441919
	Fz	0,03667284	0,04490767	0,05189995	0,03489871	0,01684041	0	0,0100355	0,07011339	0,13118354	0,00497441	0,01315345	0,09327151	0,11444947	0,02321621
	F4	0,01343304	0,02355156	0,03813913	0,01256413	0,00919004	0,0100355	0	0,05156459	0,12541505	0,00961215	0,01733599	0,08152151	0,08979167	0,04449914
	F8	0,0282995	0,02735956	0,0207103	0,033714	0,04052484	0,07011339	0,05156459	0	0,00806643	0,06152469	0,05621569	0,02577723	0,0036507	0,06196109
	FC5	0,10920586	0,09344189	0,07595838	0,0984007	0,12836604	0,13118354	0,12541505	0,00806643	0	0,15453359	0,08461349	0,00368687	0,00146385	0,12541632
	FC1	0,03428041	0,02520143	0,0322312	0,02446084	0,01042166	0,00497441	0,00961215	0,06152469	0,15453359	0	0,02439965	0,10636761	0,11532407	0,01461743
	FC2	0,050158	0,05699086	0,07113606	0,05389524	0,03137707	0,01315345	0,01733599	0,05621569	0,08461349	0,02439965	0	0,05549462	0,07296802	0,02984744
	FC6	0,09622337	0,09105767	0,0827523	0,09220698	0,1023688	0,09327151	0,08152151	0,02577723	0,00368687	0,10636761	0,05549462	0	0,01240264	0,10044084
	T7	0,06866321	0,06251511	0,0478979	0,06882662	0,10226859	0,11444947	0,08979167	0,0036507	0,00146385	0,11532407	0,07296802	0,01240264	0	0,12658182
	C3	0,04005178	0,03640821	0,04556611	0,03752056	0,01441919	0,02321621	0,04449914	0,06196109	0,12541632	0,01461743	0,02984744	0,10044084	0,12658182	0
	Cz	0,08439303	0,09108243	0,09313466	0,0845057	0,06094913	0,02446632	0,02739462	0,07312844	0,08409622	0,04711197	0,00836418	0,05935263	0,08326868	0,00767669
	C4	0,07587714	0,07362689	0,06801209	0,06909897	0,07264034	0,06968822	0,06720603	0,04886482	0,0548814	0,07621527	0,0352707	0,03872111	0,0487648	0,05552946
	T8	0,00951282	0,0111771	0,01552291	0,00376852	0,02131804	0,02466586	0,02348791	0,00364142	0,01119993	0,00860768	0,03511268	0,04254321	0,01053413	0,02134015
	CP5	0,05243111	0,04888308	0,03597927	0,05506804	0,06596364	0,07904194	0,05023766	0,02509126	0,018415	0,08307019	0,04118369	0,0278187	0,01506136	0,11251725
	CP1	0,11361532	0,10859579	0,11121139	0,10923483	0,11772431	0,04978721	0,02873547	0,07057148	0,02221955	0,08536917	0,02612447	0,02222692	0,05180834	0,10543892
	CP2	0,12627797	0,11472466	0,10521437	0,11249628	0,12613283	0,11218114	0,14816596	0,04088521	0,02382066	0,14329629	0,07505301	0,03984074	0,0256957	0,09964423
	CP6	0,04113756	0,0424758	0,0404927	0,03791918	0,06220426	0,08560663	0,10806397	0,06203402	0,03176475	0,0857677	0,0888032	0,0544265	0,03690077	0,04766605
	P7	0,04270316	0,04090967	0,03457199	0,04184514	0,03369515	0,0374189	0,01204586	0,03591757	0,03916241	0,04237291	0,01009783	0,0357348	0,03654823	0,08459772
	P3	0,0449003	0,04362162	0,04155924	0,04318646	0,02986808	0,03140649	0,01120092	0,03699242	0,04780148	0,03740903	0,00644369	0,03937739	0,04470313	0,07467539
	Pz	0,05945162	0,0534926	0,04152984	0,06002238	0,09785439	0,1011138	0,08558495	0,00902654	0,01189054	0,11464333	0,06300122	0,01065977	0,01071528	0,12671222
	P4	0,03438035	0,03164035	0,02287756	0,02865117	0,06147673	0,09459582	0,10338016	0,04374275	0,00971714	0,08252855	0,0949808	0,03062819	0,01906178	0,05340407
	P8	0,03766529	0,03425833	0,02609919	0,03471795	0,05467627	0,08117334	0,07050732	0,00777735	0,01523671	0,06846576	0,07192565	0,01306382	0,00828346	0,05550925
	POz	0,02151192	0,01758746	0,01977331	0,01849158	0,03533354	0,05392526	0,03900873	0,01657188	0,02153803	0,05390262	0,05162154	0,03871323	0,01098164	0,08673453
	O1	0,04720873	0,04978984	0,0395848	0,04926338	0,06362193	0,07870853	0,07116882	0,00267795	0,01260563	0,07556054	0,06168955	0,01155717	0,01253057	0,05883705
	O2	0,01773969	0,01235035	0,01311705	0,01521903	0,03645102	0,07087862	0,04773676	0,00571847	0,03606371	0,06395037	0,05464333	0,0442982	0,02329515	0,09917656

		subject 2														
		Cz	C4	T8	CP5	CP1	CP2	CP6	P7	P3	Pz	P4	P8	POz	O1	O2
subject 1	Fp1	0,0241686	0,03239162	0,02237687	0,02276536	0,03831507	0,03124432	0,03697426	0,03433435	0,02920667	0,0185923	0,02948938	0,02909421	0,03008037	0,0243265	0,03079246
	Fpz	0,03285324	0,03815764	0,03353936	0,02570052	0,04145427	0,04083377	0,03812021	0,02804021	0,02688188	0,02520321	0,04637472	0,04182203	0,03693162	0,03910311	0,04074968
	Fp2	0,02777798	0,03931154	0,02687506	0,02925902	0,04541027	0,03732915	0,04318558	0,03018438	0,0334333	0,02106058	0,03494645	0,03076206	0,03785987	0,03057214	0,03910855
	F7	0,03261375	0,01994286	0,03089208	0,02880958	0,04285864	0,02836183	0,02225807	0,02413956	0,02512762	0,02318243	0,03637854	0,0370076	0,04064336	0,0341923	0,04522701
	F3	0,0361968	0,03056301	0,03737786	0,02931619	0,04580266	0,0428597	0,03737996	0,02129499	0,01939681	0,0334412	0,04996914	0,04610922	0,04121701	0,04531207	0,05128286
	Fz	0,02203056	0,03612406	0,03364591	0,0201228	0,04945607	0,02924451	0,04077195	0,02532375	0,02429723	0,01574522	0,03410869	0,03665933	0,03557892	0,03092932	0,04237081
	F4	0,0290302	0,03950249	0,03507575	0,03041172	0,03890644	0,03063048	0,03877542	0,03137143	0,03465863	0,02396554	0,03822332	0,03235645	0,03772458	0,03085686	0,04241567
	F8	0,01443716	0,00627602	0,0179388	0,00412518	0,02631724	0,01939651	0,0106944	0,00173447	0,00433932	0,00176149	0,00635629	0,00713258	0,01180517	0,00907089	0,01644037
	FC5	0,02601292	0,01127617	0,02724396	0,01254867	0,03309719	0,02038314	0,0172565	0,00592528	0,0025834	0,01609028	0,02971415	0,02735637	0,02614963	0,02146174	0,03146368
	FC1	0,03042027	0,02813094	0,02289914	0,02087838	0,03219132	0,03095413	0,03506034	0,02923693	0,02783195	0,02213264	0,02581164	0,02174643	0,02406069	0,02018068	0,02297325
	FC2	0,02791514	0,02189463	0,04065183	0,0323534	0,04234272	0,03113962	0,03795668	0,02925655	0,03283307	0,0251219	0,04222084	0,04599259	0,04389755	0,04035307	0,05403604
	FC6	0,02828245	0,0183951	0,02937453	0,02684022	0,04014472	0,0224655	0,02232437	0,02687433	0,02544843	0,02337231	0,03263212	0,02995797	0,03355572	0,02565972	0,04257154
	T7	0,03188741	0,02700908	0,02787764	0,02782868	0,04026213	0,03098967	0,0281651	0,02804521	0,02500534	0,02089279	0,03795067	0,03762167	0,03444506	0,03128989	0,03887102
	C3	0,02764068	0,01011846	0,02391899	0,01175196	0,03178082	0,0205517	0,01287213	0,00465165	0,0018918	0,01873563	0,03178875	0,02876792	0,02399199	0,02681884	0,02881433
	Cz	0,02966762	0,02342278	0,03185326	0,02185516	0,03353527	0,02154801	0,02448768	0,02567611	0,02550789	0,02390944	0,03335447	0,03229038	0,03441226	0,02903421	0,04452323
	C4	0,0211676	0,00895435	0,03032481	0,02198939	0,02047505	0,02264652	0,01501282	0,02142134	0,0195474	0,02330955	0,02364305	0,02844448	0,0320464	0,02537718	0,03050706
	T8	0,02185216	0,02666831	0,00507022	0,01625787	0,01219405	0,02848415	0,01808388	0,01975253	0,02776886	0,01826595	0,01092966	0,0126214	0,00820838	0,01327489	0,01539799
	CP5	0,03198334	0,03886214	0,0269297	0,02790598	0,04352785	0,03925873	0,03809252	0,02693344	0,02731385	0,02366568	0,04307058	0,03465147	0,03461908	0,03348265	0,04059946
	CP1	0,03755534	0,02550109	0,01255328	0,01994428	0,01626002	0,0339221	0,03652225	0,02034093	0,02406705	0,01959092	0,01994711	0,01466406	0,01372309	0,00910546	0,01256667
	CP2	0,03086178	0,02222588	0,04701126	0,03410418	0,03733546	0,03089934	0,0299932	0,03517428	0,03028832	0,02924143	0,0263414	0,04069054	0,04159131	0,04062459	0,04882057
	CP6	0,03687297	0,02820932	0,03121159	0,03190829	0,0262967	0,03812199	0,0360277	0,03808194	0,03644081	0,02726225	0,02672303	0,02867521	0,02949452	0,02395996	0,03362953
	P7	0,02947281	0,03006784	0,03023083	0,0278657	0,0407245	0,02768734	0,02517386	0,02746105	0,02564874	0,0245918	0,03978918	0,03269492	0,03311923	0,02767721	0,03853629
	P3	0,03623496	0,03268636	0,03820142	0,03067521	0,05374237	0,03326027	0,03446597	0,03017505	0,02424998	0,031413	0,04480046	0,04722822	0,04552862	0,04271013	0,05149276
	Pz	0,00935294	0,01124644	0,01836611	0,00509932	0,0195398	0,01240624	0,01447855	0,00678047	0,00633902	0,00802837	0,00900693	0,0102377	0,01192545	0,01565812	0,01775887
	P4	0,04026237	0,03306781	0,02771289	0,02886062	0,01945406	0,03757685	0,03612138	0,02997574	0,03057766	0,03009081	0,02693861	0,0240991	0,02339082	0,02542022	0,02582176
	P8	0,04697814	0,03377861	0,03007948	0,02504287	0,03502893	0,05233023	0,04902942	0,0282916	0,02665591	0,02599263	0,04748751	0,0354538	0,02912856	0,03367126	0,0359841
	POz	0,02105695	0,02443889	0,04298969	0,018376	0,04166843	0,02342259	0,03044784	0,0228572	0,01854936	0,01265175	0,02694554	0,03863273	0,03378708	0,03587516	0,03971928
	O1	0,01885249	0,03330728	0,03975156	0,02291132	0,04345731	0,02578979	0,03387584	0,02204553	0,01927712	0,02085513	0,03137228	0,03271601	0,03192256	0,03415777	0,04043859
	O2	0,02504583	0,0264779	0,03727397	0,02248909	0,04372491	0,02038879	0,02902059	0,02360974	0,02271431	0,02386409	0,03297608	0,0326876	0,03510964	0,03237418	0,04686499
subject 2	Fp1	0,08439303	0,07587714	0,00951282	0,05243111	0,11361532	0,12627797	0,04113756	0,04270316	0,0449003	0,05945162	0,03438035	0,03766529	0,02151192	0,04720873	0,01773969
	Fpz	0,09108243	0,07362689	0,0111771	0,04888308	0,10859579	0,11472466	0,0424758	0,04090967	0,04362162	0,0534926	0,03164035	0,03425833	0,01758746	0,04978984	0,01235035
	Fp2	0,09313466	0,06801209	0,01552291	0,03597927	0,11121139	0,10521437	0,0404927	0,03457199	0,04155924	0,04152984	0,02287756	0,02609919	0,01977331	0,0395848	0,01311705
	F7	0,0845057	0,06909897	0,00376852	0,05506804	0,10923483	0,11249628	0,03791918	0,04184514	0,04318646	0,06002238	0,02865117	0,03471795	0,01849158	0,04926338	0,01521903
	F3	0,06094913	0,07264034	0,02131804	0,06596364	0,11772431	0,12613283	0,06220426	0,03369515	0,02986808	0,09785439	0,06147673	0,05467627	0,03533354	0,06362193	0,03645102
	Fz	0,02446632	0,06968822	0,02466586	0,07904194	0,04978721	0,11218114	0,08560663	0,0374189	0,03140649	0,1011138	0,09459582	0,08117334	0,05392526	0,07870853	0,07087862
	F4	0,02739462	0,06720603	0,02348791	0,05023766	0,02873547	0,14816596	0,10806397	0,01204586	0,01120092	0,08558495	0,10338016	0,07050732	0,03900873	0,07116882	0,04773676
	F8	0,07312844	0,04886482	0,00364142	0,02509126	0,07057148	0,04088521	0,06203402	0,03591757	0,03699242	0,00902654	0,04374275	0,00777735	0,01657188	0,00267795	0,00571847
	FC5	0,08409622	0,0548814	0,01119993	0,018415	0,02221955	0,02382066	0,03176475	0,03916241	0,04780148	0,01189054	0,00971714	0,01523671	0,02153803	0,01260563	0,03606371
	FC1	0,04711197	0,07621527	0,00860768	0,08307019	0,08536917	0,14329629	0,0857677	0,04237291	0,03740903	0,11464333	0,08252855	0,06846576	0,05390262	0,07556054	0,06395037
	FC2	0,00836418	0,0352707	0,03511268	0,04118369	0,02612447	0,07505301	0,0888032	0,01009783	0,00644369	0,06300122	0,0949808	0,07192565	0,05162154	0,06168955	0,05464333
	FC6	0,05935263	0,03872111	0,04254321	0,0278187	0,02222692	0,03984074	0,0544265	0,0357348	0,03937739	0,01065977	0,03062819	0,01306382	0,03871323	0,01155717	0,0442982
	T7	0,08326868	0,0487648	0,01053413	0,01506136	0,05180834	0,0256957	0,03690077	0,03654823	0,04470313	0,01071528	0,01906178	0,00828346	0,01098164	0,01253057	0,02329515
	C3	0,00767669	0,05552946	0,02134015	0,11251725	0,10543892	0,09964423	0,04766605	0,08459772	0,07467539	0,12671222	0,05340407	0,05550925	0,08673453	0,05883705	0,09917656
	Cz	0	0,0386211	0,041126	0,06059416	0,01089301	0,06538584	0,07037751	0,03001275	0,0209647	0,07938465	0,09848696	0,08082045	0,07322724	0,06931487	0,08247474
	C4	0,0386211	0	0,03754671	0,03450785	0,07134395	0,02014163	0,01688338	0,02326606	0,01545012	0,03939149	0,02539966	0,04669603	0,03790488	0,0423546	0,05120797
	T8	0,041126	0,03754671	0	0,02308832	0,0969013	0,03563341	0,0566468	0,03168423	0,03381974	0,00949958	0,0499639	0,01396797	0,02711463	0,00032901	0,01987211
	CP5	0,06059416	0,03450785	0,02308832	0	0,03648069	0,01205201	0,01457862	0,01021602	0,01850794	0,02020944	0,00544201	0,02313389	0,02001621	0,03012897	0,02902356
	CP1	0,01089301	0,07134395	0,0969013	0,03648069	0	0,07060697	0,04312921	0,04178926	0,02711677	0,05388114	0,02715386	0,05903062	0,10144838	0,0564453	0,12652468
	CP2	0,06538584	0,02014163	0,03563341	0,01205201	0,07060697	0	0,01421571	0,00302729	0,00396312	0,01886116	0,03551096	0,03526434	0,03179666	0,03292396	0,04581255
	CP6	0,07037751	0,01688338	0,0566468	0,01457862	0,04312921	0,01421571	0	0,01470279	0,01047479	0,02845922	0,01146141	0,04426465	0,03517507	0,05606833	0,03522346
	P7	0,03001275	0,02326606	0,03168423	0,01021602	0,04178926	0,00302729	0,01470279	0	0,00410881	0,04057228	0,01124572	0,03055916	0,02414882	0,02692322	0,02975559
	P3	0,0209647	0,01545012	0,03381974	0,01850794	0,02711677	0,00396312	0,01047479	0,00410881	0	0,04342299	0,00798616	0,02653887	0,03312013	0,02703974	0,04129976
	Pz	0,07938465	0,03939149	0,00949958	0,02020944	0,05388114	0,01886116	0,02845922	0,04057228	0,04342299	0	0,00990464	0,01483201	0,01310334	0,01474832	0,02120834
	P4	0,09848696	0,02539966	0,0499639	0,00544201	0,02715386	0,03551096	0,01146141	0,01124572	0,00798616	0,00990464	0	0,02463782	0,02615746	0,03338637	0,01941157
	P8	0,08082045	0,04669603	0,01396797	0,02313389	0,05903062	0,03526434	0,04426465	0,03055916	0,02653887	0,01483201	0,02463782	0	0,02657825	0,00033614	0,01199089
	POz	0,07322724	0,03790488	0,02711463	0,02001621	0,10144838	0,03179666	0,03517507	0,02414882	0,03312013	0,01310334	0,02615746	0,02657825	0	0,01884023	0,00583822
	O1	0,06931487	0,0423546	0,00032901	0,03012897	0,0564453	0,03292396	0,05606833	0,02692322	0,02703974	0,01474832	0,03338637	0,00033614	0,01884023	0	0,01839169
	O2	0,08247474	0,05120797	0,01987211	0,02902356	0,12652468	0,04581255	0,03522346	0,02975559	0,04129976	0,02120834	0,01941157	0,01199089	0,00583822	0,01839169	0
