# Supplementary material for: Hyperbrain features of team mental models within a juggling paradigm: a proof of concept
Source: PeerJ. 2016 Sep 20;4:e2457. doi: 10.7717/peerj.2457 (PMC5036110; doi:10.7717/peerj.2457)
Supplement: Supplemental Information 6 [file peerj-04-2457-s006.pdf]

		subject 1														
		Fp1	Fpz	Fp2	F7	F3	Fz	F4	F8	FC5	FC1	FC2	FC6	T7	C3	Cz
subject 1	Fp1	0	0,005748	0,00829012	0,02063508	0,04169036	0,01515893	0,01053388	0,07237855	0,02924648	0,01099485	0,03980928	0,02575756	0,01557094	0,0143728	0,0201444
	Fpz	0,005748	0	0,00646119	0,0205572	0,02853884	0,00921853	0,01226433	0,06182754	0,02967962	0,02106624	0,03072685	0,02238107	0,00880036	0,01565978	0,02615838
	Fp2	0,00829012	0,00646119	0	0,0267284	0,02829613	0,0118488	0,01373468	0,07255698	0,02151077	0,017607	0,03631431	0,02657054	0,02598457	0,02039578	0,01850952
	F7	0,02063508	0,0205572	0,0267284	0	0,02493714	0,02390263	0,01963205	0,0412696	0,03255678	0,02720996	0,03177585	0,01477141	0,00803028	0,01783742	0,00960947
	F3	0,04169036	0,02853884	0,02829613	0,02493714	0	0,02978176	0,0443737	0,04774551	0,01559587	0,04326127	0,01965269	0,02174804	0,00659711	0,0135876	0,02733292
	Fz	0,01515893	0,00921853	0,0118488	0,02390263	0,02978176	0	0,01334741	0,02358435	0,04229573	0,02885079	0,03227693	0,02765873	0,01552533	0,02492052	0,0323132
	F4	0,01053388	0,01226433	0,01373468	0,01963205	0,0443737	0,01334741	0	0,05064719	0,03314659	0,01660545	0,03848919	0,02526872	0,01793015	0,02117879	0,01883709
	F8	0,07237855	0,06182754	0,07255698	0,0412696	0,04774551	0,02358435	0,05064719	0	0,05641779	0,09976302	0,03532232	0,03783384	0,0330386	0,05356132	0,04916877
	FC5	0,02924648	0,02967962	0,02151077	0,03255678	0,01559587	0,04229573	0,03314659	0,05641779	0	0,02877792	0,03280351	0,03967851	0,05549457	0,02029207	0,02721936
	FC1	0,01099485	0,02106624	0,017607	0,02720996	0,04326127	0,02885079	0,01660545	0,09976302	0,02877792	0	0,02217824	0,02777068	0,02245099	0,02512676	0,03955929
	FC2	0,03980928	0,03072685	0,03631431	0,03177585	0,01965269	0,03227693	0,03848919	0,03532232	0,03280351	0,02217824	0	0,01738232	0,02023373	0,01824559	0,01328976
	FC6	0,02575756	0,02238107	0,02657054	0,01477141	0,02174804	0,02765873	0,02526872	0,03783384	0,03967851	0,02777068	0,01738232	0	0,00875358	0,01916546	0,00764817
	T7	0,01557094	0,00880036	0,02598457	0,00803028	0,00659711	0,01552533	0,01793015	0,0330386	0,05549457	0,02245099	0,02023373	0,00875358	0	0,03614641	0,00868226
	C3	0,0143728	0,01565978	0,02039578	0,01783742	0,0135876	0,02492052	0,02117879	0,05356132	0,02029207	0,02512676	0,01824559	0,01916546	0,03614641	0	0,00986828
	Cz	0,0201444	0,02615838	0,01850952	0,00960947	0,02733292	0,0323132	0,01883709	0,04916877	0,02721936	0,03955929	0,01328976	0,00764817	0,00868226	0,00986828	0
	C4	0,01219253	0,0227577	0,02544307	0,05312251	0,03060491	0,03236352	0,01412167	0,07676233	0,02462406	0,04218619	0,0209103	0,0436258	0,02585927	0,02832352	0,03744761
	T8	0,04236465	0,01686437	0,0413913	0,02311194	0,02639183	0,01501657	0,02602174	0,10111536	0,00493257	0,09864543	0,03810648	0,03899738	0,02549275	0,01153228	0,05015578
	CP5	0,0048027	0,00566654	0,00601529	0,024257	0,03746635	0,01014468	0,00639976	0,05953446	0,03158542	0,01785268	0,02926423	0,02366416	0,01278077	0,01343725	0,02022267
	CP1	0,02219273	0,01524668	0,03668619	0,04048939	0,03073654	0,01123106	0,01306693	0,07137118	0,01774926	0,05031986	0,04153545	0,04305094	0,01807667	0,02056909	0,05726948
	CP2	0,01908637	0,018021	0,02681292	0,03151441	0,02437387	0,03870973	0,01528853	0,05556778	0,01786798	0,05900951	0,02212456	0,04026491	0,03205358	0,0130871	0,04082264
	CP6	0,04013816	0,02367179	0,04492807	0,03092463	0,02932833	0,01205074	0,02436393	0,05926828	0,02397248	0,06275283	0,02258002	0,02453085	0,01847532	0,01800964	0,02937186
	P7	0,01550572	0,00736924	0,02365376	0,01615821	0,00725403	0,01200611	0,01706862	0,03429392	0,05544091	0,0216528	0,01549081	0,00923585	0,00228975	0,0416557	0,00878573
	P3	0,04529791	0,0345204	0,04406697	0,01093761	0,017575	0,03725667	0,03935439	0,02650577	0,02281669	0,0369178	0,0172692	0,01930557	0,02059004	0,02714196	0,023563
	Pz	0,03730357	0,02318547	0,02410541	0,00491978	0,01798031	0,03424042	0,02445546	0,01982322	0,02927382	0,02897752	0,02996034	0,01503523	0,01544644	0,0062341	0,0172713
	P4	0,02201235	0,01904777	0,03161456	0,04433651	0,0270453	0,00758813	0,01790372	0,08355903	0,02551265	0,0364356	0,05472728	0,05045313	0,01441932	0,01887136	0,06031052
	P8	0,04888394	0,0303343	0,04800209	0,03446546	0,05227403	0,01222763	0,0311297	0,06691856	0,02420407	0,06694568	0,03010656	0,03084879	0,01829336	0,03003468	0,03420069
	POz	0,02806969	0,03030002	0,03426728	0,03362038	0,02595649	0,0515513	0,03736556	0,04703841	0,01089295	0,02389548	0,03174524	0,04371221	0,03984802	0,00627816	0,03673302
	O1	0,03106003	0,03587527	0,02875725	0,03527772	0,02658943	0,02336468	0,02830456	0,01293907	0,01432123	0,03265113	0,04936868	0,04350429	0,03848647	0,01389118	0,03869482
	O2	0,03984499	0,03448826	0,04778926	0,02242042	0,03593295	0,03618445	0,0373889	0,02047286	0,02497966	0,03586516	0,02764467	0,02894689	0,02113966	0,01988765	0,02774371
subject 2	Fp1	0,01178248	0,01129309	0,01375432	0,00835619	0,01158885	0,0081875	0,01412929	0,00709347	0,00972517	0,02147558	0,00495943	0,00696729	0,00728695	0,01308567	0,01195934
	Fpz	0,00787339	0,00808894	0,01436217	0,00760679	0,00645808	0,00851609	0,01492992	0,00543985	0,00715263	0,01920362	0,00302618	0,00504561	0,00786416	0,00856407	0,00947086
	Fp2	0,00668469	0,00690453	0,0090756	0,00791617	0,00187208	0,01208763	0,01091843	0,0046338	0,00612373	0,01058052	0,00562897	0,00733916	0,00983181	0,00335943	0,00193432
	F7	0,01036068	0,01160361	0,0128837	0,00899498	0,00895302	0,00942953	0,01446838	0,00565019	0,00923706	0,02051089	0,00283191	0,00606514	0,00857021	0,01080119	0,01027467
	F3	0,0134002	0,00670195	0,01062935	0,01225011	0,01355351	0,00852535	0,01959158	0,0194419	0,012484	0,02093578	0,00400018	0,00567883	0,01112651	0,01090503	0,0077509
	Fz	0,0073715	0,01038896	0,00409787	0,00767693	0,0036061	0,01377243	0,00846618	0,00669282	0,00796494	0,01025686	0,0123977	0,00328739	0,00664504	0,00935984	0,00552896
	F4	0,0044317	0,00465022	0,00383821	0,00303413	0,00478801	0,00876609	0,00734348	0,00784729	0,01070369	0,01180172	0,01162911	0,0043046	0,00383993	0,00891231	0,00694464
	F8	0,01245569	0,00661132	0,00702895	0,01290539	0,00924012	0,01431214	0,00607668	0,01629338	0,01833772	0,00840758	0,01446815	0,01129941	0,00832242	0,01990749	0,01501506
	FC5	0,01626516	0,01100242	0,01238734	0,01518322	0,01420183	0,01919518	0,00999018	0,02346984	0,02197815	0,01732934	0,02212815	0,01550928	0,01079848	0,02369867	0,024587
	FC1	0,00811554	0,00538624	0,00667873	0,00911502	0,00830663	0,01166662	0,01309055	0,01547084	0,0049374	0,0148885	0,00712318	0,0035729	0,00549341	0,00495715	0,00188182
	FC2	0,00476502	0,00833245	0,00713734	0,00813448	0,00862928	0,01292297	0,00200651	0,00914128	0,01383106	0,00830096	0,01507204	0,00885253	0,01077446	0,01439807	0,01121889
	FC6	0,01100531	0,00632613	0,01121008	0,01555046	0,00852502	0,01025436	0,00598004	0,02321645	0,02231255	0,01511502	0,01574757	0,01527939	0,01132036	0,01966916	0,01851463
	T7	0,01827894	0,01121218	0,01517079	0,01491156	0,01404725	0,02090093	0,01199505	0,02379538	0,02165173	0,02040242	0,02169007	0,01239372	0,00858121	0,01974616	0,02048207
	C3	0,01061872	0,00848069	0,00745617	0,0093457	0,01236242	0,01740236	0,00930085	0,01319614	0,00515421	0,01211005	0,01353821	0,00516456	0,0102806	0,00579665	0,00547265
	Cz	0,00891274	0,01024238	0,00762886	0,00835657	0,00560035	0,01650586	0,00543593	0,01052762	0,01574398	0,00954625	0,01568707	0,0150865	0,00874067	0,01561645	0,01136743
	C4	0,01009246	0,01273624	0,01469392	0,00883796	0,01821249	0,01547161	0,00869616	0,02081759	0,01444879	0,00867132	0,01757641	0,01475093	0,00898007	0,01520333	0,01290276
	T8	0,00994283	0,00424548	0,00739016	0,00388616	0,00545702	0,01090556	0,00610561	0,00891887	0,01259164	0,00530977	0,01054312	0,00828136	0,00833415	0,01091361	0,00785916
	CP5	0,01365457	0,01351169	0,00885768	0,00718754	0,01336228	0,01996523	0,00695686	0,02048792	0,01063148	0,01265993	0,01964889	0,01132412	0,01146702	0,01548946	0,0149253
	CP1	0,01314544	0,01712396	0,00878506	0,0118072	0,00202395	0,00986137	0,00894767	0,01408849	0,01165551	0,00915724	0,00659701	0,00991392	0,01247511	0,01985535	0,00428635
	CP2	0,01295783	0,01321526	0,01198244	0,01523698	0,01717072	0,01784051	0,01027799	0,01652781	0,01610477	0,01519666	0,018134	0,01918016	0,0110407	0,01932252	0,02138951
	CP6	0,01611808	0,01164695	0,01498022	0,01901583	0,01694612	0,01923549	0,01231194	0,02770133	0,0195273	0,01494872	0,01986311	0,01791825	0,0142614	0,02198704	0,01798717
	P7	0,01138897	0,01111125	0,00872292	0,00711142	0,00880097	0,02011266	0,01121725	0,02017248	0,00381047	0,00854607	0,01598404	0,00686993	0,01087541	0,01167241	0,00793368
	P3	0,00860887	0,00848401	0,00727554	0,00911205	0,01133835	0,01758425	0,00771988	0,01880688	0,0055708	0,00871549	0,0135754	0,00910746	0,00990012	0,01094172	0,00792124
	Pz	0,02038271	0,01091132	0,011055	0,01216079	0,01651453	0,0235082	0,00992094	0,02210213	0,01853986	0,02337083	0,02440546	0,01197464	0,00776533	0,01941537	0,02048908
	P4	0,01532889	0,01090957	0,01006719	0,01891417	0,01073345	0,01313847	0,00986242	0,02444387	0,01737097	0,01186017	0,01612222	0,01898513	0,01565159	0,01871685	0,01737304
	P8	0,01346205	0,01293533	0,00834337	0,01724334	0,0082242	0,01310928	0,00996703	0,01895612	0,01620992	0,00624998	0,01604083	0,01735886	0,01497944	0,01675607	0,01904919
	POz	0,01267238	0,00997535	0,01216901	0,00900276	0,01359381	0,02216922	0,00979547	0,01970902	0,01452396	0,01576446	0,0188407	0,0078552	0,00757343	0,01604154	0,01889835
	O1	0,01488253	0,01371152	0,00831546	0,01750043	0,00797977	0,01120323	0,00890875	0,02196306	0,0134862	0,00751997	0,01831806	0,01571114	0,01785705	0,02042236	0,01580343
	O2	0,01315699	0,00536019	0,00744075	0,00687648	0,01156422	0,01743371	0,00587962	0,01662532	0,01374607	0,01234282	0,01768057	0,00760296	0,00343868	0,01487795	0,01441698

		subject 1													
		C4	T8	CP5	CP1	CP2	CP6	P7	P3	Pz	P4	P8	POz	O1	O2
subject 1	Fp1	0,01219253	0,04236465	0,0048027	0,02219273	0,01908637	0,04013816	0,01550572	0,04529791	0,03730357	0,02201235	0,04888394	0,02806969	0,03106003	0,03984499
	Fpz	0,0227577	0,01686437	0,00566654	0,01524668	0,018021	0,02367179	0,00736924	0,0345204	0,02318547	0,01904777	0,0303343	0,03030002	0,03587527	0,03448826
	Fp2	0,02544307	0,0413913	0,00601529	0,03668619	0,02681292	0,04492807	0,02365376	0,04406697	0,02410541	0,03161456	0,04800209	0,03426728	0,02875725	0,04778926
	F7	0,05312251	0,02311194	0,024257	0,04048939	0,03151441	0,03092463	0,01615821	0,01093761	0,00491978	0,04433651	0,03446546	0,03362038	0,03527772	0,02242042
	F3	0,03060491	0,02639183	0,03746635	0,03073654	0,02437387	0,02932833	0,00725403	0,017575	0,01798031	0,0270453	0,05227403	0,02595649	0,02658943	0,03593295
	Fz	0,03236352	0,01501657	0,01014468	0,01123106	0,03870973	0,01205074	0,01200611	0,03725667	0,03424042	0,00758813	0,01222763	0,0515513	0,02336468	0,03618445
	F4	0,01412167	0,02602174	0,00639976	0,01306693	0,01528853	0,02436393	0,01706862	0,03935439	0,02445546	0,01790372	0,0311297	0,03736556	0,02830456	0,0373889
	F8	0,07676233	0,10111536	0,05953446	0,07137118	0,05556778	0,05926828	0,03429392	0,02650577	0,01982322	0,08355903	0,06691856	0,04703841	0,01293907	0,02047286
	FC5	0,02462406	0,00493257	0,03158542	0,01774926	0,01786798	0,02397248	0,05544091	0,02281669	0,02927382	0,02551265	0,02420407	0,01089295	0,01432123	0,02497966
	FC1	0,04218619	0,09864543	0,01785268	0,05031986	0,05900951	0,06275283	0,0216528	0,0369178	0,02897752	0,0364356	0,06694568	0,02389548	0,03265113	0,03586516
	FC2	0,0209103	0,03810648	0,02926423	0,04153545	0,02212456	0,02258002	0,01549081	0,0172692	0,02996034	0,05472728	0,03010656	0,03174524	0,04936868	0,02764467
	FC6	0,0436258	0,03899738	0,02366416	0,04305094	0,04026491	0,02453085	0,00923585	0,01930557	0,01503523	0,05045313	0,03084879	0,04371221	0,04350429	0,02894689
	T7	0,02585927	0,02549275	0,01278077	0,01807667	0,03205358	0,01847532	0,00228975	0,02059004	0,01544644	0,01441932	0,01829336	0,03984802	0,03848647	0,02113966
	C3	0,02832352	0,01153228	0,01343725	0,02056909	0,0130871	0,01800964	0,0416557	0,02714196	0,0062341	0,01887136	0,03003468	0,00627816	0,01389118	0,01988765
	Cz	0,03744761	0,05015578	0,02022267	0,05726948	0,04082264	0,02937186	0,00878573	0,023563	0,0172713	0,06031052	0,03420069	0,03673302	0,03869482	0,02774371
	C4	0	0,03215458	0,00713392	0,06177503	0,02228824	0,04646214	0,01987727	0,01498338	0,02916736	0,07135993	0,04912511	0,02042992	0,02541352	0,02440875
	T8	0,03215458	0	0,02257049	0,11330336	0,0577176	0,06091776	0,02996732	0,02654399	0,03800995	0,10475134	0,06754656	0,0421497	0,00533365	0,02051246
	CP5	0,00713392	0,02257049	0	0,01159849	0,01679455	0,024684	0,01259128	0,03633921	0,024381	0,01516139	0,03057272	0,02613839	0,03828068	0,04001684
	CP1	0,06177503	0,11330336	0,01159849	0	0,06122149	0,03306487	0,01721547	0,01355796	0,00920286	0,03953932	0,0257736	0,04590609	0,02083034	0,01724585
	CP2	0,02228824	0,0577176	0,01679455	0,06122149	0	0,0296233	0,03230576	0,02746455	0,02438503	0,0607229	0,04554293	0,00488308	0,04071862	0,04282943
	CP6	0,04646214	0,06091776	0,024684	0,03306487	0,0296233	0	0,01612534	0,02679591	0,00610168	0,02835377	0,03521645	0,02865412	0,02321283	0,0420676
	P7	0,01987727	0,02996732	0,01259128	0,01721547	0,03230576	0,01612534	0	0,02434967	0,01709592	0,01338875	0,01615543	0,03637002	0,04552421	0,0203064
	P3	0,01498338	0,02654399	0,03633921	0,01355796	0,02746455	0,02679591	0,02434967	0	0,02011804	0,01627905	0,02697662	0,02570639	0,03090766	0,03447884
	Pz	0,02916736	0,03800995	0,024381	0,00920286	0,02438503	0,00610168	0,01709592	0,02011804	0	0,01912288	0,01694639	0,03988386	0,02392091	0,02601872
	P4	0,07135993	0,10475134	0,01516139	0,03953932	0,0607229	0,02835377	0,01338875	0,01627905	0,01912288	0	0,04936768	0,04525545	0,01943965	0,02492648
	P8	0,04912511	0,06754656	0,03057272	0,0257736	0,04554293	0,03521645	0,01615543	0,02697662	0,01694639	0,04936768	0	0,03854018	0,00573044	0,01887373
	POz	0,02042992	0,0421497	0,02613839	0,04590609	0,00488308	0,02865412	0,03637002	0,02570639	0,03988386	0,04525545	0,03854018	0	0,0451371	0,03048893
	O1	0,02541352	0,00533365	0,03828068	0,02083034	0,04071862	0,02321283	0,04552421	0,03090766	0,02392091	0,01943965	0,00573044	0,0451371	0	0,0229943
	O2	0,02440875	0,02051246	0,04001684	0,01724585	0,04282943	0,0420676	0,0203064	0,03447884	0,02601872	0,02492648	0,01887373	0,03048893	0,0229943	0
subject 2	Fp1	0,00741647	0,00701287	0,01786071	0,02896357	0,02317855	0,0298968	0,00970971	0,0182486	0,01237525	0,02112415	0,02494852	0,0211174	0,02083162	0,02205863
	Fpz	0,00483142	0,00571894	0,01555483	0,02741859	0,01962756	0,02608519	0,00852548	0,01318512	0,01150353	0,01846367	0,02862739	0,01637193	0,01691293	0,01941215
	Fp2	0,01094744	0,0054557	0,01347617	0,03035251	0,01352096	0,02478265	0,00976812	0,0092071	0,00834848	0,01385601	0,02629462	0,00726134	0,01472758	0,01476337
	F7	0,00610394	0,00802852	0,01748037	0,02958775	0,02104257	0,02465692	0,01110922	0,01266042	0,01073667	0,01651994	0,03013312	0,01897811	0,0202659	0,02196817
	F3	0,01656598	0,00923724	0,01935314	0,02498883	0,02024381	0,02781412	0,01011721	0,01666001	0,00942629	0,02118861	0,02794748	0,02268994	0,02043888	0,02075572
	Fz	0,0141745	0,0075214	0,00416031	0,02628052	0,01227801	0,02356692	0,00498565	0,00818944	0,01568898	0,01635326	0,01608487	0,00405636	0,00986055	0,01532199
	F4	0,0147833	0,00788148	0,00335199	0,02785783	0,01475679	0,02383265	0,00295361	0,01231952	0,0181642	0,01442194	0,02172361	0,00596333	0,01023104	0,01478179
	F8	0,02104803	0,01648599	0,00782211	0,02796528	0,01628793	0,0193765	0,01047294	0,00569172	0,02247454	0,01966141	0,02411167	0,01157836	0,00769302	0,0070642
	FC5	0,01600882	0,0218239	0,01241301	0,02772485	0,01919229	0,02868046	0,00891834	0,00717625	0,01931335	0,02388708	0,02389893	0,0112647	0,00745631	0,00662223
	FC1	0,01675182	0,00532238	0,00625889	0,02470454	0,01516603	0,0201555	0,0088977	0,01351737	0,01764625	0,01441752	0,02104724	0,00994542	0,01295269	0,01815264
	FC2	0,01002212	0,01194551	0,00691659	0,02670952	0,01331911	0,02292803	0,00669216	0,01054828	0,01896776	0,01627192	0,02208125	0,00594204	0,00325492	0,01096323
	FC6	0,01777007	0,01967131	0,0088894	0,02598784	0,02046438	0,02543291	0,01070117	0,00579581	0,02611555	0,02687988	0,02384582	0,01227525	0,00891133	0,00703381
	T7	0,01600081	0,02366128	0,01080933	0,02651226	0,02176977	0,02921267	0,00767345	0,00415317	0,0228375	0,02341949	0,02398097	0,01613857	0,00865602	0,00912448
	C3	0,01787017	0,0082586	0,00819435	0,02494429	0,01342305	0,02243906	0,00869672	0,00532126	0,01745351	0,01574906	0,01704182	0,00907535	0,01212564	0,01556194
	Cz	0,01186729	0,01600528	0,00771287	0,02654694	0,01417236	0,02138425	0,00651077	0,01034998	0,01827877	0,01679205	0,01955355	0,00708668	0,00831166	0,01277676
	C4	0,01247774	0,0067198	0,01207671	0,01804204	0,0124058	0,01911561	0,00538595	0,00593296	0,01481608	0,01054614	0,0113181	0,01106006	0,00709639	0,00742299
	T8	0,01706444	0,00751991	0,01140714	0,02524951	0,0109523	0,01453434	0,00820934	0,01206895	0,01995213	0,01233067	0,01859601	0,00413738	0,01442129	0,01138323
	CP5	0,0165935	0,01213837	0,00979652	0,02068537	0,01539905	0,01725037	0,00914766	0,00484556	0,01711243	0,01948185	0,01325938	0,01095232	0,00711041	0,006405
	CP1	0,01964954	0,00836062	0,01265809	0,02110187	0,0094944	0,01519308	0,01528525	0,00943292	0,01258159	0,01331964	0,01512954	0,00758786	0,00749661	0,0101569
	CP2	0,0105792	0,01781423	0,01397926	0,02734666	0,01800502	0,02433536	0,01265681	0,00949887	0,01486613	0,02172549	0,02621427	0,01004998	0,00502126	0,00906448
	CP6	0,0186349	0,01663082	0,01463612	0,02543449	0,01785935	0,02342704	0,01445027	0,00559644	0,0156107	0,02077662	0,02586217	0,01347955	0,00771228	0,00717241
	P7	0,01545659	0,00872496	0,00884928	0,01547525	0,0167337	0,01606981	0,01212066	0,01168945	0,01791362	0,01326084	0,01166784	0,0123565	0,00916116	0,00850287
	P3	0,01527183	0,0026526	0,00549875	0,01716038	0,01494388	0,01508767	0,01134625	0,01096019	0,01432314	0,01330664	0,00881804	0,01064336	0,01025272	0,00866891
	Pz	0,02046746	0,02270893	0,01476244	0,02930963	0,01826311	0,02843566	0,00646118	0,00761378	0,01770001	0,02763294	0,02755341	0,01748105	0,00944677	0,00258255
	P4	0,01991059	0,01786773	0,01344105	0,02381786	0,02076804	0,02425873	0,01517283	0,00717239	0,01226643	0,02206464	0,02369147	0,01165318	0,0052163	0,00553575
	P8	0,0180627	0,0127294	0,01468424	0,02662067	0,0184247	0,01967931	0,0133303	0,00736932	0,01113118	0,01761317	0,02015513	0,01010771	0,00541131	0,00412684
	POz	0,01857413	0,01575262	0,01026195	0,02343695	0,01357054	0,01844736	0,00570612	0,00629569	0,01664512	0,01667444	0,01638548	0,01159147	0,00980668	0,00535502
	O1	0,02046932	0,00721366	0,01535349	0,02448071	0,02027043	0,01701715	0,01374311	0,0079566	0,0128615	0,01575796	0,01958456	0,01274238	0,0070126	0,00467354
	O2	0,01966004	0,01235211	0,0056573	0,02003633	0,0137558	0,02087123	0,00307025	0,00218798	0,01498184	0,01514834	0,01895725	0,00930966	0,00870332	0,00730575

		subject 2														
		Fp1	Fpz	Fp2	F7	F3	Fz	F4	F8	FC5	FC1	FC2	FC6	T7	C3	Cz
subject 1	Fp1	0,01178248	0,00787339	0,00668469	0,01036068	0,0134002	0,0073715	0,0044317	0,01245569	0,01626516	0,00811554	0,00476502	0,01100531	0,01827894	0,01061872	0,00891274
	Fpz	0,01129309	0,00808894	0,00690453	0,01160361	0,00670195	0,01038896	0,00465022	0,00661132	0,01100242	0,00538624	0,00833245	0,00632613	0,01121218	0,00848069	0,01024238
	Fp2	0,01375432	0,01436217	0,0090756	0,0128837	0,01062935	0,00409787	0,00383821	0,00702895	0,01238734	0,00667873	0,00713734	0,01121008	0,01517079	0,00745617	0,00762886
	F7	0,00835619	0,00760679	0,00791617	0,00899498	0,01225011	0,00767693	0,00303413	0,01290539	0,01518322	0,00911502	0,00813448	0,01555046	0,01491156	0,0093457	0,00835657
	F3	0,01158885	0,00645808	0,00187208	0,00895302	0,01355351	0,0036061	0,00478801	0,00924012	0,01420183	0,00830663	0,00862928	0,00852502	0,01404725	0,01236242	0,00560035
	Fz	0,0081875	0,00851609	0,01208763	0,00942953	0,00852535	0,01377243	0,00876609	0,01431214	0,01919518	0,01166662	0,01292297	0,01025436	0,02090093	0,01740236	0,01650586
	F4	0,01412929	0,01492992	0,01091843	0,01446838	0,01959158	0,00846618	0,00734348	0,00607668	0,00999018	0,01309055	0,00200651	0,00598004	0,01199505	0,00930085	0,00543593
	F8	0,00709347	0,00543985	0,0046338	0,00565019	0,0194419	0,00669282	0,00784729	0,01629338	0,02346984	0,01547084	0,00914128	0,02321645	0,02379538	0,01319614	0,01052762
	FC5	0,00972517	0,00715263	0,00612373	0,00923706	0,012484	0,00796494	0,01070369	0,01833772	0,02197815	0,0049374	0,01383106	0,02231255	0,02165173	0,00515421	0,01574398
	FC1	0,02147558	0,01920362	0,01058052	0,02051089	0,02093578	0,01025686	0,01180172	0,00840758	0,01732934	0,0148885	0,00830096	0,01511502	0,02040242	0,01211005	0,00954625
	FC2	0,00495943	0,00302618	0,00562897	0,00283191	0,00400018	0,0123977	0,01162911	0,01446815	0,02212815	0,00712318	0,01507204	0,01574757	0,02169007	0,01353821	0,01568707
	FC6	0,00696729	0,00504561	0,00733916	0,00606514	0,00567883	0,00328739	0,0043046	0,01129941	0,01550928	0,0035729	0,00885253	0,01527939	0,01239372	0,00516456	0,0150865
	T7	0,00728695	0,00786416	0,00983181	0,00857021	0,01112651	0,00664504	0,00383993	0,00832242	0,01079848	0,00549341	0,01077446	0,01132036	0,00858121	0,0102806	0,00874067
	C3	0,01308567	0,00856407	0,00335943	0,01080119	0,01090503	0,00935984	0,00891231	0,01990749	0,02369867	0,00495715	0,01439807	0,01966916	0,01974616	0,00579665	0,01561645
	Cz	0,01195934	0,00947086	0,00193432	0,01027467	0,0077509	0,00552896	0,00694464	0,01501506	0,024587	0,00188182	0,01121889	0,01851463	0,02048207	0,00547265	0,01136743
	C4	0,00741647	0,00483142	0,01094744	0,00610394	0,01656598	0,0141745	0,0147833	0,02104803	0,01600882	0,01675182	0,01002212	0,01777007	0,01600081	0,01787017	0,01186729
	T8	0,00701287	0,00571894	0,0054557	0,00802852	0,00923724	0,0075214	0,00788148	0,01648599	0,0218239	0,00532238	0,01194551	0,01967131	0,02366128	0,0082586	0,01600528
	CP5	0,01786071	0,01555483	0,01347617	0,01748037	0,01935314	0,00416031	0,00335199	0,00782211	0,01241301	0,00625889	0,00691659	0,0088894	0,01080933	0,00819435	0,00771287
	CP1	0,02896357	0,02741859	0,03035251	0,02958775	0,02498883	0,02628052	0,02785783	0,02796528	0,02772485	0,02470454	0,02670952	0,02598784	0,02651226	0,02494429	0,02654694
	CP2	0,02317855	0,01962756	0,01352096	0,02104257	0,02024381	0,01227801	0,01475679	0,01628793	0,01919229	0,01516603	0,01331911	0,02046438	0,02176977	0,01342305	0,01417236
	CP6	0,0298968	0,02608519	0,02478265	0,02465692	0,02781412	0,02356692	0,02383265	0,0193765	0,02868046	0,0201555	0,02292803	0,02543291	0,02921267	0,02243906	0,02138425
	P7	0,00970971	0,00852548	0,00976812	0,01110922	0,01011721	0,00498565	0,00295361	0,01047294	0,00891834	0,0088977	0,00669216	0,01070117	0,00767345	0,00869672	0,00651077
	P3	0,0182486	0,01318512	0,0092071	0,01266042	0,01666001	0,00818944	0,01231952	0,00569172	0,00717625	0,01351737	0,01054828	0,00579581	0,00415317	0,00532126	0,01034998
	Pz	0,01237525	0,01150353	0,00834848	0,01073667	0,00942629	0,01568898	0,0181642	0,02247454	0,01931335	0,01764625	0,01896776	0,02611555	0,0228375	0,01745351	0,01827877
	P4	0,02112415	0,01846367	0,01385601	0,01651994	0,02118861	0,01635326	0,01442194	0,01966141	0,02388708	0,01441752	0,01627192	0,02687988	0,02341949	0,01574906	0,01679205
	P8	0,02494852	0,02862739	0,02629462	0,03013312	0,02794748	0,01608487	0,02172361	0,02411167	0,02389893	0,02104724	0,02208125	0,02384582	0,02398097	0,01704182	0,01955355
	POz	0,0211174	0,01637193	0,00726134	0,01897811	0,02268994	0,00405636	0,00596333	0,01157836	0,0112647	0,00994542	0,00594204	0,01227525	0,01613857	0,00907535	0,00708668
	O1	0,02083162	0,01691293	0,01472758	0,0202659	0,02043888	0,00986055	0,01023104	0,00769302	0,00745631	0,01295269	0,00325492	0,00891133	0,00865602	0,01212564	0,00831166
	O2	0,02205863	0,01941215	0,01476337	0,02196817	0,02075572	0,01532199	0,01478179	0,0070642	0,00662223	0,01815264	0,01096323	0,00703381	0,00912448	0,01556194	0,01277676
subject 2	Fp1	0	0,00359641	0,01076878	0,00231925	0,0611698	0,04758239	0,04078277	0,04719281	0,08167782	0,04144437	0,06782534	0,07261249	0,07066186	0,03964127	0,07059846
	Fpz	0,00359641	0	0,00334316	0,00161049	0,05065967	0,05919288	0,05338456	0,03674041	0,09316205	0,04571585	0,07924627	0,0694841	0,07386171	0,04365146	0,08247382
	Fp2	0,01076878	0,00334316	0	0,00532498	0,03571078	0,0677214	0,05879939	0,02542336	0,06959616	0,05298009	0,07452271	0,05809459	0,05428632	0,05056926	0,08154193
	F7	0,00231925	0,00161049	0,00532498	0	0,05284059	0,05201003	0,04388067	0,04191602	0,08323068	0,04168542	0,07126047	0,07008671	0,07332165	0,04334445	0,07925044
	F3	0,0611698	0,05065967	0,03571078	0,05284059	0	0,00701977	0,00760339	0,06386973	0,09798268	0,00745712	0,02507081	0,08279167	0,09351529	0,01696546	0,03133165
	Fz	0,04758239	0,05919288	0,0677214	0,05201003	0,00701977	0	0,00800842	0,07874505	0,07070714	0,00365664	0,0143181	0,07155559	0,072042	0,01925069	0,00821486
	F4	0,04078277	0,05338456	0,05879939	0,04388067	0,00760339	0,00800842	0	0,08453809	0,06580954	0,00757869	0,00868081	0,07082545	0,06717689	0,03774355	0,00925413
	F8	0,04719281	0,03674041	0,02542336	0,04191602	0,06386973	0,07874505	0,08453809	0	0,01911769	0,09137344	0,06329973	0,00948484	0,01679975	0,07115242	0,06010897
	FC5	0,08167782	0,09316205	0,06959616	0,08323068	0,09798268	0,07070714	0,06580954	0,01911769	0	0,09321756	0,03068555	0,01398331	0,00279945	0,07648297	0,03378336
	FC1	0,04144437	0,04571585	0,05298009	0,04168542	0,00745712	0,00365664	0,00757869	0,09137344	0,09321756	0	0,02358625	0,08252047	0,09586341	0,01890664	0,02509192
	FC2	0,06782534	0,07924627	0,07452271	0,07126047	0,02507081	0,0143181	0,00868081	0,06329973	0,03068555	0,02358625	0	0,05793171	0,03891244	0,03479243	0,00889688
	FC6	0,07261249	0,0694841	0,05809459	0,07008671	0,08279167	0,07155559	0,07082545	0,00948484	0,01398331	0,08252047	0,05793171	0	0,0140672	0,06680003	0,04384422
	T7	0,07066186	0,07386171	0,05428632	0,07332165	0,09351529	0,072042	0,06717689	0,01679975	0,00279945	0,09586341	0,03891244	0,0140672	0	0,09218946	0,0347095
	C3	0,03964127	0,04365146	0,05056926	0,04334445	0,01696546	0,01925069	0,03774355	0,07115242	0,07648297	0,01890664	0,03479243	0,06680003	0,09218946	0	0,02176997
	Cz	0,07059846	0,08247382	0,08154193	0,07925044	0,03133165	0,00821486	0,00925413	0,06010897	0,03378336	0,02509192	0,00889688	0,04384422	0,0347095	0,02176997	0
	C4	0,06450374	0,06571194	0,06838466	0,06492048	0,05161654	0,04960888	0,05182864	0,05535457	0,05624821	0,05515887	0,03474616	0,0456866	0,04366882	0,02928052	0,05258352
	T8	0,01999078	0,01837191	0,01703172	0,02102265	0,03309538	0,06293128	0,0623175	0,00627266	0,03006774	0,05604471	0,07099895	0,02841387	0,02639474	0,05917141	0,06631758
	CP5	0,05902872	0,05621378	0,04234214	0,07050322	0,07109729	0,04650102	0,03973203	0,02632246	0,0215257	0,0681379	0,03006165	0,02697021	0,01230013	0,07222242	0,02545079
	CP1	0,07538762	0,07297473	0,07861092	0,07440591	0,05848064	0,00957906	0,01652979	0,04796854	0,02195636	0,02504739	0,04097819	0,03973988	0,02125048	0,03837835	0,03732674
	CP2	0,08837018	0,0846503	0,07318115	0,0876032	0,07750022	0,05181676	0,06510537	0,05272054	0,02711666	0,07918236	0,0319284	0,06233261	0,03470182	0,03654428	0,02163851
	CP6	0,06011505	0,05290982	0,04514626	0,05838482	0,06666085	0,06215088	0,07526502	0,06182343	0,03653777	0,07597637	0,04979348	0,06792069	0,04398248	0,03304631	0,034436
	P7	0,04064995	0,03649174	0,03266468	0,04315111	0,05328074	0,04020599	0,03121526	0,02673641	0,02799991	0,05586755	0,0260491	0,02497133	0,02186818	0,06903145	0,02284531
	P3	0,04190745	0,03531005	0,03006498	0,04032641	0,04849016	0,03121601	0,03102461	0,02108058	0,03129692	0,05143345	0,02660301	0,02422171	0,02442792	0,0594789	0,02573084
	Pz	0,0732336	0,06589462	0,05146315	0,06849063	0,08624732	0,06456101	0,06681381	0,02772676	0,00665382	0,0948758	0,03175018	0,02463758	0,00700546	0,07948938	0,03948249
	P4	0,04826097	0,04239535	0,02956795	0,04348651	0,05887475	0,06808113	0,07564939	0,04412715	0,02233414	0,07532842	0,05512219	0,04874243	0,02914688	0,04230809	0,0502175
	P8	0,04667581	0,0392983	0,02731611	0,03643915	0,05140309	0,07295772	0,08137771	0,00858438	0,01927747	0,07635655	0,05870294	0,01281295	0,02170711	0,05812661	0,05687777
	POz	0,03766888	0,02959944	0,0202195	0,03493472	0,05794244	0,0737575	0,05902602	0,02579128	0,01203343	0,08558274	0,04005658	0,02110125	0,00578846	0,09500537	0,04298496
	O1	0,04243256	0,03436767	0,03061455	0,04382047	0,05966064	0,06751005	0,07362689	0,00460085	0,02238226	0,07908886	0,05591673	0,00578306	0,02648315	0,05510072	0,04981483
	O2	0,02779594	0,02027725	0,01095857	0,02549821	0,0448339	0,07660406	0,07162825	0,02143294	0,02592114	0,0775724	0,05009899	0,0237551	0,01717529	0,09206702	0,06078607

		subject 2													
		C4	T8	CP5	CP1	CP2	CP6	P7	P3	Pz	P4	P8	POz	O1	O2
subject 1	Fp1	0,01009246	0,00994283	0,01365457	0,01314544	0,01295783	0,01611808	0,01138897	0,00860887	0,02038271	0,01532889	0,01346205	0,01267238	0,01488253	0,01315699
	Fpz	0,01273624	0,00424548	0,01351169	0,01712396	0,01321526	0,01164695	0,01111125	0,00848401	0,01091132	0,01090957	0,01293533	0,00997535	0,01371152	0,00536019
	Fp2	0,01469392	0,00739016	0,00885768	0,00878506	0,01198244	0,01498022	0,00872292	0,00727554	0,011055	0,01006719	0,00834337	0,01216901	0,00831546	0,00744075
	F7	0,00883796	0,00388616	0,00718754	0,0118072	0,01523698	0,01901583	0,00711142	0,00911205	0,01216079	0,01891417	0,01724334	0,00900276	0,01750043	0,00687648
	F3	0,01821249	0,00545702	0,01336228	0,00202395	0,01717072	0,01694612	0,00880097	0,01133835	0,01651453	0,01073345	0,0082242	0,01359381	0,00797977	0,01156422
	Fz	0,01547161	0,01090556	0,01996523	0,00986137	0,01784051	0,01923549	0,02011266	0,01758425	0,0235082	0,01313847	0,01310928	0,02216922	0,01120323	0,01743371
	F4	0,00869616	0,00610561	0,00695686	0,00894767	0,01027799	0,01231194	0,01121725	0,00771988	0,00992094	0,00986242	0,00996703	0,00979547	0,00890875	0,00587962
	F8	0,02081759	0,00891887	0,02048792	0,01408849	0,01652781	0,02770133	0,02017248	0,01880688	0,02210213	0,02444387	0,01895612	0,01970902	0,02196306	0,01662532
	FC5	0,01444879	0,01259164	0,01063148	0,01165551	0,01610477	0,0195273	0,00381047	0,0055708	0,01853986	0,01737097	0,01620992	0,01452396	0,0134862	0,01374607
	FC1	0,00867132	0,00530977	0,01265993	0,00915724	0,01519666	0,01494872	0,00854607	0,00871549	0,02337083	0,01186017	0,00624998	0,01576446	0,00751997	0,01234282
	FC2	0,01757641	0,01054312	0,01964889	0,00659701	0,018134	0,01986311	0,01598404	0,0135754	0,02440546	0,01612222	0,01604083	0,0188407	0,01831806	0,01768057
	FC6	0,01475093	0,00828136	0,01132412	0,00991392	0,01918016	0,01791825	0,00686993	0,00910746	0,01197464	0,01898513	0,01735886	0,0078552	0,01571114	0,00760296
	T7	0,00898007	0,00833415	0,01146702	0,01247511	0,0110407	0,0142614	0,01087541	0,00990012	0,00776533	0,01565159	0,01497944	0,00757343	0,01785705	0,00343868
	C3	0,01520333	0,01091361	0,01548946	0,01985535	0,01932252	0,02198704	0,01167241	0,01094172	0,01941537	0,01871685	0,01675607	0,01604154	0,02042236	0,01487795
	Cz	0,01290276	0,00785916	0,0149253	0,00428635	0,02138951	0,01798717	0,00793368	0,00792124	0,02048908	0,01737304	0,01904919	0,01889835	0,01580343	0,01441698
	C4	0,01247774	0,01706444	0,0165935	0,01964954	0,0105792	0,0186349	0,01545659	0,01527183	0,02046746	0,01991059	0,0180627	0,01857413	0,02046932	0,01966004
	T8	0,0067198	0,00751991	0,01213837	0,00836062	0,01781423	0,01663082	0,00872496	0,0026526	0,02270893	0,01786773	0,0127294	0,01575262	0,00721366	0,01235211
	CP5	0,01207671	0,01140714	0,00979652	0,01265809	0,01397926	0,01463612	0,00884928	0,00549875	0,01476244	0,01344105	0,01468424	0,01026195	0,01535349	0,0056573
	CP1	0,01804204	0,02524951	0,02068537	0,02110187	0,02734666	0,02543449	0,01547525	0,01716038	0,02930963	0,02381786	0,02662067	0,02343695	0,02448071	0,02003633
	CP2	0,0124058	0,0109523	0,01539905	0,0094944	0,01800502	0,01785935	0,0167337	0,01494388	0,01826311	0,02076804	0,0184247	0,01357054	0,02027043	0,0137558
	CP6	0,01911561	0,01453434	0,01725037	0,01519308	0,02433536	0,02342704	0,01606981	0,01508767	0,02843566	0,02425873	0,01967931	0,01844736	0,01701715	0,02087123
	P7	0,00538595	0,00820934	0,00914766	0,01528525	0,01265681	0,01445027	0,01212066	0,01134625	0,00646118	0,01517283	0,0133303	0,00570612	0,01374311	0,00307025
	P3	0,00593296	0,01206895	0,00484556	0,00943292	0,00949887	0,00559644	0,01168945	0,01096019	0,00761378	0,00717239	0,00736932	0,00629569	0,0079566	0,00218798
	Pz	0,01481608	0,01995213	0,01711243	0,01258159	0,01486613	0,0156107	0,01791362	0,01432314	0,01770001	0,01226643	0,01113118	0,01664512	0,0128615	0,01498184
	P4	0,01054614	0,01233067	0,01948185	0,01331964	0,02172549	0,02077662	0,01326084	0,01330664	0,02763294	0,02206464	0,01761317	0,01667444	0,01575796	0,01514834
	P8	0,0113181	0,01859601	0,01325938	0,01512954	0,02621427	0,02586217	0,01166784	0,00881804	0,02755341	0,02369147	0,02015513	0,01638548	0,01958456	0,01895725
	POz	0,01106006	0,00413738	0,01095232	0,00758786	0,01004998	0,01347955	0,0123565	0,01064336	0,01748105	0,01165318	0,01010771	0,01159147	0,01274238	0,00930966
	O1	0,00709639	0,01442129	0,00711041	0,00749661	0,00502126	0,00771228	0,00916116	0,01025272	0,00944677	0,0052163	0,00541131	0,00980668	0,0070126	0,00870332
	O2	0,00742299	0,01138323	0,006405	0,0101569	0,00906448	0,00717241	0,00850287	0,00866891	0,00258255	0,00553575	0,00412684	0,00535502	0,00467354	0,00730575
subject 2	Fp1	0,06450374	0,01999078	0,05902872	0,07538762	0,08837018	0,06011505	0,04064995	0,04190745	0,0732336	0,04826097	0,04667581	0,03766888	0,04243256	0,02779594
	Fpz	0,06571194	0,01837191	0,05621378	0,07297473	0,0846503	0,05290982	0,03649174	0,03531005	0,06589462	0,04239535	0,0392983	0,02959944	0,03436767	0,02027725
	Fp2	0,06838466	0,01703172	0,04234214	0,07861092	0,07318115	0,04514626	0,03266468	0,03006498	0,05146315	0,02956795	0,02731611	0,0202195	0,03061455	0,01095857
	F7	0,06492048	0,02102265	0,07050322	0,07440591	0,0876032	0,05838482	0,04315111	0,04032641	0,06849063	0,04348651	0,03643915	0,03493472	0,04382047	0,02549821
	F3	0,05161654	0,03309538	0,07109729	0,05848064	0,07750022	0,06666085	0,05328074	0,04849016	0,08624732	0,05887475	0,05140309	0,05794244	0,05966064	0,0448339
	Fz	0,04960888	0,06293128	0,04650102	0,00957906	0,05181676	0,06215088	0,04020599	0,03121601	0,06456101	0,06808113	0,07295772	0,0737575	0,06751005	0,07660406
	F4	0,05182864	0,0623175	0,03973203	0,01652979	0,06510537	0,07526502	0,03121526	0,03102461	0,06681381	0,07564939	0,08137771	0,05902602	0,07362689	0,07162825
	F8	0,05535457	0,00627266	0,02632246	0,04796854	0,05272054	0,06182343	0,02673641	0,02108058	0,02772676	0,04412715	0,00858438	0,02579128	0,00460085	0,02143294
	FC5	0,05624821	0,03006774	0,0215257	0,02195636	0,02711666	0,03653777	0,02799991	0,03129692	0,00665382	0,02233414	0,01927747	0,01203343	0,02238226	0,02592114
	FC1	0,05515887	0,05604471	0,0681379	0,02504739	0,07918236	0,07597637	0,05586755	0,05143345	0,0948758	0,07532842	0,07635655	0,08558274	0,07908886	0,0775724
	FC2	0,03474616	0,07099895	0,03006165	0,04097819	0,0319284	0,04979348	0,0260491	0,02660301	0,03175018	0,05512219	0,05870294	0,04005658	0,05591673	0,05009899
	FC6	0,0456866	0,02841387	0,02697021	0,03973988	0,06233261	0,06792069	0,02497133	0,02422171	0,02463758	0,04874243	0,01281295	0,02110125	0,00578306	0,0237551
	T7	0,04366882	0,02639474	0,01230013	0,02125048	0,03470182	0,04398248	0,02186818	0,02442792	0,00700546	0,02914688	0,02170711	0,00578846	0,02648315	0,01717529
	C3	0,02928052	0,05917141	0,07222242	0,03837835	0,03654428	0,03304631	0,06903145	0,0594789	0,07948938	0,04230809	0,05812661	0,09500537	0,05510072	0,09206702
	Cz	0,05258352	0,06631758	0,02545079	0,03732674	0,02163851	0,034436	0,02284531	0,02573084	0,03948249	0,0502175	0,05687777	0,04298496	0,04981483	0,06078607
	C4	0	0,04893558	0,03305099	0,06243989	0,0344941	0,02324712	0,02030135	0,0170461	0,0474905	0,04599512	0,06730301	0,02936209	0,06093636	0,0369426
	T8	0,04893558	0	0,02936405	0,07986385	0,06115235	0,0659128	0,02112497	0,01531996	0,02807739	0,05225418	0,01402466	0,03031824	0,00886319	0,02610844
	CP5	0,03305099	0,02936405	0	0,00595829	0,04096344	0,03089033	0,00364469	0,00570705	0,01571076	0,02979748	0,02437809	0,01113132	0,02577534	0,02020811
	CP1	0,06243989	0,07986385	0,00595829	0	0,04601865	0,03757873	0,02248252	0,02984621	0,02844922	0,01712578	0,03850658	0,05055638	0,04694551	0,06772843
	CP2	0,0344941	0,06115235	0,04096344	0,04601865	0	0,00695342	0,03032493	0,02971323	0,04003005	0,01787305	0,03736049	0,03009504	0,03634306	0,03269641
	CP6	0,02324712	0,0659128	0,03089033	0,03757873	0,00695342	0	0,02407224	0,01671715	0,04282817	0,01256787	0,05034554	0,04036072	0,05324919	0,03868421
	P7	0,02030135	0,02112497	0,00364469	0,02248252	0,03032493	0,02407224	0	0,00102862	0,02461576	0,02121708	0,02280052	0,01195002	0,02177924	0,01501645
	P3	0,0170461	0,01531996	0,00570705	0,02984621	0,02971323	0,01671715	0,00102862	0	0,02639208	0,01699617	0,01676322	0,01387883	0,01589108	0,02034369
	Pz	0,0474905	0,02807739	0,01571076	0,02844922	0,04003005	0,04282817	0,02461576	0,02639208	0	0,03953573	0,03331592	0,0051265	0,03607787	0,01472204
	P4	0,04599512	0,05225418	0,02979748	0,01712578	0,01787305	0,01256787	0,02121708	0,01699617	0,03953573	0	0,02050168	0,03854597	0,02688564	0,03350933
	P8	0,06730301	0,01402466	0,02437809	0,03850658	0,03736049	0,05034554	0,02280052	0,01676322	0,03331592	0,02050168	0	0,03456218	0,00111002	0,03059936
	POz	0,02936209	0,03031824	0,01113132	0,05055638	0,03009504	0,04036072	0,01195002	0,01387883	0,0051265	0,03854597	0,03456218	0	0,03685695	0,0026693
	O1	0,06093636	0,00886319	0,02577534	0,04694551	0,03634306	0,05324919	0,02177924	0,01589108	0,03607787	0,02688564	0,00111002	0,03685695	0	0,03305472
	O2	0,0369426	0,02610844	0,02020811	0,06772843	0,03269641	0,03868421	0,01501645	0,02034369	0,01472204	0,03350933	0,03059936	0,0026693	0,03305472	0
