# Supplementary material for: Hyperbrain features of team mental models within a juggling paradigm: a proof of concept
Source: PeerJ. 2016 Sep 20;4:e2457. doi: 10.7717/peerj.2457 (PMC5036110; doi:10.7717/peerj.2457)
Supplement: Supplemental Information 8 [file peerj-04-2457-s008.pdf]

		subject 1																				
		Fp1	Fpz	Fp2	F7	F3	Fz	F4	F8	FC5	FC1	FC2	FC6	T7	C3	Cz	C4	T8	CP5	CP1	CP2	CP6
subject 1	Fp1	0	0,00911421	0,01066317	0,0210151	0,02762562	0,01627603	0,01119373	0,04930622	0,03189873	0,01985418	0,0184627	0,01756187	0,00926244	0,0210509	0,01415758	0,02319966	0,04117297	0,00673458	0,03583032	0,02092122	0,03550709
	Fpz	0,00911421	0	0,00638989	0,02551291	0,02427444	0,01744033	0,0102521	0,04582397	0,04792571	0,01832279	0,02659964	0,02490983	0,01054999	0,04050283	0,02185041	0,02627163	0,03242985	0,01201765	0,02987613	0,01820284	0,02915935
	Fp2	0,01066317	0,00638989	0	0,01968343	0,02565342	0,01652477	0,01264778	0,05064134	0,03334304	0,02220849	0,02282851	0,01729516	0,01099172	0,02522254	0,01532592	0,02726943	0,04143924	0,00733458	0,03563124	0,02219629	0,03368669
	F7	0,0210151	0,02551291	0,01968343	0	0,02482533	0,01829782	0,02069995	0,02642002	0,03942174	0,01788155	0,02203711	0,01024787	0,01810145	0,03842456	0,0171648	0,03138909	0,02490151	0,02522784	0,02330111	0,02181892	0,02033752
	F3	0,02762562	0,02427444	0,02565342	0,02482533	0	0,02370133	0,02509976	0,0437471	0,02800427	0,03510323	0,02329889	0,01679131	0,01453367	0,02335247	0,01759184	0,02718552	0,03475133	0,01929974	0,03635263	0,02016535	0,03186938
	Fz	0,01627603	0,01744033	0,01652477	0,01829782	0,02370133	0	0,01141163	0,02922796	0,03171667	0,02017657	0,02662446	0,01979592	0,01421168	0,02541043	0,02579476	0,02653283	0,02320436	0,01890361	0,01506054	0,02667578	0,0202973
	F4	0,01119373	0,0102521	0,01264778	0,02069995	0,02509976	0,01141163	0	0,04254086	0,02899554	0,0181104	0,0242437	0,01873125	0,01093679	0,02266302	0,01851159	0,02801186	0,03190084	0,00507026	0,03073553	0,01919315	0,02870808
	F8	0,04930622	0,04582397	0,05064134	0,02642002	0,0437471	0,02922796	0,04254086	0	0,042686	0,05305117	0,04705834	0,03097798	0,02586417	0,03800102	0,04330713	0,07744136	0,06605667	0,04728625	0,05762473	0,05614	0,05873845
	FC5	0,03189873	0,04792571	0,03334304	0,03942174	0,02800427	0,03171667	0,02899554	0,042686	0	0,02133175	0,03133421	0,02725737	0,04098184	0,02840414	0,02900113	0,02786609	0,04440272	0,03869977	0,02206284	0,02083679	0,01616021
	FC1	0,01985418	0,01832279	0,02220849	0,01788155	0,03510323	0,02017657	0,0181104	0,05305117	0,02133175	0	0,0230951	0,02041655	0,01497387	0,01901954	0,0242081	0,03880381	0,05342205	0,01679176	0,03630264	0,03580379	0,04230313
	FC2	0,0184627	0,02659964	0,02282851	0,02203711	0,02329889	0,02662446	0,0242437	0,04705834	0,03133421	0,0230951	0	0,01182703	0,02608295	0,02248095	0,01313951	0,02671201	0,04830515	0,02492336	0,03383814	0,0163281	0,0270337
	FC6	0,01756187	0,02490983	0,01729516	0,01024787	0,01679131	0,01979592	0,01873125	0,03097798	0,02725737	0,02041655	0,01182703	0	0,0245514	0,02564442	0,0114361	0,02066464	0,03320012	0,02710024	0,0264534	0,01883873	0,02171493
	T7	0,00926244	0,01054999	0,01099172	0,01810145	0,01453367	0,01421168	0,01093679	0,02586417	0,04098184	0,01497387	0,02608295	0,0245514	0	0,03544123	0,02784393	0,01493857	0,02031779	0,01041426	0,01500634	0,02515042	0,01846049
	C3	0,0210509	0,04050283	0,02522254	0,03842456	0,02335247	0,02541043	0,02266302	0,03800102	0,02840414	0,01901954	0,02248095	0,02564442	0,03544123	0	0,02712807	0,02729954	0,03402184	0,02721289	0,02341015	0,01382462	0,0167901
	Cz	0,01415758	0,02185041	0,01532592	0,0171648	0,01759184	0,02579476	0,01851159	0,04330713	0,02900113	0,0242081	0,01313951	0,0114361	0,02784393	0,02712807	0	0,02756052	0,04722189	0,01980323	0,03566886	0,01861003	0,02484795
	C4	0,02319966	0,02627163	0,02726943	0,03138909	0,02718552	0,02653283	0,02801186	0,07744136	0,02786609	0,03880381	0,02671201	0,02066464	0,01493857	0,02729954	0,02756052	0	0,06111843	0,02143471	0,05653163	0,03220983	0,04256185
	T8	0,04117297	0,03242985	0,04143924	0,02490151	0,03475133	0,02320436	0,03190084	0,06605667	0,04440272	0,05342205	0,04830515	0,03320012	0,02031779	0,03402184	0,04722189	0,06111843	0	0,03233771	0,05402763	0,04521076	0,0305419
	CP5	0,00673458	0,01201765	0,00733458	0,02522784	0,01929974	0,01890361	0,00507026	0,04728625	0,03869977	0,01679176	0,02492336	0,02710024	0,01041426	0,02721289	0,01980323	0,02143471	0,03233771	0	0,03269899	0,02081729	0,03235184
	CP1	0,03583032	0,02987613	0,03563124	0,02330111	0,03635263	0,01506054	0,03073553	0,05762473	0,02206284	0,03630264	0,03383814	0,0264534	0,01500634	0,02341015	0,03566886	0,05653163	0,05402763	0,03269899	0	0,03870614	0,03295766
	CP2	0,02092122	0,01820284	0,02219629	0,02181892	0,02016535	0,02667578	0,01919315	0,05614	0,02083679	0,03580379	0,0163281	0,01883873	0,02515042	0,01382462	0,01861003	0,03220983	0,04521076	0,02081729	0,03870614	0	0,01662116
	CP6	0,03550709	0,02915935	0,03368669	0,02033752	0,03186938	0,0202973	0,02870808	0,05873845	0,01616021	0,04230313	0,0270337	0,02171493	0,01846049	0,0167901	0,02484795	0,04256185	0,0305419	0,03235184	0,03295766	0,01662116	0
	P7	0,01007323	0,01104293	0,01078209	0,01987234	0,01240426	0,01204416	0,01454127	0,02040579	0,04134689	0,01544481	0,02330061	0,01985172	0,0029093	0,0362329	0,02601341	0,01545372	0,0168797	0,01305043	0,01176403	0,02307413	0,0174823
	P3	0,02041558	0,01813436	0,02080212	0,01488214	0,01767647	0,0200376	0,01936826	0,0264097	0,0224677	0,02217128	0,02198898	0,01937172	0,01936724	0,01664762	0,02168638	0,02155146	0,018143	0,01946819	0,00881631	0,0307834	0,02636511
	Pz	0,04216444	0,03695927	0,03926562	0,0265205	0,03023284	0,03954919	0,03311269	0,03567323	0,03174868	0,03710592	0,03540857	0,02462193	0,02324754	0,02996619	0,02989974	0,04505985	0,0255813	0,03341662	0,0200117	0,02352316	0,01001797
	P4	0,03673672	0,03057397	0,04099205	0,01470305	0,02911992	0,01661322	0,02757495	0,05958196	0,01746755	0,03899736	0,02840409	0,0172148	0,0131198	0,01676488	0,02593978	0,04733362	0,0368535	0,03023806	0,04107763	0,0287244	0,01456514
	P8	0,03796541	0,03280082	0,0333533	0,0103098	0,03082072	0,01620852	0,03119273	0,05646521	0,02340681	0,0361364	0,03083716	0,01761698	0,00796328	0,02361452	0,02801869	0,04822821	0,04429983	0,03345997	0,02549415	0,03903424	0,02932988
	POz	0,02101433	0,02209875	0,02173412	0,0223227	0,02303277	0,03055167	0,02401951	0,05197994	0,02123585	0,02515777	0,01366723	0,02226057	0,02725129	0,01263032	0,02302675	0,02813704	0,04034566	0,02347301	0,03365963	0,00834151	0,02767666
	O1	0,02153064	0,02483821	0,02469957	0,0193948	0,01974294	0,00871158	0,02392919	0,02756334	0,01701457	0,02583397	0,02827994	0,0249041	0,0266516	0,01069228	0,02566276	0,02722857	0,0187248	0,02956389	0,01092817	0,02716121	0,02119261
	O2	0,02621048	0,02436952	0,02399477	0,01853001	0,03129721	0,02229317	0,02426095	0,02824802	0,02579557	0,02006901	0,02770841	0,02151807	0,02184818	0,01543102	0,02214794	0,02794475	0,02169836	0,02601336	0,01006091	0,02589816	0,02032022
subject 2	Fp1	0,01406147	0,01274307	0,01231185	0,01278254	0,01142044	0,0131482	0,01599163	0,01329977	0,01011526	0,01676595	0,01407619	0,01463499	0,01292465	0,01228395	0,01384996	0,00987156	0,01216734	0,0134942	0,01605975	0,01681125	0,01826756
	Fpz	0,01345446	0,01145896	0,00992495	0,01068481	0,01188164	0,01218056	0,01430879	0,01253073	0,00903379	0,01479013	0,01063264	0,01226022	0,012126	0,01167531	0,01363233	0,01145252	0,01105347	0,01450808	0,01400456	0,01408003	0,01509684
	Fp2	0,01445191	0,01132481	0,01062986	0,01029274	0,01035869	0,01258992	0,01378709	0,01276964	0,00837041	0,01274669	0,0090747	0,01065651	0,01123046	0,01415226	0,01191398	0,01011166	0,01140646	0,01286485	0,01334018	0,01194763	0,01455734
	F7	0,01483446	0,01045264	0,00979476	0,01027468	0,01130572	0,01055151	0,01458652	0,0121549	0,00733799	0,01508704	0,01115474	0,01218435	0,01206234	0,01235126	0,01385883	0,01059097	0,01149528	0,01255622	0,01538756	0,01561699	0,01719241
	F3	0,01543775	0,01558441	0,01504516	0,01355072	0,0130412	0,0142222	0,01729979	0,01373776	0,01149711	0,01412548	0,01578239	0,01656665	0,0159558	0,01548983	0,01767056	0,01287545	0,01384841	0,01497332	0,0166393	0,01722096	0,01742349
	Fz	0,01249602	0,00978738	0,01011099	0,01049581	0,00934858	0,00999739	0,01331776	0,01455186	0,00984474	0,01210111	0,01194532	0,01476958	0,01089163	0,01334991	0,01437921	0,01088868	0,0106052	0,01156284	0,01356481	0,01496904	0,016122
	F4	0,01352551	0,01031975	0,01109645	0,01106874	0,00948418	0,01075441	0,01366015	0,01431673	0,01117838	0,01239056	0,01351523	0,01260112	0,00949673	0,01504155	0,01361772	0,01095334	0,00999477	0,0112004	0,01309281	0,01238137	0,01431978
	F8	0,01076924	0,00960255	0,00932547	0,00755981	0,01021876	0,00928243	0,01219319	0,01216251	0,00706433	0,01356678	0,00795247	0,01035498	0,00868629	0,01135567	0,0096112	0,00854484	0,00863048	0,01085602	0,01220523	0,01226049	0,01446286
	FC5	0,01197088	0,0092073	0,00970263	0,00859478	0,00934512	0,00922625	0,01256858	0,015763	0,00843914	0,01014741	0,00941777	0,01186527	0,01035521	0,01353568	0,012411	0,01006809	0,00991741	0,01087041	0,01110692	0,01285314	0,01495701
	FC1	0,01143448	0,01110259	0,01180631	0,01068994	0,01143987	0,01140156	0,01599768	0,01200643	0,01060814	0,01386916	0,01247189	0,01304711	0,0114186	0,01386664	0,01382166	0,01370141	0,01115343	0,01261045	0,01295242	0,01518461	0,01624094
	FC2	0,01105239	0,00883209	0,01034008	0,00935396	0,0090255	0,0109756	0,01343103	0,01535699	0,01021672	0,0116034	0,01102684	0,01304296	0,00918114	0,01385014	0,01321315	0,01046256	0,01018275	0,01010651	0,01207266	0,01313156	0,01567922
	FC6	0,01138419	0,0085432	0,0089728	0,00778242	0,00976522	0,00858698	0,01144533	0,01072568	0,00666046	0,01292407	0,008745	0,0098953	0,00933992	0,01267787	0,01111468	0,01037031	0,00946784	0,01099302	0,01178702	0,01381041	0,01439958
	T7	0,01308534	0,00980439	0,00947033	0,00818815	0,00998524	0,00875155	0,01148048	0,01284687	0,00875936	0,01073275	0,01026577	0,01051643	0,01044739	0,01235121	0,01216716	0,01106717	0,00931473	0,01168823	0,00984742	0,01370284	0,01410201
	C3	0,01222219	0,00888851	0,00911122	0,0116422	0,01096871	0,01095773	0,01292178	0,01406887	0,00941242	0,01134104	0,0123541	0,01502934	0,01091475	0,01189718	0,01551193	0,01152656	0,01078412	0,01133213	0,01342531	0,01389589	0,01533533
	Cz	0,01312347	0,00972811	0,01010679	0,0105944	0,00871678	0,01062034	0,01173079	0,01309202	0,01004645	0,01223969	0,01232618	0,01373125	0,01058045	0,01448596	0,01278487	0,01156825	0,01002827	0,01121932	0,01176829	0,01352033	0,01331998
	C4	0,01296887	0,00971487	0,01001408	0,01151401	0,00598416	0,00963612	0,01237174	0,01420647	0,0117064	0,00993922	0,01054098	0,01257671	0,01348885	0,01206852	0,01218749	0,00998816	0,01139372	0,01188813	0,01202671	0,01222127	0,01217684
	T8	0,0132169	0,00995471	0,01012378	0,00708832	0,00761125	0,01088942	0,0133065	0,01028176	0,00689507	0,01423003	0,00949774	0,00986808	0,01099924	0,0109365	0,00992136	0,00813047	0,00976821	0,01117817	0,01077951	0,01064746	0,01231789
	CP5	0,01056973	0,00882541	0,00954417	0,00883181	0,00717122	0,00819524	0,01056214	0,01478087	0,00926066	0,00941893	0,00923535	0,01132732	0,01034459	0,01167446	0,01162878	0,01092287	0,00990853	0,01025976	0,00910392	0,00830721	0,01150831
	CP1	0,01446598	0,01199012	0,01108031	0,00771403	0,00885437	0,0128113	0,01638993	0,01066159	0,00991766	0,0131149	0,01117695	0,01160821	0,01015148	0,01031221	0,01108152	0,01120861	0,00918685	0,01415355	0,00932079	0,01261193	0,01303877
	CP2	0,01131454	0,0094659	0,00866689	0,0092774	0,00826244	0,00979176	0,01382728	0,01310978	0,01128826	0,01083403	0,01105643	0,01227529	0,01022179	0,01280628	0,01233843	0,0107444	0,00981695	0,01208967	0,01045089	0,01265182	0,01355094
	CP6	0,01282449	0,01087162	0,01021895	0,00928784	0,00883573	0,00986524	0,01273785	0,01118053	0,00965826	0,01151249	0,01089159	0,01175367	0,01238704	0,01221833	0,01125142	0,01022067	0,01187945	0,01252983	0,0119831	0,01185587	0,01239656
	P7	0,01068178	0,00745446	0,01030168	0,00860877	0,00670081	0,00881443	0,00905656	0,0156164	0,00961224	0,0083538	0,01006458	0,01043406	0,01058708	0,01072293	0,01201647	0,01112004	0,0112166	0,0109922	0,00959014	0,01008068	0,0103383
	P3	0,01175913	0,00892638	0,01167673	0,00836377	0,00746526	0,0083725	0,01180219	0,01407718	0,0103107	0,00922151	0,0087014	0,01051672	0,00937218	0,01010783	0,01033725	0,01151461	0,01125037	0,01096511	0,01023409	0,00925556	0,01086581
	Pz	0,01252685	0,00991118	0,00964196	0,00785896	0,00787871	0,00848864	0,01199147	0,01264616	0,01028255	0,01193328	0,00929155	0,01086085	0,01248849	0,01412729	0,01190733	0,01196185	0,00997275	0,01117534	0,01086783	0,01139681	0,01463932
	P4	0,01188312	0,00960775	0,00989637	0,0083951	0,00827438	0,01013658	0,01134097	0,01144503	0,009845	0,01150553	0,01010768	0,010543	0,01028413	0,01452111	0,01042662	0,01030195	0,01034848	0,01151959	0,0110786	0,01209289	0,01220912
	P8	0,01287528	0,01014952	0,00942622	0,0105821	0,00739176	0,01012053	0,01229671	0,01221995	0,00827092	0,01257831	0,00835108	0,00909316	0,00841576	0,0128802	0,01086284	0,0099425	0,01089751	0,0112866	0,01167107	0,0104409	0,01237402
	POz	0,01098469	0,0100755	0,00956688	0,00899918	0,00826792	0,00867445	0,01286716	0,01566046	0,00985991	0,01213219	0,01006451	0,01144718	0,01105042	0,01204325	0,01160909	0,01035752	0,00869792	0,01195334	0,00878496	0,01165568	0,01180641
	O1	0,01244173	0,00885068	0,00951828	0,0093598	0,00729277	0,00989024	0,0135359	0,01113485	0,0069257	0,01310431	0,00733747	0,00797189	0,00985224	0,01222724	0,00965411	0,00944122	0,00927875	0,01091749	0,01116978	0,01144663	0,01141103
	O2	0,01334364	0,01201047	0,01023727	0,00937603	0,00906944	0,01025365	0,01410533	0,01426763	0,01039923	0,01289453	0,00965379	0,01269362	0,01177662	0,01303606	0,01167005	0,01109668	0,00872386	0,01280149	0,01039596	0,01183898	0,0154437

		subject 1								subject 2											
		P7	P3	Pz	P4	P8	POz	O1	O2	Fp1	Fpz	Fp2	F7	F3	Fz	F4	F8	FC5	FC1	FC2	FC6
subject 1	Fp1	0,01007323	0,02041558	0,04216444	0,03673672	0,03796541	0,02101433	0,02153064	0,02621048	0,01406147	0,01345446	0,01445191	0,01483446	0,01543775	0,01249602	0,01352551	0,01076924	0,01197088	0,01143448	0,01105239	0,01138419
	Fpz	0,01104293	0,01813436	0,03695927	0,03057397	0,03280082	0,02209875	0,02483821	0,02436952	0,01274307	0,01145896	0,01132481	0,01045264	0,01558441	0,00978738	0,01031975	0,00960255	0,0092073	0,01110259	0,00883209	0,0085432
	Fp2	0,01078209	0,02080212	0,03926562	0,04099205	0,0333533	0,02173412	0,02469957	0,02399477	0,01231185	0,00992495	0,01062986	0,00979476	0,01504516	0,01011099	0,01109645	0,00932547	0,00970263	0,01180631	0,01034008	0,0089728
	F7	0,01987234	0,01488214	0,0265205	0,01470305	0,0103098	0,0223227	0,0193948	0,01853001	0,01278254	0,01068481	0,01029274	0,01027468	0,01355072	0,01049581	0,01106874	0,00755981	0,00859478	0,01068994	0,00935396	0,00778242
	F3	0,01240426	0,01767647	0,03023284	0,02911992	0,03082072	0,02303277	0,01974294	0,03129721	0,01142044	0,01188164	0,01035869	0,01130572	0,0130412	0,00934858	0,00948418	0,01021876	0,00934512	0,01143987	0,0090255	0,00976522
	Fz	0,01204416	0,0200376	0,03954919	0,01661322	0,01620852	0,03055167	0,00871158	0,02229317	0,0131482	0,01218056	0,01258992	0,01055151	0,0142222	0,00999739	0,01075441	0,00928243	0,00922625	0,01140156	0,0109756	0,00858698
	F4	0,01454127	0,01936826	0,03311269	0,02757495	0,03119273	0,02401951	0,02392919	0,02426095	0,01599163	0,01430879	0,01378709	0,01458652	0,01729979	0,01331776	0,01366015	0,01219319	0,01256858	0,01599768	0,01343103	0,01144533
	F8	0,02040579	0,0264097	0,03567323	0,05958196	0,05646521	0,05197994	0,02756334	0,02824802	0,01329977	0,01253073	0,01276964	0,0121549	0,01373776	0,01455186	0,01431673	0,01216251	0,015763	0,01200643	0,01535699	0,01072568
	FC5	0,04134689	0,0224677	0,03174868	0,01746755	0,02340681	0,02123585	0,01701457	0,02579557	0,01011526	0,00903379	0,00837041	0,00733799	0,01149711	0,00984474	0,01117838	0,00706433	0,00843914	0,01060814	0,01021672	0,00666046
	FC1	0,01544481	0,02217128	0,03710592	0,03899736	0,0361364	0,02515777	0,02583397	0,02006901	0,01676595	0,01479013	0,01274669	0,01508704	0,01412548	0,01210111	0,01239056	0,01356678	0,01014741	0,01386916	0,0116034	0,01292407
	FC2	0,02330061	0,02198898	0,03540857	0,02840409	0,03083716	0,01366723	0,02827994	0,02770841	0,01407619	0,01063264	0,0090747	0,01115474	0,01578239	0,01194532	0,01351523	0,00795247	0,00941777	0,01247189	0,01102684	0,008745
	FC6	0,01985172	0,01937172	0,02462193	0,0172148	0,01761698	0,02226057	0,0249041	0,02151807	0,01463499	0,01226022	0,01065651	0,01218435	0,01656665	0,01476958	0,01260112	0,01035498	0,01186527	0,01304711	0,01304296	0,0098953
	T7	0,0029093	0,01936724	0,02324754	0,0131198	0,00796328	0,02725129	0,0266516	0,02184818	0,01292465	0,012126	0,01123046	0,01206234	0,0159558	0,01089163	0,00949673	0,00868629	0,01035521	0,0114186	0,00918114	0,00933992
	C3	0,0362329	0,01664762	0,02996619	0,01676488	0,02361452	0,01263032	0,01069228	0,01543102	0,01228395	0,01167531	0,01415226	0,01235126	0,01548983	0,01334991	0,01504155	0,01135567	0,01353568	0,01386664	0,01385014	0,01267787
	Cz	0,02601341	0,02168638	0,02989974	0,02593978	0,02801869	0,02302675	0,02566276	0,02214794	0,01384996	0,01363233	0,01191398	0,01385883	0,01767056	0,01437921	0,01361772	0,0096112	0,012411	0,01382166	0,01321315	0,01111468
	C4	0,01545372	0,02155146	0,04505985	0,04733362	0,04822821	0,02813704	0,02722857	0,02794475	0,00987156	0,01145252	0,01011166	0,01059097	0,01287545	0,01088868	0,01095334	0,00854484	0,01006809	0,01370141	0,01046256	0,01037031
	T8	0,0168797	0,018143	0,0255813	0,0368535	0,04429983	0,04034566	0,0187248	0,02169836	0,01216734	0,01105347	0,01140646	0,01149528	0,01384841	0,0106052	0,00999477	0,00863048	0,00991741	0,01115343	0,01018275	0,00946784
	CP5	0,01305043	0,01946819	0,03341662	0,03023806	0,03345997	0,02347301	0,02956389	0,02601336	0,0134942	0,01450808	0,01286485	0,01255622	0,01497332	0,01156284	0,0112004	0,01085602	0,01087041	0,01261045	0,01010651	0,01099302
	CP1	0,01176403	0,00881631	0,0200117	0,04107763	0,02549415	0,03365963	0,01092817	0,01006091	0,01605975	0,01400456	0,01334018	0,01538756	0,0166393	0,01356481	0,01309281	0,01220523	0,01110692	0,01295242	0,01207266	0,01178702
	CP2	0,02307413	0,0307834	0,02352316	0,0287244	0,03903424	0,00834151	0,02716121	0,02589816	0,01681125	0,01408003	0,01194763	0,01561699	0,01722096	0,01496904	0,01238137	0,01226049	0,01285314	0,01518461	0,01313156	0,01381041
	CP6	0,0174823	0,02636511	0,01001797	0,01456514	0,02932988	0,02767666	0,02119261	0,02032022	0,01826756	0,01509684	0,01455734	0,01719241	0,01742349	0,016122	0,01431978	0,01446286	0,01495701	0,01624094	0,01567922	0,01439958
	P7	0	0,01528595	0,0210951	0,01350317	0,00559873	0,02481153	0,02387874	0,01756751	0,01330342	0,01399088	0,01268953	0,01218454	0,01624208	0,01153957	0,01042097	0,01033109	0,01074498	0,01254294	0,01061629	0,01057158
	P3	0,01528595	0	0,0269434	0,01805595	0,01172833	0,02738567	0,01508543	0,01916157	0,01162424	0,00812799	0,00758916	0,00991153	0,0124013	0,00848649	0,00904531	0,0049016	0,00728253	0,01141387	0,00747554	0,00570355
	Pz	0,0210951	0,0269434	0	0,02278849	0,00863119	0,03603935	0,03541925	0,04051385	0,01258962	0,01182172	0,01177058	0,0127936	0,0137946	0,01039528	0,00855293	0,00956177	0,01092612	0,01098755	0,00946881	0,0090841
	P4	0,01350317	0,01805595	0,02278849	0	0,03470748	0,02491778	0,01057607	0,01162536	0,01863463	0,01803757	0,01621851	0,01712525	0,01719785	0,01448245	0,01487867	0,01301895	0,01323549	0,01516345	0,01357863	0,01170644
	P8	0,00559873	0,01172833	0,00863119	0,03470748	0	0,0313323	0,01459701	0,01222217	0,0183144	0,01468575	0,01281792	0,01709107	0,01634987	0,01314124	0,01428492	0,01493744	0,0119131	0,01390825	0,01413506	0,01520748
	POz	0,02481153	0,02738567	0,03603935	0,02491778	0,0313323	0	0,02751428	0,02318488	0,01195574	0,01068977	0,01092278	0,01175756	0,01302964	0,01239196	0,01217727	0,01121281	0,01008934	0,01231412	0,01100941	0,0116469
	O1	0,02387874	0,01508543	0,03541925	0,01057607	0,01459701	0,02751428	0	0,01323989	0,01127306	0,01062114	0,01204927	0,01107213	0,01252829	0,01050488	0,01051405	0,01254158	0,01104472	0,011673	0,01131164	0,01320864
	O2	0,01756751	0,01916157	0,04051385	0,01162536	0,01222217	0,02318488	0,01323989	0	0,01076925	0,00918458	0,00822586	0,00889974	0,01403574	0,00784001	0,00841368	0,0057343	0,00928014	0,0109189	0,00812765	0,00663006
subject 2	Fp1	0,01330342	0,01162424	0,01258962	0,01863463	0,0183144	0,01195574	0,01127306	0,01076925	0	0,00178133	0,00703635	0,00146693	0,0319216	0,03862907	0,03790628	0,02567377	0,04319745	0,03204688	0,04296581	0,03963656
	Fpz	0,01399088	0,00812799	0,01182172	0,01803757	0,01468575	0,01068977	0,01062114	0,00918458	0,00178133	0	0,00232217	0,00122819	0,03162294	0,04233246	0,03789669	0,02478717	0,03971649	0,03661183	0,04747354	0,03538709
	Fp2	0,01268953	0,00758916	0,01177058	0,01621851	0,01281792	0,01092278	0,01204927	0,00822586	0,00703635	0,00232217	0	0,00400748	0,0281655	0,04476105	0,0408478	0,01599613	0,02826714	0,03733282	0,04645763	0,02856243
	F7	0,01218454	0,00991153	0,0127936	0,01712525	0,01709107	0,01175756	0,01107213	0,00889974	0,00146693	0,00122819	0,00400748	0	0,0335924	0,04219933	0,04057236	0,02510847	0,04349295	0,03622637	0,04516284	0,0395637
	F3	0,01624208	0,0124013	0,0137946	0,01719785	0,01634987	0,01302964	0,01252829	0,01403574	0,0319216	0,03162294	0,0281655	0,0335924	0	0,01056259	0,01147962	0,03433262	0,04441352	0,00556826	0,0252634	0,04264663
	Fz	0,01153957	0,00848649	0,01039528	0,01448245	0,01314124	0,01239196	0,01050488	0,00784001	0,03862907	0,04233246	0,04476105	0,04219933	0,01056259	0	0,00397484	0,04069896	0,03866701	0,00232033	0,0128423	0,03410281
	F4	0,01042097	0,00904531	0,00855293	0,01487867	0,01428492	0,01217727	0,01051405	0,00841368	0,03790628	0,03789669	0,0408478	0,04057236	0,01147962	0,00397484	0	0,03648197	0,03370194	0,00636047	0,00732036	0,03890823
	F8	0,01033109	0,0049016	0,00956177	0,01301895	0,01493744	0,01121281	0,01254158	0,0057343	0,02567377	0,02478717	0,01599613	0,02510847	0,03433262	0,04069896	0,03648197	0	0,01125307	0,03815911	0,04426098	0,00511474
	FC5	0,01074498	0,00728253	0,01092612	0,01323549	0,0119131	0,01008934	0,01104472	0,00928014	0,04319745	0,03971649	0,02826714	0,04349295	0,04441352	0,03866701	0,03370194	0,01125307	0	0,047146	0,02419307	0,01256019
	FC1	0,01254294	0,01141387	0,01098755	0,01516345	0,01390825	0,01231412	0,011673	0,0109189	0,03204688	0,03661183	0,03733282	0,03622637	0,00556826	0,00232033	0,00636047	0,03815911	0,047146	0	0,01777753	0,04064372
	FC2	0,01061629	0,00747554	0,00946881	0,01357863	0,01413506	0,01100941	0,01131164	0,00812765	0,04296581	0,04747354	0,04645763	0,04516284	0,0252634	0,0128423	0,00732036	0,04426098	0,02419307	0,01777753	0	0,03622069
	FC6	0,01057158	0,00570355	0,0090841	0,01170644	0,01520748	0,0116469	0,01320864	0,00663006	0,03963656	0,03538709	0,02856243	0,0395637	0,04264663	0,03410281	0,03890823	0,00511474	0,01256019	0,04064372	0,03622069	0
	T7	0,01261558	0,0075954	0,01065043	0,01376438	0,01264654	0,01096784	0,01048096	0,00815356	0,04039061	0,03462044	0,02630298	0,03778719	0,03942349	0,03991661	0,03289276	0,01560748	0,00162168	0,04406284	0,02852721	0,01642217
	C3	0,0136176	0,00904489	0,01192533	0,01660389	0,013594	0,01248411	0,01227584	0,00859913	0,03451031	0,03585034	0,03468239	0,03755302	0,01279941	0,01147792	0,02145323	0,03714369	0,03501048	0,01145103	0,02016536	0,03435653
	Cz	0,01092602	0,00649485	0,01040281	0,0130208	0,01335687	0,01174145	0,01027697	0,00735723	0,04321987	0,05149302	0,04260699	0,04651252	0,02278896	0,0056287	0,00657192	0,03015352	0,02096957	0,0161655	0,00639769	0,03196482
	C4	0,01292908	0,00634304	0,00854488	0,01096103	0,01370396	0,00863956	0,01040981	0,0058877	0,03532541	0,0346226	0,03543888	0,03285134	0,03230858	0,0355932	0,03229065	0,02724101	0,03377159	0,03329666	0,02003823	0,02872991
	T8	0,01085479	0,00512194	0,01068082	0,01219087	0,01454989	0,01052124	0,01297775	0,00597114	0,0166086	0,01596862	0,01403191	0,01576397	0,02111355	0,03748131	0,03520436	0,0021564	0,01683465	0,03452365	0,03961717	0,0102772
	CP5	0,01133845	0,0095706	0,00973103	0,01258566	0,01033213	0,00906268	0,00884761	0,00685297	0,03403151	0,02761093	0,02672052	0,03558888	0,03179936	0,02964436	0,02393386	0,02403119	0,01541485	0,03384161	0,01867335	0,02225119
	CP1	0,01140402	0,00837143	0,01281239	0,01340631	0,01138292	0,01233101	0,01472538	0,01211933	0,02622412	0,02732082	0,02404204	0,0255316	0,02832647	0,02723185	0,03109858	0,02252618	0,01924229	0,02497657	0,03453181	0,02676881
	CP2	0,01052038	0,00618476	0,00935003	0,01206327	0,01349558	0,01100759	0,00962208	0,00652224	0,04374312	0,03577099	0,0329069	0,04051288	0,03417486	0,02808991	0,0282626	0,03540249	0,01765416	0,03635649	0,0151802	0,03346525
	CP6	0,01325433	0,00580362	0,00866219	0,01221981	0,01293881	0,01092737	0,011094	0,00698334	0,02942781	0,02817625	0,02630138	0,03138888	0,02986295	0,02994398	0,03676309	0,03454451	0,02244647	0,03453461	0,02387485	0,04216568
	P7	0,01046068	0,00858165	0,01056354	0,01223117	0,01067915	0,00867006	0,00976052	0,007428	0,0222874	0,02301343	0,02064144	0,02492837	0,0242418	0,02238671	0,01704908	0,01831959	0,01622742	0,02446331	0,01336432	0,02106415
	P3	0,00981371	0,00889658	0,01026097	0,01111819	0,01073608	0,01058175	0,01020064	0,00724436	0,02100298	0,02006125	0,01594705	0,02062387	0,02140406	0,01509918	0,0129528	0,01520878	0,01431459	0,02051577	0,00932856	0,01490437
	Pz	0,01253043	0,00874189	0,01151387	0,01176905	0,01143218	0,01087306	0,01138125	0,00881298	0,03436416	0,03365684	0,02545694	0,03806795	0,040896	0,0366703	0,0323483	0,01920523	0,00406116	0,04509329	0,02375162	0,01867656
	P4	0,00990943	0,00638071	0,01025729	0,01363501	0,01233315	0,00953153	0,01129397	0,00740417	0,02725626	0,02466493	0,01616095	0,02381993	0,0299231	0,03748427	0,03986759	0,02538663	0,01834639	0,03728591	0,0283893	0,02898392
	P8	0,0098964	0,00590217	0,01000431	0,01190372	0,01273493	0,01104545	0,01103101	0,00637991	0,02793994	0,02522618	0,01659626	0,02822061	0,03480208	0,04058042	0,03711771	0,00520593	0,0208168	0,04213163	0,03560583	0,01059399
	POz	0,01123945	0,01104096	0,00973177	0,01084779	0,01142529	0,00995756	0,01169736	0,00892226	0,02225171	0,02119039	0,01719617	0,02312252	0,02804945	0,03788444	0,03175982	0,02184016	0,00847416	0,03971309	0,0286967	0,02186048
	O1	0,00950896	0,00556254	0,01116909	0,01310427	0,01391512	0,01035269	0,01196716	0,00621873	0,03139918	0,03070273	0,01925215	0,02750971	0,03153315	0,03857324	0,03781509	0,00527068	0,02337871	0,04011219	0,03119575	0,00584466
	O2	0,01207844	0,00963586	0,01141575	0,01311654	0,01275207	0,01199382	0,01268655	0,00868676	0,0190466	0,0145803	0,01307265	0,01768907	0,03057998	0,04430617	0,03567799	0,0168517	0,01093116	0,04192764	0,03663418	0,02178635

		subject 2																
		T7	C3	Cz	C4	T8	CP5	CP1	CP2	CP6	P7	P3	Pz	P4	P8	POz	O1	O2
subject 1	Fp1	0,01308534	0,01222219	0,01312347	0,01296887	0,0132169	0,01056973	0,01446598	0,01131454	0,01282449	0,01068178	0,01175913	0,01252685	0,01188312	0,01287528	0,01098469	0,01244173	0,01334364
	Fpz	0,00980439	0,00888851	0,00972811	0,00971487	0,00995471	0,00882541	0,01199012	0,0094659	0,01087162	0,00745446	0,00892638	0,00991118	0,00960775	0,01014952	0,0100755	0,00885068	0,01201047
	Fp2	0,00947033	0,00911122	0,01010679	0,01001408	0,01012378	0,00954417	0,01108031	0,00866689	0,01021895	0,01030168	0,01167673	0,00964196	0,00989637	0,00942622	0,00956688	0,00951828	0,01023727
	F7	0,00818815	0,0116422	0,0105944	0,01151401	0,00708832	0,00883181	0,00771403	0,0092774	0,00928784	0,00860877	0,00836377	0,00785896	0,0083951	0,0105821	0,00899918	0,0093598	0,00937603
	F3	0,00998524	0,01096871	0,00871678	0,00598416	0,00761125	0,00717122	0,00885437	0,00826244	0,00883573	0,00670081	0,00746526	0,00787871	0,00827438	0,00739176	0,00826792	0,00729277	0,00906944
	Fz	0,00875155	0,01095773	0,01062034	0,00963612	0,01088942	0,00819524	0,0128113	0,00979176	0,00986524	0,00881443	0,0083725	0,00848864	0,01013658	0,01012053	0,00867445	0,00989024	0,01025365
	F4	0,01148048	0,01292178	0,01173079	0,01237174	0,0133065	0,01056214	0,01638993	0,01382728	0,01273785	0,00905656	0,01180219	0,01199147	0,01134097	0,01229671	0,01286716	0,0135359	0,01410533
	F8	0,01284687	0,01406887	0,01309202	0,01420647	0,01028176	0,01478087	0,01066159	0,01310978	0,01118053	0,0156164	0,01407718	0,01264616	0,01144503	0,01221995	0,01566046	0,01113485	0,01426763
	FC5	0,00875936	0,00941242	0,01004645	0,0117064	0,00689507	0,00926066	0,00991766	0,01128826	0,00965826	0,00961224	0,0103107	0,01028255	0,009845	0,00827092	0,00985991	0,0069257	0,01039923
	FC1	0,01073275	0,01134104	0,01223969	0,00993922	0,01423003	0,00941893	0,0131149	0,01083403	0,01151249	0,0083538	0,00922151	0,01193328	0,01150553	0,01257831	0,01213219	0,01310431	0,01289453
	FC2	0,01026577	0,0123541	0,01232618	0,01054098	0,00949774	0,00923535	0,01117695	0,01105643	0,01089159	0,01006458	0,0087014	0,00929155	0,01010768	0,00835108	0,01006451	0,00733747	0,00965379
	FC6	0,01051643	0,01502934	0,01373125	0,01257671	0,00986808	0,01132732	0,01160821	0,01227529	0,01175367	0,01043406	0,01051672	0,01086085	0,010543	0,00909316	0,01144718	0,00797189	0,01269362
	T7	0,01044739	0,01091475	0,01058045	0,01348885	0,01099924	0,01034459	0,01015148	0,01022179	0,01238704	0,01058708	0,00937218	0,01248849	0,01028413	0,00841576	0,01105042	0,00985224	0,01177662
	C3	0,01235121	0,01189718	0,01448596	0,01206852	0,0109365	0,01167446	0,01031221	0,01280628	0,01221833	0,01072293	0,01010783	0,01412729	0,01452111	0,0128802	0,01204325	0,01222724	0,01303606
	Cz	0,01216716	0,01551193	0,01278487	0,01218749	0,00992136	0,01162878	0,01108152	0,01233843	0,01125142	0,01201647	0,01033725	0,01190733	0,01042662	0,01086284	0,01160909	0,00965411	0,01167005
	C4	0,01106717	0,01152656	0,01156825	0,00998816	0,00813047	0,01092287	0,01120861	0,0107444	0,01022067	0,01112004	0,01151461	0,01196185	0,01030195	0,0099425	0,01035752	0,00944122	0,01109668
	T8	0,00931473	0,01078412	0,01002827	0,01139372	0,00976821	0,00990853	0,00918685	0,00981695	0,01187945	0,0112166	0,01125037	0,00997275	0,01034848	0,01089751	0,00869792	0,00927875	0,00872386
	CP5	0,01168823	0,01133213	0,01121932	0,01188813	0,01117817	0,01025976	0,01415355	0,01208967	0,01252983	0,0109922	0,01096511	0,01117534	0,01151959	0,0112866	0,01195334	0,01091749	0,01280149
	CP1	0,00984742	0,01342531	0,01176829	0,01202671	0,01077951	0,00910392	0,00932079	0,01045089	0,0119831	0,00959014	0,01023409	0,01086783	0,0110786	0,01167107	0,00878496	0,01116978	0,01039596
	CP2	0,01370284	0,01389589	0,01352033	0,01222127	0,01064746	0,00830721	0,01261193	0,01265182	0,01185587	0,01008068	0,00925556	0,01139681	0,01209289	0,0104409	0,01165568	0,01144663	0,01183898
	CP6	0,01410201	0,01533533	0,01331998	0,01217684	0,01231789	0,01150831	0,01303877	0,01355094	0,01239656	0,0103383	0,01086581	0,01463932	0,01220912	0,01237402	0,01180641	0,01141103	0,0154437
	P7	0,01261558	0,0136176	0,01092602	0,01292908	0,01085479	0,01133845	0,01140402	0,01052038	0,01325433	0,01046068	0,00981371	0,01253043	0,00990943	0,0098964	0,01123945	0,00950896	0,01207844
	P3	0,0075954	0,00904489	0,00649485	0,00634304	0,00512194	0,0095706	0,00837143	0,00618476	0,00580362	0,00858165	0,00889658	0,00874189	0,00638071	0,00590217	0,01104096	0,00556254	0,00963586
	Pz	0,01065043	0,01192533	0,01040281	0,00854488	0,01068082	0,00973103	0,01281239	0,00935003	0,00866219	0,01056354	0,01026097	0,01151387	0,01025729	0,01000431	0,00973177	0,01116909	0,01141575
	P4	0,01376438	0,01660389	0,0130208	0,01096103	0,01219087	0,01258566	0,01340631	0,01206327	0,01221981	0,01223117	0,01111819	0,01176905	0,01363501	0,01190372	0,01084779	0,01310427	0,01311654
	P8	0,01264654	0,013594	0,01335687	0,01370396	0,01454989	0,01033213	0,01138292	0,01349558	0,01293881	0,01067915	0,01073608	0,01143218	0,01233315	0,01273493	0,01142529	0,01391512	0,01275207
	POz	0,01096784	0,01248411	0,01174145	0,00863956	0,01052124	0,00906268	0,01233101	0,01100759	0,01092737	0,00867006	0,01058175	0,01087306	0,00953153	0,01104545	0,00995756	0,01035269	0,01199382
	O1	0,01048096	0,01227584	0,01027697	0,01040981	0,01297775	0,00884761	0,01472538	0,00962208	0,011094	0,00976052	0,01020064	0,01138125	0,01129397	0,01103101	0,01169736	0,01196716	0,01268655
	O2	0,00815356	0,00859913	0,00735723	0,0058877	0,00597114	0,00685297	0,01211933	0,00652224	0,00698334	0,007428	0,00724436	0,00881298	0,00740417	0,00637991	0,00892226	0,00621873	0,00868676
subject 2	Fp1	0,04039061	0,03451031	0,04321987	0,03532541	0,0166086	0,03403151	0,02622412	0,04374312	0,02942781	0,0222874	0,02100298	0,03436416	0,02725626	0,02793994	0,02225171	0,03139918	0,0190466
	Fpz	0,03462044	0,03585034	0,05149302	0,0346226	0,01596862	0,02761093	0,02732082	0,03577099	0,02817625	0,02301343	0,02006125	0,03365684	0,02466493	0,02522618	0,02119039	0,03070273	0,0145803
	Fp2	0,02630298	0,03468239	0,04260699	0,03543888	0,01403191	0,02672052	0,02404204	0,0329069	0,02630138	0,02064144	0,01594705	0,02545694	0,01616095	0,01659626	0,01719617	0,01925215	0,01307265
	F7	0,03778719	0,03755302	0,04651252	0,03285134	0,01576397	0,03558888	0,0255316	0,04051288	0,03138888	0,02492837	0,02062387	0,03806795	0,02381993	0,02822061	0,02312252	0,02750971	0,01768907
	F3	0,03942349	0,01279941	0,02278896	0,03230858	0,02111355	0,03179936	0,02832647	0,03417486	0,02986295	0,0242418	0,02140406	0,040896	0,0299231	0,03480208	0,02804945	0,03153315	0,03057998
	Fz	0,03991661	0,01147792	0,0056287	0,0355932	0,03748131	0,02964436	0,02723185	0,02808991	0,02994398	0,02238671	0,01509918	0,0366703	0,03748427	0,04058042	0,03788444	0,03857324	0,04430617
	F4	0,03289276	0,02145323	0,00657192	0,03229065	0,03520436	0,02393386	0,03109858	0,0282626	0,03676309	0,01704908	0,0129528	0,0323483	0,03986759	0,03711771	0,03175982	0,03781509	0,03567799
	F8	0,01560748	0,03714369	0,03015352	0,02724101	0,0021564	0,02403119	0,02252618	0,03540249	0,03454451	0,01831959	0,01520878	0,01920523	0,02538663	0,00520593	0,02184016	0,00527068	0,0168517
	FC5	0,00162168	0,03501048	0,02096957	0,03377159	0,01683465	0,01541485	0,01924229	0,01765416	0,02244647	0,01622742	0,01431459	0,00406116	0,01834639	0,0208168	0,00847416	0,02337871	0,01093116
	FC1	0,04406284	0,01145103	0,0161655	0,03329666	0,03452365	0,03384161	0,02497657	0,03635649	0,03453461	0,02446331	0,02051577	0,04509329	0,03728591	0,04213163	0,03971309	0,04011219	0,04192764
	FC2	0,02852721	0,02016536	0,00639769	0,02003823	0,03961717	0,01867335	0,03453181	0,0151802	0,02387485	0,01336432	0,00932856	0,02375162	0,0283893	0,03560583	0,0286967	0,03119575	0,03663418
	FC6	0,01642217	0,03435653	0,03196482	0,02872991	0,0102772	0,02225119	0,02676881	0,03346525	0,04216568	0,02106415	0,01490437	0,01867656	0,02898392	0,01059399	0,02186048	0,00584466	0,02178635
	T7	0	0,04160693	0,02050036	0,03074909	0,02060395	0,0103254	0,01639634	0,02245241	0,02463116	0,01239115	0,01245348	0,00375462	0,01857047	0,02683629	0,00460994	0,02714908	0,00787937
	C3	0,04160693	0	0,01530415	0,02927924	0,03314442	0,03827818	0,01886661	0,02739549	0,02663281	0,02970968	0,02501597	0,04225361	0,02739528	0,0360729	0,04416233	0,03542145	0,04560575
	Cz	0,02050036	0,01530415	0	0,0317787	0,03527235	0,01870257	0,02844719	0,01491803	0,02592718	0,01527796	0,01219822	0,02210646	0,02922405	0,0297253	0,03104958	0,02869401	0,03815576
	C4	0,03074909	0,02927924	0,0317787	0	0,0219261	0,01636119	0,02498107	0,01651363	0,00972382	0,00734732	0,00545365	0,03028267	0,02649426	0,03513227	0,02237862	0,03331242	0,03067866
	T8	0,02060395	0,03314442	0,03527235	0,0219261	0	0,02227267	0,03159894	0,03456385	0,04148679	0,01947264	0,0125998	0,01943763	0,03153105	0,01093096	0,02541223	0,00554402	0,02165496
	CP5	0,0103254	0,03827818	0,01870257	0,01636119	0,02227267	0	0,0174835	0,01694379	0,01197728	0,00301539	0,00610597	0,00803768	0,01508422	0,02179216	0,00507975	0,01943363	0,01292416
	CP1	0,01639634	0,01886661	0,02844719	0,02498107	0,03159894	0,0174835	0	0,02884617	0,01943748	0,02598999	0,02520424	0,01271865	0,01603302	0,02155967	0,0165526	0,02975975	0,02318013
	CP2	0,02245241	0,02739549	0,01491803	0,01651363	0,03456385	0,01694379	0,02884617	0	0,00436079	0,0121763	0,00943419	0,02132592	0,00839006	0,02110033	0,02245256	0,02435964	0,02130375
	CP6	0,02463116	0,02663281	0,02592718	0,00972382	0,04148679	0,01197728	0,01943748	0,00436079	0	0,00802902	0,01043954	0,02402674	0,00786749	0,03012319	0,02010486	0,0378425	0,02643122
	P7	0,01239115	0,02970968	0,01527796	0,00734732	0,01947264	0,00301539	0,02598999	0,0121763	0,00802902	0	0,00245326	0,00919398	0,00866099	0,01233907	0,00675961	0,01359596	0,01518116
	P3	0,01245348	0,02501597	0,01219822	0,00545365	0,0125998	0,00610597	0,02520424	0,00943419	0,01043954	0,00245326	0	0,00987411	0,00940701	0,01034892	0,00873708	0,01397288	0,01255397
	Pz	0,00375462	0,04225361	0,02210646	0,03028267	0,01943763	0,00803768	0,01271865	0,02132592	0,02402674	0,00919398	0,00987411	0	0,01973368	0,02363037	0,00670982	0,02907526	0,00900275
	P4	0,01857047	0,02739528	0,02922405	0,02649426	0,03153105	0,01508422	0,01603302	0,00839006	0,00786749	0,00866099	0,00940701	0,01973368	0	0,01458398	0,02002277	0,0204107	0,0212934
	P8	0,02683629	0,0360729	0,0297253	0,03513227	0,01093096	0,02179216	0,02155967	0,02110033	0,03012319	0,01233907	0,01034892	0,02363037	0,01458398	0	0,02762858	0,00121732	0,02756199
	POz	0,00460994	0,04416233	0,03104958	0,02237862	0,02541223	0,00507975	0,0165526	0,02245256	0,02010486	0,00675961	0,00873708	0,00670982	0,02002277	0,02762858	0	0,02718528	0,00448727
	O1	0,02714908	0,03542145	0,02869401	0,03331242	0,00554402	0,01943363	0,02975975	0,02435964	0,0378425	0,01359596	0,01397288	0,02907526	0,0204107	0,00121732	0,02718528	0	0,02644869
	O2	0,00787937	0,04560575	0,03815576	0,03067866	0,02165496	0,01292416	0,02318013	0,02130375	0,02643122	0,01518116	0,01255397	0,00900275	0,0212934	0,02756199	0,00448727	0,02644869	0
