# Supplementary material for: Hyperbrain features of team mental models within a juggling paradigm: a proof of concept
Source: PeerJ. 2016 Sep 20;4:e2457. doi: 10.7717/peerj.2457 (PMC5036110; doi:10.7717/peerj.2457)
Supplement: Supplemental Information 4 [file peerj-04-2457-s004.pdf]

## subject 1 (columns)

| | | Fp1 | Fpz | Fp2 | F7 | F3 | Fz | F4 | F8 | FC5 | FC1 | FC2 | FC6 | T7 | C3 | Cz | C4 | T8 | CP5 | CP1 | CP2 | CP6 | P7 | P3 | Pz | P4 | P8 | POz | O1 | O2 |
|---|---|---|---|---|---|---|---|---|---|---|---|---|---|---|---|---|---|---|---|---|---|---|---|---|---|---|---|---|---|---|
| subject 1 | Fp1 | 0 | 0,0040118 | 0,00615106 | 0,02300777 | 0,01505905 | 0,01433833 | 0,02767851 | 0,0171361 | 0,02319493 | 0,00979253 | 0,02712889 | 0,02366616 | 0,02941294 | 0,02553019 | 0,02852371 | 0,02165873 | 0,01853801 | 0,01874509 | 0,02838001 | 0,03381576 | 0,02480859 | 0,03146826 | 0,03384667 | 0,03054588 | 0,02529288 | 0,02479579 | 0,03658789 | 0,0387112 | 0,03022218 |
| | Fpz | 0,0040118 | 0 | 0,0072936 | 0,02315707 | 0,01327145 | 0,01871674 | 0,02225178 | 0,0244794 | 0,01831876 | 0,01633396 | 0,0309211 | 0,02867914 | 0,03404948 | 0,02375128 | 0,03247116 | 0,02576686 | 0,0162764 | 0,02003784 | 0,02161739 | 0,03898303 | 0,03244512 | 0,03129383 | 0,03932357 | 0,03802478 | 0,02359379 | 0,02971264 | 0,04323255 | 0,03669147 | 0,03678563 |
| | Fp2 | 0,00615106 | 0,0072936 | 0 | 0,02088151 | 0,0137013 | 0,01618396 | 0,02586109 | 0,02174909 | 0,02270556 | 0,01293324 | 0,03005198 | 0,02671294 | 0,02832202 | 0,02431465 | 0,02637963 | 0,02555824 | 0,01835463 | 0,02137065 | 0,02648075 | 0,04017212 | 0,02596317 | 0,03252847 | 0,03046354 | 0,03064993 | 0,02710476 | 0,02528985 | 0,04558267 | 0,03800172 | 0,03557506 |
| | F7 | 0,02300777 | 0,02315707 | 0,02088151 | 0 | 0,02028859 | 0,02173649 | 0,02108201 | 0,0318501 | 0,01810919 | 0,01983472 | 0,02570746 | 0,02640687 | 0,03469168 | 0,02045736 | 0,02635034 | 0,02696107 | 0,03212957 | 0,02680663 | 0,02204081 | 0,04873209 | 0,05172709 | 0,02765606 | 0,03755083 | 0,02270891 | 0,02920553 | 0,0298734 | 0,03989285 | 0,02665562 | 0,04028708 |
| | F3 | 0,01505905 | 0,01327145 | 0,0137013 | 0,02028859 | 0 | 0,0222057 | 0,01789963 | 0,02380784 | 0,02280384 | 0,01924548 | 0,0311012 | 0,02474882 | 0,03424859 | 0,02831226 | 0,02679456 | 0,02810976 | 0,02041545 | 0,01974457 | 0,0200781 | 0,03852866 | 0,02975202 | 0,03330584 | 0,03929733 | 0,03059005 | 0,0250376 | 0,02888511 | 0,04232577 | 0,03077 | 0,0294758 |
| | Fz | 0,01433833 | 0,01871674 | 0,01618396 | 0,02173649 | 0,0222057 | 0 | 0,02635078 | 0,03054686 | 0,01970369 | 0,02040977 | 0,03070391 | 0,02623702 | 0,02701997 | 0,02265461 | 0,02809226 | 0,02655781 | 0,02419677 | 0,02441798 | 0,01503771 | 0,04810907 | 0,03009936 | 0,02827337 | 0,02964593 | 0,02235451 | 0,01608565 | 0,02648565 | 0,04417702 | 0,03241992 | 0,02940039 |
| | F4 | 0,02767851 | 0,02225178 | 0,02586109 | 0,02108201 | 0,01789963 | 0,02635078 | 0 | 0,01942686 | 0,02218778 | 0,02339125 | 0,0241538 | 0,01974685 | 0,01728292 | 0,02178171 | 0,01885408 | 0,02146235 | 0,02187059 | 0,02294964 | 0,0191415 | 0,02140455 | 0,0173496 | 0,02925521 | 0,02365949 | 0,02319314 | 0,01625772 | 0,01710494 | 0,02454476 | 0,02834183 | 0,02274353 |
| | F8 | 0,0171361 | 0,0244794 | 0,02174909 | 0,0318501 | 0,02380784 | 0,03054686 | 0,01942686 | 0 | 0,025127 | 0,02156562 | 0,02945392 | 0,03718574 | 0,02943249 | 0,02882454 | 0,03139653 | 0,02748734 | 0,01668945 | 0,02288002 | 0,03467632 | 0,04426448 | 0,04306804 | 0,01450969 | 0,02804557 | 0,02145876 | 0,03040589 | 0,04245737 | 0,03824077 | 0,03294416 | 0,02824426 |
| | FC5 | 0,02319493 | 0,01831876 | 0,02270556 | 0,01810919 | 0,02280384 | 0,01970369 | 0,02218778 | 0,025127 | 0 | 0,01892228 | 0,02821003 | 0,02924008 | 0,03008758 | 0,01386874 | 0,02903004 | 0,01786352 | 0,02545031 | 0,02630547 | 0,01550375 | 0,02679312 | 0,03632212 | 0,03073094 | 0,03096174 | 0,01186821 | 0,0274419 | 0,02781234 | 0,02483348 | 0,02249988 | 0,02912218 |
| | FC1 | 0,00979253 | 0,01633396 | 0,01293324 | 0,01983472 | 0,01924548 | 0,02040977 | 0,02339125 | 0,02156562 | 0,01892228 | 0 | 0,01960972 | 0,02113207 | 0,01989164 | 0,02290846 | 0,01629624 | 0,01570129 | 0,02993838 | 0,012963 | 0,0294307 | 0,01449351 | 0,02601726 | 0,02481073 | 0,03280635 | 0,02955372 | 0,03715766 | 0,02624785 | 0,02095581 | 0,03176793 | 0,02534696 |
| | FC2 | 0,02712889 | 0,0309211 | 0,03005198 | 0,02570746 | 0,0311012 | 0,03070391 | 0,0241538 | 0,02945392 | 0,02821003 | 0,01960972 | 0 | 0,02038646 | 0,03778303 | 0,03582094 | 0,0174843 | 0,02045549 | 0,02635333 | 0,03472859 | 0,02246314 | 0,05183394 | 0,04387108 | 0,02373353 | 0,03504386 | 0,0237017 | 0,02094902 | 0,02962024 | 0,04636576 | 0,03402164 | 0,03269931 |
| | FC6 | 0,02366616 | 0,02867914 | 0,02671294 | 0,02640687 | 0,02474882 | 0,02623702 | 0,01974685 | 0,03718574 | 0,02924008 | 0,02113207 | 0,02038646 | 0 | 0,03296234 | 0,03719352 | 0,01185847 | 0,02628468 | 0,03173247 | 0,02542428 | 0,02789562 | 0,04366706 | 0,04195987 | 0,02598747 | 0,03611608 | 0,02452462 | 0,02761149 | 0,02761749 | 0,04008883 | 0,02966354 | 0,03102409 |
| | T7 | 0,02941294 | 0,03404948 | 0,02832202 | 0,03469168 | 0,03424859 | 0,02701997 | 0,01728292 | 0,02943249 | 0,03008758 | 0,01989164 | 0,03778303 | 0,03296234 | 0 | 0,03454963 | 0,03606511 | 0,03537759 | 0,03432964 | 0,02060809 | 0,01735532 | 0,04446252 | 0,03817594 | 0,02949504 | 0,02961606 | 0,02226421 | 0,02074861 | 0,02121912 | 0,03746341 | 0,02665868 | 0,03524225 |
| | C3 | 0,02553019 | 0,02375128 | 0,02431465 | 0,02045736 | 0,02831226 | 0,02265461 | 0,02178171 | 0,02882454 | 0,01386874 | 0,02290846 | 0,03582094 | 0,03719352 | 0,03454963 | 0 | 0,03446048 | 0,03451304 | 0,02062818 | 0,0317506 | 0,01547039 | 0,02947151 | 0,03689465 | 0,03069396 | 0,03764253 | 0,02377689 | 0,02111202 | 0,02593979 | 0,03523421 | 0,02760388 | 0,04250644 |
| | Cz | 0,02852371 | 0,03247116 | 0,02637963 | 0,02635034 | 0,02679456 | 0,02809226 | 0,01885408 | 0,03139653 | 0,02903004 | 0,01629624 | 0,0174843 | 0,01185847 | 0,03606511 | 0,03446048 | 0 | 0,02562143 | 0,02902903 | 0,0289184 | 0,02516352 | 0,04203922 | 0,04534886 | 0,02408925 | 0,03627209 | 0,02401742 | 0,02589461 | 0,0291603 | 0,04250379 | 0,02644918 | 0,03355346 |
| | C4 | 0,02165873 | 0,02576686 | 0,02555824 | 0,02696107 | 0,02810976 | 0,02655781 | 0,02146235 | 0,02748734 | 0,01786352 | 0,01570129 | 0,02045549 | 0,02628468 | 0,03537759 | 0,03451304 | 0,02562143 | 0 | 0,0225267 | 0,02328443 | 0,02163799 | 0,05802157 | 0,04501528 | 0,02143525 | 0,03571722 | 0,02101274 | 0,02430855 | 0,02904007 | 0,05000869 | 0,04182699 | 0,03484884 |
| | T8 | 0,01853801 | 0,0162764 | 0,01835463 | 0,03212957 | 0,02041545 | 0,02419677 | 0,02187059 | 0,01668945 | 0,02545031 | 0,02993838 | 0,02635333 | 0,03173247 | 0,03432964 | 0,02062818 | 0,02902903 | 0,0225267 | 0 | 0,01682937 | 0,03205089 | 0,0373856 | 0,04577597 | 0,02578889 | 0,02644086 | 0,01836268 | 0,03683459 | 0,03545953 | 0,03215057 | 0,02092357 | 0,02702832 |
| | CP5 | 0,01874509 | 0,02003784 | 0,02137065 | 0,02680663 | 0,01974457 | 0,02441798 | 0,02294964 | 0,02288002 | 0,02630547 | 0,012963 | 0,03472859 | 0,02542428 | 0,02060809 | 0,0317506 | 0,0289184 | 0,02328443 | 0,01682937 | 0 | 0,01895178 | 0,03488413 | 0,0288143 | 0,03252159 | 0,02973952 | 0,02631424 | 0,02338997 | 0,02657377 | 0,03621886 | 0,03291617 | 0,0304295 |
| | CP1 | 0,02838001 | 0,02161739 | 0,02648075 | 0,02204081 | 0,0200781 | 0,01503771 | 0,0191415 | 0,03467632 | 0,01550375 | 0,0294307 | 0,02246314 | 0,02789562 | 0,01735532 | 0,01547039 | 0,02516352 | 0,02163799 | 0,03205089 | 0,01895178 | 0 | 0,02145126 | 0,03820093 | 0,01977443 | 0,01972035 | 0,02393508 | 0,02280591 | 0,03824484 | 0,02007822 | 0,01990614 | 0,01698721 |
| | CP2 | 0,03381576 | 0,03898303 | 0,04017212 | 0,04873209 | 0,03852866 | 0,04810907 | 0,02140455 | 0,04426448 | 0,02679312 | 0,01449351 | 0,05183394 | 0,04366706 | 0,04446252 | 0,02947151 | 0,04203922 | 0,05802157 | 0,0373856 | 0,03488413 | 0,02145126 | 0 | 0,03017918 | 0,01789781 | 0,03099756 | 0,02837457 | 0,02252826 | 0,01614035 | 0,03986784 | 0,02356119 | 0,03539519 |
| | CP6 | 0,02480859 | 0,03244512 | 0,02596317 | 0,05172709 | 0,02975202 | 0,03009936 | 0,0173496 | 0,04306804 | 0,03632212 | 0,02601726 | 0,04387108 | 0,04195987 | 0,03817594 | 0,03689465 | 0,04534886 | 0,04501528 | 0,04577597 | 0,0288143 | 0,03820093 | 0,03017918 | 0 | 0,01698229 | 0,03385764 | 0,01567682 | 0,03759082 | 0,02461872 | 0,03173339 | 0,01255019 | 0,02315412 |
| | P7 | 0,03146826 | 0,03129383 | 0,03252847 | 0,02765606 | 0,03330584 | 0,02827337 | 0,02925521 | 0,01450969 | 0,03073094 | 0,02481073 | 0,02373353 | 0,02598747 | 0,02949504 | 0,03069396 | 0,02408925 | 0,02143525 | 0,02578889 | 0,03252159 | 0,01977443 | 0,01789781 | 0,01698229 | 0 | 0,0276242 | 0,02424834 | 0,02403983 | 0,025304 | 0,02301474 | 0,02647 | 0,02995127 |
| | P3 | 0,03384667 | 0,03932357 | 0,03046354 | 0,03755083 | 0,03929733 | 0,02964593 | 0,02365949 | 0,02804557 | 0,03096174 | 0,03280635 | 0,03504386 | 0,03611608 | 0,02961606 | 0,03764253 | 0,03627209 | 0,03571722 | 0,02644086 | 0,02973952 | 0,01972035 | 0,03099756 | 0,03385764 | 0,0276242 | 0 | 0,01494122 | 0,02296094 | 0,02809344 | 0,02584196 | 0,01664941 | 0,03043406 |
| | Pz | 0,03054588 | 0,03802478 | 0,03064993 | 0,02270891 | 0,03059005 | 0,02235451 | 0,02319314 | 0,02145876 | 0,01186821 | 0,02955372 | 0,0237017 | 0,02452462 | 0,02226421 | 0,02377689 | 0,02401742 | 0,02101274 | 0,01836268 | 0,02631424 | 0,02393508 | 0,02837457 | 0,01567682 | 0,02424834 | 0,01494122 | 0 | 0,01728725 | 0,01542791 | 0,02670043 | 0,03445295 | 0,02208616 |
| | P4 | 0,02529288 | 0,02359379 | 0,02710476 | 0,02920553 | 0,0250376 | 0,01608565 | 0,01625772 | 0,03040589 | 0,0274419 | 0,03715766 | 0,02094902 | 0,02761149 | 0,02074861 | 0,02111202 | 0,02589461 | 0,02430855 | 0,03683459 | 0,02338997 | 0,02280591 | 0,02252826 | 0,03759082 | 0,02403983 | 0,02296094 | 0,01728725 | 0 | 0,02540524 | 0,02696909 | 0,01796876 | 0,02066747 |
| | P8 | 0,02479579 | 0,02971264 | 0,02528985 | 0,0298734 | 0,02888511 | 0,02648565 | 0,01710494 | 0,04245737 | 0,02781234 | 0,02624785 | 0,02962024 | 0,02761749 | 0,02121912 | 0,02593979 | 0,0291603 | 0,02904007 | 0,03545953 | 0,02657377 | 0,03824484 | 0,01614035 | 0,02461872 | 0,025304 | 0,02809344 | 0,01542791 | 0,02540524 | 0 | 0,01962714 | 0,02428878 | 0,02607206 |
| | POz | 0,03658789 | 0,04323255 | 0,04558267 | 0,03989285 | 0,04232577 | 0,04417702 | 0,02454476 | 0,03824077 | 0,02483348 | 0,02095581 | 0,04636576 | 0,04008883 | 0,03746341 | 0,03523421 | 0,04250379 | 0,05000869 | 0,03215057 | 0,03621886 | 0,02007822 | 0,03986784 | 0,03173339 | 0,02301474 | 0,02584196 | 0,02670043 | 0,02696909 | 0,01962714 | 0 | 0,02737291 | 0,02793831 |
| | O1 | 0,0387112 | 0,03669147 | 0,03800172 | 0,02665562 | 0,03077 | 0,03241992 | 0,02834183 | 0,03294416 | 0,02249988 | 0,03176793 | 0,03402164 | 0,02966354 | 0,02665868 | 0,02760388 | 0,02644918 | 0,04182699 | 0,02092357 | 0,03291617 | 0,01990614 | 0,02356119 | 0,01255019 | 0,02647 | 0,01664941 | 0,03445295 | 0,01796876 | 0,02428878 | 0,02737291 | 0 | 0,0208926 |
| | O2 | 0,03022218 | 0,03678563 | 0,03557506 | 0,04028708 | 0,0294758 | 0,02940039 | 0,02274353 | 0,02824426 | 0,02912218 | 0,02534696 | 0,03269931 | 0,03102409 | 0,03524225 | 0,04250644 | 0,03355346 | 0,03484884 | 0,02702832 | 0,0304295 | 0,01698721 | 0,03539519 | 0,02315412 | 0,02995127 | 0,03043406 | 0,02208616 | 0,02066747 | 0,02607206 | 0,02793831 | 0,0208926 | 0 |
| subject 2 | Fp1 | 0,0240907 | 0,02343686 | 0,02351424 | 0,02386572 | 0,02288708 | 0,02263216 | 0,0295639 | 0,02487353 | 0,01935526 | 0,03065605 | 0,02662238 | 0,02383765 | 0,01856476 | 0,01722207 | 0,02751473 | 0,02584467 | 0,02625864 | 0,02012392 | 0,01310739 | 0,02008996 | 0,02475278 | 0,02093023 | 0,01332709 | 0,02032473 | 0,01649143 | 0,02294744 | 0,01868987 | 0,01993459 | 0,01680489 |
| | Fpz | 0,02004053 | 0,02248162 | 0,02064265 | 0,02337633 | 0,02061225 | 0,01820468 | 0,02661279 | 0,02055921 | 0,01898445 | 0,02152178 | 0,0260663 | 0,02377831 | 0,02054519 | 0,02001347 | 0,02644183 | 0,02064908 | 0,02482093 | 0,02201625 | 0,0111506 | 0,01752238 | 0,02140007 | 0,02399249 | 0,01960661 | 0,02093299 | 0,01307325 | 0,01928553 | 0,01471287 | 0,0184416 | 0,01300002 |
| | Fp2 | 0,01887795 | 0,01975214 | 0,01969185 | 0,02064693 | 0,01994714 | 0,01937736 | 0,02764331 | 0,02197736 | 0,01743813 | 0,01629201 | 0,02970677 | 0,02339744 | 0,01979262 | 0,02070771 | 0,02547627 | 0,02265538 | 0,01962059 | 0,01823341 | 0,01099114 | 0,01772043 | 0,01182965 | 0,02354523 | 0,02337346 | 0,02081775 | 0,01413816 | 0,01593956 | 0,01855748 | 0,02045048 | 0,01693539 |
| | F7 | 0,01607453 | 0,01931222 | 0,01853879 | 0,02209385 | 0,02035612 | 0,01498089 | 0,02568679 | 0,01706965 | 0,0156561 | 0,01533401 | 0,02712313 | 0,02460751 | 0,01577598 | 0,01481868 | 0,02943781 | 0,02279165 | 0,02440961 | 0,01567325 | 0,01106448 | 0,01860869 | 0,01719119 | 0,02485602 | 0,0221726 | 0,01884767 | 0,01563475 | 0,01428773 | 0,01516796 | 0,01495167 | 0,01373315 |
| | F3 | 0,02004865 | 0,0261081 | 0,01936362 | 0,02416624 | 0,02057096 | 0,01858844 | 0,0331883 | 0,01344982 | 0,01548636 | 0,01813002 | 0,02785849 | 0,0265878 | 0,01870253 | 0,02207164 | 0,0273359 | 0,02874528 | 0,02170905 | 0,01741238 | 0,01498825 | 0,01936235 | 0,01067808 | 0,02251881 | 0,01856809 | 0,0219584 | 0,0166248 | 0,01703174 | 0,02496309 | 0,01536767 | 0,02414032 |
| | Fz | 0,01993199 | 0,0254834 | 0,02377184 | 0,02145155 | 0,01957619 | 0,02295509 | 0,0261368 | 0,02217437 | 0,01299328 | 0,01374065 | 0,02745672 | 0,0217724 | 0,01488324 | 0,01811101 | 0,02466289 | 0,02609412 | 0,01432776 | 0,02358898 | 0,01469218 | 0,01794013 | 0,01532908 | 0,02234744 | 0,02516603 | 0,01628417 | 0,01816529 | 0,01547667 | 0,02328987 | 0,0218058 | 0,01968901 |
| | F4 | 0,02337054 | 0,02315614 | 0,02108631 | 0,02511946 | 0,02024346 | 0,02221589 | 0,02504068 | 0,01576396 | 0,01850056 | 0,0143029 | 0,02819308 | 0,02205043 | 0,01694355 | 0,02227334 | 0,02599381 | 0,03091499 | 0,01379456 | 0,02261411 | 0,0104384 | 0,01468416 | 0,01202126 | 0,02415843 | 0,02671881 | 0,01581584 | 0,01659148 | 0,01163835 | 0,02273412 | 0,02022336 | 0,0174803 |
| | F8 | 0,01893436 | 0,0176258 | 0,01895091 | 0,01956702 | 0,01986508 | 0,01889975 | 0,03048439 | 0,01806973 | 0,02060427 | 0,01874576 | 0,02230043 | 0,018894 | 0,0190626 | 0,01923516 | 0,02533742 | 0,02334744 | 0,01509571 | 0,01741369 | 0,01530356 | 0,01793939 | 0,01496832 | 0,01710784 | 0,02548181 | 0,02779715 | 0,01591799 | 0,0164147 | 0,02177627 | 0,01673839 | 0,01872276 |
| | FC5 | 0,01835571 | 0,01792203 | 0,01758215 | 0,01777704 | 0,01561866 | 0,01685514 | 0,02717378 | 0,01763951 | 0,01632848 | 0,01709067 | 0,02220961 | 0,01759077 | 0,01297304 | 0,01474702 | 0,02321548 | 0,020802 | 0,01916888 | 0,01557873 | 0,01184179 | 0,0139476 | 0,01972706 | 0,01645121 | 0,01985839 | 0,01939162 | 0,01730089 | 0,01968944 | 0,02038664 | 0,02096181 | 0,01334269 |
| | FC1 | 0,01829583 | 0,01943692 | 0,01726462 | 0,02075977 | 0,01710928 | 0,01464661 | 0,02755779 | 0,01801617 | 0,01805832 | 0,01654734 | 0,02107356 | 0,02205362 | 0,01031819 | 0,01682227 | 0,02479211 | 0,01924144 | 0,0194006 | 0,01511755 | 0,01171418 | 0,01242384 | 0,01017158 | 0,01647309 | 0,0197854 | 0,01147084 | 0,01708095 | 0,01498987 | 0,01425798 | 0,01968909 | 0,01690174 |
| | FC2 | 0,01639429 | 0,01754275 | 0,01550509 | 0,02106051 | 0,01692923 | 0,01905407 | 0,02405843 | 0,0147727 | 0,01255257 | 0,01419597 | 0,02610491 | 0,02338235 | 0,01135861 | 0,01534442 | 0,02615586 | 0,02812122 | 0,01795716 | 0,01717539 | 0,01203592 | 0,01686366 | 0,01908991 | 0,01580109 | 0,02553258 | 0,0192283 | 0,01675991 | 0,01789446 | 0,02383706 | 0,01952532 | 0,01442181 |
| | FC6 | 0,01960984 | 0,02031827 | 0,02073383 | 0,02444178 | 0,02322866 | 0,02165533 | 0,02908781 | 0,02035025 | 0,02490823 | 0,02127896 | 0,02780073 | 0,02680506 | 0,02313322 | 0,02228714 | 0,02850831 | 0,02465493 | 0,01808759 | 0,01970676 | 0,01833831 | 0,02374921 | 0,01843312 | 0,01974434 | 0,03103921 | 0,02143451 | 0,01793391 | 0,01719433 | 0,0274633 | 0,01939149 | 0,0212285 |
| | T7 | 0,02013113 | 0,0175416 | 0,01760135 | 0,02120678 | 0,01563912 | 0,01956537 | 0,02753226 | 0,0146304 | 0,02229947 | 0,01623902 | 0,02168168 | 0,02295559 | 0,01544913 | 0,01729026 | 0,02218308 | 0,02208724 | 0,02348962 | 0,0178116 | 0,01098104 | 0,01930716 | 0,01800293 | 0,02113804 | 0,01716567 | 0,01761943 | 0,0162978 | 0,01909575 | 0,02033706 | 0,02323131 | 0,01487431 |
| | C3 | 0,01819933 | 0,01987014 | 0,02005385 | 0,02590801 | 0,01924895 | 0,01861074 | 0,02461602 | 0,01849673 | 0,02090647 | 0,01839353 | 0,02461541 | 0,02436752 | 0,01928442 | 0,01954241 | 0,02483175 | 0,02052528 | 0,02203976 | 0,01962714 | 0,01478822 | 0,01763563 | 0,01575581 | 0,01648504 | 0,01340911 | 0,01611814 | 0,01202302 | 0,01565647 | 0,01744581 | 0,02032308 | 0,01734884 |
| | Cz | 0,01698293 | 0,01925282 | 0,01650549 | 0,02138759 | 0,01770564 | 0,01563513 | 0,02647095 | 0,01669583 | 0,01708403 | 0,01917848 | 0,02065805 | 0,01948795 | 0,01320872 | 0,01802807 | 0,02598514 | 0,02104126 | 0,0234877 | 0,01249076 | 0,01512751 | 0,01611471 | 0,01373743 | 0,01292515 | 0,01876153 | 0,02203725 | 0,02078191 | 0,01945007 | 0,01764152 | 0,01771251 | 0,01523247 |
| | C4 | 0,01756176 | 0,01564866 | 0,01619746 | 0,01588474 | 0,01332459 | 0,01859935 | 0,01755065 | 0,01965801 | 0,01681099 | 0,01746172 | 0,01763379 | 0,01543029 | 0,01228949 | 0,01436032 | 0,01997137 | 0,01984833 | 0,01503623 | 0,01569821 | 0,01338884 | 0,01637137 | 0,01738241 | 0,02250204 | 0,01959354 | 0,01627647 | 0,02130639 | 0,01511128 | 0,01526583 | 0,02393161 | 0,02020316 |
| | T8 | 0,0200989 | 0,02135484 | 0,0187169 | 0,02162208 | 0,01881308 | 0,01860315 | 0,02325826 | 0,02501422 | 0,02015069 | 0,02485639 | 0,02281804 | 0,01957203 | 0,02072775 | 0,02144603 | 0,0237196 | 0,02514801 | 0,01713073 | 0,02157521 | 0,01653754 | 0,0184481 | 0,02063607 | 0,01426753 | 0,02049585 | 0,02896706 | 0,01733178 | 0,01810979 | 0,02239272 | 0,01363778 | 0,02053198 |
| | CP5 | 0,02060957 | 0,0239914 | 0,02335827 | 0,02750454 | 0,02433052 | 0,02514907 | 0,02270692 | 0,01943322 | 0,02199708 | 0,01823287 | 0,02175527 | 0,02114538 | 0,02026891 | 0,02488634 | 0,0222685 | 0,01759278 | 0,02114299 | 0,02600256 | 0,01784635 | 0,01883731 | 0,01778059 | 0,02190917 | 0,01970129 | 0,02159149 | 0,0184963 | 0,01867208 | 0,01885448 | 0,02022811 | 0,02109134 |
| | CP1 | 0,01721646 | 0,02078957 | 0,01696102 | 0,02005024 | 0,02147408 | 0,02013091 | 0,02210021 | 0,02303321 | 0,02004186 | 0,01645502 | 0,0268877 | 0,01978311 | 0,01694845 | 0,01972617 | 0,02440049 | 0,02968134 | 0,02490883 | 0,01838339 | 0,02130896 | 0,02304358 | 0,01995767 | 0,01823838 | 0,02624081 | 0,02837741 | 0,02596176 | 0,01813765 | 0,02436824 | 0,02015402 | 0,01651678 |
| | CP2 | 0,01602944 | 0,01735242 | 0,01599214 | 0,02088046 | 0,01856306 | 0,01797521 | 0,03085893 | 0,01518984 | 0,01511859 | 0,01302053 | 0,02382251 | 0,02084945 | 0,0109569 | 0,01571868 | 0,02638382 | 0,02275661 | 0,01919348 | 0,01412587 | 0,01529922 | 0,01877204 | 0,01500536 | 0,01063918 | 0,02500275 | 0,01939823 | 0,01862682 | 0,01837055 | 0,02073692 | 0,01897695 | 0,01702697 |
| | CP6 | 0,01919989 | 0,01689953 | 0,01714061 | 0,01960787 | 0,01940344 | 0,01813453 | 0,02342367 | 0,01830959 | 0,0138144 | 0,01446364 | 0,02254433 | 0,02148788 | 0,01459103 | 0,01415537 | 0,0241765 | 0,02657898 | 0,01722745 | 0,01767718 | 0,01565623 | 0,01887532 | 0,01087587 | 0,01491048 | 0,02304853 | 0,02162893 | 0,01870319 | 0,01935667 | 0,01726919 | 0,01760793 | 0,01411065 |
| | P7 | 0,02344486 | 0,02272323 | 0,0249013 | 0,02623608 | 0,02179389 | 0,02938594 | 0,02232802 | 0,02224518 | 0,01939197 | 0,02099754 | 0,02285389 | 0,02141722 | 0,02297525 | 0,0221241 | 0,02557786 | 0,02206939 | 0,02670368 | 0,02555469 | 0,0193174 | 0,02227484 | 0,01860208 | 0,01907931 | 0,02531344 | 0,02597563 | 0,02238888 | 0,01237602 | 0,02033154 | 0,02248295 | 0,0257197 |
| | P3 | 0,02652261 | 0,02368394 | 0,02762925 | 0,02773457 | 0,02210373 | 0,0265424 | 0,01902898 | 0,02604664 | 0,0171502 | 0,02673228 | 0,02445234 | 0,02196591 | 0,0266264 | 0,0221335 | 0,02190796 | 0,01926957 | 0,02229286 | 0,02683645 | 0,0190801 | 0,01973696 | 0,0184567 | 0,02271274 | 0,02052338 | 0,02701657 | 0,018334 | 0,01629261 | 0,02376207 | 0,02131505 | 0,02423037 |
| | Pz | 0,02107219 | 0,02137717 | 0,02424217 | 0,01801264 | 0,01754799 | 0,01727404 | 0,03005465 | 0,02071884 | 0,01823656 | 0,02395685 | 0,0207393 | 0,01694329 | 0,01342072 | 0,01764516 | 0,02572208 | 0,02090287 | 0,02003785 | 0,02142872 | 0,01612266 | 0,01896764 | 0,02318998 | 0,01552634 | 0,01731239 | 0,01973965 | 0,015558 | 0,02209399 | 0,01861488 | 0,01643078 | 0,01530643 |
| | P4 | 0,01932506 | 0,01996385 | 0,01837349 | 0,02036855 | 0,01792513 | 0,01638395 | 0,02375596 | 0,0184632 | 0,01934833 | 0,01755792 | 0,02253394 | 0,02133817 | 0,01323177 | 0,01895609 | 0,02936019 | 0,02568369 | 0,0217412 | 0,01341527 | 0,0167603 | 0,01924505 | 0,01323049 | 0,015164 | 0,02305523 | 0,02223825 | 0,02158974 | 0,0202705 | 0,02300724 | 0,01563394 | 0,01617395 |
| | P8 | 0,01590371 | 0,01749139 | 0,01449166 | 0,02203581 | 0,01688466 | 0,01666047 | 0,02537747 | 0,02266493 | 0,0182452 | 0,01366864 | 0,02480932 | 0,01914085 | 0,01297942 | 0,0182416 | 0,02764605 | 0,02290367 | 0,01551064 | 0,01616781 | 0,01614579 | 0,02118883 | 0,01826791 | 0,0174619 | 0,02103313 | 0,02278809 | 0,02033491 | 0,02031381 | 0,02059708 | 0,01555529 | 0,01466665 |
| | POz | 0,02043581 | 0,01893267 | 0,01936431 | 0,02158108 | 0,01646133 | 0,0165745 | 0,02547531 | 0,02163136 | 0,01595207 | 0,02308441 | 0,01868699 | 0,02040587 | 0,01392387 | 0,01694181 | 0,0259959 | 0,02092426 | 0,02132718 | 0,01788422 | 0,01915541 | 0,02001988 | 0,02112021 | 0,01562027 | 0,02009073 | 0,01921691 | 0,01487455 | 0,02014216 | 0,0196267 | 0,02056746 | 0,01917488 |
| | O1 | 0,01566025 | 0,0166678 | 0,01291791 | 0,01763226 | 0,01609642 | 0,01434234 | 0,02833005 | 0,02222347 | 0,01447363 | 0,01560765 | 0,02350479 | 0,01876566 | 0,01355215 | 0,01785678 | 0,02409301 | 0,02081122 | 0,01392787 | 0,01288341 | 0,0173871 | 0,02192039 | 0,01824096 | 0,01790842 | 0,01758902 | 0,02413565 | 0,01823474 | 0,01803395 | 0,02413378 | 0,01544777 | 0,012559 |
| | O2 | 0,01835558 | 0,01969449 | 0,01846927 | 0,01808669 | 0,01589702 | 0,01702644 | 0,02401195 | 0,01821165 | 0,01635864 | 0,01803106 | 0,02008888 | 0,01818588 | 0,01386077 | 0,01650772 | 0,02266152 | 0,02215023 | 0,02035615 | 0,01757478 | 0,01449389 | 0,01961536 | 0,02218421 | 0,01673612 | 0,01971677 | 0,01749469 | 0,01717207 | 0,01747045 | 0,02035723 | 0,01989515 | 0,01563075 |

## subject 2 (columns)

| | | Fp1 | Fpz | Fp2 | F7 | F3 | Fz | F4 | F8 | FC5 | FC1 | FC2 | FC6 | T7 | C3 | Cz | C4 | T8 | CP5 | CP1 | CP2 | CP6 | P7 | P3 | Pz | P4 | P8 | POz | O1 | O2 |
|---|---|---|---|---|---|---|---|---|---|---|---|---|---|---|---|---|---|---|---|---|---|---|---|---|---|---|---|---|---|---|
| subject 1 | Fp1 | 0,0240907 | 0,02004053 | 0,01887795 | 0,01607453 | 0,02004865 | 0,01993199 | 0,02337054 | 0,01893436 | 0,01835571 | 0,01829583 | 0,01639429 | 0,01960984 | 0,02013113 | 0,01819933 | 0,01698293 | 0,01756176 | 0,0200989 | 0,02060957 | 0,01721646 | 0,01602944 | 0,01919989 | 0,02344486 | 0,02652261 | 0,02107219 | 0,01932506 | 0,01590371 | 0,02043581 | 0,01566025 | 0,01835558 |
| | Fpz | 0,02343686 | 0,02248162 | 0,01975214 | 0,01931222 | 0,0261081 | 0,0254834 | 0,02315614 | 0,0176258 | 0,01792203 | 0,01943692 | 0,01754275 | 0,02031827 | 0,0175416 | 0,01987014 | 0,01925282 | 0,01564866 | 0,02135484 | 0,0239914 | 0,02078957 | 0,01735242 | 0,01689953 | 0,02272323 | 0,02368394 | 0,02137717 | 0,01996385 | 0,01749139 | 0,01893267 | 0,0166678 | 0,01969449 |
| | Fp2 | 0,02351424 | 0,02064265 | 0,01969185 | 0,01853879 | 0,01936362 | 0,02377184 | 0,02108631 | 0,01895091 | 0,01758215 | 0,01726462 | 0,01550509 | 0,02073383 | 0,01760135 | 0,02005385 | 0,01650549 | 0,01619746 | 0,0187169 | 0,02335827 | 0,01696102 | 0,01599214 | 0,01714061 | 0,0249013 | 0,02762925 | 0,02424217 | 0,01837349 | 0,01449166 | 0,01936431 | 0,01291791 | 0,01846927 |
| | F7 | 0,02386572 | 0,02337633 | 0,02064693 | 0,02209385 | 0,02416624 | 0,02145155 | 0,02511946 | 0,01956702 | 0,01777704 | 0,02075977 | 0,02106051 | 0,02444178 | 0,02120678 | 0,02590801 | 0,02138759 | 0,01588474 | 0,02162208 | 0,02750454 | 0,02005024 | 0,02088046 | 0,01960787 | 0,02623608 | 0,02773457 | 0,01801264 | 0,02036855 | 0,02203581 | 0,02158108 | 0,01763226 | 0,01808669 |
| | F3 | 0,02288708 | 0,02061225 | 0,01994714 | 0,02035612 | 0,02057096 | 0,01957619 | 0,02024346 | 0,01986508 | 0,01561866 | 0,01710928 | 0,01692923 | 0,02322866 | 0,01563912 | 0,01924895 | 0,01770564 | 0,01332459 | 0,01881308 | 0,02433052 | 0,02147408 | 0,01856306 | 0,01940344 | 0,02179389 | 0,02210373 | 0,01754799 | 0,01792513 | 0,01688466 | 0,01646133 | 0,01609642 | 0,01589702 |
| | Fz | 0,02263216 | 0,01820468 | 0,01937736 | 0,01498089 | 0,01858844 | 0,02295509 | 0,02221589 | 0,01889975 | 0,01685514 | 0,01464661 | 0,01905407 | 0,02165533 | 0,01956537 | 0,01861074 | 0,01563513 | 0,01859935 | 0,01860315 | 0,02514907 | 0,02013091 | 0,01797521 | 0,01813453 | 0,02938594 | 0,0265424 | 0,01727404 | 0,01638395 | 0,01666047 | 0,0165745 | 0,01434234 | 0,01702644 |
| | F4 | 0,0295639 | 0,02661279 | 0,02764331 | 0,02568679 | 0,0331883 | 0,0261368 | 0,02504068 | 0,03048439 | 0,02717378 | 0,02755779 | 0,02405843 | 0,02908781 | 0,02753226 | 0,02461602 | 0,02647095 | 0,01755065 | 0,02325826 | 0,02270692 | 0,02210021 | 0,03085893 | 0,02342367 | 0,02232802 | 0,01902898 | 0,03005465 | 0,02375596 | 0,02537747 | 0,02547531 | 0,02833005 | 0,02401195 |
| | F8 | 0,02487353 | 0,02055921 | 0,02197736 | 0,01706965 | 0,01344982 | 0,02217437 | 0,01576396 | 0,01806973 | 0,01763951 | 0,01801617 | 0,0147727 | 0,02035025 | 0,0146304 | 0,01849673 | 0,01669583 | 0,01965801 | 0,02501422 | 0,01943322 | 0,02303321 | 0,01518984 | 0,01830959 | 0,02224518 | 0,02604664 | 0,02071884 | 0,0184632 | 0,02266493 | 0,02163136 | 0,02222347 | 0,01821165 |
| | FC5 | 0,01935526 | 0,01898445 | 0,01743813 | 0,0156561 | 0,01548636 | 0,01299328 | 0,01850056 | 0,02060427 | 0,01632848 | 0,01805832 | 0,01255257 | 0,02490823 | 0,02229947 | 0,02090647 | 0,01708403 | 0,01681099 | 0,02015069 | 0,02199708 | 0,02004186 | 0,01511859 | 0,0138144 | 0,01939197 | 0,0171502 | 0,01823656 | 0,01934833 | 0,0182452 | 0,01595207 | 0,01447363 | 0,01635864 |
| | FC1 | 0,03065605 | 0,02152178 | 0,01629201 | 0,01533401 | 0,01813002 | 0,01374065 | 0,0143029 | 0,01874576 | 0,01709067 | 0,01654734 | 0,01419597 | 0,02127896 | 0,01623902 | 0,01839353 | 0,01917848 | 0,01746172 | 0,02485639 | 0,01823287 | 0,01645502 | 0,01302053 | 0,01446364 | 0,02099754 | 0,02673228 | 0,02395685 | 0,01755792 | 0,01366864 | 0,02308441 | 0,01560765 | 0,01803106 |
| | FC2 | 0,02662238 | 0,0260663 | 0,02970677 | 0,02712313 | 0,02785849 | 0,02745672 | 0,02819308 | 0,02230043 | 0,02220961 | 0,02107356 | 0,02610491 | 0,02780073 | 0,02168168 | 0,02461541 | 0,02065805 | 0,01763379 | 0,02281804 | 0,02175527 | 0,0268877 | 0,02382251 | 0,02254433 | 0,02285389 | 0,02445234 | 0,0207393 | 0,02253394 | 0,02480932 | 0,01868699 | 0,02350479 | 0,02008888 |
| | FC6 | 0,02383765 | 0,02377831 | 0,02339744 | 0,02460751 | 0,0265878 | 0,0217724 | 0,02205043 | 0,018894 | 0,01759077 | 0,02205362 | 0,02338235 | 0,02680506 | 0,02295559 | 0,02436752 | 0,01948795 | 0,01543029 | 0,01957203 | 0,02114538 | 0,01978311 | 0,02084945 | 0,02148788 | 0,02141722 | 0,02196591 | 0,01694329 | 0,02133817 | 0,01914085 | 0,02040587 | 0,01876566 | 0,01818588 |
| | T7 | 0,01856476 | 0,02054519 | 0,01979262 | 0,01577598 | 0,01870253 | 0,01488324 | 0,01694355 | 0,0190626 | 0,01297304 | 0,01031819 | 0,01135861 | 0,02313322 | 0,01544913 | 0,01928442 | 0,01320872 | 0,01228949 | 0,02072775 | 0,02026891 | 0,01694845 | 0,0109569 | 0,01459103 | 0,02297525 | 0,0266264 | 0,01342072 | 0,01323177 | 0,01297942 | 0,01392387 | 0,01355215 | 0,01386077 |
| | C3 | 0,01722207 | 0,02001347 | 0,02070771 | 0,01481868 | 0,02207164 | 0,01811101 | 0,02227334 | 0,01923516 | 0,01474702 | 0,01682227 | 0,01534442 | 0,02228714 | 0,01729026 | 0,01954241 | 0,01802807 | 0,01436032 | 0,02144603 | 0,02488634 | 0,01972617 | 0,01571868 | 0,01415537 | 0,0221241 | 0,0221335 | 0,01764516 | 0,01895609 | 0,0182416 | 0,01694181 | 0,01785678 | 0,01650772 |
| | Cz | 0,02751473 | 0,02644183 | 0,02547627 | 0,02943781 | 0,0273359 | 0,02466289 | 0,02599381 | 0,02533742 | 0,02321548 | 0,02479211 | 0,02615586 | 0,02850831 | 0,02218308 | 0,02483175 | 0,02598514 | 0,01997137 | 0,0237196 | 0,0222685 | 0,02440049 | 0,02638382 | 0,0241765 | 0,02557786 | 0,02190796 | 0,02572208 | 0,02936019 | 0,02764605 | 0,0259959 | 0,02409301 | 0,02266152 |
| | C4 | 0,02584467 | 0,02064908 | 0,02265538 | 0,02279165 | 0,02874528 | 0,02609412 | 0,03091499 | 0,02334744 | 0,020802 | 0,01924144 | 0,02812122 | 0,02465493 | 0,02208724 | 0,02052528 | 0,02104126 | 0,01984833 | 0,02514801 | 0,01759278 | 0,02968134 | 0,02275661 | 0,02657898 | 0,02206939 | 0,01926957 | 0,02090287 | 0,02568369 | 0,02290367 | 0,02092426 | 0,02081122 | 0,02215023 |
| | T8 | 0,02625864 | 0,02482093 | 0,01962059 | 0,02440961 | 0,02170905 | 0,01432776 | 0,01379456 | 0,01509571 | 0,01916888 | 0,0194006 | 0,01795716 | 0,01808759 | 0,02348962 | 0,02203976 | 0,0234877 | 0,01503623 | 0,01713073 | 0,02114299 | 0,02490883 | 0,01919348 | 0,01722745 | 0,02670368 | 0,02229286 | 0,02003785 | 0,0217412 | 0,01551064 | 0,02132718 | 0,01392787 | 0,02035615 |
| | CP5 | 0,02012392 | 0,02201625 | 0,01823341 | 0,01567325 | 0,01741238 | 0,02358898 | 0,02261411 | 0,01741369 | 0,01557873 | 0,01511755 | 0,01717539 | 0,01970676 | 0,0178116 | 0,01962714 | 0,01249076 | 0,01569821 | 0,02157521 | 0,02600256 | 0,01838339 | 0,01412587 | 0,01767718 | 0,02555469 | 0,02683645 | 0,02142872 | 0,01341527 | 0,01616781 | 0,01788422 | 0,01288341 | 0,01757478 |
| | CP1 | 0,01310739 | 0,0111506 | 0,01099114 | 0,01106448 | 0,01498825 | 0,01469218 | 0,0104384 | 0,01530356 | 0,01184179 | 0,01171418 | 0,01203592 | 0,01833831 | 0,01098104 | 0,01478822 | 0,01512751 | 0,01338884 | 0,01653754 | 0,01784635 | 0,02130896 | 0,01529922 | 0,01565623 | 0,0193174 | 0,0190801 | 0,01612266 | 0,0167603 | 0,01614579 | 0,01915541 | 0,0173871 | 0,01449389 |
| | CP2 | 0,02008996 | 0,01752238 | 0,01772043 | 0,01860869 | 0,01936235 | 0,01794013 | 0,01468416 | 0,01793939 | 0,0139476 | 0,01242384 | 0,01686366 | 0,02374921 | 0,01930716 | 0,01763563 | 0,01611471 | 0,01637137 | 0,0184481 | 0,01883731 | 0,02304358 | 0,01877204 | 0,01887532 | 0,02227484 | 0,01973696 | 0,01896764 | 0,01924505 | 0,02118883 | 0,02001988 | 0,02192039 | 0,01961536 |
| | CP6 | 0,02475278 | 0,02140007 | 0,01182965 | 0,01719119 | 0,01067808 | 0,01532908 | 0,01202126 | 0,01496832 | 0,01972706 | 0,01017158 | 0,01908991 | 0,01843312 | 0,01800293 | 0,01575581 | 0,01373743 | 0,01738241 | 0,02063607 | 0,01778059 | 0,01995767 | 0,01500536 | 0,01087587 | 0,01860208 | 0,0184567 | 0,02318998 | 0,01323049 | 0,01826791 | 0,02112021 | 0,01824096 | 0,02218421 |
| | P7 | 0,02093023 | 0,02399249 | 0,02354523 | 0,02485602 | 0,02251881 | 0,02234744 | 0,02415843 | 0,01710784 | 0,01645121 | 0,01647309 | 0,01580109 | 0,01974434 | 0,02113804 | 0,01648504 | 0,01292515 | 0,02250204 | 0,01426753 | 0,02190917 | 0,01823838 | 0,01063918 | 0,01491048 | 0,01907931 | 0,02271274 | 0,01552634 | 0,015164 | 0,0174619 | 0,01562027 | 0,01790842 | 0,01673612 |
| | P3 | 0,01332709 | 0,01960661 | 0,02337346 | 0,0221726 | 0,01856809 | 0,02516603 | 0,02671881 | 0,02548181 | 0,01985839 | 0,0197854 | 0,02553258 | 0,03103921 | 0,01716567 | 0,01340911 | 0,01876153 | 0,01959354 | 0,02049585 | 0,01970129 | 0,02624081 | 0,02500275 | 0,02304853 | 0,02531344 | 0,02052338 | 0,01731239 | 0,02305523 | 0,02103313 | 0,02009073 | 0,01758902 | 0,01971677 |
| | Pz | 0,02032473 | 0,02093299 | 0,02081775 | 0,01884767 | 0,0219584 | 0,01628417 | 0,01581584 | 0,02779715 | 0,01939162 | 0,01147084 | 0,0192283 | 0,02143451 | 0,01761943 | 0,01611814 | 0,02203725 | 0,01627647 | 0,02896706 | 0,02159149 | 0,02837741 | 0,01939823 | 0,02162893 | 0,02597563 | 0,02701657 | 0,01973965 | 0,02223825 | 0,02278809 | 0,01921691 | 0,02413565 | 0,01749469 |
| | P4 | 0,01649143 | 0,01307325 | 0,01413816 | 0,01563475 | 0,0166248 | 0,01816529 | 0,01659148 | 0,01591799 | 0,01730089 | 0,01708095 | 0,01675991 | 0,01793391 | 0,0162978 | 0,01202302 | 0,02078191 | 0,02130639 | 0,01733178 | 0,0184963 | 0,02596176 | 0,01862682 | 0,01870319 | 0,02238888 | 0,018334 | 0,015558 | 0,02158974 | 0,02033491 | 0,01487455 | 0,01823474 | 0,01717207 |
| | P8 | 0,02294744 | 0,01928553 | 0,01593956 | 0,01428773 | 0,01703174 | 0,01547667 | 0,01163835 | 0,0164147 | 0,01968944 | 0,01498987 | 0,01789446 | 0,01719433 | 0,01909575 | 0,01565647 | 0,01945007 | 0,01511128 | 0,01810979 | 0,01867208 | 0,01813765 | 0,01837055 | 0,01935667 | 0,01237602 | 0,01629261 | 0,02209399 | 0,0202705 | 0,02031381 | 0,02014216 | 0,01803395 | 0,01747045 |
| | POz | 0,01868987 | 0,01471287 | 0,01855748 | 0,01516796 | 0,02496309 | 0,02328987 | 0,02273412 | 0,02177627 | 0,02038664 | 0,01425798 | 0,02383706 | 0,0274633 | 0,02033706 | 0,01744581 | 0,01764152 | 0,01526583 | 0,02239272 | 0,01885448 | 0,02436824 | 0,02073692 | 0,01726919 | 0,02033154 | 0,02376207 | 0,01861488 | 0,02300724 | 0,02059708 | 0,0196267 | 0,02413378 | 0,02035723 |
| | O1 | 0,01993459 | 0,0184416 | 0,02045048 | 0,01495167 | 0,01536767 | 0,0218058 | 0,02022336 | 0,01673839 | 0,02096181 | 0,01968909 | 0,01952532 | 0,01939149 | 0,02323131 | 0,02032308 | 0,01771251 | 0,02393161 | 0,01363778 | 0,02022811 | 0,02015402 | 0,01897695 | 0,01760793 | 0,02248295 | 0,02131505 | 0,01643078 | 0,01563394 | 0,01555529 | 0,02056746 | 0,01544777 | 0,01989515 |
| | O2 | 0,01680489 | 0,01300002 | 0,01693539 | 0,01373315 | 0,02414032 | 0,01968901 | 0,0174803 | 0,01872276 | 0,01334269 | 0,01690174 | 0,01442181 | 0,0212285 | 0,01487431 | 0,01734884 | 0,01523247 | 0,02020316 | 0,02053198 | 0,02109134 | 0,01651678 | 0,01702697 | 0,01411065 | 0,0257197 | 0,02423037 | 0,01530643 | 0,01617395 | 0,01466665 | 0,01917488 | 0,012559 | 0,01563075 |
| subject 2 | Fp1 | 0 | 0,0223955 | 0,03170051 | 0,02308837 | 0,03187272 | 0,0381127 | 0,04286024 | 0,03688047 | 0,04237355 | 0,03688831 | 0,04318784 | 0,03912113 | 0,04009479 | 0,03684339 | 0,035083 | 0,02713467 | 0,02615366 | 0,02695965 | 0,0384279 | 0,03868544 | 0,03441716 | 0,02627412 | 0,02362441 | 0,03298218 | 0,03192645 | 0,03041897 | 0,02971265 | 0,03088119 | 0,03214341 |
| | Fpz | 0,0223955 | 0 | 0,02625335 | 0,01568705 | 0,03974413 | 0,04499762 | 0,03578358 | 0,04195621 | 0,03957977 | 0,03881219 | 0,04901509 | 0,04464409 | 0,04053644 | 0,04195551 | 0,03789872 | 0,0331846 | 0,03249442 | 0,03072456 | 0,04264897 | 0,03944203 | 0,03832679 | 0,02576605 | 0,02201552 | 0,0319483 | 0,03910997 | 0,03280362 | 0,03075644 | 0,03367404 | 0,02987829 |
| | Fp2 | 0,03170051 | 0,02625335 | 0 | 0,02295325 | 0,03199618 | 0,03786359 | 0,03327392 | 0,03182258 | 0,03159555 | 0,03251354 | 0,03386971 | 0,03483948 | 0,02931567 | 0,03566748 | 0,03339638 | 0,03040426 | 0,03318137 | 0,02654486 | 0,04204977 | 0,02982731 | 0,03086059 | 0,02517877 | 0,01964127 | 0,03311193 | 0,03510946 | 0,03054106 | 0,03256442 | 0,03204129 | 0,03337302 |
| | F7 | 0,02308837 | 0,01568705 | 0,02295325 | 0 | 0,03781789 | 0,04088656 | 0,03560484 | 0,0387798 | 0,04135726 | 0,04161412 | 0,04153809 | 0,04324657 | 0,03246362 | 0,04382822 | 0,03571048 | 0,02652769 | 0,03471912 | 0,03463637 | 0,03829888 | 0,0336421 | 0,03481352 | 0,02959231 | 0,02849382 | 0,04010952 | 0,03974004 | 0,03231245 | 0,03303046 | 0,03400173 | 0,03066629 |
| | F3 | 0,03187272 | 0,03974413 | 0,03199618 | 0,03781789 | 0 | 0,03039504 | 0,03583046 | 0,04676647 | 0,04854056 | 0,03042318 | 0,03658501 | 0,0439544 | 0,03781912 | 0,03292978 | 0,05112368 | 0,03490857 | 0,03968407 | 0,0327492 | 0,04131718 | 0,0404504 | 0,03910591 | 0,02951744 | 0,02549615 | 0,04062684 | 0,04724914 | 0,04611932 | 0,03893142 | 0,04295195 | 0,04288064 |
| | Fz | 0,0381127 | 0,04499762 | 0,03786359 | 0,04088656 | 0,03039504 | 0 | 0,0139652 | 0,03548615 | 0,03605308 | 0,01982254 | 0,01892872 | 0,03291736 | 0,03230651 | 0,02477752 | 0,02837866 | 0,04015601 | 0,04140747 | 0,03315855 | 0,03837187 | 0,03087622 | 0,04202206 | 0,03254776 | 0,02612272 | 0,03248544 | 0,03529181 | 0,04178972 | 0,0374347 | 0,03760679 | 0,04007329 |
| | F4 | 0,04286024 | 0,03578358 | 0,03327392 | 0,03560484 | 0,03583046 | 0,0139652 | 0 | 0,03584251 | 0,03364169 | 0,01445009 | 0,01967787 | 0,02992921 | 0,03093128 | 0,02736226 | 0,02633845 | 0,03750264 | 0,03982382 | 0,03381763 | 0,03625838 | 0,02266312 | 0,03164649 | 0,02983849 | 0,02617741 | 0,03213521 | 0,03184299 | 0,03413802 | 0,03487477 | 0,03933237 | 0,0352842 |
| | F8 | 0,03688047 | 0,04195621 | 0,03182258 | 0,0387798 | 0,04676647 | 0,03548615 | 0,03584251 | 0 | 0,02315751 | 0,04058899 | 0,0318612 | 0,02041144 | 0,0179275 | 0,03273921 | 0,03023449 | 0,0219963 | 0,01899494 | 0,01865076 | 0,03638339 | 0,02981566 | 0,03531234 | 0,02874725 | 0,02189787 | 0,03454404 | 0,03283467 | 0,02785966 | 0,02906057 | 0,02900253 | 0,02956022 |
| | FC5 | 0,04237355 | 0,03957977 | 0,03159555 | 0,04135726 | 0,04854056 | 0,03605308 | 0,03364169 | 0,02315751 | 0 | 0,03768251 | 0,03000452 | 0,02960613 | 0,01200973 | 0,02852844 | 0,02008368 | 0,02721143 | 0,0282864 | 0,02112953 | 0,03606603 | 0,01757456 | 0,03063247 | 0,02863325 | 0,02759106 | 0,03103854 | 0,02693691 | 0,02407642 | 0,02368776 | 0,02675972 | 0,02416087 |
| | FC1 | 0,03688831 | 0,03881219 | 0,03251354 | 0,04161412 | 0,03042318 | 0,01982254 | 0,01445009 | 0,04058899 | 0,03768251 | 0 | 0,02445738 | 0,03337667 | 0,03782033 | 0,0205145 | 0,0283809 | 0,03003797 | 0,03417222 | 0,03330443 | 0,0377861 | 0,02440915 | 0,03763109 | 0,0320635 | 0,03088825 | 0,02944363 | 0,03682501 | 0,04233429 | 0,03426188 | 0,03598139 | 0,03710957 |
| | FC2 | 0,04318784 | 0,04901509 | 0,03386971 | 0,04153809 | 0,03658501 | 0,01892872 | 0,01967787 | 0,0318612 | 0,03000452 | 0,02445738 | 0 | 0,02937858 | 0,0308529 | 0,03036404 | 0,02760737 | 0,02872774 | 0,03602733 | 0,03591245 | 0,03448632 | 0,02081461 | 0,03118907 | 0,02415328 | 0,02354569 | 0,03501947 | 0,02919595 | 0,03413237 | 0,03781043 | 0,03734501 | 0,03766475 |
| | FC6 | 0,03912113 | 0,04464409 | 0,03483948 | 0,04324657 | 0,0439544 | 0,03291736 | 0,02992921 | 0,02041144 | 0,02960613 | 0,03337667 | 0,02937858 | 0 | 0,02486914 | 0,02756804 | 0,03563086 | 0,03223913 | 0,01895524 | 0,0190788 | 0,03944027 | 0,02826262 | 0,04025753 | 0,02216127 | 0,01544467 | 0,02695114 | 0,03261272 | 0,0307041 | 0,02533087 | 0,03059892 | 0,02972701 |
| | T7 | 0,04009479 | 0,04053644 | 0,02931567 | 0,03246362 | 0,03781912 | 0,03230651 | 0,03093128 | 0,0179275 | 0,01200973 | 0,03782033 | 0,0308529 | 0,02486914 | 0 | 0,02995152 | 0,02663387 | 0,03288408 | 0,0222096 | 0,01860113 | 0,03300965 | 0,02459383 | 0,03653338 | 0,02748699 | 0,02912145 | 0,0322584 | 0,03124705 | 0,03061306 | 0,02579472 | 0,032963 | 0,03211475 |
| | C3 | 0,03684339 | 0,04195551 | 0,03566748 | 0,04382822 | 0,03292978 | 0,02477752 | 0,02736226 | 0,03273921 | 0,02852844 | 0,0205145 | 0,03036404 | 0,02756804 | 0,02995152 | 0 | 0,02575836 | 0,02772467 | 0,02669632 | 0,02884377 | 0,03323608 | 0,03009436 | 0,04244397 | 0,03144402 | 0,02924505 | 0,03102488 | 0,03536516 | 0,02951942 | 0,02655919 | 0,03220785 | 0,0348819 |
| | Cz | 0,035083 | 0,03789872 | 0,03339638 | 0,03571048 | 0,05112368 | 0,02837866 | 0,02633845 | 0,03023449 | 0,02008368 | 0,0283809 | 0,02760737 | 0,03563086 | 0,02663387 | 0,02575836 | 0 | 0,02264525 | 0,02374416 | 0,02738278 | 0,03057936 | 0,02799442 | 0,02290099 | 0,02828993 | 0,02465223 | 0,02570224 | 0,03166889 | 0,03410889 | 0,02748274 | 0,03178233 | 0,02449478 |
| | C4 | 0,02713467 | 0,0331846 | 0,03040426 | 0,02652769 | 0,03490857 | 0,04015601 | 0,03750264 | 0,0219963 | 0,02721143 | 0,03003797 | 0,02872774 | 0,03223913 | 0,03288408 | 0,02772467 | 0,02264525 | 0 | 0,01904533 | 0,02380174 | 0,02047881 | 0,02989159 | 0,02965116 | 0,0244715 | 0,02389425 | 0,03058649 | 0,02401467 | 0,02275648 | 0,03199288 | 0,02339839 | 0,02574625 |
| | T8 | 0,02615366 | 0,03249442 | 0,03318137 | 0,03471912 | 0,03968407 | 0,04140747 | 0,03982382 | 0,01899494 | 0,0282864 | 0,03417222 | 0,03602733 | 0,01895524 | 0,0222096 | 0,02669632 | 0,02374416 | 0,01904533 | 0 | 0,02003497 | 0,03623367 | 0,02606647 | 0,03105566 | 0,02268988 | 0,01979684 | 0,02768363 | 0,02598289 | 0,03080969 | 0,02359334 | 0,02667671 | 0,02241283 |
| | CP5 | 0,02695965 | 0,03072456 | 0,02654486 | 0,03463637 | 0,0327492 | 0,03315855 | 0,03381763 | 0,01865076 | 0,02112953 | 0,03330443 | 0,03591245 | 0,0190788 | 0,01860113 | 0,02884377 | 0,02738278 | 0,02380174 | 0,02003497 | 0 | 0,02568437 | 0,02741273 | 0,02619996 | 0,02402657 | 0,019391 | 0,03467069 | 0,0247431 | 0,0221216 | 0,02603843 | 0,02301111 | 0,03070244 |
| | CP1 | 0,0384279 | 0,04264897 | 0,04204977 | 0,03829888 | 0,04131718 | 0,03837187 | 0,03625838 | 0,03638339 | 0,03606603 | 0,0377861 | 0,03448632 | 0,03944027 | 0,03300965 | 0,03323608 | 0,03057936 | 0,02047881 | 0,03623367 | 0,02568437 | 0 | 0,02478899 | 0,02565993 | 0,02446962 | 0,02876773 | 0,0400958 | 0,02136707 | 0,03245989 | 0,04119869 | 0,02869957 | 0,03858184 |
| | CP2 | 0,03868544 | 0,03944203 | 0,02982731 | 0,0336421 | 0,0404504 | 0,03087622 | 0,02266312 | 0,02981566 | 0,01757456 | 0,02440915 | 0,02081461 | 0,02826262 | 0,02459383 | 0,03009436 | 0,02799442 | 0,02989159 | 0,02606647 | 0,02741273 | 0,02478899 | 0 | 0,02443395 | 0,01996232 | 0,02086971 | 0,02867361 | 0,02739198 | 0,0303145 | 0,02933589 | 0,03161054 | 0,02729453 |
| | CP6 | 0,03441716 | 0,03832679 | 0,03086059 | 0,03481352 | 0,03910591 | 0,04202206 | 0,03164649 | 0,03531234 | 0,03063247 | 0,03763109 | 0,03118907 | 0,04025753 | 0,03653338 | 0,04244397 | 0,02290099 | 0,02965116 | 0,03105566 | 0,02619996 | 0,02565993 | 0,02443395 | 0 | 0,0223604 | 0,02287087 | 0,03735158 | 0,02691773 | 0,02131506 | 0,03906962 | 0,02817592 | 0,03724579 |
| | P7 | 0,02627412 | 0,02576605 | 0,02517877 | 0,02959231 | 0,02951744 | 0,03254776 | 0,02983849 | 0,02874725 | 0,02863325 | 0,0320635 | 0,02415328 | 0,02216127 | 0,02748699 | 0,03144402 | 0,02828993 | 0,0244715 | 0,02268988 | 0,02402657 | 0,02446962 | 0,01996232 | 0,0223604 | 0 | 0,01076162 | 0,03676661 | 0,02159789 | 0,0207895 | 0,03049455 | 0,02064708 | 0,03810988 |
| | P3 | 0,02362441 | 0,02201552 | 0,01964127 | 0,02849382 | 0,02549615 | 0,02612272 | 0,02617741 | 0,02189787 | 0,02759106 | 0,03088825 | 0,02354569 | 0,01544467 | 0,02912145 | 0,02924505 | 0,02465223 | 0,02389425 | 0,01979684 | 0,019391 | 0,02876773 | 0,02086971 | 0,02287087 | 0,01076162 | 0 | 0,03112501 | 0,02183498 | 0,02170876 | 0,02940408 | 0,02345694 | 0,02885783 |
| | Pz | 0,03298218 | 0,0319483 | 0,03311193 | 0,04010952 | 0,04062684 | 0,03248544 | 0,03213521 | 0,03454404 | 0,03103854 | 0,02944363 | 0,03501947 | 0,02695114 | 0,0322584 | 0,03102488 | 0,02570224 | 0,03058649 | 0,02768363 | 0,03467069 | 0,0400958 | 0,02867361 | 0,03735158 | 0,03676661 | 0,03112501 | 0 | 0,03057554 | 0,03412522 | 0,01645828 | 0,03172264 | 0,01995096 |
| | P4 | 0,03192645 | 0,03910997 | 0,03510946 | 0,03974004 | 0,04724914 | 0,03529181 | 0,03184299 | 0,03283467 | 0,02693691 | 0,03682501 | 0,02919595 | 0,03261272 | 0,03124705 | 0,03536516 | 0,03166889 | 0,02401467 | 0,02598289 | 0,0247431 | 0,02136707 | 0,02739198 | 0,02691773 | 0,02159789 | 0,02183498 | 0,03057554 | 0 | 0,02738045 | 0,03406151 | 0,02639879 | 0,02911747 |
| | P8 | 0,03041897 | 0,03280362 | 0,03054106 | 0,03231245 | 0,04611932 | 0,04178972 | 0,03413802 | 0,02785966 | 0,02407642 | 0,04233429 | 0,03413237 | 0,0307041 | 0,03061306 | 0,02951942 | 0,03410889 | 0,02275648 | 0,03080969 | 0,0221216 | 0,03245989 | 0,0303145 | 0,02131506 | 0,0207895 | 0,02170876 | 0,03412522 | 0,02738045 | 0 | 0,03237049 | 0,02168688 | 0,02821428 |
| | POz | 0,02971265 | 0,03075644 | 0,03256442 | 0,03303046 | 0,03893142 | 0,0374347 | 0,03487477 | 0,02906057 | 0,02368776 | 0,03426188 | 0,03781043 | 0,02533087 | 0,02579472 | 0,02655919 | 0,02748274 | 0,03199288 | 0,02359334 | 0,02603843 | 0,04119869 | 0,02933589 | 0,03906962 | 0,03049455 | 0,02940408 | 0,01645828 | 0,03406151 | 0,03237049 | 0 | 0,03217779 | 0,0130023 |
| | O1 | 0,03088119 | 0,03367404 | 0,03204129 | 0,03400173 | 0,04295195 | 0,03760679 | 0,03933237 | 0,02900253 | 0,02675972 | 0,03598139 | 0,03734501 | 0,03059892 | 0,032963 | 0,03220785 | 0,03178233 | 0,02339839 | 0,02667671 | 0,02301111 | 0,02869957 | 0,03161054 | 0,02817592 | 0,02064708 | 0,02345694 | 0,03172264 | 0,02639879 | 0,02168688 | 0,03217779 | 0 | 0,02644431 |
| | O2 | 0,03214341 | 0,02987829 | 0,03337302 | 0,03066629 | 0,04288064 | 0,04007329 | 0,0352842 | 0,02956022 | 0,02416087 | 0,03710957 | 0,03766475 | 0,02972701 | 0,03211475 | 0,0348819 | 0,02449478 | 0,02574625 | 0,02241283 | 0,03070244 | 0,03858184 | 0,02729453 | 0,03724579 | 0,03810988 | 0,02885783 | 0,01995096 | 0,02911747 | 0,02821428 | 0,0130023 | 0,02644431 | 0 |
