# Supplementary material for: Hyperbrain features of team mental models within a juggling paradigm: a proof of concept
Source: PeerJ. 2016 Sep 20;4:e2457. doi: 10.7717/peerj.2457 (PMC5036110; doi:10.7717/peerj.2457)
Supplement: Supplemental Information 7 [file peerj-04-2457-s007.pdf]

subject 1

		Fp1	Fpz	Fp2	F7	F3	Fz	F4	F8	FC5	FC1	FC2	FC6	T7	C3	Cz	C4	T8	CP5	CP1	CP2	CP6	P7	P3	Pz	P4	P8	POz	O1	O2
subject 1	Fp1	0	0,00800892	0,00582781	0,02670181	0,02519163	0,01483241	0,00915072	0,04540086	0,0286007	0,02304253	0,02463801	0,02456565	0,01568634	0,03196792	0,02248909	0,02803667	0,02845448	0,005902	0,04328967	0,02561642	0,03838712	0,01509998	0,01810211	0,02176639	0,03391269	0,03500939	0,01914046	0,01940896	0,02845733
	Fpz	0,00800892	0	0,00890637	0,03478297	0,0213421	0,01423209	0,00658041	0,04317025	0,0396984	0,019596	0,02673512	0,02731126	0,00948382	0,04334421	0,0269558	0,02673168	0,02100626	0,0056533	0,03020755	0,01726056	0,02792966	0,01147297	0,01624123	0,0158248	0,02126918	0,02715358	0,01959916	0,02253929	0,02926466
	Fp2	0,00582781	0,00890637	0	0,0279997	0,02291136	0,01704516	0,00997796	0,06014432	0,02607901	0,0279282	0,02376087	0,02179928	0,01664223	0,02939642	0,02264187	0,02998542	0,02760332	0,00723676	0,04378379	0,02435607	0,03283334	0,01368141	0,02135416	0,019938	0,0319958	0,03514702	0,02403357	0,01882073	0,03009197
	F7	0,02670181	0,03478297	0,0279997	0	0,02624179	0,02987475	0,02061444	0,01880299	0,03382195	0,01266217	0,03399118	0,01165186	0,0271238	0,0708592	0,00944589	0,02587288	0,02662021	0,02954825	0,01452339	0,02324307	0,01472444	0,02042856	0,01575108	0,01586501	0,01637174	0,01131727	0,02330907	0,02414449	0,01511439
	F3	0,02519163	0,0213421	0,02291136	0,02624179	0	0,0205794	0,01602969	0,06091949	0,03006948	0,02687832	0,02869169	0,01822018	0,0170688	0,02807074	0,02139091	0,02890123	0,02736186	0,01911403	0,03923612	0,02407893	0,02676551	0,0133651	0,0165919	0,01356344	0,0298538	0,03464984	0,02773674	0,01295363	0,02989066
	Fz	0,01483241	0,01423209	0,01704516	0,02987475	0,0205794	0	0,00964692	0,0252712	0,03005981	0,02241307	0,02660918	0,022076	0,00986898	0,03244883	0,02720669	0,02980954	0,01897752	0,01299877	0,01989986	0,02785593	0,01491918	0,00798791	0,01406455	0,02318409	0,01707656	0,0193782	0,03377181	0,01648199	0,03343951
	F4	0,00915072	0,00658041	0,00997796	0,02061444	0,01602969	0,00964692	0	0,05530155	0,02566425	0,01295187	0,02822434	0,02242063	0,01567234	0,02845795	0,01794876	0,01980686	0,02597847	0,00358061	0,03492521	0,01974912	0,02757169	0,01969836	0,02215483	0,0217314	0,02667603	0,03224545	0,0243758	0,02178536	0,02968238
	F8	0,04540086	0,04317025	0,06014432	0,01880299	0,06091949	0,0252712	0,05530155	0	0,03518777	0,05939364	0,03621168	0,02463749	0,02247639	0,03821843	0,0315654	0,06664232	0,05046866	0,04931141	0,05179909	0,05289594	0,05423955	0,02265014	0,01707722	0,01600341	0,05320579	0,05222692	0,04180658	0,02358806	0,02377179
	FC5	0,0286007	0,0396984	0,02607901	0,03382195	0,03006948	0,03005981	0,02566425	0,03518777	0	0,01239793	0,03323752	0,02924044	0,04480457	0,09704402	0,02879325	0,02403231	0,03191295	0,03589505	0,01698151	0,00962297	0,01049074	0,04737914	0,02226606	0,02179396	0,0155045	0,01694881	0,00995126	0,0113511	0,01876733
	FC1	0,02304253	0,019596	0,0279282	0,01266217	0,02687832	0,02241307	0,01295187	0,05939364	0,01239793	0	0,01370138	0,01288699	0,01100134	0,01110362	0,02194591	0,03868025	0,0467654	0,0174272	0,04613423	0,04838332	0,05051672	0,01189109	0,01162103	0,02620278	0,04095473	0,03300583	0,02270687	0,02007838	0,01542787
	FC2	0,02463801	0,02673512	0,02376087	0,03399118	0,02869169	0,02660918	0,02822434	0,03621168	0,03323752	0,01370138	0	0,01971712	0,0214505	0,03446734	0,02039746	0,03310453	0,04206334	0,02660509	0,02724944	0,01958914	0,02078772	0,0168278	0,02196034	0,01494685	0,02505113	0,0202461	0,02671213	0,0300868	0,0350623
	FC6	0,02456565	0,02731126	0,02179928	0,01165186	0,01822018	0,022076	0,02242063	0,02463749	0,02924044	0,01288699	0,01971712	0	0,02286916	0,04756543	0,00418316	0,02240104	0,03772778	0,02923171	0,01961509	0,02963646	0,01799003	0,01426447	0,01733804	0,01196592	0,01859957	0,0150366	0,02886262	0,02440736	0,0156459
	T7	0,01568634	0,00948382	0,01664223	0,0271238	0,0170688	0,00986898	0,01567234	0,02247639	0,04480457	0,01100134	0,0214505	0,02286916	0	0,04375205	0,02180957	0,0181296	0,02360856	0,01369033	0,01071715	0,0222382	0,01091127	0,00350797	0,01768603	0,00808787	0,01362603	0,01126254	0,03020434	0,02730145	0,02248323
	C3	0,03196792	0,04334421	0,02939642	0,0708592	0,02807074	0,03244883	0,02845795	0,03821843	0,09704402	0,01110362	0,03446734	0,04756543	0,04375205	0	0,04843297	0,03251863	0,0408025	0,03577215	0,0213149	0,01630693	0,01785299	0,03646351	0,01930096	0,01656148	0,02620605	0,01946651	0,01305291	0,01461905	0,01251693
	Cz	0,02248909	0,0269558	0,02264187	0,00944589	0,02139091	0,02720669	0,01794876	0,0315654	0,02879325	0,02194591	0,02039746	0,00418316	0,02180957	0,04843297	0	0,02604596	0,0361618	0,02703822	0,02194613	0,02614464	0,02044448	0,01987287	0,01887022	0,01146377	0,02398629	0,02005281	0,02568315	0,02216424	0,01509587
	C4	0,02803667	0,02673168	0,02998542	0,02587288	0,02890123	0,02980954	0,01980686	0,06664232	0,02403231	0,03868025	0,03310453	0,02240104	0,0181296	0,03251863	0,02604596	0	0,05163143	0,0256938	0,04435926	0,03045389	0,02915308	0,0154644	0,0223551	0,01706735	0,03833319	0,0445572	0,02521974	0,02144774	0,02409058
	T8	0,02845448	0,02100626	0,02760332	0,02662021	0,02736186	0,01897752	0,02597847	0,05046866	0,03191295	0,0467654	0,04206334	0,03772778	0,02360856	0,0408025	0,0361618	0,05163143	0	0,02281408	0,030753	0,03712496	0,03694069	0,02293197	0,01685846	0,02521305	0,0441409	0,04823498	0,03168508	0,01416732	0,02044234
	CP5	0,005902	0,0056533	0,00723676	0,02954825	0,01911403	0,01299877	0,00358061	0,04931141	0,03589505	0,0174272	0,02660509	0,02923171	0,01369033	0,03577215	0,02703822	0,0256938	0,02281408	0	0,03360354	0,0217359	0,03039577	0,01480032	0,0189321	0,01837386	0,02296333	0,0284422	0,01684211	0,02928919	0,02766447
	CP1	0,04328967	0,03020755	0,04378379	0,01452339	0,03923612	0,01989986	0,03492521	0,05179909	0,01698151	0,04613423	0,02724944	0,01961509	0,01071715	0,0213149	0,02194613	0,04435926	0,030753	0,03360354	0	0,04506743	0,03410364	0,01132096	0,01142575	0,02289139	0,0320638	0,02917631	0,0327042	0,01825599	0,01764103
	CP2	0,02561642	0,01726056	0,02435607	0,02324307	0,02407893	0,02785593	0,01974912	0,05289594	0,00962297	0,04838332	0,01958914	0,02963646	0,0222382	0,01630693	0,02614464	0,03045389	0,03712496	0,0217359	0,04506743	0	0,01550558	0,02137983	0,03130279	0,0169473	0,03255655	0,04058785	0,00927295	0,0289028	0,03199417
	CP6	0,03838712	0,02792966	0,03283334	0,01472444	0,02676551	0,01491918	0,02757169	0,05423955	0,01049074	0,05051672	0,02078772	0,01799003	0,01091127	0,01785299	0,02044448	0,02915308	0,03694069	0,03039577	0,03410364	0,01550558	0	0,01393681	0,02465118	0,01441067	0,01736528	0,02936819	0,01868392	0,01446732	0,01741423
	P7	0,01509998	0,01147297	0,01368141	0,02042856	0,0133651	0,00798791	0,01969836	0,02265014	0,04737914	0,01189109	0,0168278	0,01426447	0,00350797	0,03646351	0,01987287	0,0154644	0,02293197	0,01480032	0,01132096	0,02137983	0,01393681	0	0,01758232	0,00823802	0,01551658	0,0117855	0,02945454	0,02800033	0,01677206
	P3	0,01810211	0,01624123	0,02135416	0,01575108	0,0165919	0,01406455	0,02215483	0,01707722	0,02226606	0,01162103	0,02196034	0,01733804	0,01768603	0,01930096	0,01887022	0,0223551	0,01685846	0,0189321	0,01142575	0,03130279	0,02465118	0,01758232	0	0,01381458	0,01853031	0,01682854	0,03622097	0,02004285	0,03308011
	Pz	0,02176639	0,0158248	0,019938	0,01586501	0,01356344	0,02318409	0,0217314	0,01600341	0,02179396	0,02620278	0,01494685	0,01196592	0,00808787	0,01656148	0,01146377	0,01706735	0,02521305	0,01837386	0,02289139	0,0169473	0,01441067	0,00823802	0,01381458	0	0,01574924	0,01556524	0,02408114	0,02384865	0,01852862
	P4	0,03391269	0,02126918	0,0319958	0,01637174	0,0298538	0,01707656	0,02667603	0,05320579	0,0155045	0,04095473	0,02505113	0,01859957	0,01362603	0,02620605	0,02398629	0,03833319	0,0441409	0,02296333	0,0320638	0,03255655	0,01736528	0,01551658	0,01853031	0,01574924	0	0,03362713	0,02747652	0,01055481	0,00921233
	P8	0,03500939	0,02715358	0,03514702	0,01131727	0,03464984	0,0193782	0,03224545	0,05222692	0,01694881	0,03300583	0,0202461	0,0150366	0,01126254	0,01946651	0,02005281	0,0445572	0,04823498	0,0284422	0,02917631	0,04058785	0,02936819	0,0117855	0,01682854	0,01556524	0,03362713	0	0,03030907	0,0183084	0,01447539
	POz	0,01914046	0,01959916	0,02403357	0,02330907	0,02773674	0,03377181	0,0243758	0,04180658	0,00995126	0,02270687	0,02671213	0,02886262	0,03020434	0,01305291	0,02568315	0,02521974	0,03168508	0,01684211	0,0327042	0,00927295	0,01868392	0,02945454	0,03622097	0,02408114	0,02747652	0,03030907	0	0,03589587	0,03704873
	O1	0,01940896	0,02253929	0,01882073	0,02414449	0,01295363	0,01648199	0,02178536	0,02358806	0,0113511	0,02007838	0,0300868	0,02440736	0,02730145	0,01461905	0,02216424	0,02144774	0,01416732	0,02928919	0,01825599	0,0289028	0,01446732	0,02800033	0,02004285	0,02384865	0,01055481	0,0183084	0,03589587	0	0,01877192
	O2	0,02845733	0,02926466	0,03009197	0,01511439	0,02989066	0,03343951	0,02968238	0,02377179	0,01876733	0,01542787	0,0350623	0,0156459	0,02248323	0,01251693	0,01509587	0,02409058	0,02044234	0,02766447	0,01764103	0,03199417	0,01741423	0,01677206	0,03308011	0,01852862	0,00921233	0,01447539	0,03704873	0,01877192	0
subject 2	Fp1	0,0123005	0,01266502	0,01093662	0,01439076	0,00979673	0,01196728	0,01182732	0,0132629	0,017121	0,01459776	0,01199432	0,01390975	0,0116071	0,01775229	0,01247957	0,01284912	0,01389842	0,01178569	0,01343453	0,01370965	0,01259412	0,01155741	0,0144575	0,0113066	0,01259758	0,01639653	0,01005712	0,01028005	0,01416036
	Fpz	0,01188231	0,01203351	0,01245764	0,01356627	0,00820884	0,01213633	0,01122153	0,01377168	0,0154534	0,01437227	0,01050562	0,01073609	0,01103131	0,01643309	0,0119164	0,01263173	0,01290519	0,01193521	0,0125844	0,01264467	0,0127246	0,01093301	0,01385734	0,00958176	0,01181586	0,01442701	0,01092005	0,00906295	0,01107505
	Fp2	0,01067093	0,01026385	0,0125745	0,01266625	0,00697717	0,00960848	0,01345819	0,01544233	0,01572794	0,0118598	0,00911409	0,00911965	0,01028288	0,01555421	0,00902543	0,01385594	0,01226063	0,00998696	0,01232618	0,01150054	0,01286116	0,00848512	0,01285388	0,01010752	0,0115515	0,01250362	0,010238	0,00709871	0,01071377
	F7	0,01144918	0,0123223	0,0108172	0,01449656	0,00884138	0,01072158	0,01187767	0,01370568	0,01685472	0,01699348	0,01143511	0,01144002	0,01213285	0,01670814	0,01272399	0,01256094	0,01295642	0,01273623	0,01403275	0,01433719	0,0127288	0,01078701	0,01478191	0,01083799	0,0119962	0,01516918	0,00991132	0,00953905	0,01403696
	F3	0,01472245	0,01407249	0,01541035	0,01164463	0,0135637	0,01522747	0,01442986	0,01796578	0,01791687	0,01351713	0,0108209	0,00849637	0,0115352	0,01443196	0,00769937	0,01368813	0,01166333	0,01426841	0,01362258	0,01203026	0,01414489	0,01129836	0,01311496	0,01359238	0,01381467	0,01895159	0,01209318	0,01533109	0,01447852
	Fz	0,01145276	0,01190976	0,01188842	0,01121506	0,00784032	0,01027821	0,01269526	0,01588989	0,01465956	0,01262862	0,00936676	0,00716523	0,01106514	0,01399184	0,00855937	0,01219599	0,01083031	0,01123845	0,00847685	0,01044033	0,01132471	0,00924656	0,01077721	0,01166493	0,01172884	0,01202021	0,00749777	0,007263	0,00985552
	F4	0,01151807	0,01096692	0,01162584	0,01070142	0,00921471	0,01173825	0,01246172	0,01731192	0,01346472	0,00990692	0,00946436	0,00875798	0,0087579	0,01220279	0,00820113	0,01361802	0,01173522	0,01064073	0,00851157	0,00937447	0,01058473	0,0080595	0,01118277	0,01266489	0,00984467	0,01213042	0,00828571	0,00654986	0,01045905
	F8	0,00908237	0,00851488	0,00848189	0,0097767	0,00817243	0,00684099	0,00947732	0,01639237	0,01313506	0,00842896	0,00852596	0,00817926	0,00870723	0,01301586	0,00951599	0,01137889	0,01281531	0,00807906	0,00956255	0,0110013	0,01029885	0,00818655	0,00773282	0,00939901	0,00891307	0,01027365	0,00845889	0,00575536	0,01066659
	FC5	0,01122201	0,01018567	0,0118844	0,01131602	0,00612681	0,00716209	0,01024225	0,01401612	0,01423871	0,01081728	0,00919604	0,00825511	0,00882388	0,01641296	0,00761842	0,0125126	0,00993411	0,00978474	0,00908355	0,0111451	0,01078711	0,00836042	0,00874837	0,00762247	0,0094727	0,01066181	0,00826434	0,00651745	0,01018399
	FC1	0,0109247	0,01243588	0,01179722	0,01346651	0,00973896	0,01095267	0,01302251	0,01719187	0,01657916	0,01231919	0,00890938	0,00904055	0,01039313	0,01389304	0,00749119	0,01143309	0,01187348	0,0122105	0,00781167	0,00963759	0,01155581	0,00918701	0,01023167	0,01244838	0,01144017	0,01443475	0,00921202	0,01002702	0,01265521
	FC2	0,00928996	0,0122079	0,01224489	0,01220167	0,00962193	0,01121931	0,01093399	0,01533639	0,01261405	0,01086481	0,01059359	0,00733232	0,00740219	0,01263586	0,00801981	0,01464833	0,01004964	0,01006951	0,00743934	0,01007827	0,01250787	0,00802395	0,01045351	0,01107028	0,00876729	0,01148142	0,00792439	0,00745773	0,00955693
	FC6	0,00942864	0,00973302	0,00898284	0,01141905	0,0072304	0,00756691	0,00946626	0,01731474	0,01257214	0,00944901	0,00752715	0,00904519	0,00931281	0,01276922	0,0098795	0,01061868	0,0105473	0,00885058	0,01008735	0,01183983	0,01041716	0,00786515	0,00823522	0,00865151	0,00925895	0,01160034	0,00852804	0,0060131	0,01261736
	T7	0,01038074	0,01160287	0,01064899	0,01252762	0,00753885	0,00773475	0,01094765	0,01474239	0,0154206	0,01002902	0,00837394	0,00866939	0,0088928	0,01677273	0,00848207	0,01140307	0,01066422	0,01086475	0,00965409	0,01014835	0,01131247	0,00996197	0,00855186	0,00752938	0,01085008	0,01048271	0,00742036	0,00710385	0,01072291
	C3	0,01039096	0,01022663	0,0109926	0,00972021	0,00876282	0,01020619	0,00977658	0,01539526	0,01486149	0,01107157	0,00938194	0,00852761	0,01015895	0,0155526	0,007858	0,01041908	0,01181397	0,0100374	0,0094158	0,01061835	0,00988807	0,00827295	0,00739872	0,00860429	0,0114082	0,01097379	0,00741776	0,00887286	0,01240928
	Cz	0,01100631	0,01088428	0,01159842	0,01187723	0,01040013	0,00807643	0,01018207	0,01560802	0,01316661	0,01113467	0,00936149	0,00779741	0,00645402	0,0133269	0,00825779	0,01117336	0,01153201	0,009577	0,00898435	0,01058123	0,01108308	0,00488842	0,01036397	0,01120254	0,00943771	0,01277355	0,00701274	0,00632255	0,01237854
	C4	0,01138904	0,01144768	0,01112529	0,008557	0,00905833	0,01146769	0,01058397	0,01225786	0,01263231	0,01047808	0,00946697	0,00644797	0,00756003	0,01374611	0,00754041	0,01271088	0,00795154	0,01157706	0,00889363	0,01043683	0,01160215	0,00858556	0,00985674	0,00966522	0,00990871	0,00989005	0,00830303	0,01043967	0,00804876
	T8	0,00934888	0,00822205	0,0076064	0,01130719	0,00863891	0,00699248	0,00933428	0,01361056	0,01327568	0,00959972	0,00880524	0,00904963	0,01046122	0,01379073	0,01023602	0,00993857	0,01301683	0,00778014	0,00878826	0,01030377	0,01105914	0,00904112	0,00737009	0,00831193	0,00885853	0,01077542	0,00804741	0,0070088	0,01074381
	CP5	0,00843215	0,00813936	0,00959697	0,00863776	0,00739625	0,00850018	0,00814403	0,01339937	0,01184933	0,01011435	0,00936952	0,00825884	0,00805583	0,01456209	0,00927313	0,0110636	0,01150587	0,00847186	0,00957451	0,01026005	0,0111694	0,00812622	0,00683295	0,00797527	0,01252389	0,01062872	0,00762725	0,00858392	0,01063003
	CP1	0,01293767	0,01044761	0,01248551	0,01153533	0,01244826	0,00949615	0,01157288	0,01303668	0,01392833	0,00996844	0,00969077	0,00945846	0,01031984	0,01489709	0,00775833	0,01016026	0,0109932	0,01028568	0,00968956	0,00824865	0,0110509	0,00866202	0,01100476	0,00998727	0,00977289	0,01179136	0,00862952	0,00964293	0,01173238
	CP2	0,01169927	0,01071929	0,01425785	0,01096907	0,01063361	0,01144714	0,01200089	0,01374224	0,01382892	0,01080662	0,01177137	0,00779492	0,00779195	0,01145366	0,00792622	0,01321072	0,01010968	0,01001239	0,00981916	0,01045823	0,01305234	0,00668072	0,01257364	0,01020808	0,01054966	0,01303671	0,00920376	0,00785646	0,00865986
	CP6	0,01161218	0,01270982	0,01305565	0,01079584	0,01041881	0,01081738	0,01076787	0,01420669	0,01471619	0,00936525	0,0106181	0,0077934	0,00748967	0,01368318	0,00737872	0,01443918	0,01159947	0,01145195	0,00968975	0,01112027	0,0120148	0,00720543	0,01278781	0,00998647	0,00951371	0,01209702	0,00911594	0,01064746	0,0079474
	P7	0,00816326	0,00769116	0,00930873	0,00834272	0,00745529	0,0081636	0,00900659	0,0129041	0,01040046	0,00949902	0,00977951	0,00834759	0,00456858	0,01230127	0,00825661	0,01053528	0,01212898	0,00710622	0,00945781	0,00994015	0,01145058	0,00571893	0,00756184	0,00848835	0,01147862	0,01036927	0,00743372	0,00889652	0,00929461
	P3	0,00865606	0,0070056	0,0092665	0,00563334	0,0083632	0,00783076	0,00993467	0,01239321	0,01006428	0,00847846	0,00846384	0,00631708	0,00469901	0,01242518	0,00895655	0,00916089	0,01347139	0,00720069	0,00991179	0,00976099	0,01092736	0,00487537	0,00731291	0,01000403	0,0089312	0,00959816	0,00652579	0,00832526	0,00890234
	Pz	0,01195562	0,01011365	0,01276005	0,00996209	0,00696575	0,00861349	0,01070227	0,01409461	0,01327852	0,01201801	0,00847362	0,0097922	0,0096484	0,01626203	0,00865629	0,01230164	0,01262994	0,00952507	0,01130888	0,01122271	0,01114812	0,008042	0,00904553	0,00635122	0,01255289	0,01214751	0,00719635	0,00656456	0,01117731
	P4	0,01111024	0,01160614	0,01284158	0,01042702	0,00945875	0,0097829	0,01267084	0,01472923	0,0148838	0,01151826	0,0101949	0,0078588	0,00738871	0,01415924	0,00980677	0,01397783	0,01208822	0,01097646	0,01086179	0,011289	0,01310288	0,00648592	0,01019713	0,00954724	0,011138	0,0133474	0,00934961	0,00911938	0,00855864
	P8	0,01154107	0,00965955	0,0098775	0,00899633	0,00581974	0,00791577	0,01164898	0,01552947	0,01539926	0,01103671	0,00838657	0,0076708	0,00887864	0,01452154	0,00779304	0,01218127	0,01274652	0,00999938	0,01099927	0,01040151	0,01150808	0,00836471	0,00799281	0,00952022	0,00994104	0,01410848	0,00721043	0,00753125	0,00733533
	POz	0,0112685	0,01037257	0,01046231	0,00949041	0,00786919	0,00954131	0,00996574	0,01392305	0,01049949	0,01109344	0,00949676	0,00864396	0,00842273	0,01550547	0,00871989	0,01104575	0,00966972	0,00903263	0,00882287	0,01125998	0,01233812	0,00872704	0,00802362	0,00832161	0,01154602	0,01056438	0,0077544	0,00893765	0,00946218
	O1	0,01175667	0,01008555	0,00996443	0,00944654	0,0068754	0,00732228	0,01238419	0,01574589	0,01530808	0,00921399	0,00765031	0,0081431	0,00881967	0,01420316	0,00823246	0,01285435	0,01293599	0,01003446	0,00880399	0,01026345	0,01061009	0,00850224	0,00884081	0,00961273	0,00952153	0,01255214	0,00882237	0,007453	0,00787486
	O2	0,00955999	0,00910624	0,01082387	0,01191501	0,00499076	0,00810536	0,01023304	0,01431743	0,01399822	0,01037473	0,00873476	0,01039954	0,00958403	0,01628275	0,00924607	0,0121412	0,0111578	0,00864412	0,0100108	0,01096034	0,01180677	0,00980405	0,008732	0,00928817	0,01264859	0,01165794	0,00766966	0,00633083	0,00915029

subject 2

		Fp1	Fpz	Fp2	F7	F3	Fz	F4	F8	FC5	FC1	FC2	FC6	T7	C3	Cz	C4	T8	CP5	CP1	CP2	CP6	P7	P3	Pz	P4	P8	POz	O1	O2
subject 1	Fp1	0,0123005	0,01188231	0,01067093	0,01144918	0,01472245	0,01145276	0,01151807	0,00908237	0,01122201	0,0109247	0,00928996	0,00942864	0,01038074	0,01039096	0,01100631	0,01138904	0,00934888	0,00843215	0,01293767	0,01169927	0,01161218	0,00816326	0,00865606	0,01195562	0,01111024	0,01154107	0,0112685	0,01175667	0,00955999
	Fpz	0,01266502	0,01203351	0,01026385	0,0123223	0,01407249	0,01190976	0,01096692	0,00851488	0,01018567	0,01243588	0,0122079	0,00973302	0,01160287	0,01022663	0,01088428	0,01144768	0,00822205	0,00813936	0,01044761	0,01071929	0,01270982	0,00769116	0,0070056	0,01011365	0,01160614	0,00965955	0,01037257	0,01008555	0,00910624
	Fp2	0,01093662	0,01245764	0,0125745	0,0108172	0,01541035	0,01188842	0,01162584	0,00848189	0,0118844	0,01179722	0,01224489	0,00898284	0,01064899	0,0109926	0,01159842	0,01112529	0,0076064	0,00959697	0,01248551	0,01425785	0,01305565	0,00930873	0,0092665	0,01276005	0,01284158	0,0098775	0,01046231	0,00996443	0,01082387
	F7	0,01439076	0,01356627	0,01266625	0,01449656	0,01164463	0,01121506	0,01070142	0,0097767	0,01131602	0,01346651	0,01220167	0,01141905	0,01252762	0,00972021	0,01187723	0,008557	0,01130719	0,00863776	0,01153533	0,01096907	0,01079584	0,00834272	0,00563334	0,00996209	0,01042702	0,00899633	0,00949041	0,00944654	0,01191501
	F3	0,00979673	0,00820884	0,00697717	0,00884138	0,0135637	0,00784032	0,00921471	0,00817243	0,00612681	0,00973896	0,00962193	0,0072304	0,00753885	0,00876282	0,01040013	0,00905833	0,00863891	0,00739625	0,01244826	0,01063361	0,01041881	0,00745529	0,0083632	0,00696575	0,00945875	0,00581974	0,00786919	0,0068754	0,00499076
	Fz	0,01196728	0,01213633	0,00960848	0,01072158	0,01522747	0,01027821	0,01173825	0,00684099	0,00716209	0,01095267	0,01121931	0,00756691	0,00773475	0,01020619	0,00807643	0,01146769	0,00699248	0,00850018	0,00949615	0,01144714	0,01081738	0,0081636	0,00783076	0,00861349	0,0097829	0,00791577	0,00954131	0,00732228	0,00810536
	F4	0,01182732	0,01122153	0,01345819	0,01187767	0,01442986	0,01269526	0,01246172	0,00947732	0,01024225	0,01302251	0,01093399	0,00946626	0,01094765	0,00977658	0,01018207	0,01058397	0,00933428	0,00814403	0,01157288	0,01200089	0,01076787	0,00900659	0,00993467	0,01070227	0,01267084	0,01164898	0,00996574	0,01238419	0,01023304
	F8	0,0132629	0,01377168	0,01544233	0,01370568	0,01796578	0,01588989	0,01731192	0,01639237	0,01401612	0,01719187	0,01533639	0,01731474	0,01474239	0,01539526	0,01560802	0,01225786	0,01361056	0,01339937	0,01303668	0,01374224	0,01420669	0,0129041	0,01239321	0,01409461	0,01472923	0,01552947	0,01392305	0,01574589	0,01431743
	FC5	0,017121	0,0154534	0,01572794	0,01685472	0,01791687	0,01465956	0,01346472	0,01313506	0,01423871	0,01657916	0,01261405	0,01257214	0,0154206	0,01486149	0,01316661	0,01263231	0,01327568	0,01184933	0,01392833	0,01382892	0,01471619	0,01040046	0,01006428	0,01327852	0,0148838	0,01539926	0,01049949	0,01530808	0,01399822
	FC1	0,01459776	0,01437227	0,0118598	0,01699348	0,01351713	0,01262862	0,00990692	0,00842896	0,01081728	0,01231919	0,01086481	0,00944901	0,01002902	0,01107157	0,01113467	0,01047808	0,00959972	0,01011435	0,00996844	0,01080662	0,00936525	0,00949902	0,00847846	0,01201801	0,01151826	0,01103671	0,01109344	0,00921399	0,01037473
	FC2	0,01199432	0,01050562	0,00911409	0,01143511	0,0108209	0,00936676	0,00946436	0,00852596	0,00919604	0,00890938	0,01059359	0,00752715	0,00837394	0,00938194	0,00936149	0,00946697	0,00880524	0,00936952	0,00969077	0,01177137	0,0106181	0,00977951	0,00846384	0,00847362	0,0101949	0,00838657	0,00949676	0,00765031	0,00873476
	FC6	0,01390975	0,01073609	0,00911965	0,01144002	0,00849637	0,00716523	0,00875798	0,00817926	0,00825511	0,00904055	0,00733232	0,00904519	0,00866939	0,00852761	0,00779741	0,00644797	0,00904963	0,00825884	0,00945846	0,00779492	0,0077934	0,00834759	0,00631708	0,0097922	0,0078588	0,0076708	0,00864396	0,0081431	0,01039954
	T7	0,0116071	0,01103131	0,01028288	0,01213285	0,0115352	0,01106514	0,0087579	0,00870723	0,00882388	0,01039313	0,00740219	0,00931281	0,0088928	0,01015895	0,00645402	0,00756003	0,01046122	0,00805583	0,01031984	0,00779195	0,00748967	0,00456858	0,00469901	0,0096484	0,00738871	0,00887864	0,00842273	0,00881967	0,00958403
	C3	0,01775229	0,01643309	0,01555421	0,01670814	0,01443196	0,01399184	0,01220279	0,01301586	0,01641296	0,01389304	0,01263586	0,01276922	0,01677273	0,0155526	0,0133269	0,01374611	0,01379073	0,01456209	0,01489709	0,01145366	0,01368318	0,01230127	0,01242518	0,01626203	0,01415924	0,01452154	0,01550547	0,01420316	0,01628275
	Cz	0,01247957	0,0119164	0,00902543	0,01272399	0,00769937	0,00855937	0,00820113	0,00951599	0,00761842	0,00749119	0,00801981	0,0098795	0,00848207	0,007858	0,00825779	0,00754041	0,01023602	0,00927313	0,00775833	0,00792622	0,00737872	0,00825661	0,00895655	0,00865629	0,00980677	0,00779304	0,00871989	0,00823246	0,00924607
	C4	0,01284912	0,01263173	0,01385594	0,01256094	0,01368813	0,01219599	0,01361802	0,01137889	0,0125126	0,01143309	0,01464833	0,01061868	0,01140307	0,01041908	0,01117336	0,01271088	0,00993857	0,0110636	0,01016026	0,01321072	0,01443918	0,01053528	0,00916089	0,01230164	0,01397783	0,01218127	0,01104575	0,01285435	0,0121412
	T8	0,01389842	0,01290519	0,01226063	0,01295642	0,01166333	0,01083031	0,01173522	0,01281531	0,00993411	0,01187348	0,01004964	0,0105473	0,01066422	0,01181397	0,01153201	0,00795154	0,01301683	0,01150587	0,0109932	0,01010968	0,01159947	0,01212898	0,01347139	0,01262994	0,01208822	0,01274652	0,00966972	0,01293599	0,0111578
	CP5	0,01178569	0,01193521	0,00998696	0,01273623	0,01426841	0,01123845	0,01064073	0,00807906	0,00978474	0,0122105	0,01006951	0,00885058	0,01086475	0,0100374	0,009577	0,01157706	0,00778014	0,00847186	0,01028568	0,01001239	0,01145195	0,00710622	0,00720069	0,00952507	0,01097646	0,00999938	0,00903263	0,01003446	0,00864412
	CP1	0,01343453	0,0125844	0,01232618	0,01403275	0,01362258	0,00847685	0,00851157	0,00956255	0,00908355	0,00781167	0,00743934	0,01008735	0,00965409	0,0094158	0,00898435	0,00889363	0,00878826	0,00957451	0,00968956	0,00981916	0,00968975	0,00945781	0,00991179	0,01130888	0,01086179	0,01099927	0,00882287	0,00880399	0,0100108
	CP2	0,01370965	0,01264467	0,01150054	0,01433719	0,01203026	0,01044033	0,00937447	0,0110013	0,0111451	0,00963759	0,01007827	0,01183983	0,01014835	0,01061835	0,01058123	0,01043683	0,01030377	0,01026005	0,00824865	0,01045823	0,01112027	0,00994015	0,00976099	0,01122271	0,011289	0,01040151	0,01125998	0,01026345	0,01096034
	CP6	0,01259412	0,0127246	0,01286116	0,0127288	0,01414489	0,01132471	0,01058473	0,01029885	0,01078711	0,01155581	0,01250787	0,01041716	0,01131247	0,00988807	0,01108308	0,01160215	0,01105914	0,0111694	0,0110509	0,01305234	0,0120148	0,01145058	0,01092736	0,01114812	0,01310288	0,01150808	0,01233812	0,01061009	0,01180677
	P7	0,01155741	0,01093301	0,00848512	0,01078701	0,01129836	0,00924656	0,0080595	0,00818655	0,00836042	0,00918701	0,00802395	0,00786515	0,00996197	0,00827295	0,00488842	0,00858556	0,00904112	0,00812622	0,00866202	0,00668072	0,00720543	0,00571893	0,00487537	0,008042	0,00648592	0,00836471	0,00872704	0,00850224	0,00980405
	P3	0,0144575	0,01385734	0,01285388	0,01478191	0,01311496	0,01077721	0,01118277	0,00773282	0,00874837	0,01023167	0,01045351	0,00823522	0,00855186	0,00739872	0,01036397	0,00985674	0,00737009	0,00683295	0,01100476	0,01257364	0,01278781	0,00756184	0,00731291	0,00904553	0,01019713	0,00799281	0,00802362	0,00884081	0,008732
	Pz	0,0113066	0,00958176	0,01010752	0,01083799	0,01359238	0,01166493	0,01266489	0,00939901	0,00762247	0,01244838	0,01107028	0,00865151	0,00752938	0,00860429	0,01120254	0,00966522	0,00831193	0,00797527	0,00998727	0,01020808	0,00998647	0,00848835	0,01000403	0,00635122	0,00954724	0,00952022	0,00832161	0,00961273	0,00928817
	P4	0,01259758	0,01181586	0,0115515	0,0119962	0,01381467	0,01172884	0,00984467	0,00891307	0,0094727	0,01144017	0,00876729	0,00925895	0,01085008	0,0114082	0,00943771	0,00990871	0,00885853	0,01252389	0,00977289	0,01054966	0,00951371	0,01147862	0,0089312	0,01255289	0,011138	0,00994104	0,01154602	0,00952153	0,01264859
	P8	0,01639653	0,01442701	0,01250362	0,01516918	0,01895159	0,01202021	0,01213042	0,01027365	0,01066181	0,01443475	0,01148142	0,01160034	0,01048271	0,01097379	0,01277355	0,00989005	0,01077542	0,01062872	0,01179136	0,01303671	0,01209702	0,01036927	0,00959816	0,01214751	0,0133474	0,01410848	0,01056438	0,01255214	0,01165794
	POz	0,01005712	0,01092005	0,010238	0,00991132	0,01209318	0,00749777	0,00828571	0,00845889	0,00826434	0,00921202	0,00792439	0,00852804	0,00742036	0,00741776	0,00701274	0,00830303	0,00804741	0,00762725	0,00862952	0,00920376	0,00911594	0,00743372	0,00652579	0,00719635	0,00934961	0,00721043	0,0077544	0,00882237	0,00766966
	O1	0,01028005	0,00906295	0,00709871	0,00953905	0,01533109	0,007263	0,00654986	0,00575536	0,00651745	0,01002702	0,00745773	0,0060131	0,00710385	0,00887286	0,00632255	0,01043967	0,0070088	0,00858392	0,00964293	0,00785646	0,01064746	0,00889652	0,00832526	0,00656456	0,00911938	0,00753125	0,00893765	0,007453	0,00633083
	O2	0,01416036	0,01107505	0,01071377	0,01403696	0,01447852	0,00985552	0,01045905	0,01066659	0,01018399	0,01265521	0,00955693	0,01261736	0,01072291	0,01240928	0,01237854	0,00804876	0,01074381	0,01063003	0,01173238	0,00865986	0,0079474	0,00929461	0,00890234	0,01117731	0,00855864	0,00733533	0,00946218	0,00787486	0,00915029
subject 2	Fp1	0	0,00085331	0,00366674	0,00125795	0,04495619	0,03964453	0,04329592	0,02981651	0,03582966	0,03882193	0,04723049	0,04035176	0,03102674	0,03463321	0,04251634	0,04684739	0,02311152	0,03043392	0,03166402	0,04678772	0,03819798	0,02413914	0,02090633	0,0289292	0,03171534	0,03010043	0,01988103	0,03233946	0,01278472
	Fpz	0,00085331	0	0,00136366	0,00078716	0,0433462	0,04235431	0,03882789	0,0213436	0,03708356	0,04072596	0,04740264	0,0327202	0,02823379	0,03701977	0,04587978	0,04062332	0,02072036	0,0308776	0,03478598	0,03978329	0,03730502	0,02062855	0,01992429	0,02526982	0,027352	0,02576735	0,01804549	0,02970886	0,01075494
	Fp2	0,00366674	0,00136366	0	0,00186984	0,04060532	0,04516487	0,04513935	0,01497871	0,02743846	0,03923661	0,04574508	0,02860196	0,02314276	0,03587298	0,04241243	0,04558958	0,01880913	0,02342885	0,02757472	0,03713289	0,03339158	0,01891995	0,01709197	0,02196523	0,02390046	0,01926714	0,01522689	0,02468535	0,00793686
	F7	0,00125795	0,00078716	0,00186984	0	0,04702722	0,03947849	0,04038732	0,02656557	0,03913568	0,03701345	0,04486575	0,03481482	0,03278874	0,04047517	0,0492798	0,04168932	0,01885312	0,03089115	0,03874871	0,04261049	0,03445236	0,02353319	0,01963152	0,02819023	0,02994992	0,02658774	0,01806311	0,03035565	0,01085303
	F3	0,04495619	0,0433462	0,04060532	0,04702722	0	0,02496997	0,02299859	0,04423748	0,05170693	0,01290089	0,03211951	0,04732961	0,05091856	0,01753109	0,03761301	0,04535203	0,03589534	0,03467604	0,02541434	0,05217218	0,04294759	0,02409373	0,01774347	0,0473392	0,04184721	0,04354371	0,04078927	0,03932681	0,0375836
	Fz	0,03964453	0,04235431	0,04516487	0,03947849	0,02496997	0	0,00750617	0,03638015	0,02712539	0,0050409	0,0138412	0,03103592	0,02903177	0,01310024	0,00444284	0,04452272	0,03619196	0,02652943	0,01590767	0,02593651	0,02997898	0,01759198	0,01397311	0,02752867	0,03198591	0,03461157	0,03225688	0,03180584	0,03406056
	F4	0,04329592	0,03882789	0,04513935	0,04038732	0,02299859	0,00750617	0	0,04445931	0,029769	0,00878014	0,00614483	0,04066369	0,03680139	0,01851745	0,0078338	0,03181821	0,04177622	0,02363977	0,01870569	0,02351693	0,03210338	0,01397688	0,01108246	0,02882032	0,03514443	0,03488832	0,03175365	0,03429117	0,03706897
	F8	0,02981651	0,0213436	0,01497871	0,02656557	0,04423748	0,03638015	0,04445931	0	0,01249175	0,04023888	0,04019063	0,00346029	0,01528509	0,03498745	0,03211229	0,03324604	0,00308644	0,02608226	0,03021858	0,04194634	0,0416773	0,02148863	0,01905832	0,01684307	0,03441255	0,00721589	0,02169226	0,00646518	0,01569729
	FC5	0,03582966	0,03708356	0,02743846	0,03913568	0,05170693	0,02712539	0,029769	0,01249175	0	0,04160705	0,02617666	0,0177194	0,00241601	0,03250955	0,01763199	0,04236174	0,01736139	0,01488354	0,01674648	0,02558778	0,03126377	0,0142728	0,01397321	0,00485644	0,0221175	0,020119	0,00793255	0,02363062	0,00976582
	FC1	0,03882193	0,04072596	0,03923661	0,03701345	0,01290089	0,0050409	0,00878014	0,04023888	0,04160705	0	0,02030604	0,03605653	0,0408392	0,01087466	0,01730617	0,04140141	0,03421301	0,02630239	0,01875359	0,0349456	0,03650837	0,01867474	0,01547443	0,03529263	0,03953672	0,04272156	0,03666859	0,03540482	0,0388261
	FC2	0,04723049	0,04740264	0,04574508	0,04486575	0,03211951	0,0138412	0,00614483	0,04019063	0,02617666	0,02030604	0	0,03934884	0,03188045	0,02520309	0,00696597	0,02197343	0,03838368	0,02117995	0,02215695	0,01066597	0,02115404	0,01244943	0,01008596	0,02419005	0,02401819	0,03099241	0,0326108	0,03686141	0,0316637
	FC6	0,04035176	0,0327202	0,02860196	0,03481482	0,04732961	0,03103592	0,04066369	0,00346029	0,0177194	0,03605653	0,03934884	0	0,01814192	0,03308042	0,03164611	0,03079607	0,00935214	0,02467758	0,02827178	0,0470497	0,04385531	0,02217185	0,01811592	0,01956392	0,03557199	0,00981688	0,02254446	0,00559864	0,01790722
	T7	0,03102674	0,02823379	0,02314276	0,03278874	0,05091856	0,02903177	0,03680139	0,01528509	0,00241601	0,0408392	0,03188045	0,01814192	0	0,0322667	0,02383898	0,03387774	0,01781642	0,01027904	0,01425577	0,02748336	0,02967904	0,011471	0,01215155	0,00447611	0,02307121	0,02511569	0,00439577	0,02727043	0,00584445
	C3	0,03463321	0,03701977	0,03587298	0,04047517	0,01753109	0,01310024	0,01851745	0,03498745	0,03250955	0,01087466	0,02520309	0,03308042	0,0322667	0	0,02015335	0,0338173	0,02900705	0,02861439	0,01499511	0,02849755	0,02482665	0,02285662	0,02071889	0,02867269	0,02926265	0,03460776	0,03412891	0,03103364	0,04237683
	Cz	0,04251634	0,04587978	0,04241243	0,0492798	0,03761301	0,00444284	0,0078338	0,03211229	0,01763199	0,01730617	0,00696597	0,03164611	0,02383898	0,02015335	0	0,04026526	0,03333238	0,02491024	0,02129958	0,01111825	0,0182282	0,01619386	0,01416465	0,02147288	0,02438526	0,02539774	0,02626532	0,02380463	0,03171966
	C4	0,04684739	0,04062332	0,04558958	0,04168932	0,04535203	0,04452272	0,03181821	0,03324604	0,04236174	0,04140141	0,02197343	0,03079607	0,03387774	0,0338173	0,04026526	0	0,02747774	0,01796934	0,01949129	0,02288177	0,01650269	0,0096158	0,00683538	0,03363073	0,03320165	0,04717394	0,02612008	0,04288076	0,03446283
	T8	0,02311152	0,02072036	0,01880913	0,01885312	0,03589534	0,03619196	0,04177622	0,00308644	0,01736139	0,03421301	0,03838368	0,00935214	0,01781642	0,02900705	0,03333238	0,02747774	0	0,02145924	0,03536025	0,04248635	0,04785756	0,0186191	0,01602514	0,01660368	0,04143362	0,01105437	0,02057542	0,00543167	0,01696291
	CP5	0,03043392	0,0308776	0,02342885	0,03089115	0,03467604	0,02652943	0,02363977	0,02608226	0,01488354	0,02630239	0,02117995	0,02467758	0,01027904	0,02861439	0,02491024	0,01796934	0,02145924	0	0,01471259	0,02162734	0,01628929	0,00236038	0,005582	0,01149387	0,01606292	0,02062133	0,00672754	0,020571	0,01429141
	CP1	0,03166402	0,03478598	0,02757472	0,03874871	0,02541434	0,01590767	0,01870569	0,03021858	0,01674648	0,01875359	0,02215695	0,02827178	0,01425577	0,01499511	0,02129958	0,01949129	0,03536025	0,01471259	0	0,01703728	0,00990138	0,01639993	0,02065457	0,01581099	0,00715706	0,02990649	0,02007433	0,03467923	0,02153007
	CP2	0,04678772	0,03978329	0,03713289	0,04261049	0,05217218	0,02593651	0,02351693	0,04194634	0,02558778	0,0349456	0,01066597	0,0470497	0,02748336	0,02849755	0,01111825	0,02288177	0,04248635	0,02162734	0,01703728	0	0,00499081	0,01384324	0,01055476	0,02252724	0,0075767	0,03433956	0,02639592	0,03590114	0,03087317
	CP6	0,03819798	0,03730502	0,03339158	0,03445236	0,04294759	0,02997898	0,03210338	0,0416773	0,03126377	0,03650837	0,02115404	0,04385531	0,02967904	0,02482665	0,0182282	0,01650269	0,04785756	0,01628929	0,00990138	0,00499081	0	0,00729172	0,00751326	0,02577656	0,00932723	0,03628186	0,02350547	0,04319276	0,0275582
	P7	0,02413914	0,02062855	0,01891995	0,02353319	0,02409373	0,01759198	0,01397688	0,02148863	0,0142728	0,01867474	0,01244943	0,02217185	0,011471	0,02285662	0,01619386	0,0096158	0,0186191	0,00236038	0,01639993	0,01384324	0,00729172	0	0,00140744	0,010716	0,00901706	0,01463301	0,00701897	0,01302096	0,01398102
	P3	0,02090633	0,01992429	0,01709197	0,01963152	0,01774347	0,01397311	0,01108246	0,01905832	0,01397321	0,01547443	0,01008596	0,01811592	0,01215155	0,02071889	0,01416465	0,00683538	0,01602514	0,005582	0,02065457	0,01055476	0,00751326	0,00140744	0	0,01288783	0,00857183	0,00955666	0,00906134	0,0114981	0,01261616
	Pz	0,0289292	0,02526982	0,02196523	0,02819023	0,0473392	0,02752867	0,02882032	0,01684307	0,00485644	0,03529263	0,02419005	0,01956392	0,00447611	0,02867269	0,02147288	0,03363073	0,01660368	0,01149387	0,01581099	0,02252724	0,02577656	0,010716	0,01288783	0	0,02069162	0,02526826	0,01008531	0,02639557	0,00605007
	P4	0,03171534	0,027352	0,02390046	0,02994992	0,04184721	0,03198591	0,03514443	0,03441255	0,0221175	0,03953672	0,02401819	0,03557199	0,02307121	0,02926265	0,02438526	0,03320165	0,04143362	0,01606292	0,00715706	0,0075767	0,00932723	0,00901706	0,00857183	0,02069162	0	0,02124237	0,02165319	0,02755448	0,02237773
	P8	0,03010043	0,02576735	0,01926714	0,02658774	0,04354371	0,03461157	0,03488832	0,00721589	0,020119	0,04272156	0,03099241	0,00981688	0,02511569	0,03460776	0,02539774	0,04717394	0,01105437	0,02062133	0,02990649	0,03433956	0,03628186	0,01463301	0,00955666	0,02526826	0,02124237	0	0,02419404	0,00494017	0,0219089
	POz	0,01988103	0,01804549	0,01522689	0,01806311	0,04078927	0,03225688	0,03175365	0,02169226	0,00793255	0,03666859	0,0326108	0,02254446	0,00439577	0,03412891	0,02626532	0,02612008	0,02057542	0,00672754	0,02007433	0,02639592	0,02350547	0,00701897	0,00906134	0,01008531	0,02165319	0,02419404	0	0,02550789	0,00612421
	O1	0,03233946	0,02970886	0,02468535	0,03035565	0,03932681	0,03180584	0,03429117	0,00646518	0,02363062	0,03540482	0,03686141	0,00559864	0,02727043	0,03103364	0,02380463	0,04288076	0,00543167	0,020571	0,03467923	0,03590114	0,04319276	0,01302096	0,0114981	0,02639557	0,02755448	0,00494017	0,02550789	0	0,02922543
	O2	0,01278472	0,01075494	0,00793686	0,01085303	0,0375836	0,03406056	0,03706897	0,01569729	0,00976582	0,0388261	0,0316637	0,01790722	0,00584445	0,04237683	0,03171966	0,03446283	0,01696291	0,01429141	0,02153007	0,03087317	0,0275582	0,01398102	0,01261616	0,00605007	0,02237773	0,0219089	0,00612421	0,02922543	0
